# Diarylmethane synthesis through Re_2_O_7_-catalyzed bimolecular dehydrative Friedel–Crafts reactions†
†Electronic supplementary information (ESI) available: Experimental protocols and spectral data. See DOI: 10.1039/c8sc03570a


**DOI:** 10.1039/c8sc03570a

**Published:** 2018-09-13

**Authors:** Qi Qin, Youwei Xie, Paul E. Floreancig

**Affiliations:** a School of Chemistry and Chemical Engineering , Huazhong University of Science and Technology , Wuhan 430074 , P. R. China . Email: xieyw@hust.edu.cn; b Department of Chemistry , University of Pittsburgh , Pittsburgh , Pennsylvania 15260 , USA . Email: florean@pitt.edu

## Abstract

This manuscript describes the application of Re_2_O_7_ to the syntheses of diarylmethanes from benzylic alcohols through solvolysis followed by Friedel–Crafts alkylation. The reactions are characterized by broad substrate scope, low catalyst loadings, high chemical yields, and minimal waste generation. The intermediate perrhenate esters are superior leaving groups to chlorides and bromides in these reactions. The polarity and water sequestering capacity of hexafluoroisopropyl alcohol are critical to the success of these processes. Re_2_O_7_ is a precatalyst for HOReO_3_, which serves as a less costly and easily handled promoter for these reactions. Oxorhenium catalysts selectively activate alcohols in the presence of similarly substituted acetates, indicating a unique chemoselectivity and mechanism in comparison to Brønsted acid catalysis.

## Abstract 2

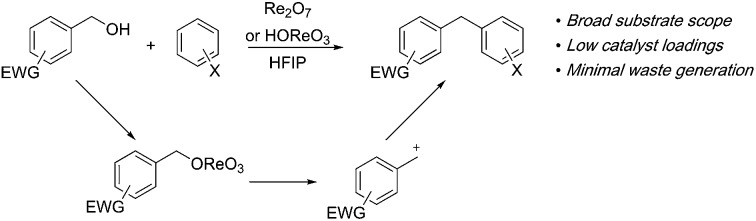
This manuscript describes the application of Re_2_O_7_ to the syntheses of diarylmethanes from benzylic alcohols through solvolysis followed by Friedel–Crafts alkylation.

## Introduction

A report in 2007 from the American Chemical Society Green Chemistry Institute Pharmaceutical Roundtable Research Grant Program identified “OH activation for nucleophilic substitution” as a process for which reactions are currently used but better reagents are preferred.^1^ This can be achieved by transiently enhancing the hydroxy group's nucleofugacity through association with an electrophilic catalyst. The benefit of this approach to substitution chemistry lies in the avoidance of a distinct step to generate a leaving group and through the generation of water as an environmentally benign by-product. A number of recent reviews have highlighted the benefits of dehydrative coupling reactions in complex molecule synthesis.^2^ However, alcohol activation to generate minimally-stabilized carbocations remains rare. The Hall group made a notable advance in this area by showing^3^ that benzylic alcohols bearing electron-withdrawing groups such as –CF_3_ or –CO_2_Me ionize efficiently in the presence of a ferrocenyl boronic acid catalyst in (CF3)_2_CHOH (HFIP) and CH_3_NO_2_ to form benzylic cations that react with arenes in Friedel–Crafts reactions. The Moran group subsequently showed^4^ that previously inaccessible benzylic cations can be formed through TfOH-mediated benzylic alcohol ionization in HFIP at elevated temperatures.

Our studies in the applications of Re_2_O_7_ to allylic alcohol transposition reactions^5^,^6^ resulted in a new dehydrative cyclization protocol in which allylic cations react with pendent hydroxy groups.^7^,^8^ Re_2_O_7_ proved to be superior to Brønsted acids for promoting these processes, leading us to speculate that it could be an effective catalyst for enabling bimolecular dehydrative coupling reactions. This manuscript describes the realization of this objective by demonstrating that Re_2_O_7_ acts as an exceptional catalyst for converting benzylic alcohols to carbocations. This serves as an entry to efficient, green Friedel–Crafts reactions with broad substrate scope at low catalyst loadings, which is another transformation in the “reactions are currently used but better reagents are preferred” list. Experiments highlight the role of HFIP in promoting efficient reactivity and define the reactivity of perrhenate as a leaving group in ionization processes.

## Results and discussion

**Scheme 1:**
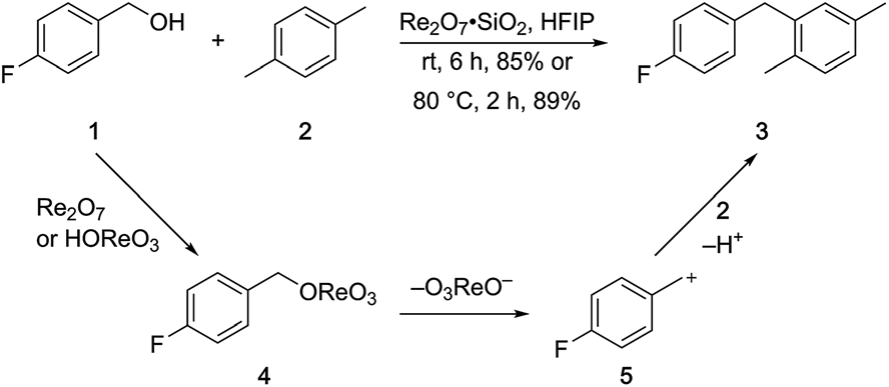
Re_2_O_7_-mediated dehydrative Friedel–Crafts alkylation.

*p*-Fluorobenzyl alcohol (**1**) served as the initial substrate for this study (Scheme 1) since a fluoro group (*σ*_p_ = +0.06)^9^ will provide modest destabilization for the intermediate benzylic cation. Exposing **1** to 10% (w/w) Re_2_O_7_·SiO_2_ (ref. 5c) (1 mol%) and *p*-xylene (3 equiv.) in HFIP (0.5 M) provided diarylmethane **3** in 85% yield within 6 h at rt. Re_2_O_7_·SiO_2_ was used as the catalyst rather than crystalline Re_2_O_7_ because it can be measured more easily for reactions in which low quantities of catalyst are required. Conducting the reaction at 80 °C resulted in the reaction being complete within 2 h while delivering **3** in 89% yield. A reasonable pathway for this process involves the conversion of **1** to perrhenate ester **4** by reaction with Re_2_O_7_ or HOReO_3_. The ionization of **4** to form cation **5** followed by the addition of **2** yields diarylmethane **3**. The weak nucleophilicity arenes and the substituent effects shown below strongly implicate an ionization-based mechanism.

**Fig. 1:**
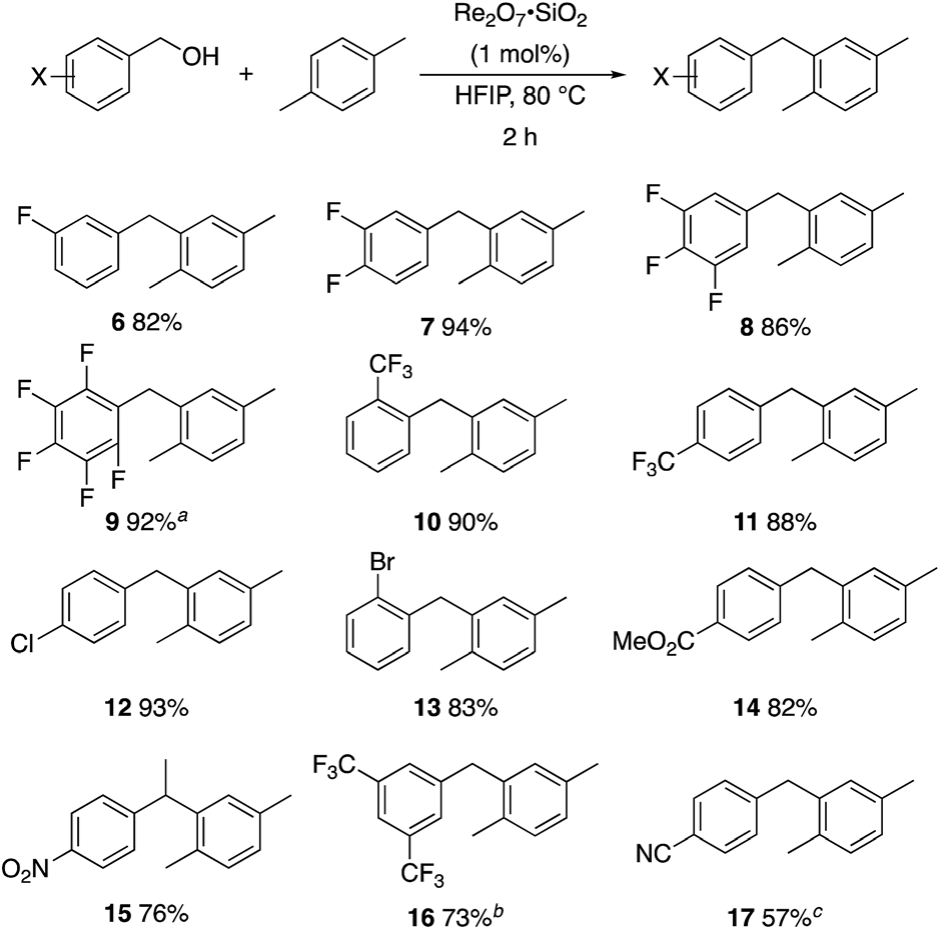
Representative scope of the dehydrative diarylmethane synthesis. ^*a*^Reaction was conducted at 100 °C for 24 h. The product was isolated in 95% yield when the reaction was conducted at 80 °C for 5 h with crystalline Re_2_O_7_. ^*b*^Reaction was conducted with crystalline Re_2_O_7_ for 23 h. ^*c*^Reaction was conducted with crystalline Re_2_O_7_ for 48 h.

A partial scope of the process is shown in Fig. 1. These transformations use *p*-xylene as the nucleophile (3 equiv.), HFIP as the solvent (used as supplied from the source), and Re_2_O_7_·SiO_2_ as the catalyst (1 mol%) with the alcohol concentration being 0.5 M. The reactions were conducted in a sealed vial at 80 °C unless otherwise noted (oil bath temperature) and were stopped after 2 h without monitoring progress.

The reaction is effective for regioisomeric fluorobenzylic alcohols and polyfluorinated substrates. This is noteworthy because of the strong inductive cation destabilization displayed by *m*-fluoro groups (*σ*_μ_ = +0.34). Pentafluorobenzyl alcohol reacts somewhat more sluggishly but it can be converted to the diarylmethane efficiently at slightly higher temperature and over a longer reaction time. Other inductively electron-withdrawing moieties, such as trifluoromethyl groups (*σ*_p_ = +0.54) do not suppress ionization. Chloro and bromo groups (*σ*_p_ = +0.23) are also tolerated in the processes. Groups that moderately destabilize the intermediate cation through conjugation, such as the carbomethoxy group (*σ*_p_ = +0.45), do not suppress ionization. *p*-Cyanobenzyl alcohol is not a suitable substrate for this process under the standard conditions (*σ*_p_ = +0.66 for the cyano group), but can be employed if crystalline Re_2_O_7_ is employed rather than Re_2_O_7_·SiO_2_. We postulate that the SiO_2_, while facilitating reagent handling, provides a buffering effect that diminishes reactivity. Crystalline Re_2_O_7_ can also be used to effect ionization of 3,5-bis-trifluoromethylbenzyl alcohol (*σ*_μ_ = +0.43 for the CF_3_ group), though this reaction requires heating for 2 d to achieve a moderate product yield. *p*-Nitrobenzyl alcohol (*σ*_p_ = +0.78 for the nitro group) reacts very slowly (39% after 2 d at 100 °C, not shown), though *p*-nitrophenethyl alcohol reacts smoothly under the standard reaction conditions due to the cation stabilizing effect of the additional methyl group. Small amounts of dialkylated products are also formed in these reactions though generally in less than 10% yield.

**Scheme 2:**
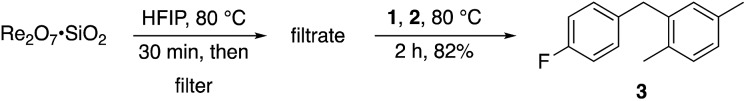
Demonstration of catalyst release from SiO_2_.

A study of initial reaction rates showed that Re_2_O_7_·SiO_2_ is slightly less reactive than Re_2_O_7_ in promoting reactions with most substrates. Our prior studies on the use of Re_2_O_7_·SiO_2_,5*c* which is simply prepared from stirring Re_2_O_7_ with SiO_2_ in Et_2_O followed by solvent evaporation, showed that the reclaimed catalyst was less potent in subsequent transformations. This indicates that catalytic activity arises from Re_2_O_7_ leaching from the SiO_2_ surface. This was tested (Scheme 2) by stirring Re_2_O_7_·SiO_2_ in HFIP at 80 °C for 30 min and removing the silica gel by filtration (PTFE filter). Compounds **1** and **2** were then added to the filtrate to generate **3** in 82% yield, indicating that Re_2_O_7_ is released from SiO_2_ into solution to a sufficient degree to promote the reaction of a moderately reactive substrate. This behavior is distinct from agents in which Re_2_O_7_ is grafted onto oxygenated surfaces at high temperature.^10^ We therefore postulate that the difference in reactivity between crystalline Re_2_O_7_ and Re_2_O_7_·SiO_2_ arises from the lower concentrations of the relevant catalyst in solution due to incomplete release from or readsorption onto the SiO_2_ surface.

We next studied the reactivity of various simple arene nucleophiles in this reaction. This study utilized pentafluorobenzyl alcohol (**18**) as a relatively unreactive substrate. The results are shown in Table 1. Reaction rates generally correlated with expected nucleophilicity trends. Thus benzene reacts efficiently but significantly more slowly than *p*-xylene. Increasing the number of methyl groups leads to faster reactions. Reactions with nucleophiles that have more than one reactive site lead to regioisomeric product mixtures. Naphthalene also serves as a competent nucleophile, with reaction rates that are comparable to methylated benzene derivatives. Anisole reacts at a rate that is comparable to toluene, despite the superior electron donating ability of the methoxy group in comparison to a methyl group. We postulate that the HFIP forms hydrogen bonds to the oxygen of the methoxy group, thereby inhibiting its capacity to act as an electron donor. Regiocontrol was not high in these processes, as expected for electrophilic aromatic substitution reactions with extremely reactive electrophiles.

**Table 1:** Reactivity comparison of simple arene nucleophiles^*a*^

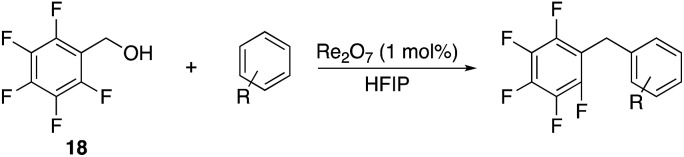			
Entry	Product	Yield^*b*^	Temp, *t*
1	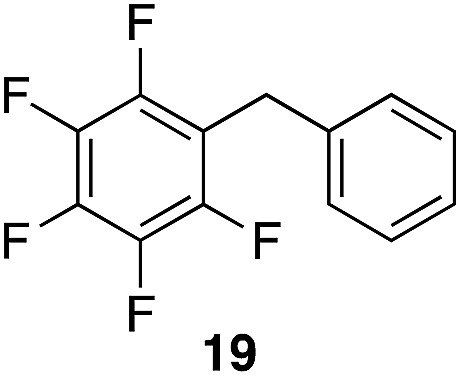	92%	100 °C, 72 h
2	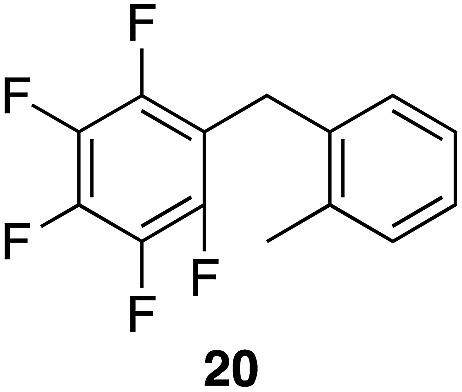	99%, 1.8 : 1.0 : 1.7, *o* : *m* : *p*	80 °C, 24 h
3	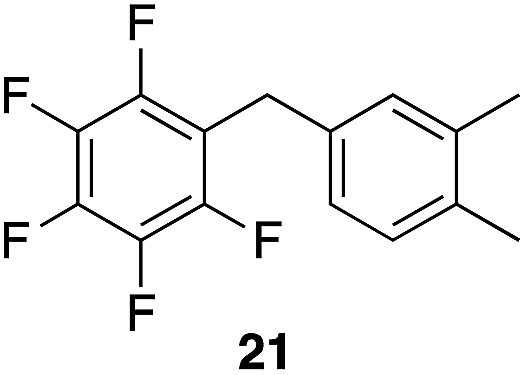	97%, 3 : 2, 1,2,4 : 1,2,3	80 °C, 5 h
4	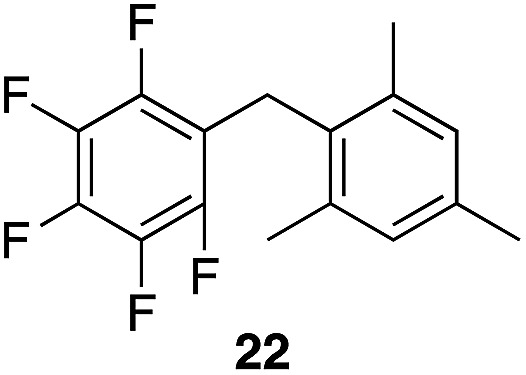	99%	80 °C, 4 h
5	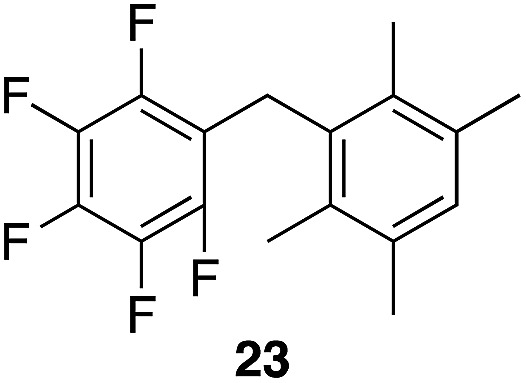	97%	80 °C, 4 h
6	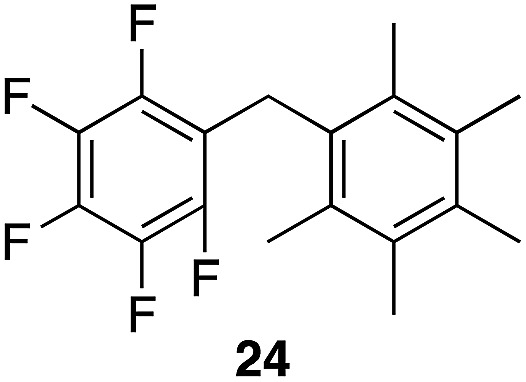	94%	80 °C, 4 h
7	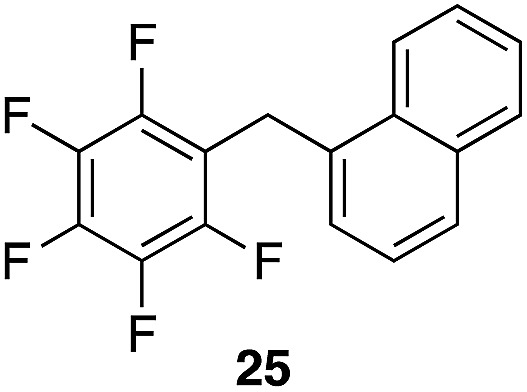	97%, 3 : 1, α : β	80 °C, 4 h
8	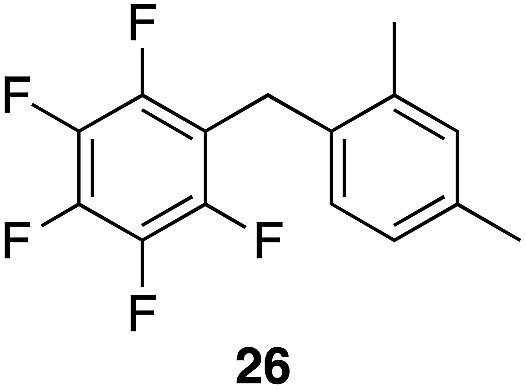	97%, 8.4 : 2.0 : 1.0, 1,2,4 : 1,2,6 : 1,3,5	80 °C, 5 h
9	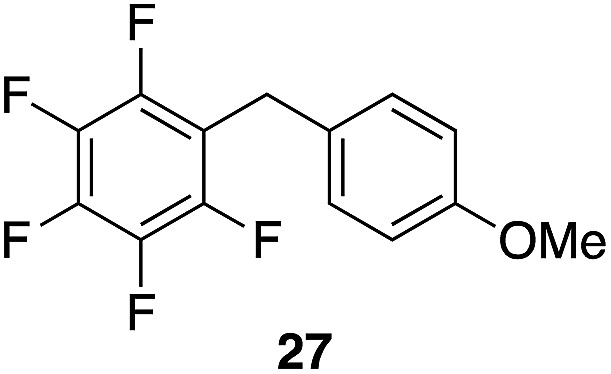	98%, 2.8 : 1, *p* : *o*	80 °C, 24 h

^*a*^Reactions proceeded with pentafluorobenzyl alcohol (1 equiv.), the nucleophilic arene (3–5 equiv.), and crystalline Re_2_O_7_ in HFIP at the indicated temperatures and for the indicated times.

^*b*^Yields refer to purified materials. Ratios were determined by ^1^H NMR.

The high electrophilicity of the intermediate carbocations in these processes led us to explore haloarene nucleophiles (Table 2). Haloarenes were predominantly used as the nucleophiles in these reactions due to the capacity to convert the products to other materials through cross-coupling reactions and because of the established benefits of incorporating fluorine into pharmaceutical agents.^11^ The reactions with weaker nucleophiles were somewhat less efficient, due to increased polyalkylation resulting from the formation of products that are less sterically hindered than those derived from *p*-xylene, regioisomeric mixtures were produced,^12^ as expected for Friedel–Crafts reactions on halobenzenes, with fluorobenzene being more selective for *para*-additions than the other halobenzenes. Despite these issues the reactions delivered useful yields of the desired products. Mayr proposed that arene nucleophilicity correlates with *σ*^+^ Hammett values.^13^ The success of fluoro- (*σ*^+^ = –0.07, entries 1, 4, and 5), chloro- (*σ*^+^ = +0.12), bromo- (*σ*^+^ = +0.15), and iodo- (*σ*^+^ = +0.14) benzenes, coupled with our observation of only trace amounts of the adduct with methyl benzoate (*σ*^+^ = +0.32) by LC-MS (not shown), suggest that the *σ*^+^ cut-off for substituents to show useful reactivity is approximately +0.2. Regiocontrol can be dictated by avoiding steric interactions, as seen in the selective formation of **33** (entry 8).^12^ The capacity to remove the *tert*-butyl group through retro Friedel–Crafts chemistry^14^ make this a potentially powerful approach to preparing *ortho*-disubstituted products.^15^

**Table 2:** Expanding the nucleophile scope^*a*^

Entry	Arene	Alcohol	Product	Yield^*b*^, *p* : *o*
1	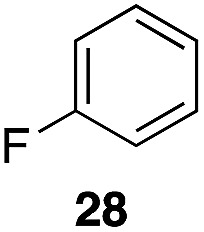	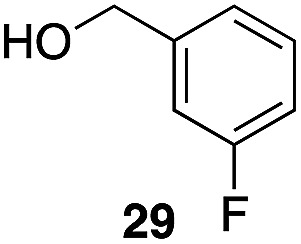	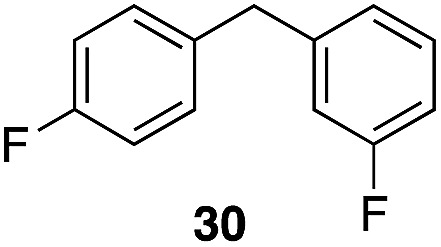	40%, 1.6 : 1
2	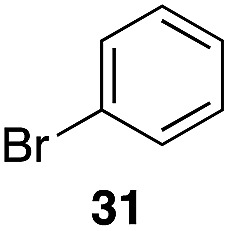	29	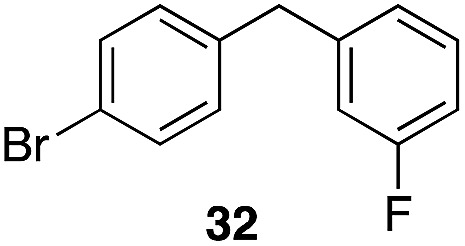	46%, 1 : 1
3	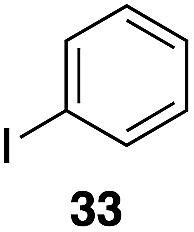	29	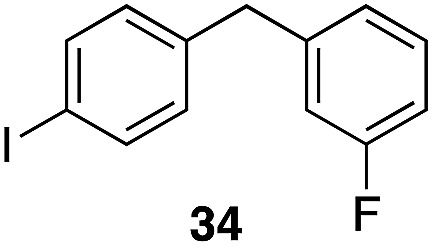	62%, 1.2 : 1
4	28	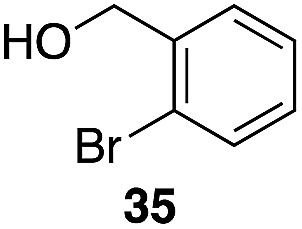	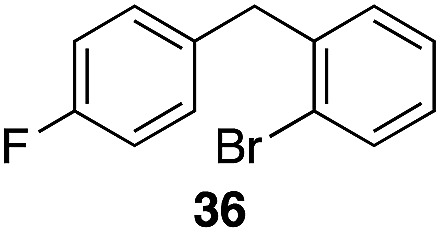	55%, 2.8 : 1
5	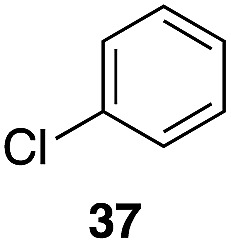	35	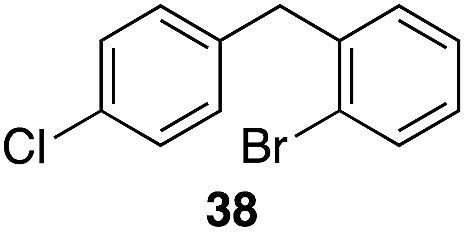	56%, 2.2 : 1
6	31	1	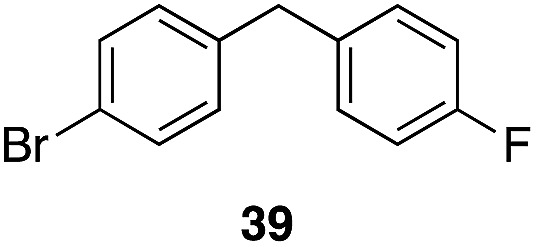	56%, 2 : 1
7	31	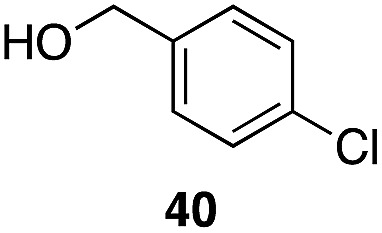	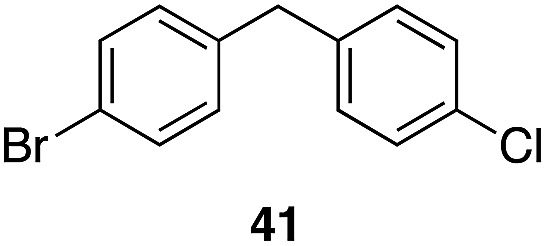	53%, 1.8 : 1
8	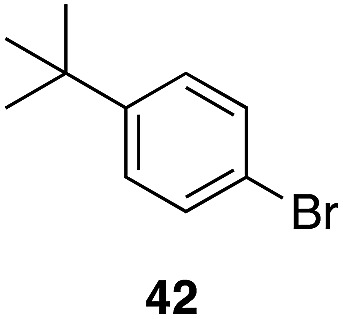	29	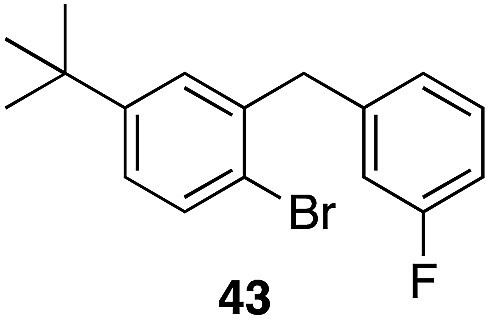	43%, 1 : 0

^*a*^All reactions were run with 10% Re_2_O_7_·SiO_2_ (1 mol%) in HFIP (0.5 M) with three equiv. of the nucleophile.

^*b*^Isolated yields of regioisomeric mixtures. Ratios were determined by ^1^H NMR.

**Scheme 3:**
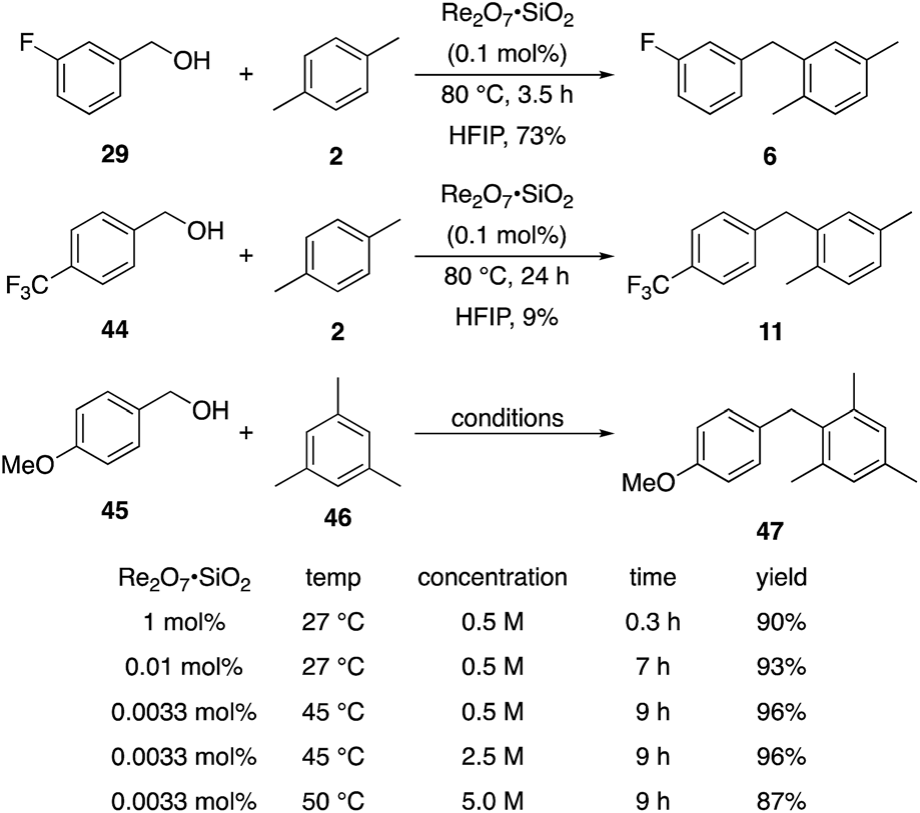
Reactions at lower catalyst loading.

We next tested whether the catalyst loading could be lowered below 1 mol% (Scheme 3). Initial experiments using 0.1 mol% Re_2_O_7_·SiO_2_ showed that *m*-fluorobenzyl alcohol reacted with *p*-xylene to yield **6** in 73% yield after 3.5 h. The less reactive substrate *p*-trifluoromethyl benzyl alcohol (**44**), however, only provided diarylmethane **11** in 9% yield after 24 h. These results indicate that success with very low catalyst loading is dependent upon the ease of carbocation formation. This led us to investigate the limits of catalyst loading with a substrate that readily undergoes ionization and an active nucleophile. The reaction of *p*-methoxybenzyl alcohol (**45**) with mesitylene (**46**) proceeds to completion in <20 min at rt to yield **47** in 90% yield in the presence of 1 mol% Re_2_O_7_·SiO_2_, in accord with predictions about the influence of cation stability on reaction rates. Lowering the catalyst loading by a factor of 100 (0.01 mol%) resulted in the reaction proceeding to complete conversion after 7 h at rt and a 93% isolated yield of **47**. Lowering the catalyst loading by another factor of 3 (0.0033 mol%, 33 ppm) resulted in the need for gentle heating, with the reaction requiring 9 h at 45 °C to proceed to completion with a 96% yield of **47**. The concentration of the reaction was increased in consideration of large scale reactions being the likely applications of extremely low catalyst loading. Conducting the reaction at 2.5 M and 45 °C with 33 ppm Re_2_O_7_ provided results that were essentially identical to those from the corresponding reaction at 0.5 M. Notably, increasing the concentration further to 5.0 M led to a slightly less efficient process, presumably due to the change in solvent properties as the reaction approaches being solvent free, and dialkylation became a minor competitive pathway. Although HFIP is not an inherently green solvent,^16^ the capacity to conduct these reactions at high concentrations and removing the solvent through distillation without an aqueous work-up improves the process mass intensity^17^ and mitigates concerns of using a halogenated solvent for these processes.

**Scheme 4:**
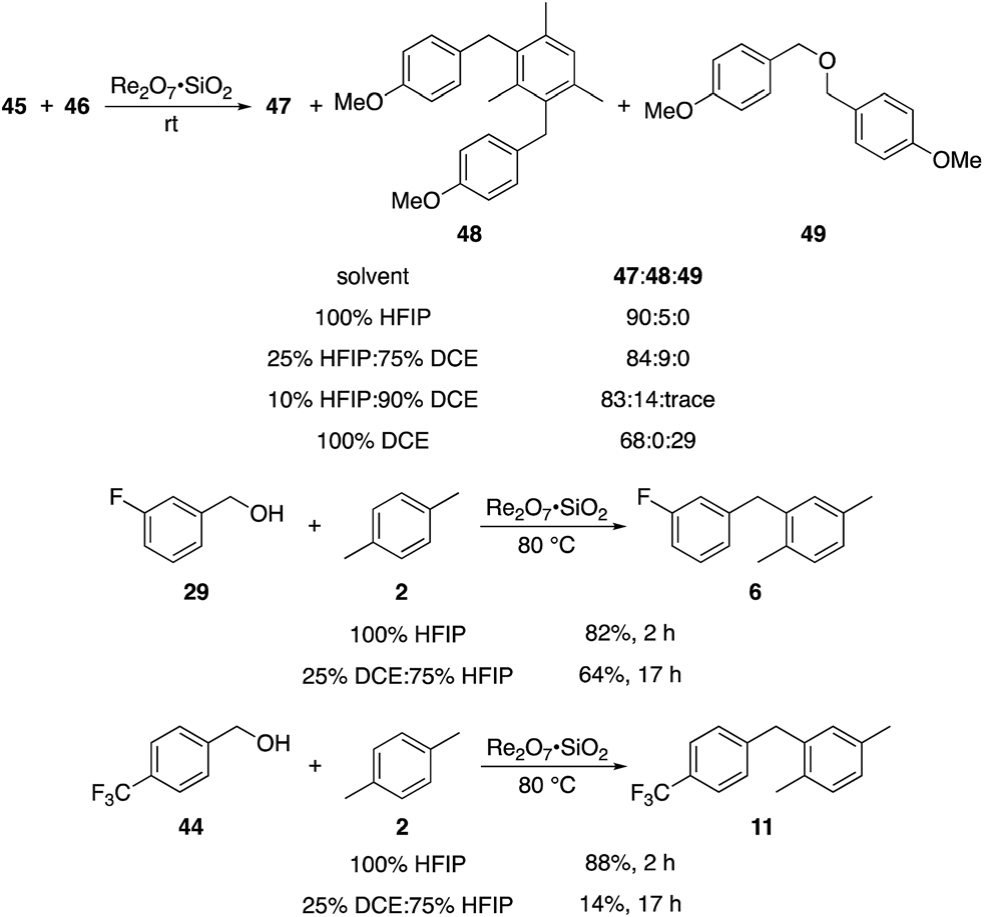
Solvent effects.

The concentration studies led us to study the role of HFIP in promoting the transformation. Ghorai and co-workers showed^18^ in related processes that Re_2_O_7_-mediated Friedel–Crafts reactions with highly activated benzylic alcohols in CH_3_CN are significantly slower than the reactions that we have studied, indicating that the efficiencies of our processes can be attributed to the solvent. The roles of fluorinated alcohols in promoting chemical reactions is a topic of significant interest, and has been reviewed thoroughly.^19^ HFIP is an extremely polar solvent as determined by solvatochromism studies.^20^ This can be attributed to its exceptional capacity as a hydrogen bond donor.^21^ HFIP is also a highly effective sequestering agent for H_2_O,^22^ which can have a strong influence on equilibrium concentrations of the intermediate benzylic cations. We conducted the reaction of highly reactive alcohol **45** with **46** in solvent mixtures containing varying fractions of HFIP to determine its role in promoting the coupling reactions (Scheme 4). We observed no qualitative difference in moving from 100% HFIP to 75% HFIP:25% 1,2-dichloroethane (DCE), 50% HFIP:50% DCE, and 25% HFIP:75% DCE. A reaction in 90% DCE:10% HFIP proceeded rapidly, but provided dialkylated product **48** in 14% yield in addition to 83% of **47**, indicating that this solvent system actually enhances reactivity relative to pure HFIP. Notably, conducting the reaction in pure DCE resulted in the formation of **47** in 68% yield and ether **49** in 29% yield. Moderately reactive alcohol **29** reacted with **2** in 25% DCE:75% HFIP with at 80 °C to yield **6** in 64% yield after 17 h, and somewhat unreactive alcohol **44** reacted with **2** under these conditions to yield **11** in just 14% yield. These results indicate that the polarity of HFIP is very important for reactions in which the intermediate carbocation is not highly stabilized, and slight changes in the solvent mixture can have a pronounced effect on reaction efficiency. Additionally HFIP's hydrogen bonding capacity is important for blunting the nucleophilicity of the hydroxy groups in the substrates, consistent with Mayr's studies on the nucleophilicity of HFIP/H_2_O mixtures,^23^ and eliminating dehydrative ether formation.

**Scheme 5:**
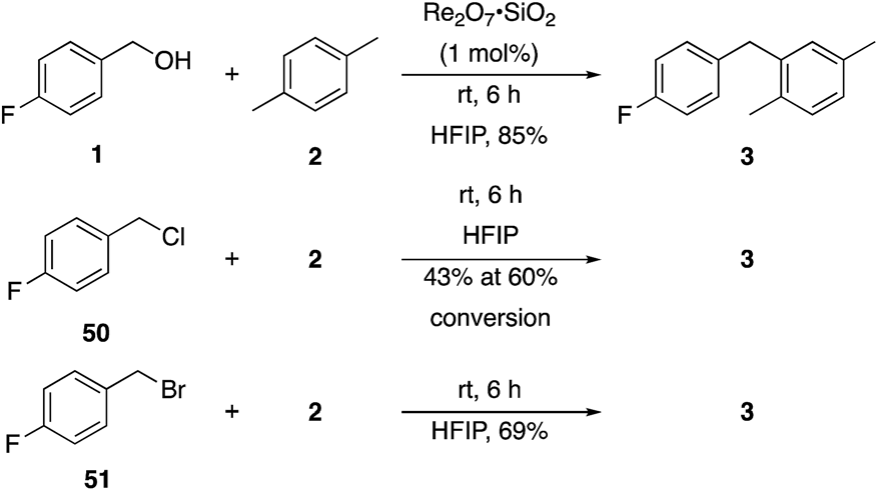
Leaving group comparison.

Additionally we conducted studies to determine whether the unusual efficiency of these reactions could be attributed to the reaction medium or to the nucleofugacity of perrhenate. The p*K*_a_ of perrhenic acid is –1.25 in water, which is significantly higher than those of HCl and HBr. Therefore carbocation formation through the loss of perrhenate should be less efficient than through the loss of chloride or bromide. We compared (Scheme 5) the Friedel–Crafts reactions of *p*-fluorobenzyl alcohol (**1**) with *p*-xylene (**2**) in the presence and absence of Re_2_O_7_·SiO_2_ to the reactions of the corresponding benzylic chloride (**50**) and bromide (**51**). The reaction of **1** with **2** in the presence of 1 mol% Re_2_O_7_·SiO_2_ in HFIP at rt, as described above, delivered diarylmethane **3** in 85% isolated yield. No reaction was observed in the absence of Re_2_O_7_·SiO_2_ or in the presence of SiO_2_, thereby confirming the need for Re_2_O_7_. Only 60% of chloride **50** was consumed after 6 h at rt, giving **3** in 43% yield. The reaction of bromide **51** with **2** in HFIP provided **3** in 69% yield after 6 h. Therefore the Re_2_O_7_-mediated conditions generate a significantly better leaving group than bromide or chloride. The rate difference is particularly striking in consideration of the low concentrations of the intermediate perrhenate esters and indicates that these species are unexpectedly effective leaving groups.

**Scheme 6:**
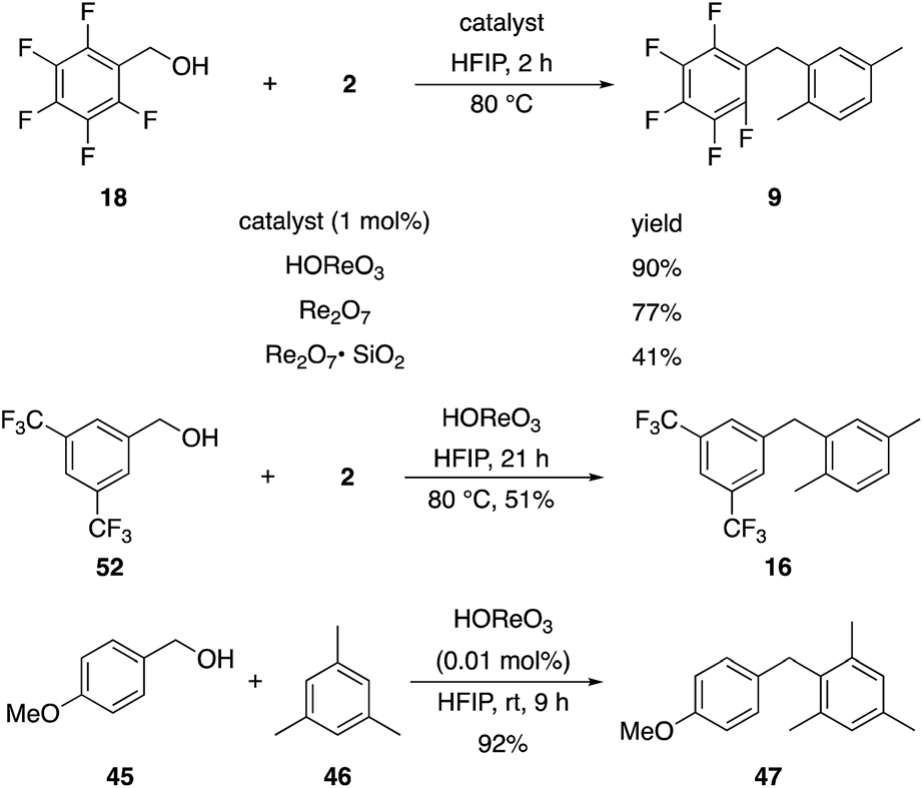
Catalyst comparison.

2,6-Di-*tert*-butylpyridine completely suppresses these reactions. This led us to speculate that HOReO_3_ is the relevant catalyst. We tested the viability of perrhenic acid (76.5 weight% in H_2_O) as a catalyst by comparing its efficacy in the coupling of the slow-reacting substrate pentafluorobenzyl alcohol (**18**) and *p*-xylene with crystalline Re_2_O_7_ and Re_2_O_7_·SiO_2_ (Scheme 6) to yield **9**. This study showed that HOReO_3_ is comparable to crystalline Re_2_O_7_ and is significantly superior to Re_2_O_7_·SiO_2_ in promoting these reactions. The highly deactivated substrate 3,5-bis-trifluoromethylbenzyl alcohol (**52**) provided a 51% yield of **16** after 23 h with 2 mol% HOReO_3_, which is somewhat less efficient than the result shown in Fig. 1 for Re_2_O_7_. Several aspects of these results are noteworthy. The loading of rhenium with HOReO_3_ is half of what is required for the Re_2_O_7_ reactions for most substrates, making its use significantly more economical since Re_2_O_7_ is approximately 5 times more expensive than HOReO_3_ on a per mole comparison.^24^ Additionally, HOReO_3_ is a liquid that can be handled readily. Thus utilizing HOReO_3_ provides the reactivity of crystalline Re_2_O_7_ and the handling ease of Re_2_O_7_·SiO_2_ while being much less expensive. We conclude based on these results and those described later that reactions with Re_2_O_7_ and HOReO_3_ most likely proceed through the same fundamental mechanism. HOReO_3_ is also an extremely effective catalyst at low loadings, with 0.01 mol% sufficing to provide **47** from **45** and **46** in 92% yield within 9 h at rt.

**Fig. 2:**
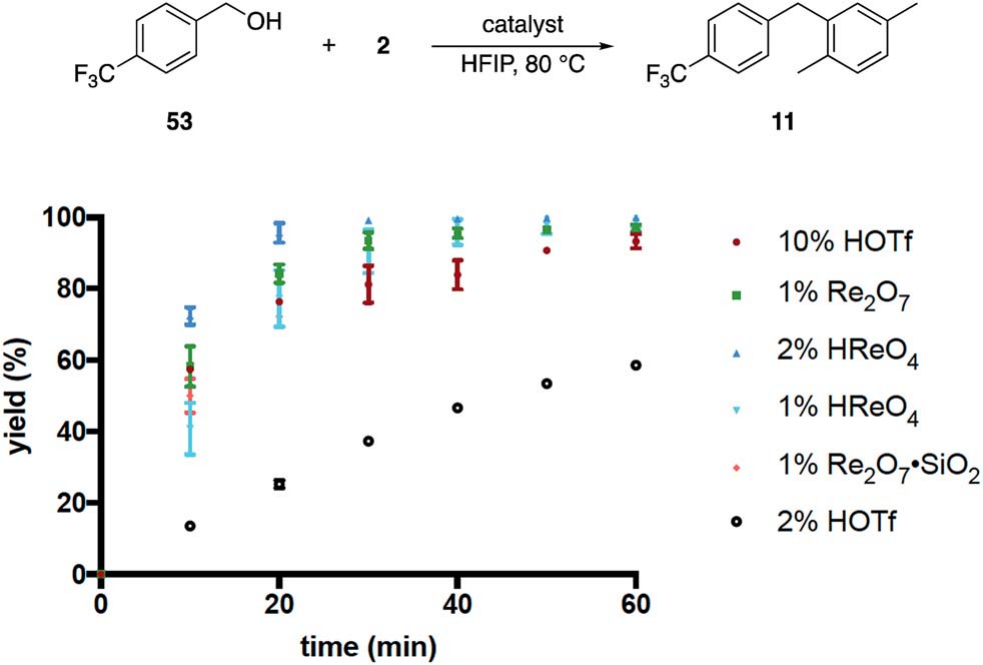
Reaction kinetics as a function of catalyst.

These results, coupled with Moran's observation of catalysis by TfOH activation,^4^ lead to questions about the mechanism of the alcohol activation. Although HOReO_3_ is a much weaker Brønsted acid (p*K*_a_ of –14 for TfOH) we cannot eliminate the possibility that HFIP causes an anomalous increase in the acidity of HOReO_3_, resulting in these reactions proceeding through protonation rather than perrhenate ester formation. We addressed this issue (Fig. 2) initially through comparing the rates of Re_2_O_7_ (1 mol%), TfOH (10 mol% and 2 mol%), HOReO_3_ (2 mol% and 1 mol%), and Re_2_O_7_·SiO_2_ (1 mol%) in the conversion of **53** to **11**. These experiments showed that 1 mol% Re_2_O_7_, 10 mol% TfOH, and 2 mol% HOReO_3_ promoted the transformation at approximately the same rate. Lowering the loading of TfOH to 2 mol% resulted in a reaction that was even slower than the Re_2_O_7_·SiO_2_ catalyzed process. Thus if the oxorhenium catalysts act solely as Brønsted acids then the HFIP environment must cause HOReO_3_ to be a stronger acid than TfOH. Notably, *p*-TsOH (p*K*_a_ = –2.8) does not promote the coupling reaction, indicating that the potential acidity enhancement by HFIP is not a general phenomenon.

**Fig. 3:**
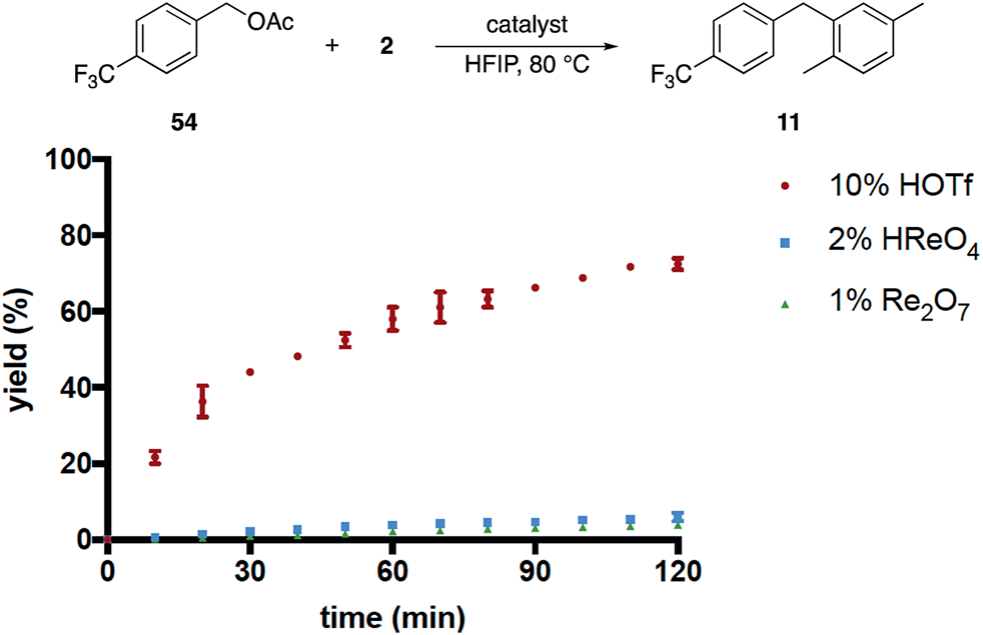
Acetate ionization by different catalysts.

We then compared the rates of HOReO_3_, Re_2_O_7_ and TfOH in the conversion of acetate **54** to **11** (Fig. 3) under the assumption that Brønsted acid catalysis will promote ionization even more effectively for this substrate relative to the corresponding alcohol while perrhenate ester formation would be suppressed. The reaction with TfOH proceeded to a significant extent within 2 h but, surprisingly, was slower than the reaction of the corresponding alcohol. We attribute this to acetic acid being sequestered less effectively than water, leading to lower concentrations of the intermediate benzylic cation. The reactions with HOReO_3_ and Re_2_O_7_, however, proceeded to <5% conversion at the same time and temperature. This result is inconsistent with a simple solvent-mediated acidity enhancement since the oxorhenium catalysts are approximately equivalent to TfOH in their abilities to promote alcohol ionization.

**Scheme 7:**
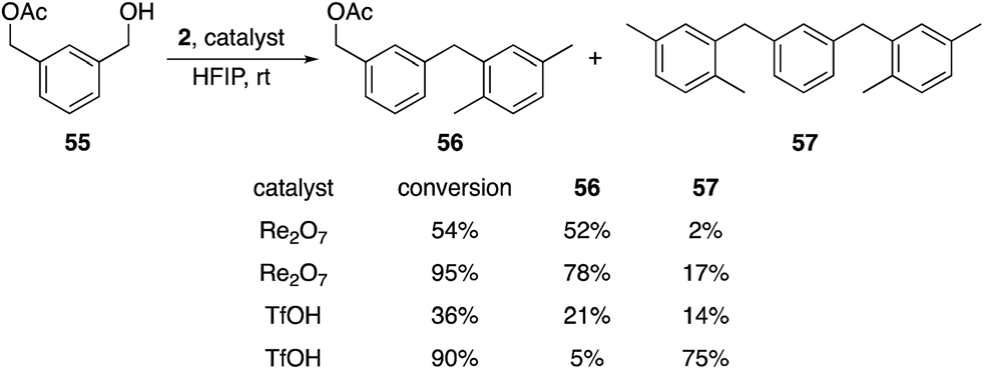
Reactions with a difunctionalized substrate.

Additionally we exposed difunctional compound **55** and **2** to Re_2_O_7_ and TfOH at partial (1 h) and nearly complete (2.25 h) conversion (Scheme 7). We observed selective formation of **56**, the product of alcohol activation, in 52% yield with Re_2_O_7_ at 54% conversion, with only 2% of the disubstitution product **57**. TfOH produced a 21% yield of **56** and a 14% yield of **47** at 36% conversion. At 95% conversion the reaction with Re_2_O_7_ provided a 78% yield of **56** and only a 17% yield of **57**, while TfOH produced a 5% yield of **56** and a 75% yield of **57** at 90% conversion. These results are consistent with Re_2_O_7_ showing a strong preference for alcohol ionization over acetate ionization while TfOH shows little to no selectivity. This system provides a very stringent challenge for the systems because the cation arising from product ionization is more stable than that derived from the starting material, as *σ*_m_ values for benzylic groups increase as the electronegativity of the substituent increases.^9^ Thus monosubstitution at high conversion is a challenging transformation that requires a selective catalyst. The absence of the monoalkylation product resulting from preferential acetate ionization suggests that this species is significantly more reactive than the starting material, which is consistent with the more positive *σ*_m_ value for the acetoxymethyl group relative to the hydroxymethyl group.^9^ This study further supports our hypothesis that the oxorhenium-catalyzed reactions do not proceed by simple alcohol protonation by a Brønsted acid with solvent-promoted enhanced acidity, and suggest that a perrhenate ester is likely to be relevant intermediate. A secondary role for a Brønsted acid cannot be discarded by our studies, however, with the highly non-basic nature of HFIP potentially allowing for protonated perrhenate esters to be the direct precursors to benzylic cations. *The heightened selectivity for activating hydroxy groups by oxorhenium catalysts, however, demonstrates an advantage for these agents over strong Brønsted acids with respect to chemoselectivity.*

## Conclusions

We have described an efficient protocol for diarylmethane synthesis through Friedel–Crafts reactions between benzene derivatives and benzylic alcohols. Re_2_O_7_, either in its crystalline form or adsorbed onto silica gel, is an exceptionally effective catalyst for these processes. The transformation has a very broad scope, including electron deficient benzylic alcohols, and requires low catalyst loading. The use of highly polar HFIP as the reaction solvent is important for enhancing rates and for minimizing the nucleophilicity of the hydroxy groups of the benzylic alcohols. The intermediates in these reactions are exceptional leaving groups, proving to be superior to chlorides and bromides. Re_2_O_7_ can be replaced by perrhenic acid to minimize cost and facilitate handling. Mechanistic studies confirmed that the mechanism of these reactions does not proceed through simple protonation, with the perrhenate ester or protonated perrhenate ester being the most likely reactive intermediate. This leads to unique chemoselectivity in which alcohols can be activated in preference to superior leaving groups. This work shows the capacity of oxorhenium species to promote important carbon–carbon bond forming reactions from readily available substrates while mitigating environmental impact.^25^

## Conflicts of interest

The authors declare no competing interests.

## Supplementary Material

Supplementary information
