# Supplementary material for: Diarylmethane synthesis through Re2O7-catalyzed bimolecular dehydrative Friedel–Crafts reactions
Source: Chem Sci. 2018 Sep 13;9(45):8528–34. doi: 10.1039/c8sc03570a (PMC6251338; doi:10.1039/c8sc03570a)
Supplement: Supplementary file 1 [file SC-009-C8SC03570A-s001.pdf]

Electronic Supplementary Material (ESI) for Chemical Science.
This journal is © The Royal Society of Chemistry 2018

Diarylmethane Synthesis through Re_2O_7-Catalyzed Bimolecular Dehydrative Friedel-Crafts Reactions

Qi Qin,[b] Youwei Xie[a]* and Paul E. Floreancig[b]*

School of Chemistry and Chemical Engineering, Huazhong University of Science and Technology, Wuhan 430074, P. R. China
and
Department of Chemistry, University of Pittsburgh, Pittsburgh, Pennsylvania 15260, USA

Table of Contents

General protocols 2

Experimental protocols for basic Friedel-Crafts reactions 3

Re_2O_7•SiO_2 release studies 13

Low catalyst loading experiments 14

Solvent studies 16

Leaving group comparison 17

Catalyst comparison 18

Kinetics studies 19

Competition studies 23

^1H, ^{13}C, and ^{19}F NMR spectra 25

General Experimental Proton (^{1}H NMR) and carbon (^{13}C NMR) nuclear magnetic resonance spectra were taken on a Bruker Avance 300 spectrometer at 300 MHz and 75 MHz respectively, a Bruker Avance 400 spectrometer at 400 MHz and 100 MHz, a Bruker Avance 500 spectrometer at 500 MHz and 125 MHz. The chemical shifts are reported in parts per million (ppm) on the delta (δ) scale. for ^{1}H NMR, tetramethylsilane in $CDCl_3$ was used as reference: TMS = 0.00 ppm. For ^{13}C NMR the solvent peak was used as a reference value: $CDCl_3$ = 77.2 ppm. For ^{19}F NMR CF_3CO_2H was used as the reference: CF_3CO_2H = –76.55 ppm. Data are reported as follows: m = multiplet, s = singlet; d = doublet; t = triplet; q = quartet; p = pentet; s = sextet; dd = doublet of doublets; dt = doublet of triplets; ddd = doublet of doublet of doublets etc. Analytical TLC was performed on E. Merck pre-coated (25 mm) silica gel 60 F_{254} plates. Visualization was done under UV (254 nm) or by staining (10 g Phosphomolybdic Acid, 90 mL absolute ethanol). Flash chromatography was done using SiliCycle SiliaFlash P60 40-63µm 60 Å silica gel. Hexafluoroisopropanol (HFIP) was purchased from Oakwood Chemicals and used directly unless otherwise specified. Reagent grade ethyl acetate, diethyl ether, acetone, dichloromethane, methanol, pentane and hexanes (commercial mixture) were purchased from Fisher Scientific and were used as-is for chromatography. For low-ppm level reaction, all the reagents were purified according to "Purification of Laboratory Chemicals Sixth edition". Mesitylene was dried with $CaCl_2$ and distilled from sodium. *p*-methoxybenzyl alcohol was purified by shaking with aqueous KOH and extracting with diethyl ether, then the extract was treated with saturated NaHS, filtered, washed and dried over CaO and then distilled under reduced pressure to obtain pure *p*-methoxybenzyl alcohol. HFIP was redistilled from 3Å molecular sieves.

Preparation of Re_2O_7•SiO_2

A slurry of SiO_2 (1.55 g) and of Re_2O_7 (172 mg) in Et_2O (10 mL) was stirred in a round bottom flask at rt for 3 h, then solvent was removed under reduced pressure. The resulting powder was dried under vacuum overnight. The catalyst was transferred to a vial, wrapped in aluminum foil, and stored in a desiccator.

General procedure A for the Re_2O_7/Re_2O_7•SiO_2 mediated Friedel-Crafts alkylation

To a solution of the substrate (0.2 mmol) and arene (0.6 mmol, 3 equiv) in HFIP (0.4 mL) was added Re_2O_7 (0.01 equiv) or Re_2O_7•SiO_2 (10% w/w, 0.01 equiv). The reaction mixture was sealed in a 1-dram vial (Chemglass CG-4904-05 with a polypropylene screw cap containing a PTFE faced silicone septum) and stirred at the indicated temperature for the indicated time. The reaction was quenched by Et_3N, then the solvent was partially removed under vacuum. The crude mixture was then purified by flash column chromatography.

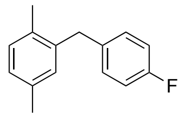

2-(4-Fluorobenzyl)-1,4-dimethylbenzene (3)

General reaction protocol A was followed with **1** (25 mg, 0.2 mmol), **2** (64 mg, 0.6 mmol), Re_2O_7•SiO_2 (9.6 mg, 0.002 mmol), and HFIP (0.4 mL). The reaction mixture was heated to 80 °C for 2 h then was quenched with Et_3N, concentrated under vacuum and purified through flash chromatography (100% hexane to 2% ethyl acetate in hexane) to give the desired product (38 mg, 89% yield). ^{1}H NMR (500 MHz, $CDCl_3$) δ 7.09–7.03 (m, 3H), 6.97-6.91 (m, 3H), 6.90 (s, 1H), 3.90 (s, 2H), 2.28 (s, 3H), 2.17 (s, 3H); ^{13}C NMR (100 MHz, $CDCl_3$) δ 161.4 (d, J = 243.6 Hz), 138.7, 136.3 (d, J = 3.3 Hz), 135.6, 133.5, 130.8, 130.4, 130.2 (d, J = 8.2 Hz), 127.4, 115.5 (d, J = 20.7 Hz), 38.8, 21.1, 19.3; ^{19}F NMR (470.4 MHz, $CDCl_3$; CF_3CO_2H – ext. std.) δ –118.8 (ddd, J = 13.8, 8.7, 5.2 Hz); HRMS (ESI) $C_{15}H_{14}F$ $[M–H]^+$: m/z calcd. 213.1074; found 213.1071.

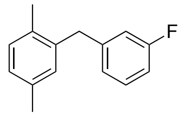

2-(3-Fluorobenzyl)-1,4-dimethylbenzene (6)

General reaction protocol A was followed with 3-fluorobenzyl alcohol (25 mg, 0.2 mmol), **2** (64 mg, 0.6 mmol), Re_2O_7•SiO_2 (9.6 mg, 0.002 mmol), and HFIP (0.4 mL). The reaction mixture was heated to 80 °C for 2 h then was quenched with Et_3N, concentrated under vacuum, and purified by flash chromatography (100% hexane to 2% ethyl acetate in hexane) to give the desired product (35 mg, 82% yield). ^{1}H NMR (500 MHz, $CDCl_3$) δ 7.21 (td, J = 7.9, 6.1 Hz, 1H), 7.05 (d, J = 7.6 Hz, 1H), 6.97 (d, J = 7.6 Hz, 1H), 6.93-6.88 (m, 2H), 6.86 (td, J = 12.7, 2.4 Hz, 1H), 6.79 (dt, J = 10.0, 2.0 Hz, 1H), 3.93 (s, 2H), 2.29 (s, 3H), 2.17 (s, 3H); ^{13}C NMR (125 MHz, $CDCl_3$) δ 163.0 (d, J = 245.3 Hz), 143.3 (d, J = 7.6 Hz), 137.8, 135.5, 133.4, 130.8, 130.3, 129.7 (d, J = 8.2 Hz), 127.4, 124.3 (d, J = 2.7 Hz), 115.5 (d, J = 20.9 Hz), 112.8 (d, J = 20.9 Hz), 39.2 (d, J = 1.1 Hz), 21.0, 19.1; ^{19}F NMR (470.4 MHz, $CDCl_3$; CF_3CO_2H – ext. std.) δ –114.6 (m); HRMS (ESI) $C_{15}H_{14}F$ $[M–H]^+$: m/z calcd. 213.1074; found 213.1071.

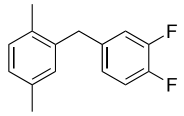

2-(3,4-Difluorobenzyl)-1,4-dimethylbenzene (7)

General reaction protocol A was followed with 3,4-difluorobenzyl alcohol (28 mg, 0.2 mmol), **2** (64 mg, 0.6 mmol), Re_2O_7•SiO_2 (9.6 mg, 0.002 mmol), and HFIP (0.4 mL). The reaction mixture was heated to 80 °C for 2 h then was quenched with Et_3N, concentrated under vacuum, and purified by flash chromatography (100% hexane to 2% ethyl acetate in hexane) to give the desired product (44 mg, 94% yield). ^{1}H NMR (500 MHz, $CDCl_3$) δ 7.07-6.94 (m, 3H), 6.91-6.78 (m, 3H), 3.88 (d, 2H), 2.29 (s, 3H), 2.15 (s, 3H); ^{13}C NMR (125 MHz, $CDCl_3$) δ 150.4 (dd, J = 247, 12.7 Hz), 148.9 (dd, J = 247, 12.7 Hz), 137.8, 137.8 (dd, J = 9.1, 3.6 Hz), 135.8, 133.5,130.8, 130.6, 127.7, 124.5 (dd, J = 6.3, 3.6 Hz), 117.5 (d, J = 17.1 Hz), 117.1 (d, J = 17.1 Hz), 38.7, 21.1, 19.2; ^{19}F NMR (470.4 MHz, $CDCl_3$; CF_3CO_2H – ext. std.) δ -139.3 (ddd, J = 20.8, 12.2, 8.6 Hz, 1F), -143.1 (dddd, J = 20.8, 10.4, 6.9, 3.5 Hz, 1F); HRMS (ESI) $C_{15}H_{13}F_2$ $[M–H]^+$: m/z calcd. 231.0980; found 231.0980.

5-(2,5-Dimethylbenzyl)-1,2,3-trifluorobenzene (8)

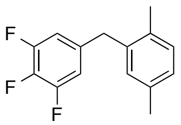

General reaction protocol A was followed with 2,3,4-trifluorobenzyl alcohol (32 mg, 0.2 mmol), **2** (64 mg, 0.6 mmol), Re_2O_7•SiO_2 (9.6 mg, 0.002 mmol), and HFIP (0.4 mL). The reaction mixture was heated to 80 °C for 2 h then was quenched with Et_3N, concentrated under vacuum, and purified by flash chromatography (100% hexane to 2% ethyl acetate in hexane) to give the desired product (44 mg, 86% yield). ^{1}H NMR (500 MHz, $CDCl_3$) δ 7.06 (d, J = 7.7 Hz, 1H), 7.00 (d, J = 7.7 Hz, 1H), 6.89 (s, 1H), 6.73-6.66 (m, 2H), 3.86 (s, 2H), 2.30 (s, 3H), 2.15 (s, 3H); ^{13}C NMR (125 MHz, $CDCl_3$) δ 151.3 (ddd, J = 248.9 Hz, 10.0, 3.6 Hz), 138.3 (dt, J = 248.9, 15.6 Hz), 137.2 (td, J = 7.0 Hz, 4.0 Hz), 137.2, 137.0, 136.0, 133.5, 130.9, 130.7, 128.0, 112.5 (dd, J = 16.3, 5.4 Hz), 38.8, 21.1, 19.2; ^{19}F NMR (470.4 MHz, $CDCl_3$; CF_3CO_2H – ext. std.) δ –135.8 (dd, J = 20.8, 8.7 Hz, 2F), –165.2 (tt, J = 20.8, 6.9 Hz, 1F); HRMS (ESI) $C_{15}H_{12}F_3$ $[M-H]^+$: m/z calcd. 249.0886; found 249.0890.

1-(2,5-Dimethylbenzyl)-2,3,4,5,6-pentafluorobenzene (9)

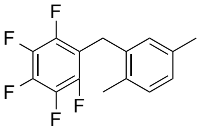

General reaction protocol A was followed with 2,3,4,5,6-pentafluorobenzyl alcohol (41 mg, 0.21 mmol), **2** (64 mg, 0.6 mmol), Re_2O_7•SiO_2 (9.6 mg, 0.002 mmol), and HFIP (0.4 mL). The reaction mixture was stirred for 24 h at 100 °C then was quenched with Et_3N, concentrated under vacuum, and purified by flash chromatography (100% hexane to 2% ethyl acetate in hexane) to give the desired product (54 mg, 92% yield). ^{1}H NMR (500 MHz, $CDCl_3$) δ 7.05 (d, J = 7.85 Hz, 1H), 6.95 (d, J = 8.20 Hz, 1H), 6.74 (s, 1H), 3.96 (s, 2H), 2.34 (s, 3H), 2.24 (s, 3H); ^{13}C NMR (125 MHz, $CDCl_3$) δ 146.4 (m), 144.5 (m), 141.1 (m), 139.1 (m), 138.8 (m), 136.8 (m), 135.9, 135.2, 133.0, 130.5, 129.1, 129.0, 127.9, 114.0 (td, J = 27.8, 4.1 Hz), 25.6, 21.1, 19.2; ^{19}F NMR (470.4 MHz, $CDCl_3$; CF_3CO_2H – ext. std.) δ –143.2 (dd, J = 22.5, 8.6 Hz, 2F), –157.9 (t, J = 20.8 Hz, 1F), –163.4 (td, J = 21.6, 8.6 Hz, 2F); HRMS (ESI) $C_{15}H_{11}F_5$ $[M]^+$: m/z calcd. 286.0781; found 286.0809.

1,4-Dimethyl-2-(2-(trifluoromethyl)benzyl)benzene (10)

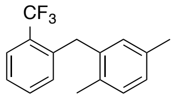

General reaction protocol A was followed with 2-trifluoromethylbenzyl alcohol (35 mg, 0.2 mmol), **2** (64 mg, 0.6 mmol), Re_2O_7•SiO_2 (9.6 mg, 0.002 mmol), and HFIP (0.4 mL). The reaction mixture was heated to 80 °C for 2 h then was quenched with Et_3N, concentrated under vacuum, and purified by flash chromatography (100% hexane to 2% ethyl acetate in hexane) to give the desired product (48 mg, 90% yield). ^{1}H NMR (500 MHz, $CDCl_3$) δ 7.67 (d, J = 7.7 Hz, 1H), 7.36 (t, J = 7.5 Hz, 1H), 7.28 (t, J = 7.5 Hz, 1H), 7.08 (d, J = 7.8 Hz, 1H), 7.00 (d, J = 7.8 Hz, 1H), 6.91 (d, J = 7.8 Hz, 1H), 6.84 (s, 1H), 4.13 (s, 2H), 2.28 (s, 3H), 2.13 (s, 3H); ^{13}C NMR (125 MHz, $CDCl_3$) δ 139.5, 137.4, 135.7, 133.8, 132.0, 131.3, 130.5, 130.4, 128.8 (q, J = 29.7 Hz), 127.6, 126.1, 126.0 (q, J = 5.8 Hz), 124.9 (q, J = 274 Hz), 35.6 (d, J = 2.9 Hz), 21.1, 19.1; ^{19}F NMR (470.4 MHz, $CDCl_3$; CF_3CO_2H – ext. std.) δ –61.6 (s); HRMS (ESI) $C_{16}H_{15}F_3$ $[M^+]$: m/z calcd. 264.1126; found 264.1147.

1,4-Dimethyl-2-(4-(trifluoromethyl)benzyl)benzene (11)

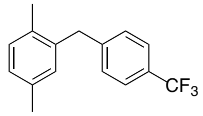

General reaction protocol A was followed with 4-trifluorobenzyl alcohol (35 mg, 0.2 mmol), **2** (64 mg, 0.6 mmol), Re_2O_7•SiO_2 (9.6 mg, 0.002 mmol), and HFIP (0.4 mL). The reaction mixture was heated to 80 °C for 2 h then was quenched with Et_3N, concentrated under vacuum and purified by flash chromatography (100% hexane to 2% ethyl acetate in hexane) to give the products (47 mg, 88% yield). ^{1}H NMR (500 MHz, $CDCl_3$) δ 7.51 (d, *J* = 8.1 Hz, 2H), 7.21 (d, *J* = 8.2 Hz, 2H), 7.06 (d, *J* = 8.0 Hz, 1H), 6.99 (d, *J* = 8.0 Hz, 1H), 6.91 (s, 1H), 3.99 (s, 2H), 2.29 (s, 3H), 2.17 (s, 3H); ^{13}C NMR (125 MHz, $CDCl_3$) δ 145.0, 137.7, 135.8, 133.6, 131.0, 130.6, 129.1, 128.5 (q, *J* = 32.4 Hz), 127.7, 125.6 (q, *J* = 3.9 Hz), 124.5 (q, *J* = 272.3 Hz), 39.4, 21.1, 19.3; ^{19}F NMR (470.4 MHz, $CDCl_3$; CF_3CO_2H – ext. std.) δ -63.2 (s); HRMS (ESI) $C_{16}H_{15}F_3$ [M^+]: *m*/*z* calcd. 264.1126; found 264.1110.

2-(4-Chlorobenzyl)-1,4-dimethylbenzene (12)

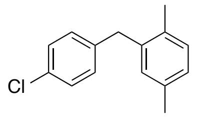

General reaction protocol A was followed with 4-chlorobenzyl alcohol (28 mg, 0.2 mmol), **2** (64 mg, 0.6 mmol), Re_2O_7•SiO_2 (9.6 mg, 0.002 mmol), and HFIP (0.4 mL). The reaction mixture was heated to 80 °C for 2 h then was quenched with Et_3N, concentrated under vacuum, and purified by flash chromatography (100% hexane to 2% ethyl acetate in hexane) to give the desired product (43 mg, 93% yield). ^{1}H NMR (500 MHz, $CDCl_3$) δ 7.24–7.20 (m, 2H), 7.06–7.01 (m, 3H), 6.97 (dd, *J* = 7.6, 1.3 Hz, 1H), 6.89 (s, 1H), 3.90 (s, 2H), 2.29 (s, 3H), 2.17 (s, 3H); ^{13}C NMR (125 MHz, $CDCl_3$) δ 139.2, 138.3, 135.7, 133.5, 131.8, 130.8, 130.5, 130.2, 128.6, 127.5, 38.9, 21.1, 19.3; HRMS (ESI) $C_{15}H_{14}Cl$ $[M-H]^+$: *m*/*z* calcd. 229.0779; found 229.0779

2-(2-Bromobenzyl)-1,4-dimethylbenzene (13)

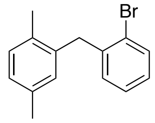

General reaction protocol A was followed with 2-bromobenzyl alcohol (37 mg, 0.2 mmol), **2** (64 mg, 0.6 mmol), Re_2O_7•SiO_2 (9.6 mg, 0.002 mmol), and HFIP (0.4 mL). The reaction mixture was heated to 80 °C for 2 h then was quenched with Et_3N, concentrated under vacuum and purified by flash chromatography (100% hexane to 2% ethyl acetate in hexane) to give the desired product (46 mg, 83% yield). ^{1}H NMR (500 MHz, $CDCl_3$) δ 7.57 (dd, *J* = 8.0, 1.1 Hz, 1H), 7.15 (td, *J* = 11.2, 1.0 Hz, 1H), 7.08 (d, *J* = 7.6 Hz, 1H), 7.05 (dd, *J* = 7.7 Hz, 1.5 Hz, 1H), 6.98 (d, *J* = 7.2 Hz, 1H), 6.84 (dd, *J* = 7.6, 1.1 Hz, 1H), 6.81 (s, 1H), 4.00 (s, 2H), 2.27 (s, 3H), 2.17 (s, 3H); ^{13}C NMR (125 MHz, $CDCl_3$) δ 140.0, 137.4, 135.7, 133.8, 132.8, 130.7, 130.4, 130.3, 127.8, 127.5, 127.5, 125.2, 39.6, 21.1, 19.2; HRMS (ESI) $C_{15}H_{14}Br$ $[M-H]^+$: *m*/*z* calcd. 273.0273; found 273.0278.

Methyl 4-(2,5-dimethylbenzyl)benzoate (14)

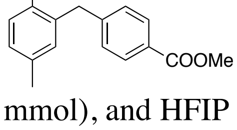

General reaction protocol A was followed with 4-carbomethoxybenzyl alcohol (33 mg, 0.2 mmol), **2** (64 mg, 0.6 mmol), Re_2O_7•SiO_2 (9.6 mg, 0.002 mmol), and HFIP (0.4 mL). The reaction mixture was heated to 80 °C for 2 h then was quenched with Et_3N, concentrated under vacuum and purified through flash chromatography (100% hexane

to 2% ethyl acetate in hexane) to give the desired product (42 mg, 82% yield). ^{1}H NMR (500 MHz, $CDCl_3$) δ 7.93 (d, *J* = 8.2 Hz, 2H), 7.18 (d, *J* = 8.2 Hz, 2H), 7.05 (d, *J* = 7.7 Hz, 1H), 6.98 (d, *J* = 8.1 Hz, 1H), 6.90 (s, 1H), 3.98 (s, 2H), 3.88 (s, 3H), 2.28 (s, 3H), 2.16 (s, 3H); ^{13}C NMR (125 MHz, $CDCl_3$) δ 143.3, 136.4, 136.0, 133.3, 131.6 (q, *J* = 32.9), 130.7 (d, *J* = 5.4 Hz), 128.7 (d, *J* = 3.3 Hz), 128.1, 124.5, 122.4, 120.2 (Sep, *J* = 3.9 Hz), 39.2, 21.0, 19.2: HRMS (ESI) $C_{17}H_{19}O_2$ $[M+H]^+$: *m/z* calcd. 255.1380; found 255.1381.

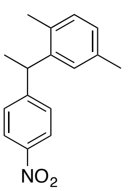

1,4-Dimethyl-2-(1-(4-nitrophenyl)ethyl)benzene (15)

General reaction protocol A was followed with 1-(4-nitrophenyl) ethanol (30 mg, 0.18 mmol), **2** (60 mg, 0.54 mmol), Re_2O_7•SiO_2 (8.7 mg, 0.002 mmol), and HFIP (0.36 mL). The reaction mixture was heated to 80 °C for 2 h then was quenched with Et_3N, concentrated under vacuum and purified by flash chromatography (100% hexane to 2% ethyl acetate in hexane) to give the desired product (35 mg, 76% yield). ^{1}H NMR (500 MHz, $CDCl_3$) δ 8.11 (dt, *J* = 9.3, 2.3 Hz, 2H), 7.30 (dt, *J* = 9.3, 2.5 Hz), 7.06-7.00 (m, 2H), 6.98 (d, *J* = 7.3 Hz, 1H), 4.38 (q, *J* = 7.1 Hz, 1H), 2.32 (s, 3H), 2.15 (s, 3H), 1.62 (d, *J* = 7.2 Hz, 3H); ^{13}C NMR (125 MHz, $CDCl_3$) δ 154.3, 146.4, 142.2, 135.9, 132.9, 130.8, 128.6, 127.6, 127.5, 123.8, 41.2, 21.8, 21.3, 19.4; HRMS (ESI) $C_{16}H_{18}NO_2$ $[M+H]^+$: *m/z* calcd. 256.1332; found 256.1335.

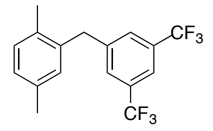

2-(3,5-Bis(trifluoromethyl)benzyl)-1,4-dimethylbenzene (16)

General reaction protocol A was followed with 3,5-bis(trifluoromethyl) benzyl alcohol (49 mg, 0.2 mmol), **2** (64 mg, 0.6 mmol), Re_2O_7 (1.0 mg, 0.002 mmol), and HFIP (0.4 mL). The reaction mixture was heated to 80 °C for 23 h then was quenched with Et_3N, concentrated under vacuum and purified by flash chromatography (100% hexane to 2% ethyl acetate in hexane) to give the desired product (48 mg, 73% yield). ^{1}H NMR (500 MHz, $CDCl_3$) δ 7.71 (s, 1H), 7.56 (s, 2H), 7.09 (d, *J* = 7.8 Hz, 1H), 7.02 (d, *J* = 7.8 Hz, 1H), 6.90 (s, 1H), 4.06 (s, 2H), 2.31 (s, 3H), 2.17 (s, 3H); ^{13}C NMR (125 MHz, $CDCl_3$) δ 143.2, 136.4, 136.0, 133.3, 131.7 (q, *J* = 33 Hz), 130.7, 130.7, 128.8 (q, *J* = 4.2 Hz), 128.0, 123.4 (q, *J* = 270.4 Hz), 120.2 (sep, *J* = 3.8 Hz), 39.1, 21.0, 19.2; ^{19}F NMR (470.4 MHz, $CDCl_3$; CF_3CO_2H – ext. std.) δ –63.7 (s); HRMS (ESI) $C_{17}H_{14}F_6$ $[M^+]$: *m/z* calcd. 332.1000; found 332.0990.

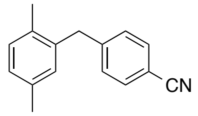

4-(2,5-Dimethylbenzyl)benzonitrile (17)

General reaction protocol A was followed with 4-cyanobenzyl alcohol (27 mg, 0.2 mmol), **2** (64 mg, 0.6 mmol), Re_2O_7 (1.0 mg, 0.002 mmol), and HFIP (0.4 mL). The reaction mixture was stirred at 80 °C for 48 h then was quenched with Et_3N, concentrated under vacuum, and purified by flash chromatography (100% hexane to 2% ethyl acetate in hexane) to give the desired product (25 mg, 57% yield). ^{1}H NMR (500 MHz, $CDCl_3$) δ 7.55 (dt, *J* = 8.4, 1.9 Hz, 2H), 7.21 (d, *J* = 8.4 Hz, 2H), 7.07 (d, *J* = 7.7 Hz, 1H), 7.00 (dd, *J* = 7.7, 1.2 Hz, 1H), 6.90 (s, 1H), 4.00 (s, 2H), 2.30 (s, 3H), 2.15 (s, 3H); ^{13}C NMR (125 MHz,

$CDCl_3$) δ 146.4, 137.0, 135.8, 133.4, 132.2, 130.8, 130.5, 129.4, 127.7, 119.1, 109.9, 39.6, 20.9, 19.1; HRMS (ESI) $C_{16}H_{16}N$ $[M+H]^+$: *m/z* calcd. 222.1277; found 222.1276.

General procedure B for substituted arene nucleophiles

Unless specified otherwise, **18** (0.2 mmol, 40mg) was added to a mixture of the arene (0.6 mmol, 3.0 equiv), Re_2O_7 (1.0 mg, 0.002 mmol, 1 mol%) and HFIP (0.4 mL). The mixture was stirred vigorously at the indicated temperature and monitored by TLC until full conversion of the benzyl alcohol. The reaction was then treated with triethylamine (10 μL). Purification was performed by column chromatography using EtOAc/hexanes as the eluents.

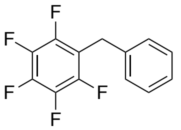

1-Benzyl-2,3,4,5,6-pentafluorobenzene (19)

Prepared according to general procedure B with benzene (5.0 equiv.), at 100 °C, for 3 d. Purification by flash chromatography gave the desired product (47.6 mg, 92%). ^1H NMR (501 MHz, CD_2Cl_2) δ 7.32-7.27 (m, 1H), 7.26-7.20 (m, 2H), 4.04 (t, *J* = 2.0 Hz, 1H); ^{13}C NMR (126 MHz, CD_2Cl_2) δ 137.6, 128.7, 128.2, 126.9, 28.0; ^{19}F NMR (471 MHz, CD_2Cl_2) δ –142.13 - –145.77 (m, 2F), –154.43 - –160.33 (m, 1F), –163.42 (td, *J* = 22.0, 8.3 Hz, 2F); HRMS (EI) for $C_{13}H_7F_5$ $[M]^+$: *m/z* calcd. 258.0462, found 258.0464.

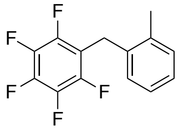

1,2,3,4,5-Pentafluoro-6-(x-methylbenzyl)benzene (20, x = 2, 3, or 4)

Prepared according to general procedure B with toluene (3.0 equiv) at 80 °C, for 24 h. Purification provided the desired product (53.9 mg, 99% yield) (*o*:*m*:*p* = 1.8:1:1.7). ^1H NMR (501 MHz, CD_2Cl_2) δ 7.31-6.71 (m, 4H), 4.02 (s, 0.8H), 3.99 (s, 1.2H), 2.37 (s, 1.2H), 2.30 (s, 0.6H), 2.29 (s, 1.2H); ^{13}C NMR (126 MHz, CD_2Cl_2) δ 138.5, 137.4, 136.7, 136.2, 135.3, 134.5, 130.3, 129.3, 128.9, 128.5, 128.1, 127.9, 127.6, 126.9, 126.1, 125.2, 27.9, 27.6, 25.5, 21.0, 20.6, 19.2; ^{19}F NMR (471 MHz, CD_2Cl_2) δ –142.9 - –143.1 (m, 0.8F), –143.7 - –143.9 (m, 0.4F), –144.0 - –144.1 (m, 0.8F), –158.0 - –158.1 (m, 0.4F), –158.3 - –158.7 (m, 0.6F), –163.3 - –163.7 (m, 2F); HRMS (EI) $C_{14}H_9F_5$ $[M]^+$: *m/z* calcd. 272.0618, found 272.0622.

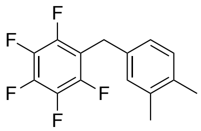

1-(x,y-Dimethylbenzyl)-2,3,4,5,6-pentafluorobenzene (21, x,y = 2, 3 or 3,4)

Prepared according to general procedure B with *o*-xylene (3.0 equiv) at 80 °C, for 5 h. Purification provided the desired product (56.4 mg, 98% yield, 2,3:3,4 = 2:3). ^1H NMR (501 MHz, CD_2Cl_2) δ 7.09 – 6.73 (m, 3H), 4.04 (s, 0.8H), 3.96 (s, 1.2H), 2.30 (s, 1.2H), 2.27 (s, 1.2H), 2.21 (s, 1.8H), 2.21 (s, 1.8H); ^{13}C NMR (126 MHz, CD_2Cl_2) δ 137.1, 137.0, 135.3, 135.1, 134.8, 134.7, 129.8, 129.4, 128.6, 125.7, 125.5, 125.4, 27.5, 26.1, 20.4, 19.4, 19.0, 14.8; ^{19}F NMR (471 MHz, CD_2Cl_2) δ –143.0 (d, *J* = 13.8 Hz, 0.8F), –143.6 - –144.7 (m, 1.2F), –158.2 (t, *J* = 20.6 Hz, 0.4F), –158.4 - –159.1 (m, 0.6F), –163.1 - –164.3 (m, 2F); HRMS (EI) $C_{15}H_{11}F_5$ $[M]^+$: *m/z* calcd. 286.0775, found 286.0780.

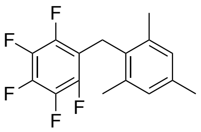

1,2,3,4,5-pentafluoro-6-(2,4,6-trimethylbenzyl)benzene (22)

Prepared according to general procedure B with 1,3,5-mesitylene (3.0 equiv), at 80 °C, for 4 h. Purification provided the desired product (59.8 mg, 99%). ^{1}H NMR (501 MHz, CD_2Cl_2) δ 6.85 (s, 2H), 4.03 (s, 2H), 2.24 (s, 9H); ^{13}C NMR (126 MHz, CD_2Cl_2) δ 136.9, 136.4, 130.7, 129.1, 22.97, 22.96, 22.94, 22.93, 20.5, 19.78, 19.77, 19.75; ^{19}F NMR (471 MHz, CD_2Cl_2) δ –142.7 - –143.0 (m, 2F), –158.9 (t, *J* = 20.7 Hz, 1F), –164.0 (td, *J* = 21.8, 7.9 Hz, 2F); HRMS (EI): *m*/*z* $C_{16}H_{13}F_5$ $[M]^+$: calcd. 300.0937, found 300.0936.

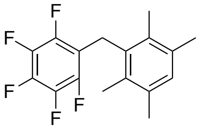

1,2,3,4,5-Pentafluoro-6-(2,3,5,6-tetramethylbenzyl)benzene (23)

Prepared according to general procedure B with 1,2,4,5-tetramethylbenzene (3.0 equiv) at 80 °C, for 4 h. Purification provided the desired product (61 mg, 97%). ^{1}H NMR (501 MHz, CD_2Cl_2) δ 6.91 (s, 1H), 4.12 (s, 2H), 2.22 (s, 6H), 2.16 (s, 6H); ^{13}C NMR (126 MHz, CD_2Cl_2) δ 133.7, 133.6, 132.9, 130.5, 24.0, 20.3, 15.5; ^{19}F NMR (471 MHz, CD_2Cl_2) δ –141.5 - –144.3 (m, 2F), –159.1 (t, *J* = 20.9 Hz, 1F), –164.1 (td, *J* = 21.5, 7.7 Hz, 2F); HRMS (EI): *m*/*z* $C_{17}H_{15}F_5$ $[M]^+$: calcd. 314.1094, found 314.1091.

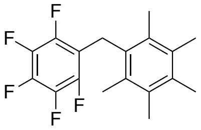

1,2,3,4,5-Pentafluoro-6-(2,3,4,5,6-pentamethylbenzyl)benzene (24)

Prepared according to general procedure B with 1,2,3,4,5-pentamethylbenzene (2.0 equiv) at 80 °C for 4 h. Purification provided the desired product (61.8 mg, 94%). ^{1}H NMR (501 MHz, CD_2Cl_2) δ 4.13 (s, 2H), 2.24 (s, 3H), 2.22 (s, 6H), 2.21 (s, 6H); ^{13}C NMR (126 MHz, CD_2Cl_2) δ 133.8, 132.62, 132.59, 131.1, 24.7, 16.8, 16.8, 16.7; ^{19}F NMR (471 MHz, CD_2Cl_2) δ –140.6 - –145.4 (m, 2F), –159.2 (t, *J* = 20.7 Hz, 1F), –164.2 (td, *J* = 21.8, 7.6 Hz, 2F); HRMS (EI): *m*/*z* $C_{18}H_{17}F_5$ $[M]^+$: calcd. 328.1250, found 328.1248.

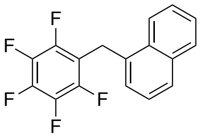

x-((Perfluorophenyl)methyl)naphthalene (25, x = 1 or 2)

Prepared according to general procedure B with naphthalene (3.0 equiv), at 80 °C for 4 h. Purification provided the desired product (60.1 mg, 97%, **1**:**2** = 3:1). ^{1}H NMR (501 MHz, CD_2Cl_2) δ 8.18 – 7.03 (m, 7H), 4.50 (s, 0.5H), 4.19 (d, *J* = 2.1 Hz, 1.5H); ^{13}C NMR (126 MHz, CD_2Cl_2) δ 135.0, 133.8, 133.5, 132.8, 132.3, 131.5, 128.8, 128.4, 128.1, 127.8, 127.7, 127.53, 127.47, 126.7, 126.5, 126.4, 126.3, 125.9, 125.8, 125.5, 125.4, 122.9, 28.20, 28.19, 28.18, 28.16, 25.2; ^{19}F NMR (471 MHz, CD_2Cl_2) δ –142.1 - –143.1 (m, 0.5F), –143.3 - –145.0 (m, 1.5F), –157.6 (t, *J* = 20.9 Hz, 0.25F), –158.0 (t, *J* = 20.7 Hz, 0.75F), –163.3 (tdd, *J* = 22.0, 13.7, 7.9 Hz, 2F); HRMS (EI): *m*/*z* $C_{17}H_9F_5$ $[M]^+$: calcd. 308.0619, found 308.0618.

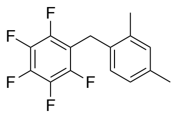

1-(x,y-Dimethylbenzyl)-2,3,4,5,6-pentafluorobenzene (x,y = 2, 4; 2,6 or 3,5) (26)

Prepared according to general procedure B with *m*-xylene (3.0 equiv) at 80 °C for 5 h. Purification provided the desired product (55.5 mg, 97%, 1,2,4:1,2,6:1,3,5 = 8.4:2.0:1.0). ^{1}H NMR (501 MHz, CD_2Cl_2) δ 7.14 – 6.76 (m, 3H), 4.07 (s, 0.35H), 3.97 (s, 1.47H), 3.95 (s, 0.18H), 2.49 – 2.13 (m, 6H); ^{13}C NMR (126 MHz, CD_2Cl_2) δ 138.35, 137.30, 137.1, 136.6, 135.9, 133.8, 132.2, 131.4, 131.1, 128.4, 128.3, 127.94, 127.92, 126.8, 126.7, 126.5, 125.9, 27.8, 25.10, 25.09, 23.3, 20.9, 20.6, 20.5, 19.88, 19.86, 19.85, 19.1; ^{19}F NMR (471 MHz, CD_2Cl_2) δ –142.4 - –143.0 (m, 0.35F), –142.9 - –143.6 (m, 1.47F), –158.3 (t, *J* = 20.7 Hz, 0.73F), –158.5 - –158.6 (m, 0.09F), –158.7 (t, *J* = 20.8 Hz, 0.18F), –163.6 (td, *J* = 28.7, 8.0 Hz, 1.65H), –163.9 (td, *J* = 22.1, 8.0 Hz, 0.35H); HRMS (EI): *m/z* $C_{15}H_{11}F_5$ $[M]^+$: calcd. 286.0775, found 286.0779.

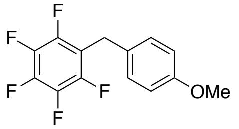

1,2,3,4,5-Pentafluoro-6-(4-methoxybenzyl)benzene (27)

Prepared according to general procedure B with anisole (3.0 equiv), at 80 °C for 24 h. Purification provided the desired product (56.5 mg, 98 % yield, *o*:*p* = 1.0:2.8). *para*-Isomer: ^{1}H NMR (501 MHz, CD_2Cl_2) δ 7.15 (ddd, *J* = 7.7, 1.5, 0.7 Hz, 1H), 6.92-6.53 (m, 1H), 3.96 (s, 1H), 3.75 (s, 2H); ^{13}C NMR (126 MHz, CD_2Cl_2) δ 158.6, 129.5, 129.3, 114.0, 55.1, 27.2; ^{19}F NMR (471 MHz, CD_2Cl_2) δ –144.20 - –144.3 (m, 2F), –158.4 - –158.7 (m, 1F), –163.5 (td, *J* = 21.8, 8.1 Hz, 2F); HRMS (EI): *m/z* $C_{14}H_9F_5O$ $[M]^+$: calcd. 288.0568, found 288.0571.

ortho-Isomer: ^{1}H NMR (501 MHz, CD_2Cl_2) δ 7.22 (td, *J* = 7.9, 1.7 Hz, 1H), 7.03 (d, *J* = 7.5 Hz, 1H), 6.86 (d, *J* = 7.6 Hz, 2H), 4.00 (s, 2H), 3.80 (s, 3H); ^{13}C NMR (126 MHz, CD_2Cl_2) δ 157.3, 129.3, 128.2, 125.5, 120.3, 110.3, 55.2, 23.0; ^{19}F NMR (471 MHz, CD_2Cl_2) δ –142.1 - –144.7 (m, 2F), –158.9 (t, *J* = 20.7 Hz, 2F), –164.3 (dd, *J* = 21.2, 13.7 Hz, 2F); HRMS (EI): *m/z* $C_{14}H_9F_5O$ $[M]^+$: calcd. 288.0568, found 288.0573.

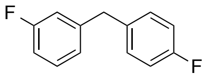

1-Fluoro-3-(4-fluorobenzyl)benzene (30)

General reaction protocol A was followed with 3-fluorobenzyl alcohol (23 mg, 0.2 mmol), fluorobenzene (58 mg, 0.6 mmol), Re_2O_7•SiO_2 (9.6 mg, 0.002 mmol), and HFIP (0.4 mL). The reaction mixture was heated to 80 °C for 2 h then was quenched with Et_3N, concentrated under vacuum and purified by flash chromatography (100% hexane to 2% ethyl acetate in hexane) to give the desired products as a mixture of regioisomers (16 mg, 40% yield, *p*/*o*=1.6/1). ^{1}H NMR (500 MHz, $CDCl_3$) δ 7.75-6.85 (m, 8H), 4.07 (s, 0.75 H), 3.88 (s, 1.25 H); ^{13}C NMR (125 MHz, $CDCl_3$) δ 163.2 (d, *J* = 245.8 Hz), 163.2 (d, *J* = 248.6 Hz), 161.7 (d, *J* = 244.5 Hz), 161.1 (d, *J* = 245.5 Hz), 143.6 (d, *J* = 7.3 Hz), 142.6 (d, *J* = 7.2 Hz), 136.1 (d, *J* = 3.8 Hz), 131.2 (d, *J* = 4.5 Hz), 130.5 (d, *J* = 8.4 Hz), 130.1 (d, *J* = 8.2 Hz), 130.0 (d, *J* = 8.2 Hz), 128.4 (d, *J* = 8.2 Hz), 127.4 (d, *J* = 15.9 Hz), 124.6 (d, *J* = 2.7 Hz), 124.5 (d, *J* = 2.7 Hz), 124.3 (d, *J* = 3.6 Hz), 115.9 (d, *J* = 21.3 Hz), 115.8 (d, *J* = 20.8 Hz), 115.6 (d, *J* = 21.5 Hz), 115.5 (d, *J* = 20.9 Hz), 113.3 (d, *J* = 20.9 Hz), 113.3 (d, *J* = 20.9 Hz), 40.9 (d, *J* = 2.1 Hz), 34.8 (d, *J* = 1.8 Hz); ^{19}F NMR (470.4 MHz, $CDCl_3$; CF_3CO_2H – ext. std.) δ –114.3 (m, 0.62F), –

114.4 (m, 0.38F), –117.8 (tt, *J* = 13.0, 5.2 Hz, 0.62F); –118.7 (dt, *J* = 12.1, 5.2 Hz, 0.38F); HRMS $C_{13}H_{10}F_2$ [M^+]: *m/z* calcd. 204.0751; found 204.0715. The regiochemistry was assigned by analyzing the ^{19}F NMR spectrum.

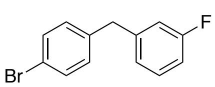

1-(4-Bromobenzyl)-3-fluorobenzene (32)

General reaction protocol A was followed with 3-fluorobenzyl alcohol (23 mg, 0.2 mmol), bromobenzene (93 mg, 0.6 mmol), Re_2O_7•SiO_2 (9.6 mg, 0.002 mmol), and HFIP (0.4 mL). The reaction mixture was heated to 80 °C for 2 h then was quenched with Et_3N, concentrated under vacuum and purified by flash chromatography (100% hexane to 2% ethyl acetate in hexane) to give the desired products as a mixture of regioisomers (25 mg, 46% yield, *p*/*o*=1/1). ^{1}H NMR (500 MHz, $CDCl_3$) δ 7.57 (dd, *J* = 8.2 Hz, 1 H), 7.41 (d, *J* = 8.2 Hz, 1H), 7.27–7.21 (m, 1.5H), 7.15 (dd, *J* = 7.5, 1.4 Hz, 0.5H), 7.10 (td, *J* = 7.8, 1.4 Hz, 0.5H), 7.05 (d, *J* = 8.1 Hz, 1 H), 6.94–6.81 (m, 3H), 4.10 (s, 1H), 3.91 (s, 1H); ^{13}C NMR (125 MHz, $CDCl_3$) δ 163.2 (d, *J* = 246 Hz), 163.1 (d, *J* = 246 Hz), 143.1 (d, *J* = 7.3 Hz), 142.2 (d, *J* = 7.3 Hz), 139.7, 139.4, 133.2, 131.8, 131.3, 130.8, 130.1 (d, *J* = 8.2 Hz), 130.0 (d, *J* = 8.2 Hz), 128.3, 127.7, 125.0, 124.7 (d, *J* = 2.7 Hz), 124.6 (d, *J* = 2.7 Hz), 120.4, 116.0 (d, *J* = 22.1 Hz), 115.9 (d, *J* = 21.3 Hz), 113.4 (d, *J* = 21.0 Hz), 113.3 (d, *J* = 21.9 Hz), 41.6 (d, *J* = 1.8 Hz), 41.1 (d, *J* = 2.1 Hz); ^{19}F NMR (470.4 MHz, $CDCl_3$; CF_3CO_2H – ext. std.) δ –114.1 (td, *J* = 9.5, 6.9 Hz, 1F), –114.3 (td, *J* = 9.5, 5.2 Hz, 1F); HRMS (ESI) $C_{13}H_{10}BrF$ [M^+]: *m/z* calcd. 263.9950; found 263.9967. The regiochemistry was assigned based on 1D TOCSY experiments.

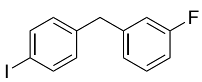

1-(3-Fluorobenzyl)-4-iodobenzene (34)

General reaction protocol A was followed with 3-fluorobenzyl alcohol (23 mg, 0.2 mmol), iodobenzene (122 mg, 0.6 mmol), Re_2O_7•SiO_2 (9.6 mg, 0.002 mmol), and HFIP (0.4 mL). The reaction mixture was heated to 80 °C for 2 h then was quenched with Et_3N, concentrated under vacuum and purified by flash chromatography (100% hexane to 2% ethyl acetate in hexane) to give the desired products as a mixture of regioisomers (38 mg, 62% yield, *p*/*o* = 1.2/1). A more polar product was recovered (16.8 mg) during the reaction. GC-MS analysis showed the second product is consisted of multiple dialkylated product. ^{1}H NMR (400 MHz, $CDCl_3$) δ 7.78 (dd, *J* = 8.0, 1.0 Hz, 0.45H), 7.64–7.60 (m, 1.41H), 7.53 (d, *J* = 8.3 Hz, 1.0H), 7.24 (td, *J* = 11.2, 1.0 Hz, 0.84H), 7.19–7.12 (m, 1.60H), 7.06–6.99 (m, 1.98H), 6.90–6.72 (m, 4.87H), 4.10 (s, 0.90H), 3.90 (s, 1.10H); ^{13}C NMR (100 MHz, $CDCl_3$) δ 163.0 (d, *J* = 245.7 Hz), 163.0 (d, *J* = 245.7 Hz), 143.0 (d, *J* = 7.3 Hz), 142.8, 142.1 (d, *J* = 7.3 Hz), 140.0, 139.7, 137.7, 131.0, 130.4, 130.0 (d, *J* = 8.5 Hz), 130.0 (d, *J* = 8.5 Hz), 128.5, 128.3, 124.7 (d, *J* = 2.9 Hz), 124.5 (d, *J* = 2.9 Hz), 115.9 (d, *J* = 21.3 Hz), 115.8 (d, *J* = 21.3 Hz), 113.3 (d, *J* = 21.3 Hz), 113.2 (d, *J* = 21.3 Hz), 101.2, 91.8, 46.3, 41.1 (d, *J* = 1.5 Hz); ^{19}F NMR (470.4 MHz, $CDCl_3$; CF_3CO_2H – ext. std.) δ –114.2 (m), –114.3 (td, *J* = 9.5, 5.2 Hz); HRMS (ESI) $C_{13}H_{10}FI$ [M^+]: *m/z* calcd. 311.9811; found 311.9796. The regiochemistry was assigned based on 1D TOCSY experiments.

1-Bromo-2-(fluorobenzyl)benzene (36)

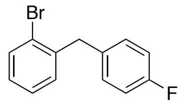

General reaction protocol A was followed with 2-bromobenzyl alcohol (37 mg, 0.2 mmol), fluorobenzene (58 mg, 0.6 mmol), Re_2O_7•SiO_2 (9.6 mg, 0.002 mmol), and HFIP (0.4 mL). The reaction mixture was heated to 80 °C for 2 h then was quenched with Et_3N, concentrated under vacuum and purified by flash chromatography (100% hexane to 2% ethyl acetate in hexane) to give the desired products as a mixture of regioisomers (29 mg, 55% yield, *p*/*o*=2.8/1). ^{1}H NMR (500 MHz, $CDCl_3$) δ 7.56–6.94 (m, 8H), 4.13 (s, 0.53H), 4.07 (s, 1.47H); ^{13}C NMR (125 MHz, $CDCl_3$) δ 161.7 (d, *J* = 244.3 Hz), 161.2 (d, *J* = 245.9 Hz), 140.4, 139.2, 135.3 (d, *J* = 2.7 Hz), 133.1, 133.0, 131.1, 131.1(d, *J* = 2.7 Hz), 131.0, 130.5 (d, *J* = 8.2 Hz), 128.3 (d, *J* = 8.4 Hz), 128.2, 127.7, 127.7, 126.6, 126.5, 125.0, 125.0,124.2 (d, *J* = 2.7 Hz), 115.4 (d, *J* = 20.7 Hz), 115.4 (d, *J* = 20.7 Hz), 41.1, 34.9 (d, *J* = 2.7 Hz); ^{19}F NMR (470.4 MHz, $CDCl_3$; CF_3CO_2H – ext. std.) δ –117.9 (ddd, *J* = 13.9, 8.7, 5.2 Hz, 1.47 F), –118.2 (dt, *J* = 12.1, 5.2 Hz, 0.53 F); HRMS (ESI) $C_{13}H_{10}BrF$ [M^+]: *m*/*z* calcd. 263.9950; found 263.9971. The regiochemistry was assigned by analyzing the ^{19}F NMR spectrum.

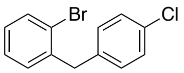

1-Bromo-2-(4-chlorobenzyl)benzene (38)

General reaction protocol A was followed with 2-bromobenzyl alcohol (37 mg, 0.2 mmol), chlorobenzene (66 mg, 0.6 mmol), Re_2O_7•SiO_2 (9.6 mg, 0.002 mmol), and HFIP (0.4 mL). The reaction mixture was heated to 80 °C for 2 h then was quenched with Et_3N, concentrated under vacuum and purified by flash chromatography (100% hexane to 2% ethyl acetate in hexane) to give the desired products as a mixture of regioisomers (31 mg, 56% yield, *p*/*o* = 2.2/1). ^{1}H NMR (500 MHz, $CDCl_3$) δ 7.56 (dd, *J* = 8.0, 0.9 Hz, 0.33 H), 7.42–7.37 (m, 1.30H), 7.27–7.22 (m, 2.30 H), 7.13–7.08 (m, 1.30 H), 7.07 (m, 1.30 H), 7.02 (m, 1.30 H), 4.07 (s, 0.70 H), 3.88 (s, 1.30 H); ^{13}C NMR (125 MHz, $CDCl_3$, signals corresponding to both regioisomers are reported) δ 140.0, 139.0, 138.1, 137.3, 134.6, 133.1, 133.0, 132.3, 131.2, 130.9, 130.8, 130.4, 129.7, 128.8, 128.3, 128.2, 128.0, 127.7, 127.7, 127.0, 125.2, 125.0, 41.3, 39.5; HRMS (ESI) $C_{13}H_9BrCl$ $[M\text{-}H]^+$: *m*/*z* calcd. 278.9571; found 278.9578. The regiochemistry was assigned based on analogy of the chemical shifts for the benzylic hydrogens compared to other compounds.

1-Bromo-4-(4-fluorobenzyl)benzene (39)

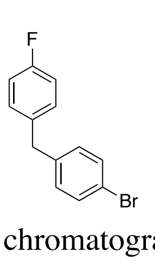

General reaction protocol A was followed with 4-fluorobenzyl alcohol (23 mg, 0.2 mmol), bromobenzene (93 mg, 0.6 mmol), Re_2O_7•SiO_2 (9.6 mg, 0.002 mmol), and HFIP (0.4 mL). The reaction mixture was heated to 80 °C for 2 h then was quenched with Et_3N, concentrated under vacuum and purified by flash chromatography (100% hexane to 2% ethyl acetate in hexane) to give the desired products as a mixture of regioisomers (30 mg, 56% yield, *p*/*o*=2/1). ^{1}H NMR (400 MHz, $CDCl_3$) δ 7.56–6.94 (m, 8 H), 4.08 (s, 0.67 H), 3.89 (s, 1.33 H); ^{13}C NMR (125 MHz, $CDCl_3$) δ 161.7 (d, *J* = 244.7 Hz), 161.7 (d, *J* = 244.0 Hz), 140.4, 140.1, 136.3 (d, *J* = 2.9 Hz), 135.3 (d, *J* = 2.9 Hz), 133.1, 131.8, 131.7, 131.1, 130.7, 130.5 (d, *J* = 7.9 Hz), 130.4 (d, *J* = 7.6 Hz), 130.2, 128.2, 127.7,

125.0, 120.2, 115.5 (d, J = 21.2 Hz), 115.4 (d, J = 21.2 Hz), 41.1, 40.6; ^{19}F NMR (470.4 MHz, $CDCl_3$; CF_3CO_2H – ext. std.) δ –117.8 (tt, J = 8.7, 5.2 Hz, 2F), –117.9 (tt, J = 8.7, 5.2 Hz, 1F); HRMS (ESI) $C_{13}H_{10}BrF$ [M^+]: *m/z* calcd. 263.9950; found 263.9979. The regiochemistry was assigned based on 1D TOCSY experiments.

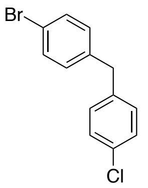

1-Bromo-4-(4-chlorobenzyl)benzene (41)

General reaction protocol A was followed with 4-chlorobenzyl alcohol (28 mg, 0.2 mmol), bromobenzene (94 mg, 0.6 mmol), Re_2O_7•SiO_2 (9.6 mg, 0.002 mmol), and HFIP (0.4 mL). The reaction mixture was heated to 80 °C for 2 h then was quenched with Et_3N, concentrated under vacuum and purified by flash chromatography (100% hexane to 2% ethyl acetate in hexane) to give the desired products as a mixture of regioisomers (30 mg, 53% yield, *p*/*o*=2.2/1). ^{1}H NMR (400 MHz, $CDCl_3$) δ 7.56 (dd, J = 8.0, 0.9 Hz, 0.33 H), 7.40 (d, J = 8.4 Hz, 1.30 H), 7.27–7.22 (m, 2.30 H), 7.13–7.08 (m, 1.30 H), 7.07 (d, J = 8.3 Hz, 1.30 H), 7.02 (d, J = 8.3 Hz, 1.30 H), 3.99 (s, 0.70 H), 3.80 (s, 1.30 H); ^{13}C NMR (125 MHz, $CDCl_3$) δ 140.0, 139.0, 138.1, 137.3, 134.6, 133.1, 133.0, 132.3, 131.2, 130.9, 130.8, 130.4, 129.7, 128.8, 128.3, 128.2, 128.0, 127.7, 127.7, 127.0, 125.2, 125.0, 41.3, 39.5; HRMS (ESI) $C_{13}H_9BrCl$ [M–H]$^+$: *m/z* calcd. 278.9571; found 278.9579. The regiochemistry was assigned based on 1D TOCSY experiments.

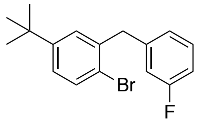

1-Bromo-4-(*tert*-butyl)-2-(3-fluorobenzyl)benzene (43)

General reaction protocol A was followed with 3-fluorobenzyl alcohol (23 mg, 0.2 mmol), 4-*tert*-butylbromobenzene (127 mg, 0.6 mmol), Re_2O_7•SiO_2 (9.6 mg, 0.002 mmol), and HFIP (0.4 mL). The reaction mixture was heated to 80 °C for 2 h then was quenched with Et_3N, concentrated under vacuum and purified by flash chromatography (100% hexane to 2% ethyl acetate in hexane) to give the desired product (28 mg, 43% yield). ^{1}H NMR (500 MHz, $CDCl_3$) δ 7.47 (d, J = 8.4 Hz, 1 H), 7.24 (td, J = 8.0, 6.1 Hz, 1H), 7.19 (d, J = 2.5 Hz, 1H), 7.13 (dd, J = 8.4, 2.5 Hz, 1 H), 6.97 (d, J = 7.9 Hz, 1 H), 6.92–6.84 (m, 2H), 4.10 (s, 2 H), 1.27 (s, 9 H); ^{13}C NMR (125 MHz, $CDCl_3$) δ 163.2 (d, J = 246.5 Hz), 151.0, 142.5 (d, J = 7.2 Hz), 138.9, 132.7, 129.9 (d, J = 8.2 Hz), 128.6, 125.6, 124.6 (d, J = 2.7 Hz), 121.8, 115.8 (d, J = 21.8 Hz), 113.2 (d, J = 21.0 Hz), 41.7 (d, J = 1.8 Hz), 34.7, 31.4; ^{19}F NMR (470.4 MHz, $CDCl_3$; CF_3CO_2H – ext. std.) δ –114.5 (m); HRMS (ESI) $C_{17}H_{18}BrF$ [M^+]: *m/z* calcd. 320.0576; found 320.0592. The regiochemistry was assigned based on 1D NOE studies.

Re_2O_7•SiO_2 release study

Re_2O_7•SiO_2 (9.6 mg, 0.002 mmol) was stirred in HFIP (0.4 ml) at 80 °C for 0.5 h. The mixture was filtered through PTFE (0.2 μm membrane) with an extra 0.1 ml of HFIP. 4-Fluorobenzyl alcohol (21.8 μl, 0.2 mmol) and *p*-xylene (74.0 μl, 0.6 mmol) were added to the filtrate. The reaction mixture was stirred at 80 °C for 2 h then the reaction was quenched with Et_3N. The crude mixture was condensed under vacuum and purified by flash chromatography (hexane to 3% ethyl acetate in hexane) to give the product (35.3 mg, 82% yield).

Reactions with low catalyst loading

The following reaction were carried out in 20 mL scintillation vial (washed with KOH/EtOH and HCl (1 M) before use). Mesitylene was dried with $CaCl_2$ and distilled from sodium. *p*-methoxybenzyl alcohol was purified by shaking with aqueous KOH and extracting with diethyl ether, then the extract was treated with saturated NaHS, filtered, washed and dried over CaO and then distilled under reduced pressure to obtain pure *p*-methoxybenzyl alcohol. HFIP was redistilled from 3Å molecular sieves. Failure to purify the reagents properly leads to catalyst deactivation at very low loadings.

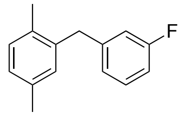

The general reaction protocol was followed with 3-fluorobenzyl alcohol (250 mg, 2.0 mmol), *p*-xylene (640 mg, 6.0 mmol), Re_2O_7•SiO_2 (9.6 mg, 0.002 mmol), and HFIP (4 mL), but the mixture was stirred at 80 °C for 3.5 h. The reaction was quenched with Et_3N, concentrated under vacuum, and purified by flash chromatography (100% hexane to 3% ethyl acetate in hexane) to give the desired product (315 mg, 73% yield).

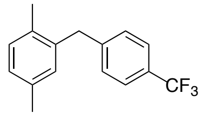

The general reaction protocol was followed with 4-trifluorobenzyl alcohol (350 mg, 2.0 mmol), **2** (640 mg, 6.0 mmol), Re_2O_7•SiO_2 (9.6 mg, 0.002 mmol), and HFIP (0.4 mL), except the mixture was heated to 80 °C for 24 h. The reaction was quenched with Et_3N, concentrated under vacuum, and purified by flash chromatography (100% hexane to 2% ethyl acetate in hexane) to give the desired product (45 mg, 9% yield).

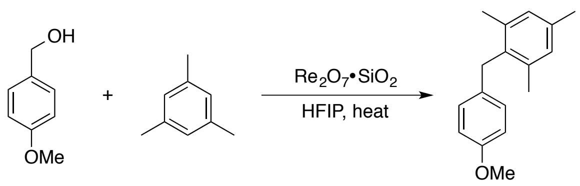

1 mol% catalyst, 0.5 M

The general reaction protocol was followed with 4-methoxybenzyl alcohol (28 mg, 0.2 mmol), mesitylene (72 mg, 0.6 mmol), Re_2O_7•SiO_2 (9.6 mg, 0.002 mmol), and HFIP (0.4 mL), except that the mixture was stirred at rt for 10 min. The reaction was quenched with Et_3N, concentrated under vacuum, and purified by flash chromatography (100% hexane to 8% ethyl acetate in hexane) to give the monoalkylated product (43.0 mg, 90% yield) and (2.1 mg, 6% yield) of the dialkylated product. All spectral data for the monoalkylated product matched those in the literature.[1] Characterization data for the dialkylated product: ^{1}H NMR (500 MHz, $CDCl_3$) δ 6.96–6.89 (m, 5H), 6.80–6.75 (m, 4H), 4.00 (s, 4H), 3.76 (s, 6H), 2.23 (s, 6H), 2.08 (s, 3H); ^{13}C NMR (125 MHz, $CDCl_3$) δ 157.9, 136.4, 135.3, 135.1, 132.4, 130.1, 128.9, 114.0, 55.4, 34.7, 20.4, 16.3; HRMS (ESI) $C_{25}H_{28}O_2$ [M^+]: *m/z* calcd. 360.2089; found 360.2101.

0.01 mol% catalyst, 0.5 M

To a flame-dried 20 mL scintillation vial filled with argon were added *p*-methoxybenzyl alcohol (380 mg, 2.8 mmol), mesitylene (1.0 g, 8.3 mmol) and HFIP (5.5 mL). Re_2O_7 (1.3 mg, 0.0002

[1] M. Hofmann, N. Hampel, T. Kanzian and H. Mayr, *Angew. Chem. Int. Ed.* 2004, **43**, 5402.

mmol) was added and the reaction mixture was stirred at rt for 7 h. The reaction was quenched with Et_3N, concentrated under vacuum, and purified by flash chromatography (100% hexane to 6% ethyl acetate in hexane) to give the product (614 mg, 93% yield) and dialkylated product (27 mg).

0.003 mol% catalyst, 0.5 M

To a flame-dried 20 mL scintillation vial filled with argon were added *p*-methoxybenzyl alcohol (843 mg, 6.1 mmol), mesitylene (2.20 g, 18.3 mmol), and HFIP (12 mL). Re_2O_7 (1.0 mg, 0.0002 mmol) was added and the reaction mixture was stirred in a 45 °C oil bath for 9 h. The reaction was quenched with Et_3N, concentrated under vacuum and purified by flash chromatography (100% hexane to 6% ethyl acetate in hexane) to give the product (1.41 g, 96% yield).

0.003 mol% catalyst, 2.5 M

To a flame-dried 20 mL scintillation vial filled with argon were added *p*-methoxybenzyl alcohol (828 mg, 6.0 mmol), mesitylene (2.20 g, 18.3 mmol), and HFIP (2.4 mL). Re_2O_7 (1.0 mg, 0.0002 mmol) was added and the reaction mixture was stirred in a 50 °C oil bath for 9 h. The reaction was quenched with Et_3N, concentrated under vacuum, and purified by flash chromatography (100% hexane to 6% ethyl acetate in hexane) to give the mono-alkylated product (1.40 g, 96% yield), along with 4% dialkylated product.

0.003 mol% catalyst, 5.0 M

To a flame-dried 20 mL scintillation vial filled with argon were added *p*-methoxybenzyl alcohol (821 mg, 5.9 mmol), mesitylene (2.20 g, 18.3 mmol), and HFIP (1.2 mL). Re_2O_7 (1.0 mg, 0.0002 mmol) was added and the reaction mixture was stirred in a 50 °C oil bath for 9 h. The reaction was quenched with Et_3N, concentrated under vacuum, and purified by flash chromatography (100% hexane to 6% ethyl acetate in hexane) to give the monoalkylated product (1.24 g, 87% yield), along with 8% dialkylated product.

Solvent studies

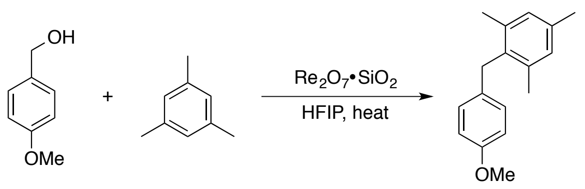

3:1 DCE:HFIP

The general reaction protocol was followed with 4-methoxybenzyl alcohol (28 mg, 0.2 mmol), mesitylene (72 mg, 0.6 mmol), Re_2O_7•SiO_2 (9.6 mg, 0.002 mmol), DCE (0.3 mL) and HFIP (0.1 mL), except that the mixture was stirred at rt for 10 min. The reaction was quenched with Et_3N, concentrated under vacuum, and purified by flash chromatography (100% hexane to 8% ethyl acetate in hexane) to give the monoalkylated product (41 mg, 84% yield) and the dialkylated product (3.3 mg, 9% yield).

9:1 DCE:HFIP

The general reaction protocol was followed with 4-methoxybenzyl alcohol (28 mg, 0.2 mmol), mesitylene (72 mg, 0.6 mmol), Re_2O_7•SiO_2 (9.6 mg, 0.002 mmol), DCE (0.36 mL) and HFIP (0.04 mL), except that the mixture was stirred at rt for 1 h. The reaction was quenched with Et_3N, concentrated under vacuum, and purified by flash chromatography (100% hexane to 8% ethyl acetate in hexane) to give the monoalkylated product (40 mg, 83% yield) and the dialkylated product (5.0 mg, 14% yield).

100% DCE

The general reaction protocol was followed with 4-methoxybenzyl alcohol (28 mg, 0.2 mmol), mesitylene (72 mg, 0.6 mmol), Re_2O_7•SiO_2 (9.6 mg, 0.002 mmol), and DCE (0.4 mL), except that the mixture was stirred at rt for 4 h. The reaction was quenched with Et_3N, concentrated under vacuum, and purified by flash chromatography (100% hexane to 8% ethyl acetate in hexane) to give the monoalkylated product (30 mg, 68% yield) and the benzyl ether (7.4 mg, 29% yield).

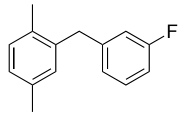

The general reaction protocol was followed with 3-fluorobenzyl alcohol (25 mg, 0.2 mmol), **2** (64 mg, 0.6 mmol), Re_2O_7•SiO_2 (9.6 mg, 0.002 mmol), and HFIP (0.3 mL) and DCE (0.1 mL). The reaction mixture was heated to 80 °C for 17 h then was quenched with Et_3N, concentrated under vacuum, and purified by flash chromatography (100% hexane to 2% ethyl acetate in hexane) to give the desired product (27 mg, 64% yield).

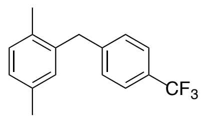

The general reaction protocol was followed with 4-trifluorobenzyl alcohol (35 mg, 0.2 mmol), **2** (64 mg, 0.6 mmol), Re_2O_7•SiO_2 (9.6 mg, 0.002 mmol), HFIP (0.3 mL) and DCE (0.1 mL). The reaction mixture was heated to 80 °C for 17 h then was quenched with Et_3N, concentrated under vacuum and purified by flash chromatography (100% hexane to 2% ethyl acetate in hexane) to give the products (7 mg, 14% yield).

Leaving group comparison

Alcohol

The general reaction protocol was followed with **1** (25 mg, 0.2 mmol), **2** (64 mg, 0.6 mmol), Re_2O_7•SiO_2 (9.6 mg, 0.002 mmol), and HFIP (0.4 mL). The reaction mixture stirred at rt for 6 h then was quenched with Et_3N, concentrated under vacuum and purified through flash chromatography (100% hexane to 2% ethyl acetate in hexane) to give the desired product (37 mg, 85% yield).

Bromide

4-Fluorobenzyl bromide (34 mg, 0.18 mmol), **2** (64 mg, 0.6 mmol), and HFIP (0.36 ml) were stirred at rt for 6 h, then concentrated under vacuum to get crude spectra (bromide/product = 1/11). Then the mixture was purified through flash chromatography (hexane to 2% ethyl acetate in hexane) to give the diarylmethane (27 mg, 69% yield).

Chloride

4-Fluorobenzyl chloride (43 mg, 0.30 mmol), *p*-xylene (96 mg, 0.9 mmol) and HFIP (0.6 ml). were stirred at rt for 6 h, then concentrated under vacuum. ^{1}H NMR analysis of the crude mixture with 4-dimethylamino pyridine (3.8 mg, 0.03 mmol) as internal standard showed a benzyl chloride/product ratio of 1/1.06, with an NMR yield of the product of 27.2 mg (43%) and 26 mg (40%) of the starting material.

Catalyst comparison

Re_2O_7

The general reaction protocol was followed with 2,3,4,5,6-pentafluorobenzyl alcohol (41 mg, 0.21 mmol), **2** (64 mg, 0.6 mmol), Re_2O_7 (1.0 mg, 0.002 mmol), and HFIP (0.4 mL). The reaction mixture was stirred for 2 h at 80 °C then was quenched with Et_3N, concentrated under vacuum, and purified by flash chromatography (100% hexane to 2% ethyl acetate in hexane) to give the desired product (54 mg, 90% yield).

Re_2O_7•SiO_2

The general reaction protocol was followed with 2,3,4,5,6-pentafluorobenzyl alcohol (36 mg, 0.18 mmol), **2** (64 mg, 0.6 mmol), Re_2O_7•SiO_2 (8.7 mg, 0.002 mmol), and HFIP (0.36 mL). The reaction mixture was stirred for 2 h at 80 °C then was quenched with Et_3N, concentrated under vacuum, and purified by flash chromatography (100% hexane to 2% ethyl acetate in hexane) to give the desired product (21 mg, 41% yield).

$HOReO_3$

The general reaction protocol was followed with 2,3,4,5,6-pentafluorobenzyl alcohol (49 mg, 0.25 mmol), **2** (79 mg, 0.75 mmol), $HOReO_3$ (76.5 wt% in H_2O, 0.38 µL, 0.003 mmol), and HFIP (0.4 mL). The reaction mixture was stirred for 2 h at 80 °C then was quenched with Et_3N, concentrated under vacuum, and purified by flash chromatography (100% hexane to 2% ethyl acetate in hexane) to give the desired product (55 mg, 77% yield).

$HOReO_3$ with an unreactive substrate

The general reaction protocol was followed with 3,5-bis-trifluoromethylbenzyl alcohol (49 mg, 0.2 mmol), **2** (64 mg, 0.6 mmol), $HOReO_3$ (76.5 wt% in H_2O, 0.61 µL, 0.004 mmol), and HFIP (0.4 mL). The reaction mixture was stirred for 21 h at 80 °C then was quenched with Et_3N, concentrated under vacuum, and purified by flash chromatography (100% hexane to 2% ethyl acetate in hexane) to give the desired product (34 mg, 51% yield).

Low catalyst loading $HOReO_3$

To a flame-dried 20 ml scintillation vial filled with argon were added *p*-methoxybenzyl alcohol (1.85 g, 13.4 mmol), mesitylene (4.8 g, 40 mmol) and HFIP (5.4 mL). $HOReO_3$ (0.20 µl, 0.0013 mmol, 76.5% in H_2O) was added and the mixture was stirred at rt for 9 h. The reaction was quenched with Et_3N, concentrated under vacuum, and purified by flash chromatography (100% hexane to 6% ethyl acetate in hexane) to give the monoalkylated product (2.96 g, 92%), and the dialkylated product (208 mg).

Kinetics studies

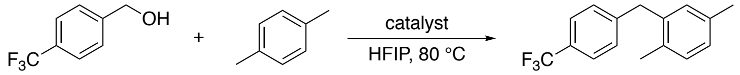

General procedure for kinetics experiments with a benzylic alcohol

A solution of 4-dimethylaminopyridine (0.5 M) and 1,4-difluorobenzene (0.5 M) in $CDCl_3$ was made prior to the reaction (solution A). To a 1 dram vial were added *p*-trifluoromethylbenzyl alcohol (1.0 equiv), *p*-xylene (3.0 equiv), the catalyst and the appropriate amount of HFIP to keep the concentration at 0.5 M. The vial was secured with a polypropylene screw cap containing a PTFE faced silicone septum (Chemglass CG-4904-05). The reaction mixture was stirred at 80 °C for 2 h. Every 10 min, 10 µl of reaction crude was drawn, then mixed with 500 µl $CDCl_3$ and 10 µl of solution A. ^{1}H NMR and ^{19}F NMR spectra were taken at ambient temperature. The yield was calculated according to the following equation:

$$Y = \frac{P}{S + P} \times 100\%$$

^{19}F NMR was used to calculate the ratio between starting material (S) and product (P). S represents the integration for 4-trifluoromethyl benzyl alcohol, P represents the integration for 1,4-dimethyl-2-(4-(trifluoromethyl)benzyl)benzene.

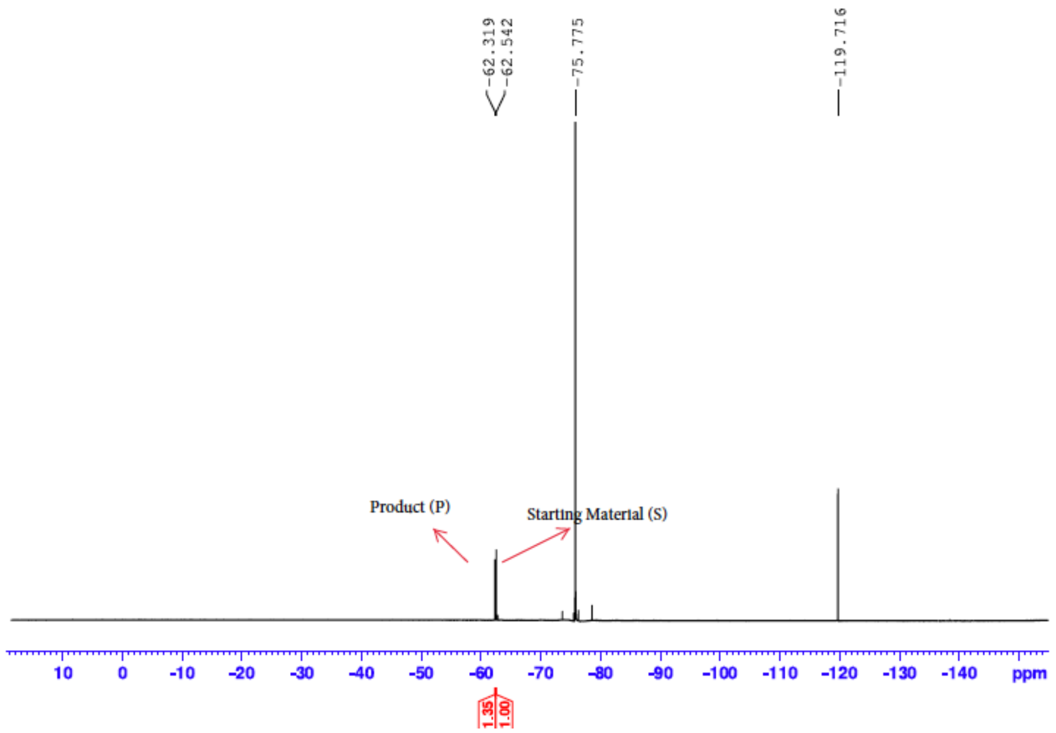

Experimental data:

10% TfOH

t [min]	Y (%) RXN 1	Y (%) RXN 2
10	57.4	57.4
20	76.7	75.8
30	84.8	77.4
40	86.7	80.9
50	90.9	90.4
60	91.8	94.7

2% TfOH

t [min]	Y (%) RXN 1	Y (%) RXN 2
10	13.8	13.1
20	24.3	25.9
30	36.9	37.6
40	46.8	46.3
50	53.4	53.4
60	58.4	58.7

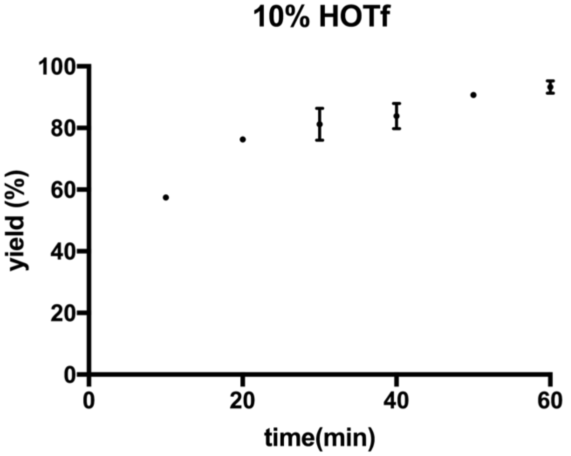

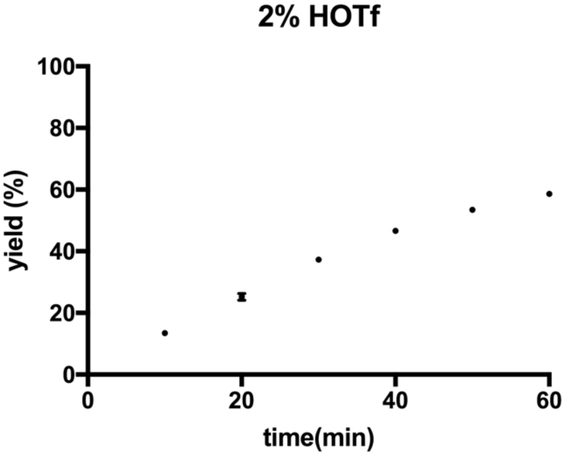

1% Re_2O_7

t [min]	Y (%) RXN 1	Y (%) RXN 2
10	62.3	54.2
20	82.4	86.0
30	91.8	95.1
40	94.6	96.4
50	97.0	96.1
60	97.6	96.2

1% Re_2O_7•SiO_2

t [min]	Y (%) RXN 1	Y (%) RXN 2
10	46.7	53.3
20	71.9	72.3
30	81.1	80.1
40	88.1	87.0
50	90.0	90.0
60	92.8	92.8

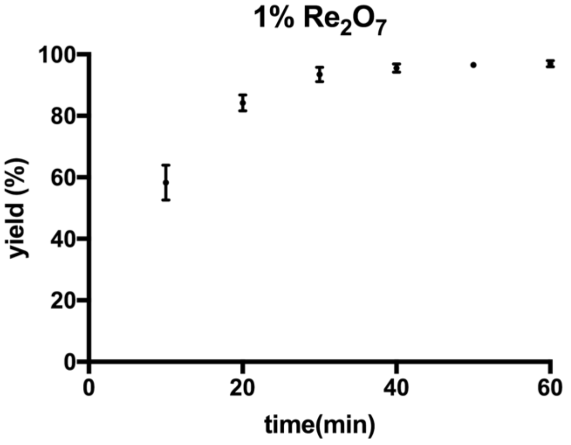

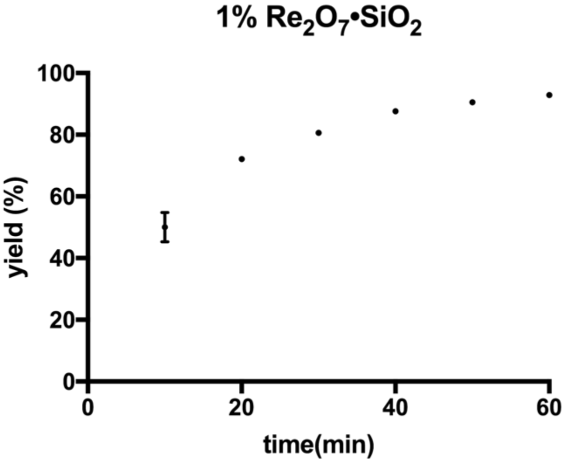

2% $HReO_4$

t [min]	Y (%) RXN 1	Y (%) RXN
10	73.9	70.5
20	97.5	93.6
30	99.5	98.7
40	99.5	99.5
50	99.7	99.7
60	99.8	99.8

1% $HReO_4$

t [min]	Y (%) RXN 1	Y (%) RXN 2
10	35.6	45.9
20	71.6	82.8
30	86.1	94.7
40	93.3	98.4
50	96.0	99.5
60	97.7	99.7

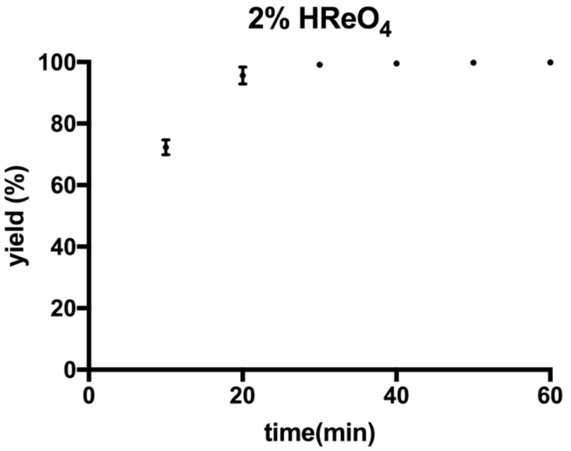

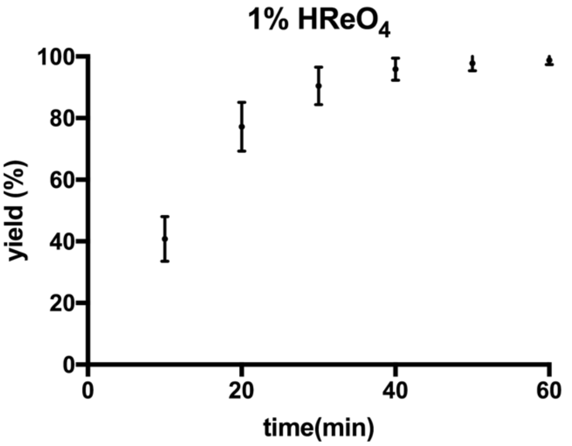

General procedure for kinetics experiments with a benzylic acetate

These experiments were conducted in exactly the same manner as for the benzylic alcohol except that *p*-trifluoromethylbenzyl acetate was used as the substrate.

10% HOTf

t [min]	Y (%) RXN1	Y (%) RXN2
10	22.8	20.4
20	39.2	33.4
30	44.5	43.4
40	48.0	48.3
50	53.7	51.1
60	60.2	55.8
70	63.9	58.2
80	64.7	61.7
90	66.6	65.9
100	69.0	68.7
110	71.1	72.3
120	71.4	73.5

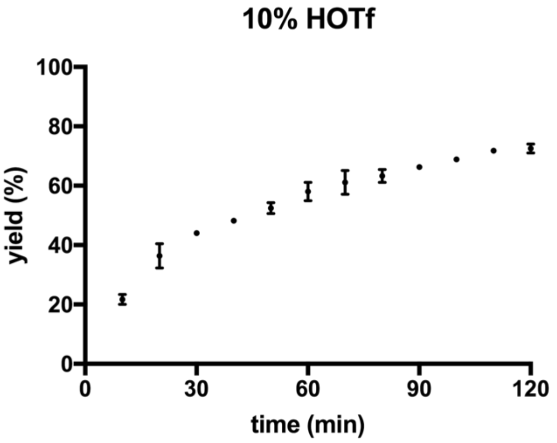

1% Re_2O_7

t [min]	Y (%) RXN1	Y (%) RXN2
10	0.1	0.1
20	0.4	0.5
30	0.7	1.2
40	0.9	1.4
50	1.4	1.7
60	2.0	2.3
70	2.2	2.4
80	2.7	2.9
90	2.7	3.4
100	2.8	3.8
110	2.9	4.1
120	3.2	4.5

2% $HReO_4$

t [min]	Y (%) RXN1	Y (%) RXN2
10	0.5	0.7
20	1.9	1.1
30	2.0	2.1
40	2.8	2.4
50	3.8	3.2
60	4.1	3.7
70	4.5	3.8
80	4.8	4.2
90	5.1	4.2
100	5.7	4.8
110	5.7	4.8
120	6.7	5.1

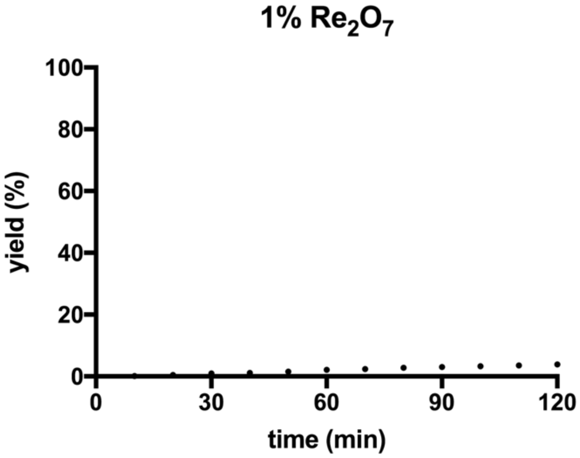

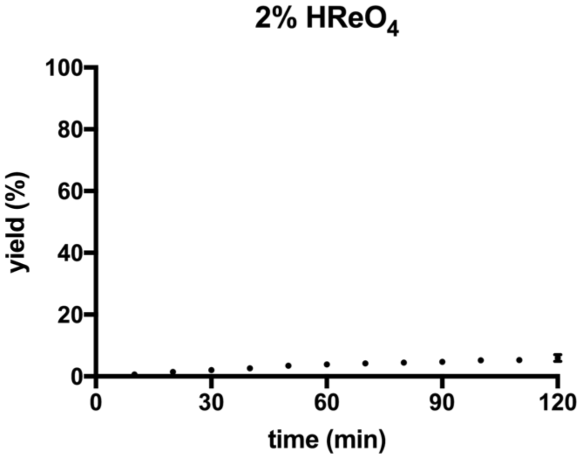

Competition studies

TfOH, low conversion

General reaction protocol A was followed with 3-(hydroxymethyl)benzyl acetate[2] **55** (35.0 mg, 0.19 mmol), *p*-xylene (143.7 µl, 1.17 mmol), trifluoromethanesulfonic acid (1.7 µl, 0.019 mmol) and HFIP (0.38 ml). The reaction mixture was stirred at ambient temperature for 1 h, then was quenched by 4-dimethylaminopyridine (3.1 mg, 0.025 mmol). The crude mixture was condensed under vacuum and purified by flash chromatography (hexane to 5% ethyl acetate in hexane) to give 1,3-bis(2,5-dimethylbenzyl)benzene **57** (8.6 mg, 14% yield) and 3-(2,5-dimethylbenzyl)benzyl acetate **56** (11.1 mg, 21% yield). Increasing the eluent polarity (30% ethyl acetate in hexane to 50% ethyl acetate in hexane) led to the recovery starting material (22.5 mg, 64%).

TfOH, high conversion

General reaction protocol A was followed with **55** (30.1 mg, 0.17 mmol), *p*-xylene (123.6 µl, 1.00 mmol), trifluoromethanesulfonic acid (1.5 µl, 0.017 mmol) and HFIP (0.34 ml). The reaction mixture was stirred at ambient temperature for 2.25 h, then was quenched by 4-dimethylaminopyridine. The crude mixture was condensed under vacuum and purified by flash chromatography (hexane to 5% ethyl acetate in hexane) to give **57** (39.4 mg, 75% yield) and **56** (11.1 mg, 21% yield). Increasing the eluent polarity (30% ethyl acetate in hexane to 50% ethyl acetate in hexane) led to the recovery starting material (3.2 mg, 10%).

Re_2O_7, low conversion

General reaction protocol A was followed with **55** (37.2 mg, 0.21 mmol), *p*-xylene (152.9 µl, 1.24 mmol), Re_2O_7 (1.0 mg, 0.002 mmol) and HFIP (0.42 ml). The reaction mixture was stirred at ambient temperature for 1 h, then was quenched by 4-dimethylaminopyridine. The crude mixture was condensed under vacuum and purified by flash chromatography (hexane to 5% ethyl acetate in hexane) to give **57** (1.2 mg, 2% yield) and **56** (28.6 mg, 52% yield). Increasing the eluent polarity (30% ethyl acetate in hexane to 50% ethyl acetate in hexane) led to the recovery starting material (17.2 mg, 46%).

Re_2O_7, high conversion

General reaction protocol A was followed with **55** (29.8 mg, 0.17 mmol), *p*-xylene (122.3 µl, 0.99 mmol), Re_2O_7 (0.8 mg, 0.0017 mmol) and HFIP (0.34 ml). The reaction mixture was stirred at ambient temperature for 2.25 h, then was quenched by 4-dimethylaminopyridine. The crude mixture was condensed under vacuum and purified by flash chromatography (hexane to 5% ethyl acetate in hexane) to give **57** (9.3 mg, 17% yield) and **56** (34.7 mg, 76% yield). Increasing the

[2] M. Dow, F. Marchetti, K. A. Abrahams, L. Vaz, G. S. Besra, S. Warriner and A. Nelson, *Chem. Eur. J.* 2017, **23**, 7207.

eluent polarity (30% ethyl acetate in hexane to 50% ethyl acetate in hexane) led to the recovery starting material (1.7 mg, 5%).

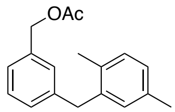

3-(2,5-Bimethylbenzyl)benzyl acetate (56)

^{1}H NMR (400 MHz, $CDCl_3$) δ 7.27-7.22 (m, 1H), 7.17 (d, *J* = 7.6 Hz, 1H), 7.12 (s, 1H), 7.09-7.03 (m, 2H), 6.96 (d, *J* = 7.6 Hz, 1H), 6.92 (s, 1H), 5.05 (s, 2H), 3.95 (s, 2H), 2.29 (s, 3H), 2.19 (s, 3H), 2.08 (s, 3H); ^{13}C NMR (100 MHz, $CDCl_3$) δ 171.0, 141.1, 138.5, 136.1, 135.6, 133.5, 130.9, 130.4, 128.8, 128.7, 128.7, 127.3, 125.9, 66.5, 39.4, 21.2, 21.1, 19.3; HRMS (ESI) $C_{18}H_{20}O_2$ $[M]^+$: *m/z* calcd. 268.1463; found 268.1489.

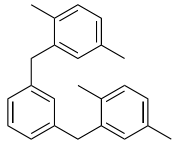

1,3-bis(2,5-dimethylbenzyl)benzene (57)

^{1}H NMR (400 MHz, $CDCl_3$) δ 7.19-7.12 (m, 1H), 7.03 (d, *J* = 7.6 Hz, 2H), 6.97-6.90 (m, 5H), 6.88 (s, 2H), 3.89 (s, 4H), 2.27 (s, 6H), 2.17 (s, 6H); ^{13}C NMR (100 MHz, $CDCl_3$) δ 140.6, 139.0, 135.4, 133.5, 130.8, 130.3, 129.6, 128.6, 127.1, 126.4, 39.5, 21.1, 19.3; HRMS (ESI) $C_{24}H_{26}$ $[M]^+$: *m/z* calcd. 314.2035; found 314.2044.

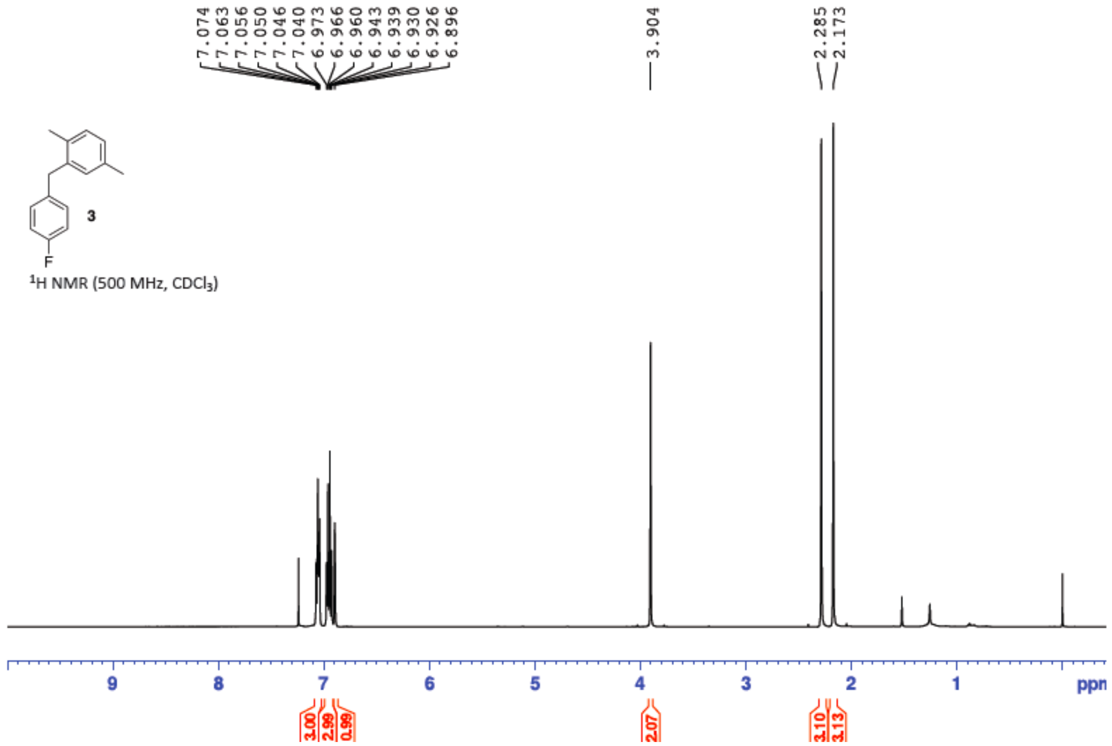

7.074
7.063
7.056
7.050
7.046
7.040
6.973
6.966
6.960
6.943
6.939
6.930
6.926
6.896
3.904
2.285
2.173
3
F
¹H NMR (500 MHz, CDCl₃)
9
8
7
6
5
4
3
2
1
ppm
3.00
2.99
0.99
2.07
3.10
3.13

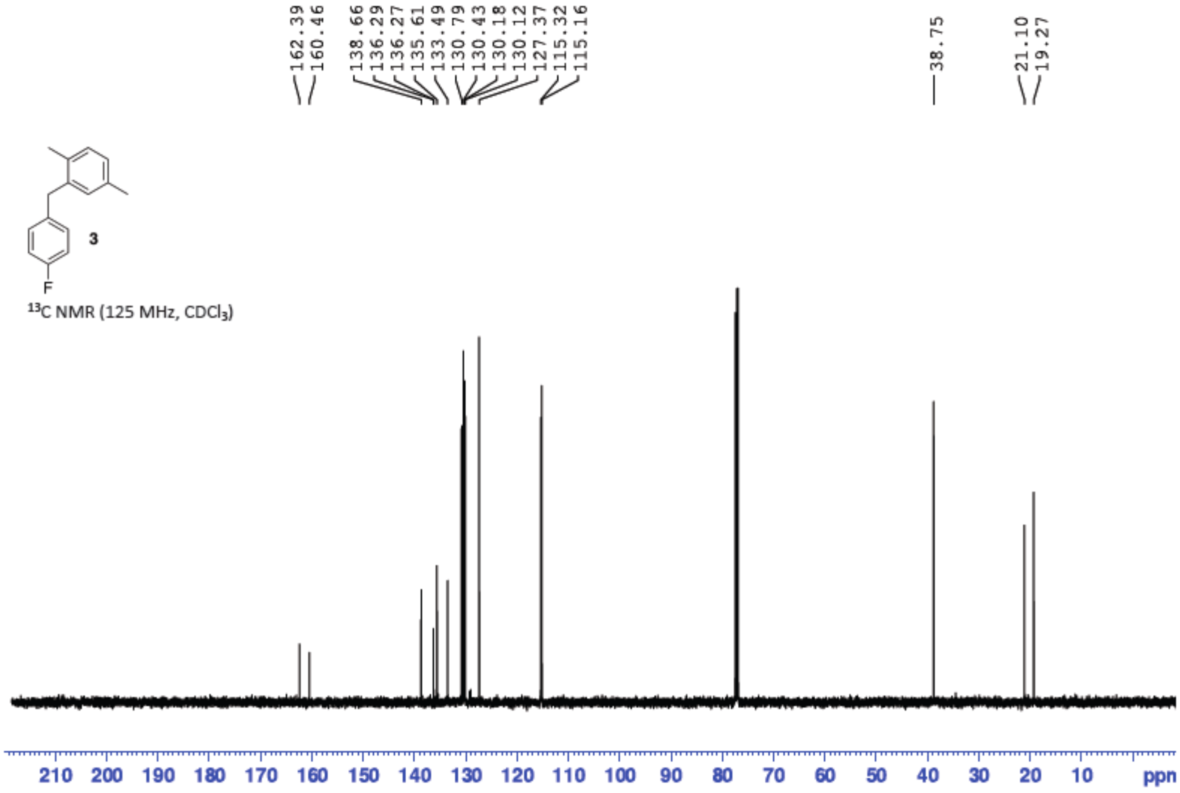

162.39
160.46
138.66
136.29
136.27
135.61
133.49
130.79
130.43
130.18
130.12
127.37
115.32
115.16
38.75
21.10
19.27
3
F
¹³C NMR (125 MHz, CDCl₃)
210
200
190
180
170
160
150
140
130
120
110
100
90
80
70
60
50
40
30
20
10
ppm

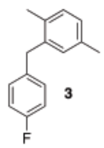

3

^{19}F NMR (470.4 MHz, $CDCl_3$; CF_3CO_2H - ext. std.)

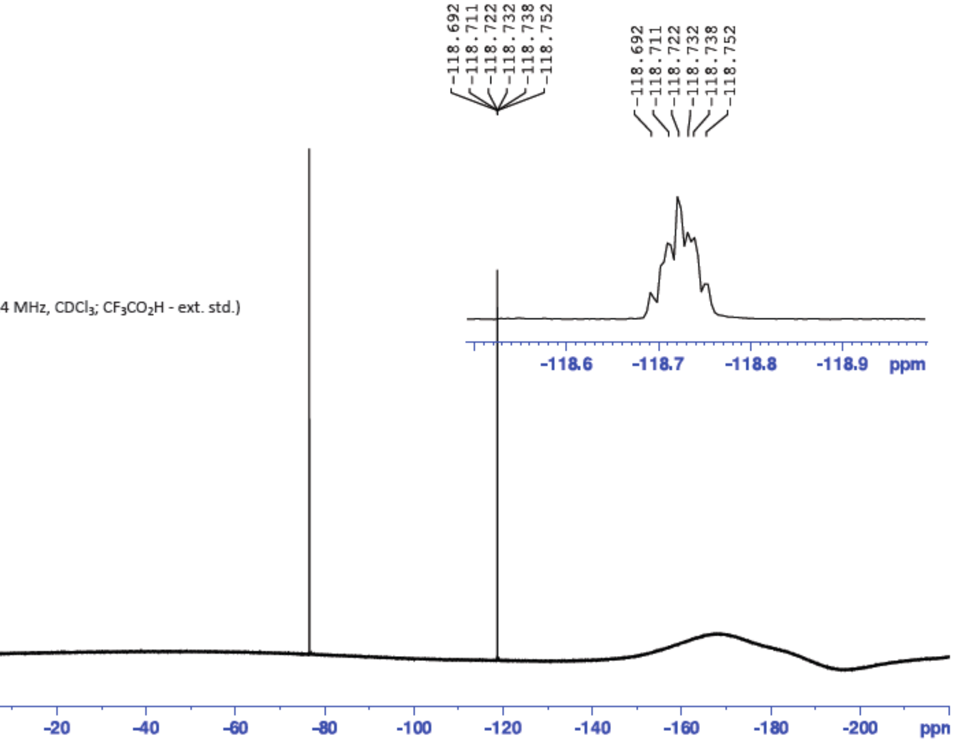

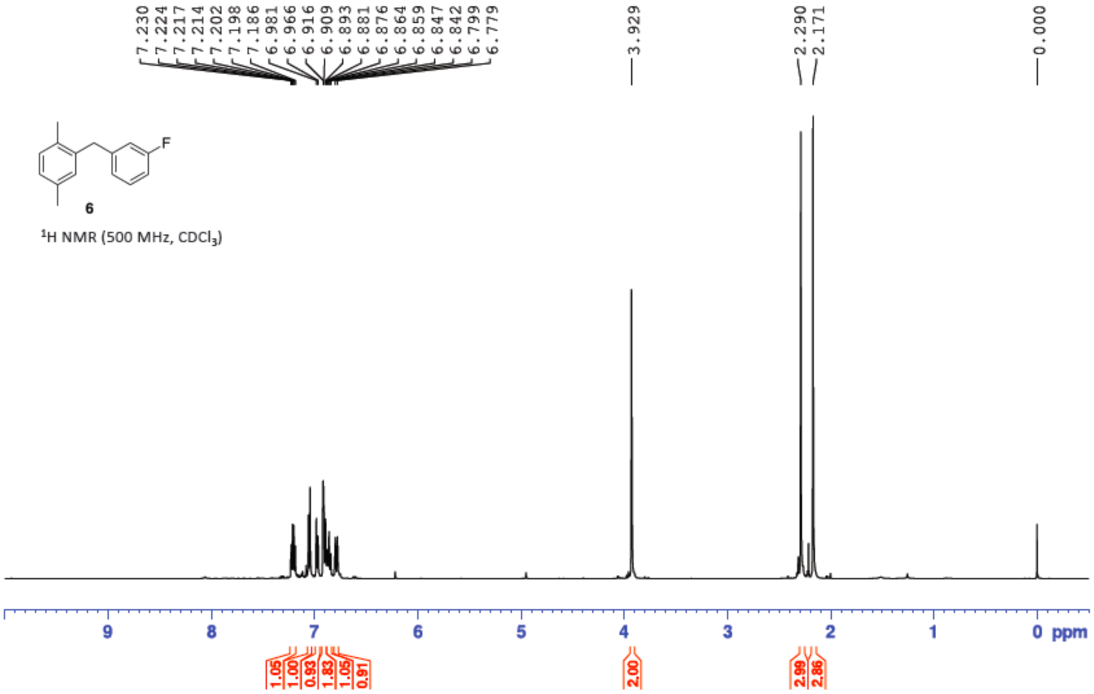
7.230 7.224 7.217 7.214 7.202 7.198 7.186 6.981 6.966 6.916 6.909 6.893 6.881 6.876 6.864 6.859 6.847 6.842 6.799 6.779
3.929
2.290 2.171
0.000
F
6
¹H NMR (500 MHz, CDCl₃)
9 8 7 6 5 4 3 2 1 0 ppm
1.05 1.00 0.93 1.83 1.05 0.91
2.00
2.99 2.86

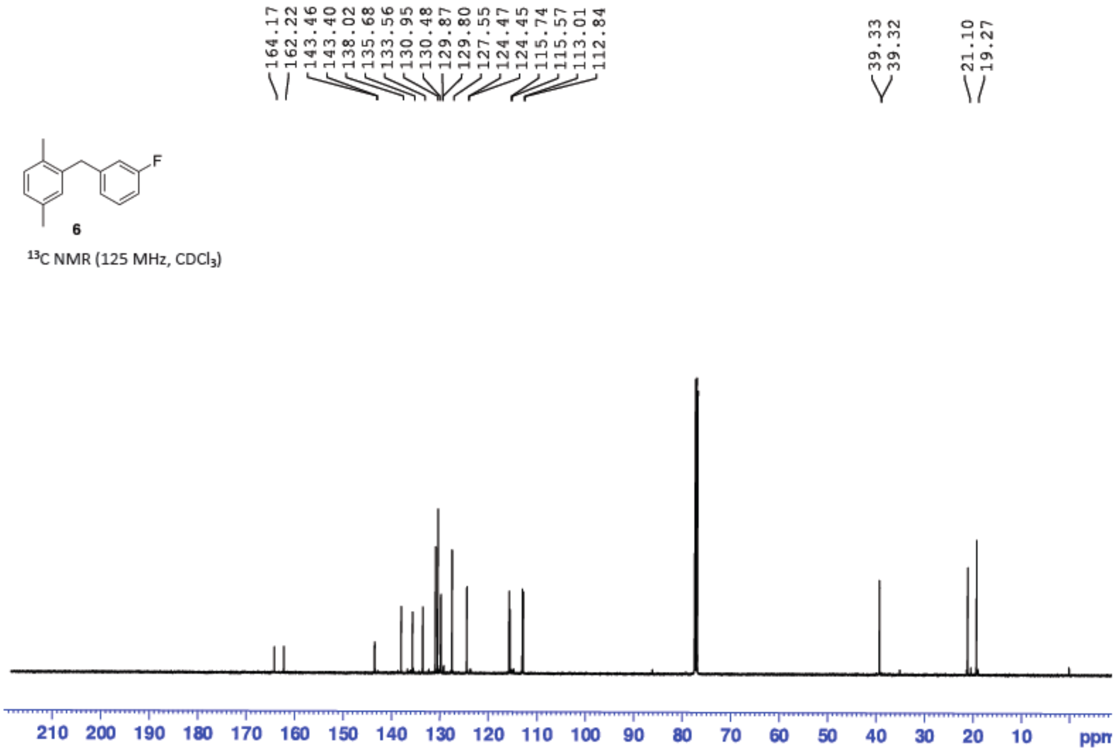
164.17 162.22 143.46 143.40 138.02 135.68 133.56 130.95 130.48 129.87 129.80 127.55 124.47 124.45 115.74 115.57 113.01 112.84
39.33 39.32
21.10 19.27
F
6
¹³C NMR (125 MHz, CDCl₃)
210 200 190 180 170 160 150 140 130 120 110 100 90 80 70 60 50 40 30 20 10 ppm

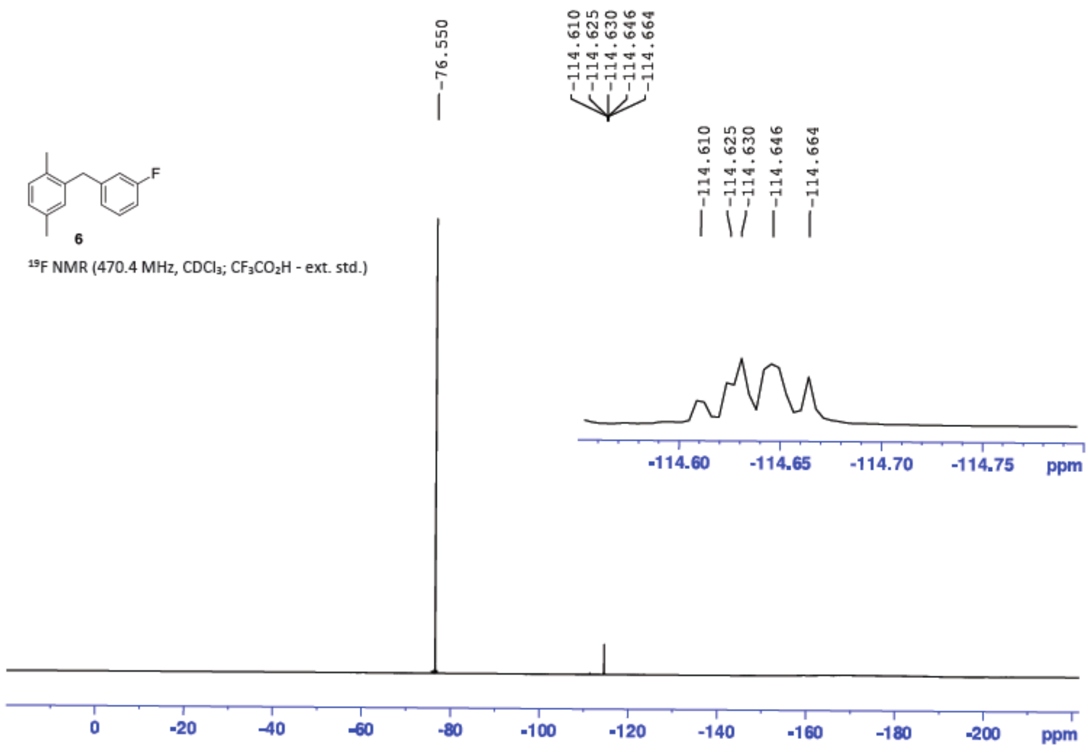

-76.550
-114.610
-114.625
-114.630
-114.646
-114.664
F
6
^{19}F NMR (470.4 MHz, $CDCl_3$; CF_3CO_2H - ext. std.)
-114.60
-114.65
-114.70
-114.75
ppm
0
-20
-40
-60
-80
-100
-120
-140
-160
-180
-200
ppm

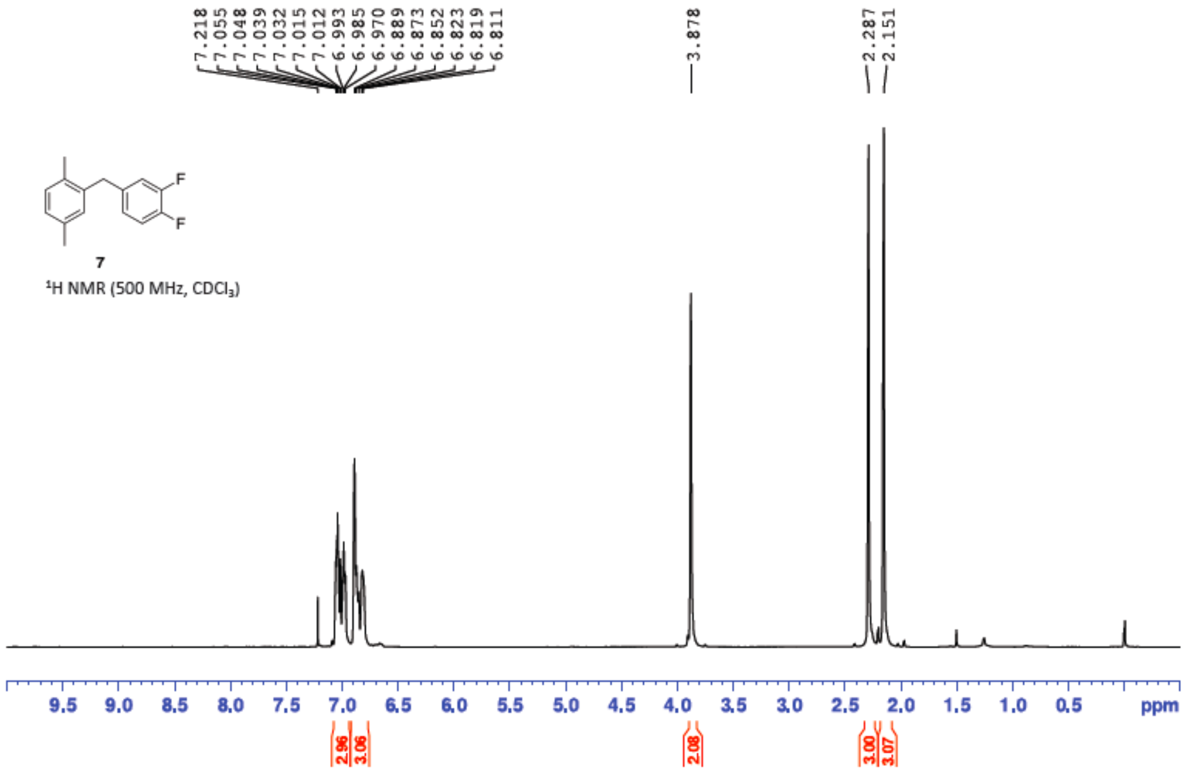
7
¹H NMR (500 MHz, CDCl₃)
ppm

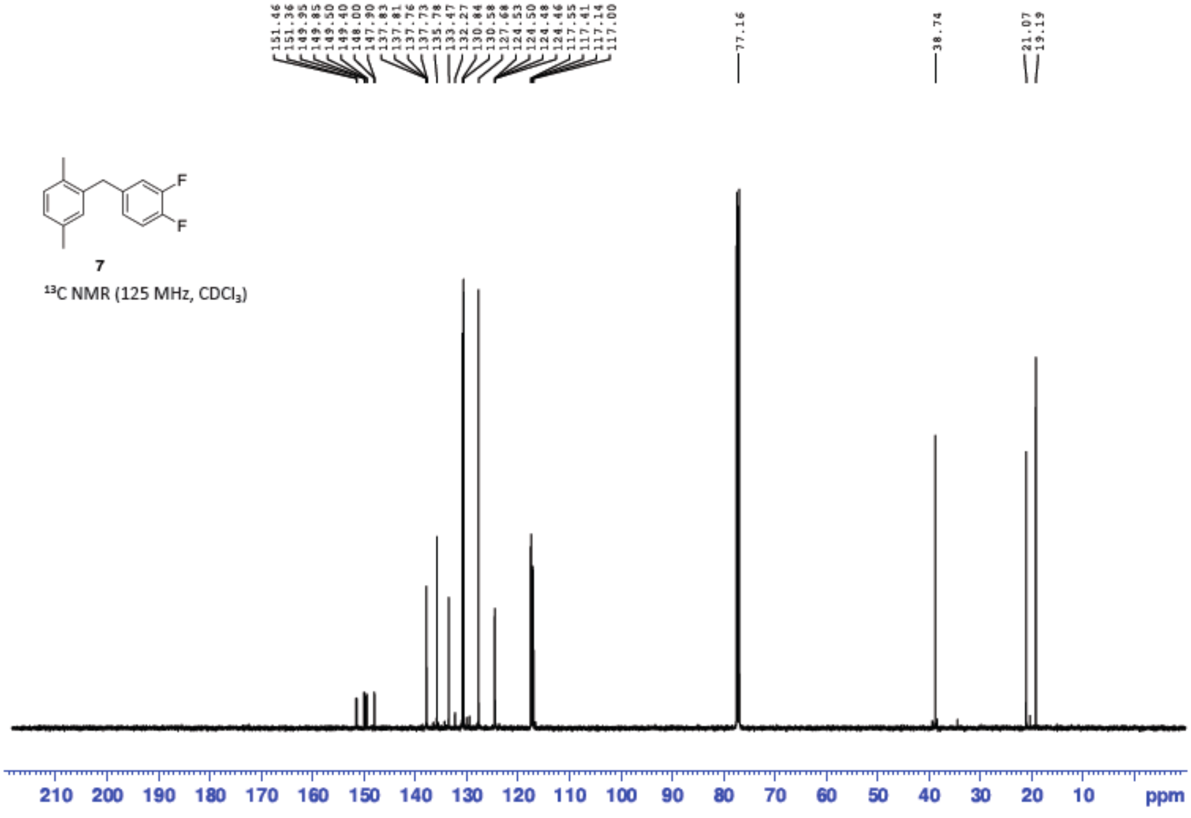
7
¹³C NMR (125 MHz, CDCl₃)
ppm

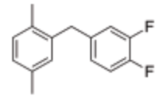

7

^{19}F NMR (470.4 MHz, $CDCl_3$; CF_3CO_2H - ext. std.)

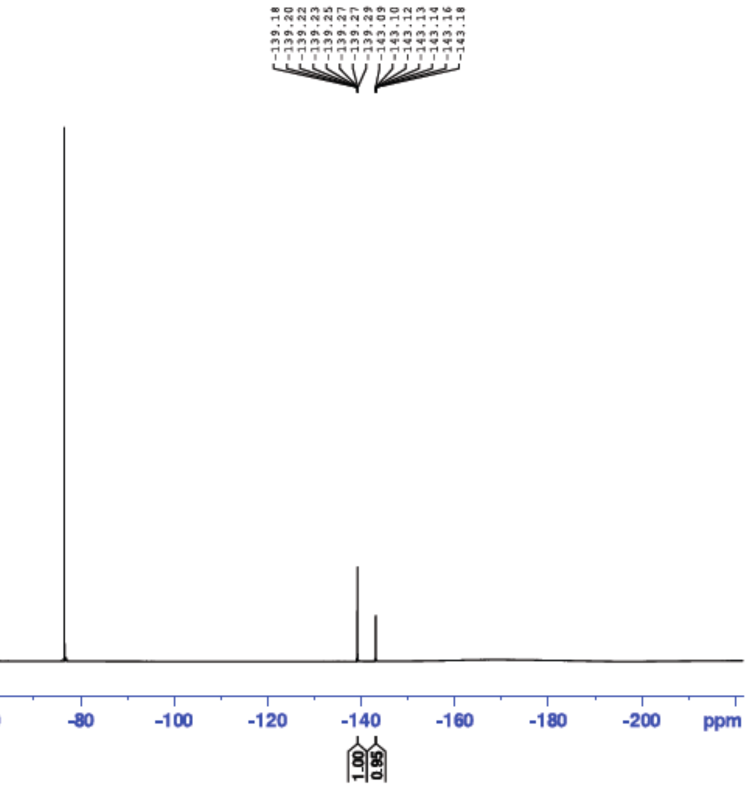

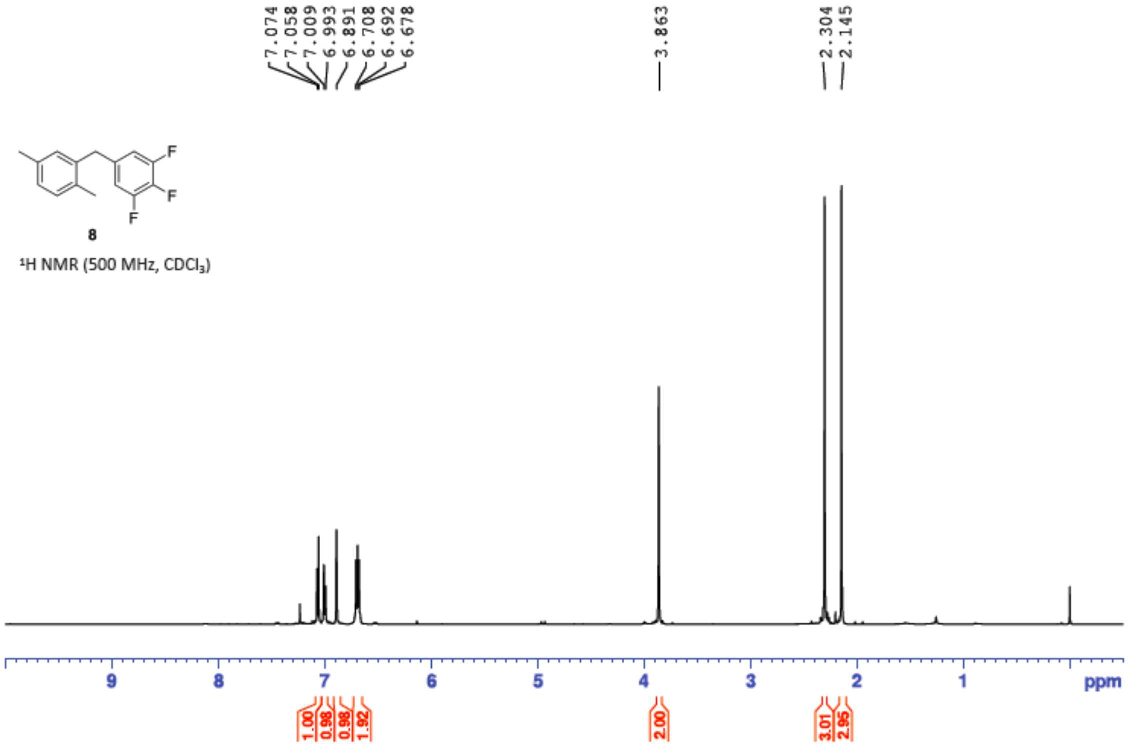

7.074
7.058
7.009
6.993
6.891
6.708
6.692
6.678
3.863
2.304
2.145
8
1H NMR (500 MHz, CDCl3)
9
8
7
6
5
4
3
2
1
ppm
1.00
0.98
0.98
1.92
2.00
3.01
2.95

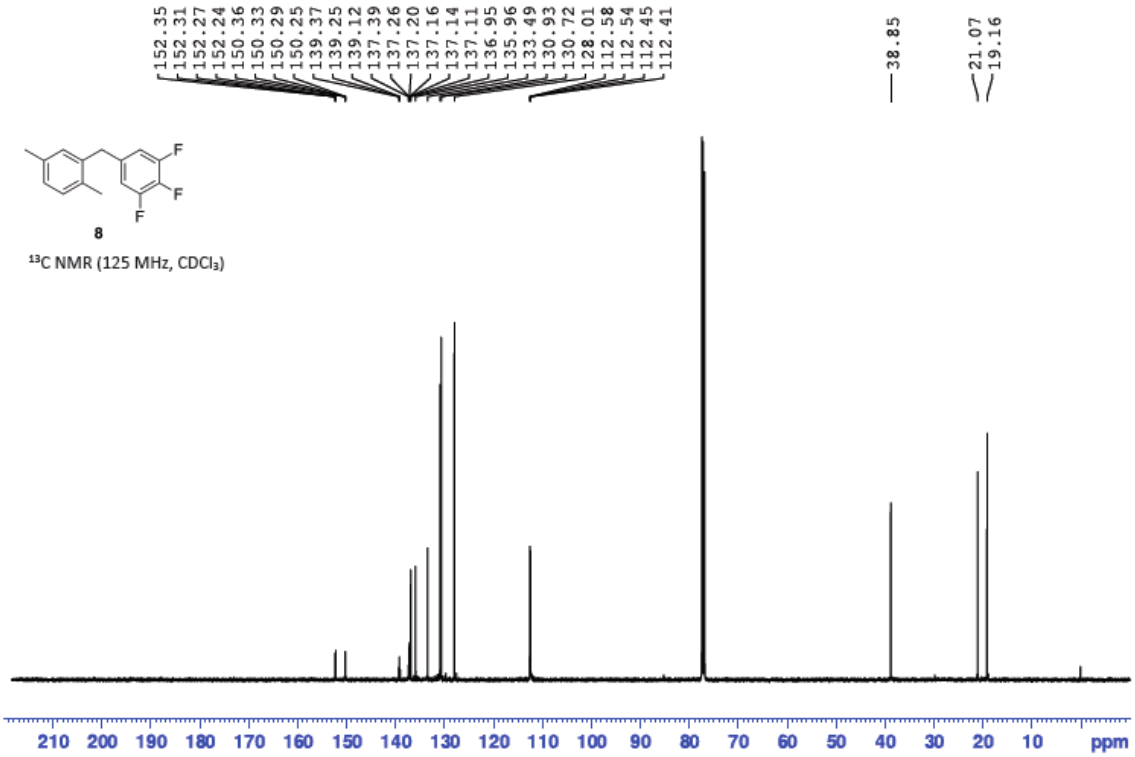

152.35
152.31
152.27
152.24
150.36
150.33
150.29
150.25
139.37
139.25
139.12
137.39
137.26
137.20
137.16
137.14
137.11
136.95
135.96
133.49
130.93
130.72
128.01
112.58
112.54
112.45
112.41
38.85
21.07
19.16
8
13C NMR (125 MHz, CDCl3)
210
200
190
180
170
160
150
140
130
120
110
100
90
80
70
60
50
40
30
20
10
ppm

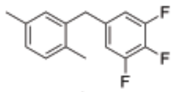

8

^{19}F NMR (470.4 MHz, $CDCl_3$; CF_3CO_2H - ext. std.)

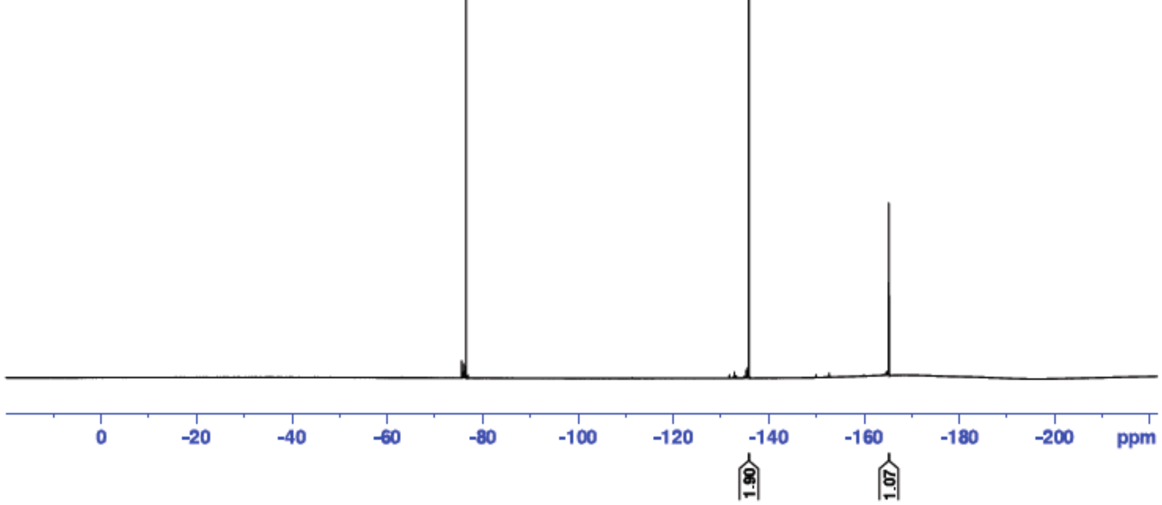

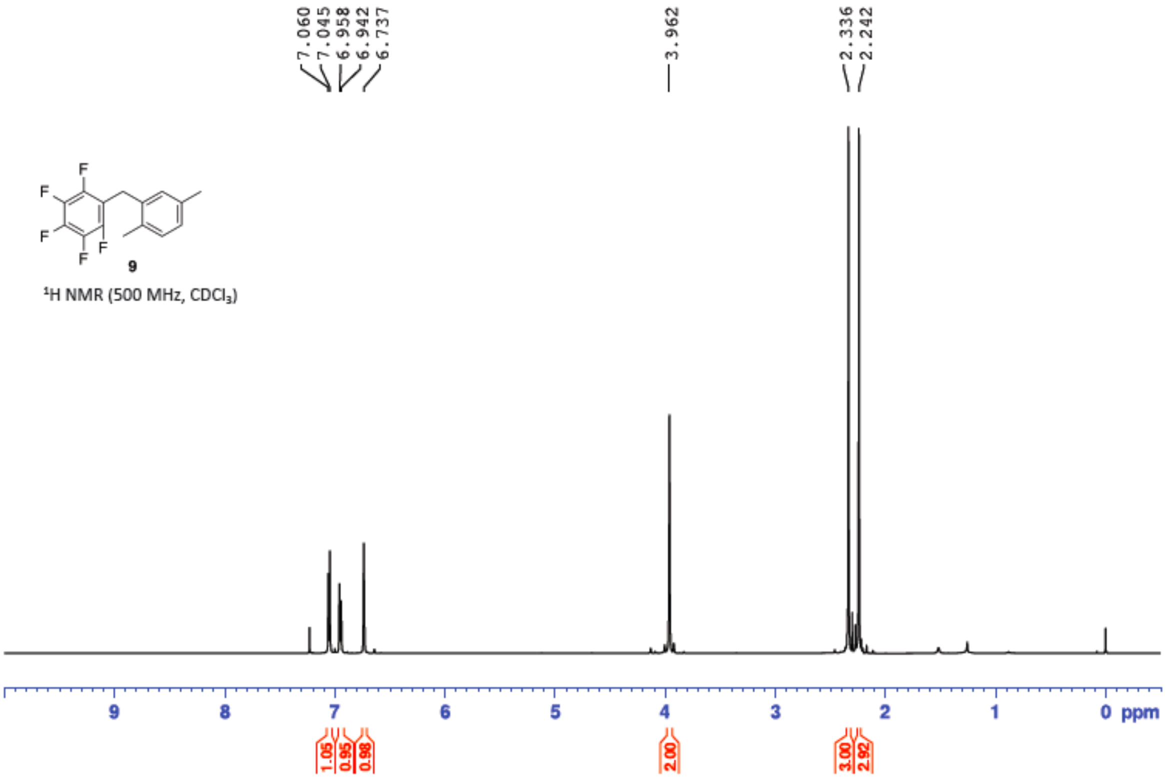
7.060
7.045
6.958
6.942
6.737
3.962
2.336
2.242
F
F
F
F
F
9
^{1}H NMR (500 MHz, CDCl$_3$)
9
8
7
6
5
4
3
2
1
0 ppm
1.05
0.95
0.98
2.00
3.00
2.92

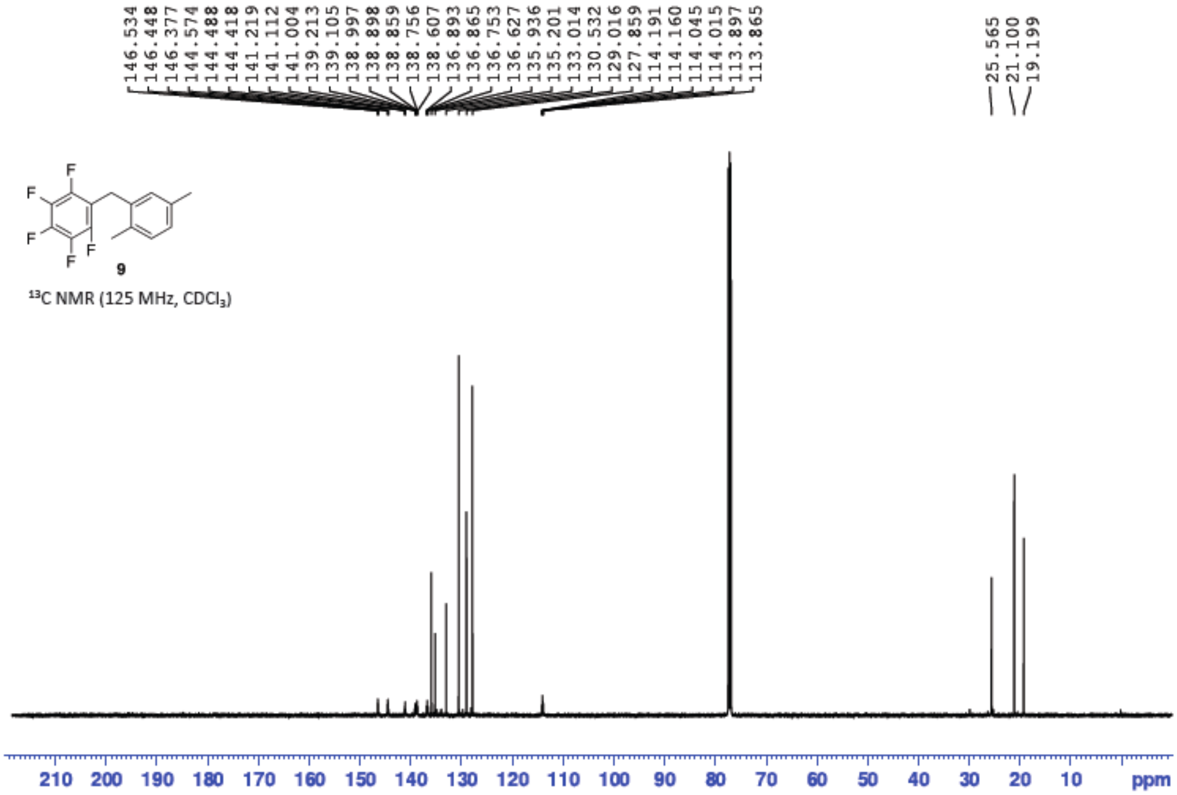
146.534
146.448
146.377
144.574
144.488
144.418
141.219
141.112
141.004
139.213
139.105
138.997
138.898
138.859
138.756
138.607
136.893
136.865
136.753
136.627
135.936
135.201
133.014
130.532
129.016
127.859
114.191
114.160
114.045
114.015
113.897
113.865
25.565
21.100
19.199
F
F
F
F
F
9
^{13}C NMR (125 MHz, CDCl$_3$)
210
200
190
180
170
160
150
140
130
120
110
100
90
80
70
60
50
40
30
20
10
ppm

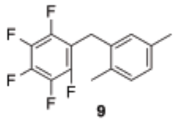

9

^{19}F NMR (470.4 MHz, $CDCl_3$; CF_3CO_2H - ext. std.)

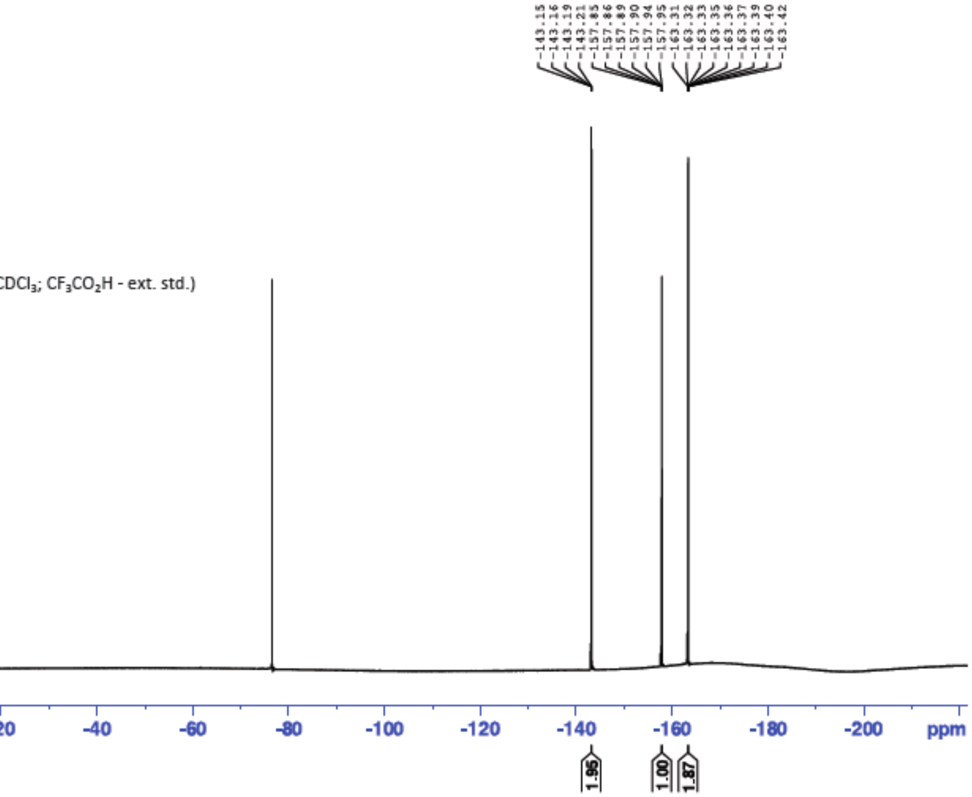

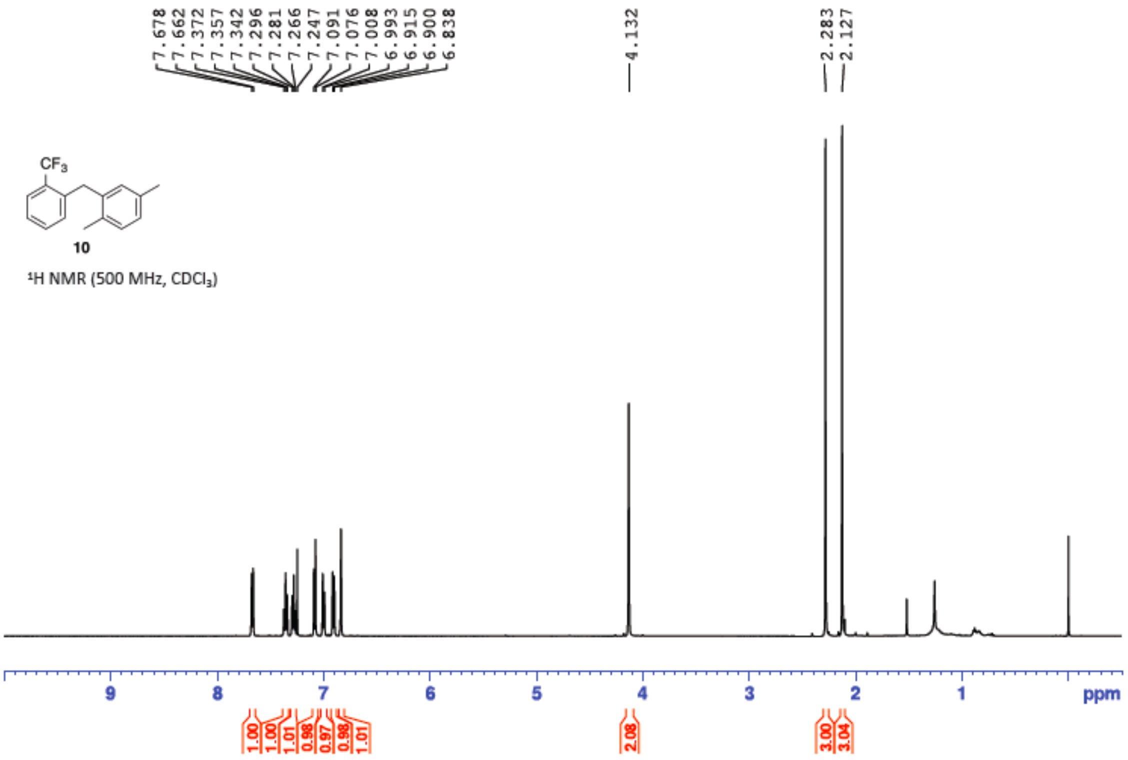
7.678
7.662
7.372
7.357
7.342
7.296
7.281
7.266
7.247
7.091
7.076
7.008
6.993
6.915
6.900
6.838
4.132
2.283
2.127
CF3
10
1H NMR (500 MHz, CDCl3)
9
8
7
6
5
4
3
2
1
ppm
1.00
1.00
1.01
0.98
0.97
0.98
1.01
2.08
3.00
3.04

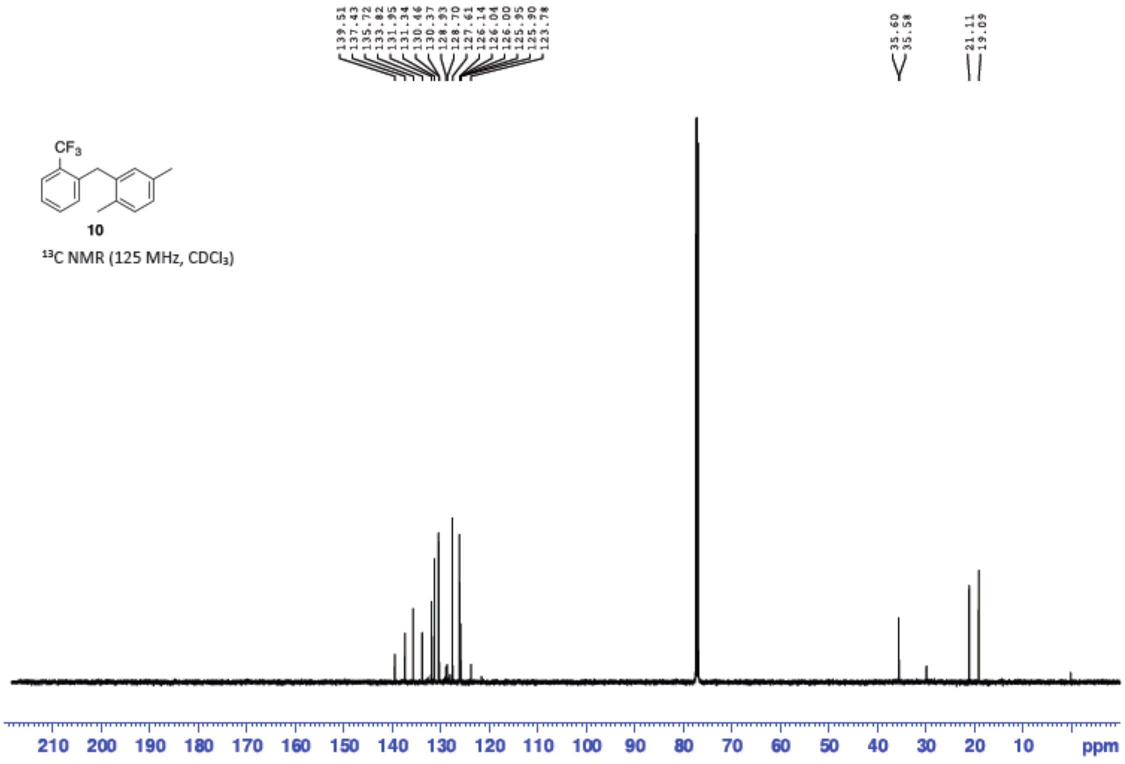
139.51
137.43
135.72
133.82
131.95
131.34
130.46
130.37
128.93
128.70
127.61
126.14
126.04
126.00
125.95
125.90
123.78
35.60
35.58
21.11
19.09
CF3
10
13C NMR (125 MHz, CDCl3)
210
200
190
180
170
160
150
140
130
120
110
100
90
80
70
60
50
40
30
20
10
ppm

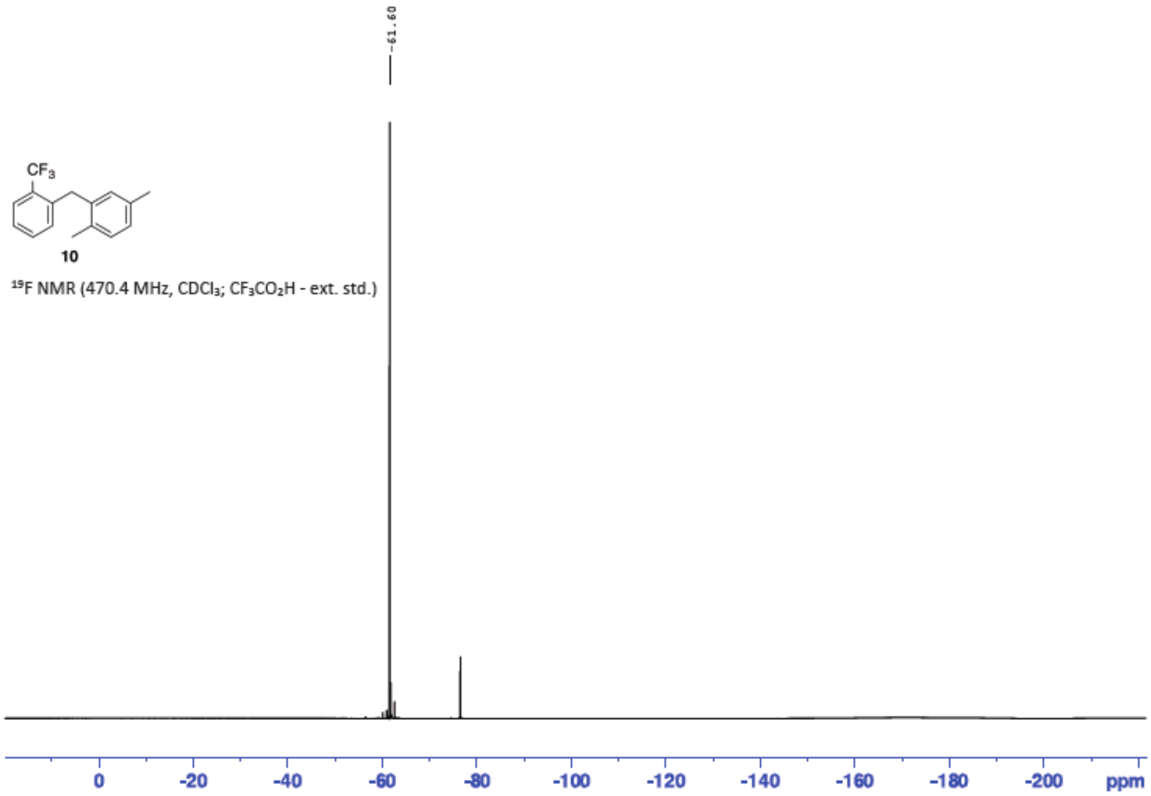

10

^{19}F NMR (470.4 MHz, $CDCl_3$; CF_3CO_2H - ext. std.)

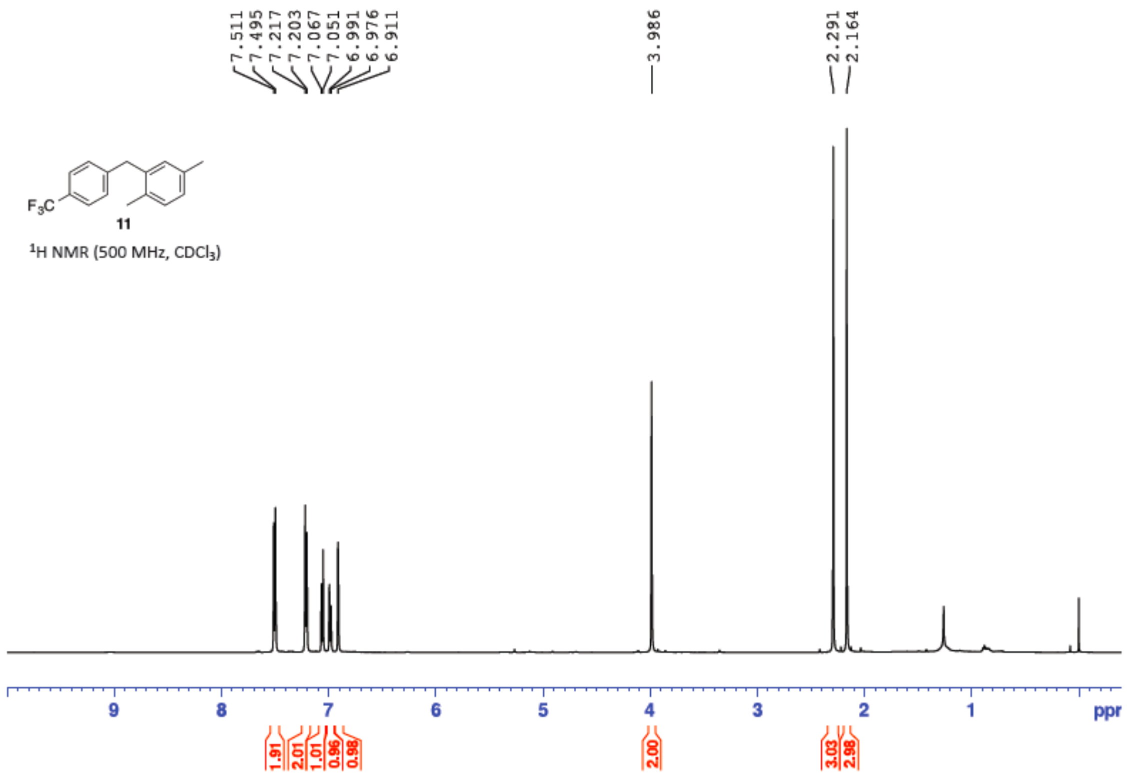
7.511
7.495
7.217
7.203
7.067
7.051
6.991
6.976
6.911
3.986
2.291
2.164
F3C
11
1H NMR (500 MHz, CDCl3)
9
8
7
6
5
4
3
2
1
ppm
1.91
2.01
1.01
0.96
0.98
2.00
3.03
2.98

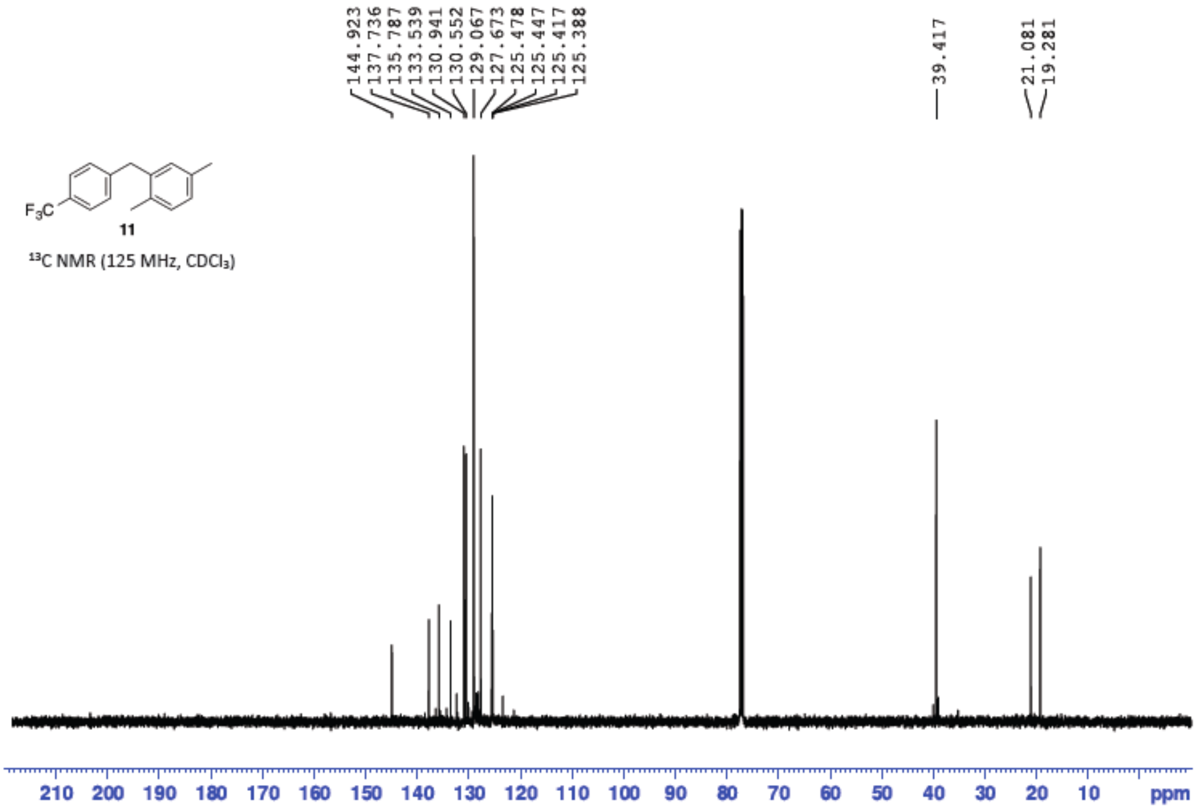
144.923
137.736
135.787
133.539
130.941
130.552
129.067
127.673
125.478
125.447
125.417
125.388
39.417
21.081
19.281
F3C
11
13C NMR (125 MHz, CDCl3)
210
200
190
180
170
160
150
140
130
120
110
100
90
80
70
60
50
40
30
20
10
ppm

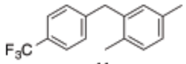

11

^{19}F NMR (470.4 MHz, $CDCl_3$; CF_3CO_2H - ext. std.)

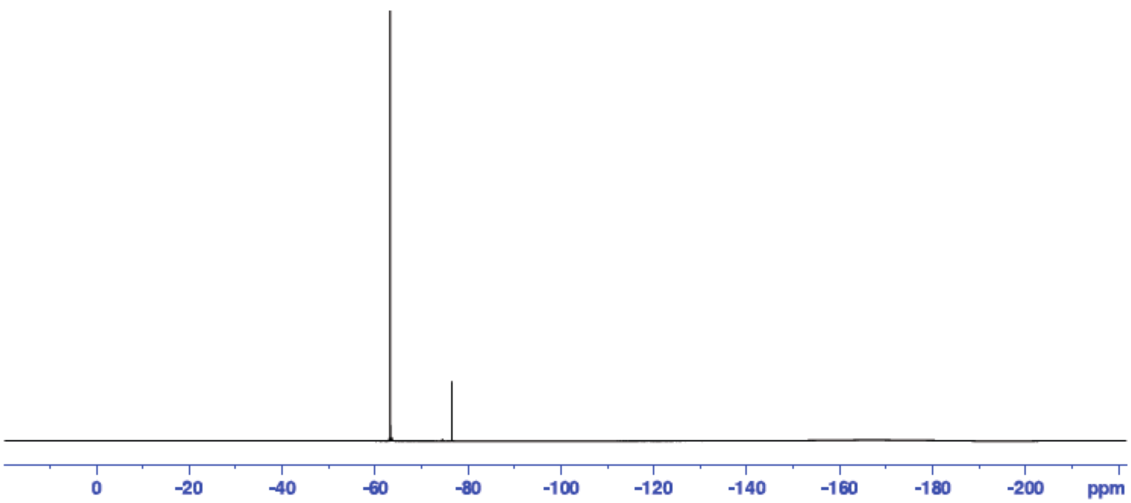

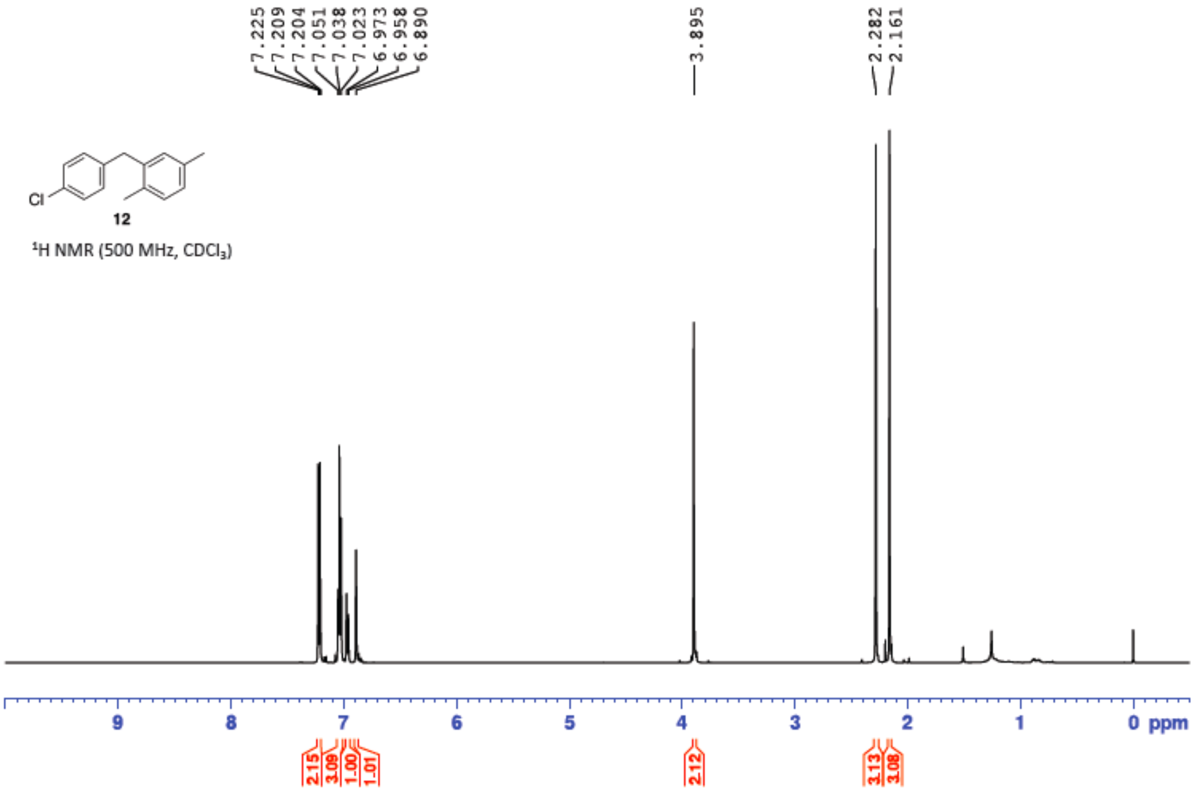
7.225
7.209
7.204
7.051
7.038
7.023
6.973
6.958
6.890
3.895
2.282
2.161
Cl
12
¹H NMR (500 MHz, CDCl₃)
9 8 7 6 5 4 3 2 1 0 ppm
2.15
3.09
1.00
1.01
2.12
3.13
3.08

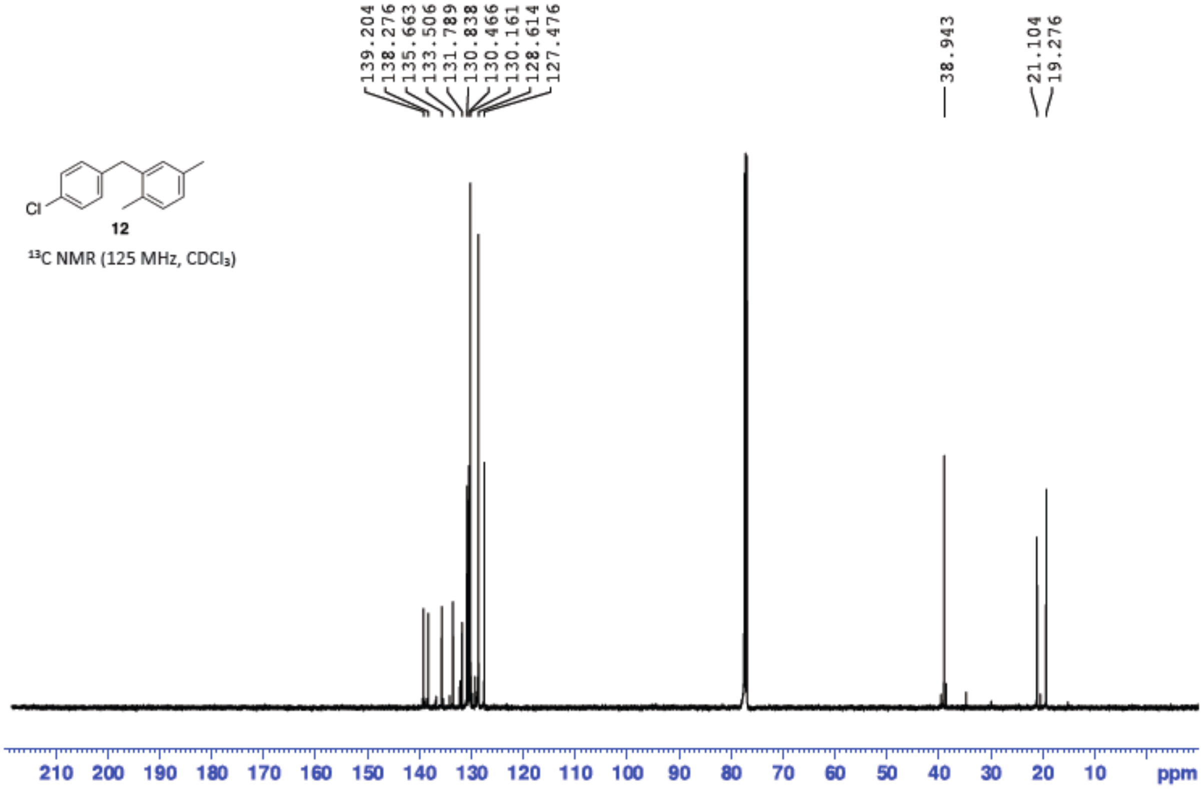
139.204
138.276
135.663
133.506
131.789
130.838
130.466
130.161
128.614
127.476
38.943
21.104
19.276
Cl
12
¹³C NMR (125 MHz, CDCl₃)
210 200 190 180 170 160 150 140 130 120 110 100 90 80 70 60 50 40 30 20 10 ppm

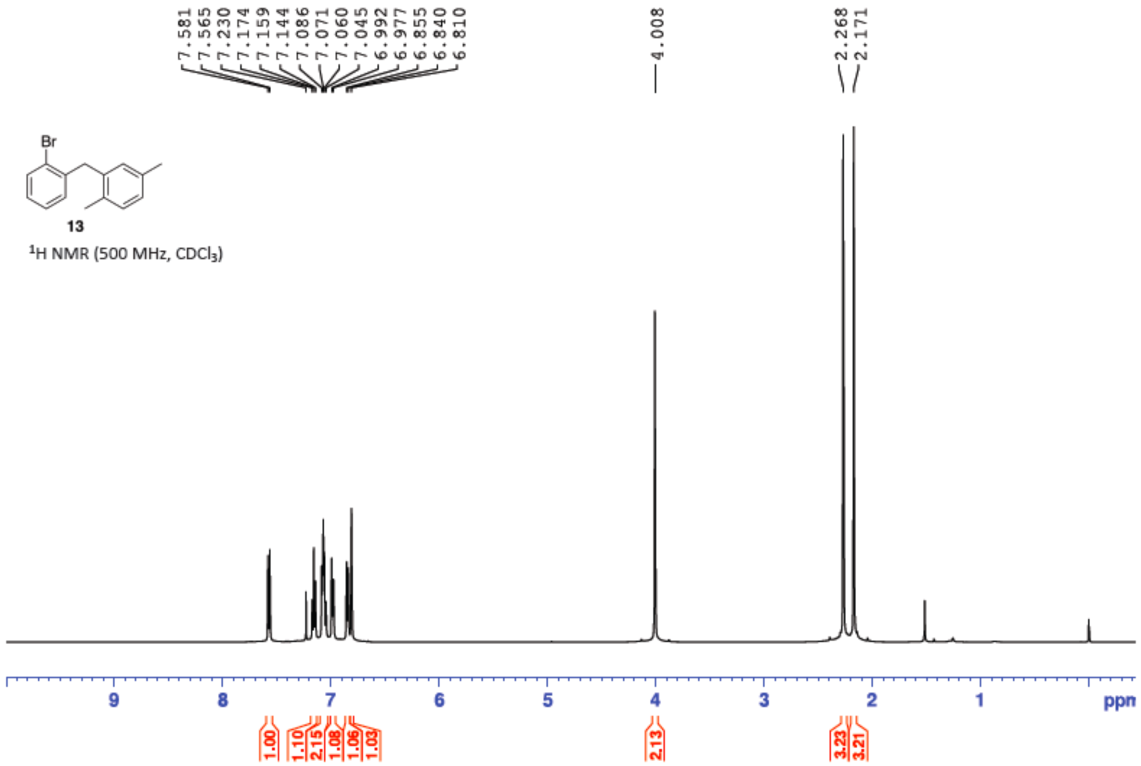
7.581
7.565
7.230
7.174
7.159
7.144
7.086
7.071
7.060
7.045
6.992
6.977
6.855
6.840
6.810
4.008
2.268
2.171
Br
13
^{1}H NMR (500 MHz, $CDCl_3$)
9
8
7
6
5
4
3
2
1
ppm
1.00
1.10
2.15
1.08
1.06
1.03
2.13
3.23
3.21

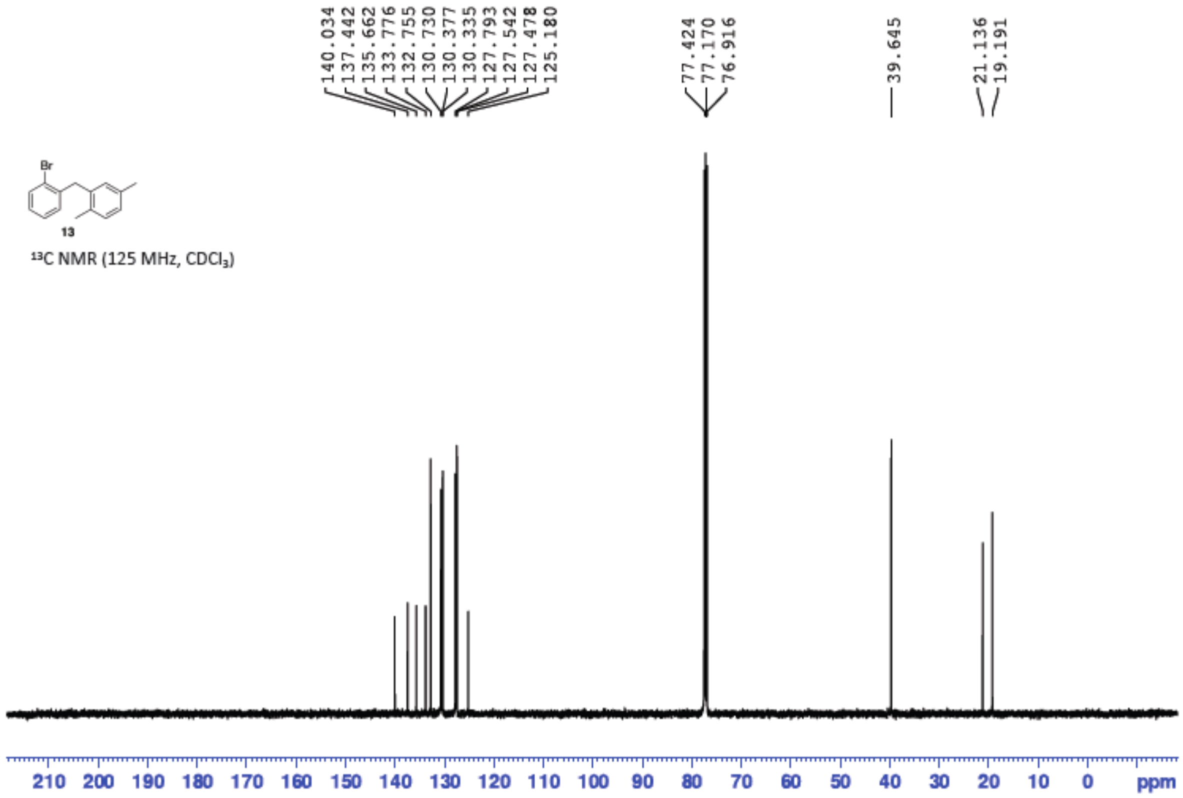
140.034
137.442
135.662
133.776
132.755
130.730
130.377
130.335
127.793
127.542
127.478
125.180
77.424
77.170
76.916
39.645
21.136
19.191
Br
13
^{13}C NMR (125 MHz, $CDCl_3$)
210 200 190 180 170 160 150 140 130 120 110 100 90 80 70 60 50 40 30 20 10 0 ppm

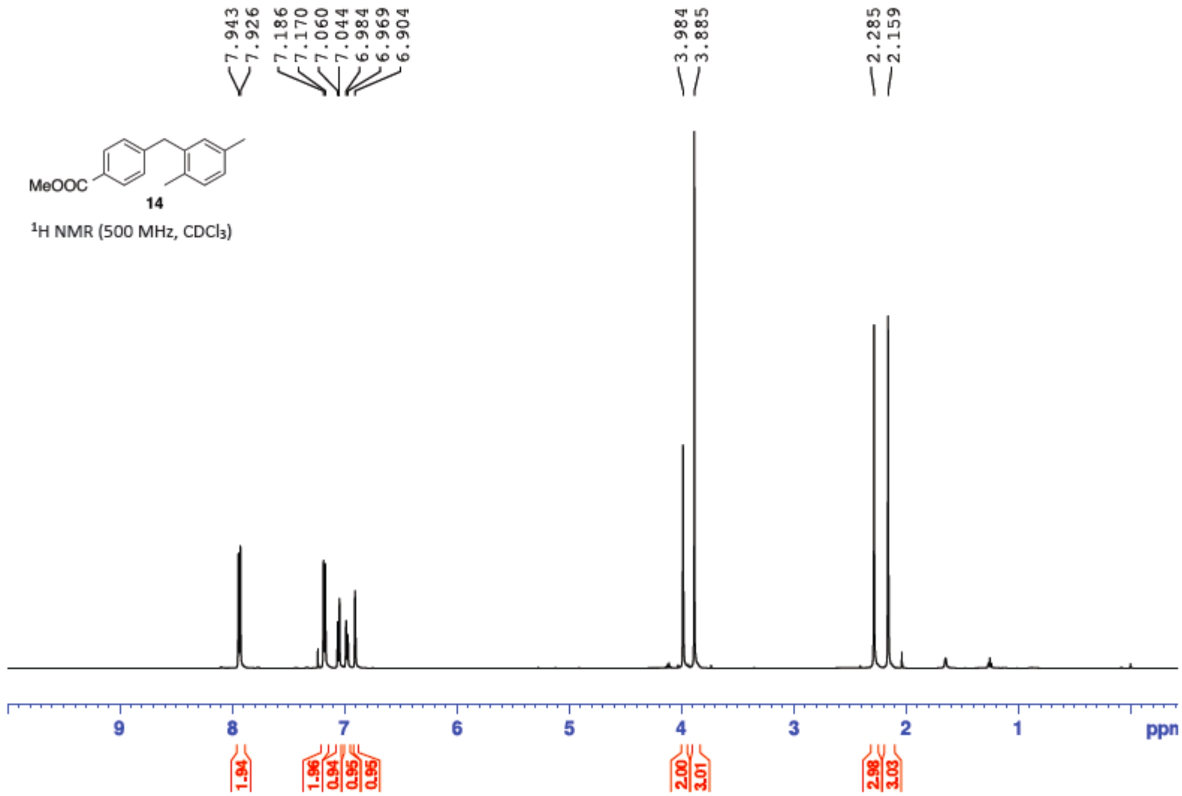
7.943
7.926
7.186
7.170
7.060
7.044
6.984
6.969
6.904
3.984
3.885
2.285
2.159
MeOOC
14
1H NMR (500 MHz, CDCl3)
9
8
7
6
5
4
3
2
1
ppm
1.94
1.96
0.94
0.95
0.95
2.00
3.01
2.98
3.03

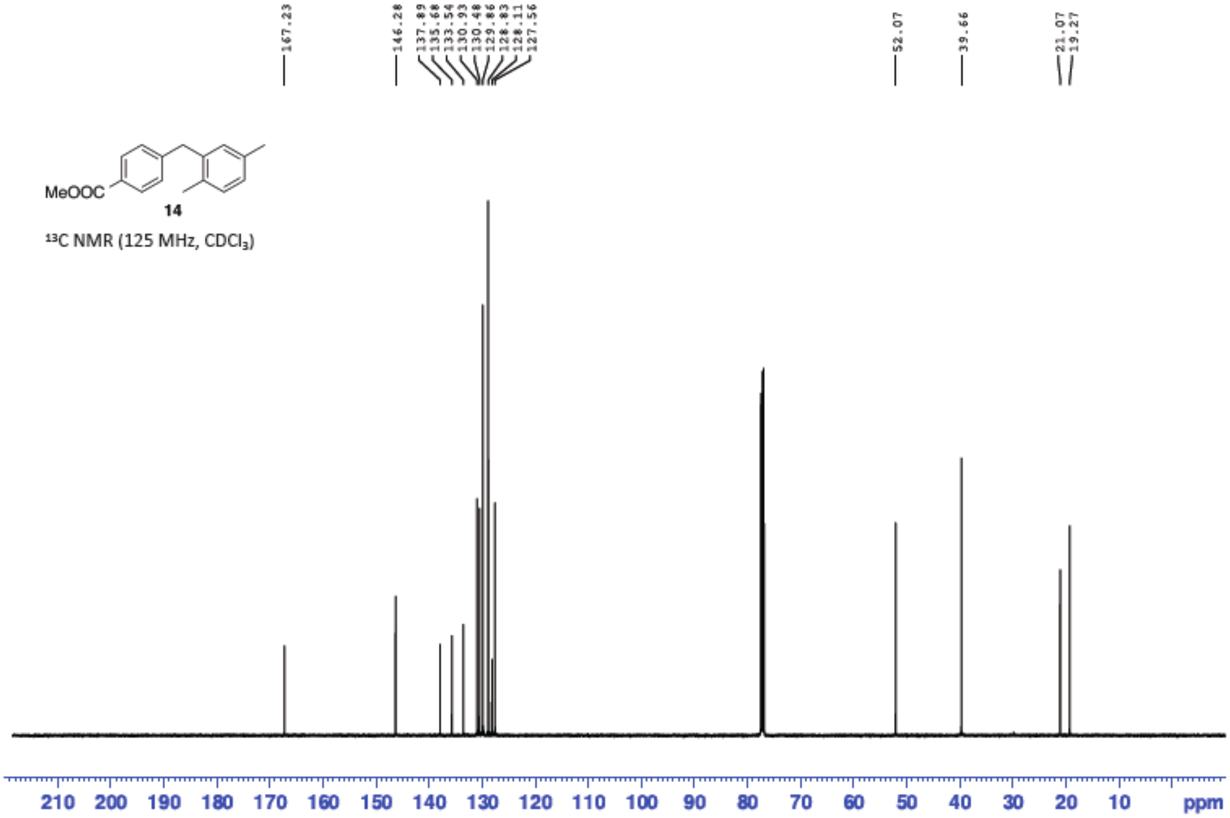
167.23
146.28
137.89
135.68
133.54
130.93
130.48
129.86
128.83
128.11
127.56
52.07
39.66
21.07
19.27
MeOOC
14
13C NMR (125 MHz, CDCl3)
210
200
190
180
170
160
150
140
130
120
110
100
90
80
70
60
50
40
30
20
10
ppm

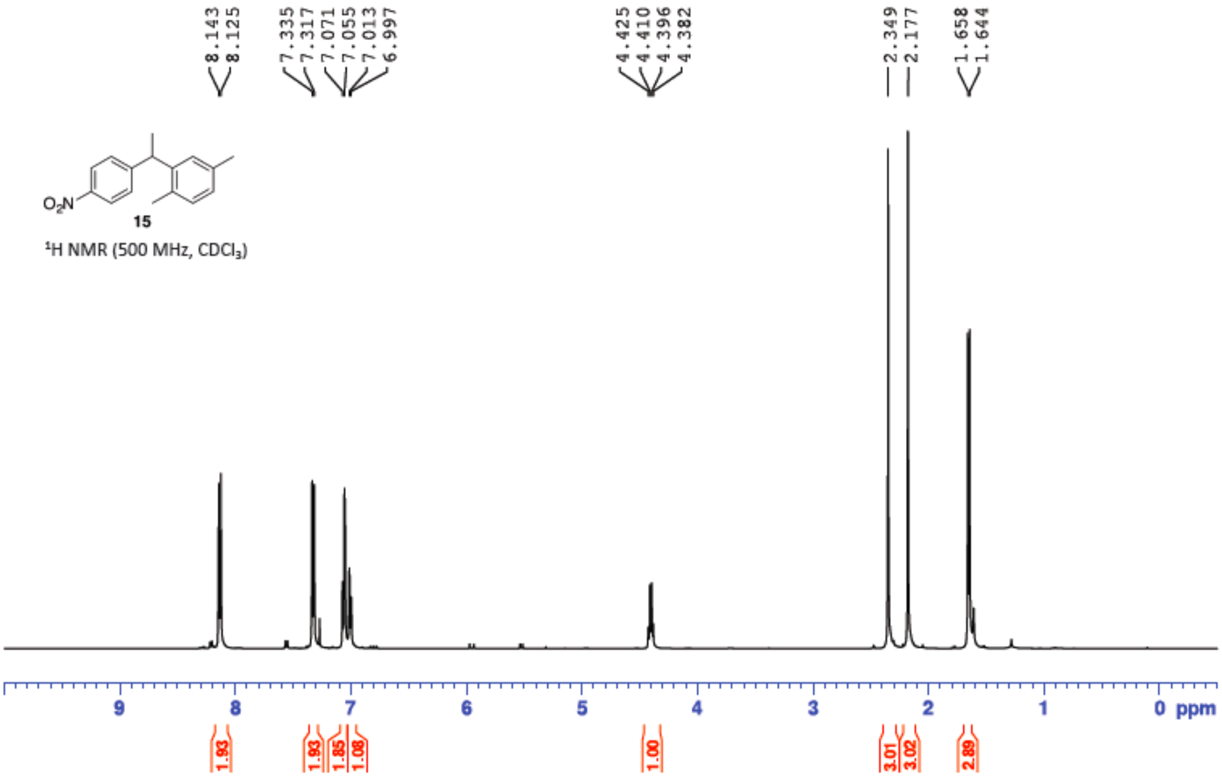
8.143
8.125
7.335
7.317
7.071
7.055
7.013
6.997
4.425
4.410
4.396
4.382
2.349
2.177
1.658
1.644
O2N
15
1H NMR (500 MHz, CDCl3)
9
8
7
6
5
4
3
2
1
0 ppm
1.93
1.93
1.85
1.08
1.00
3.01
3.02
2.89

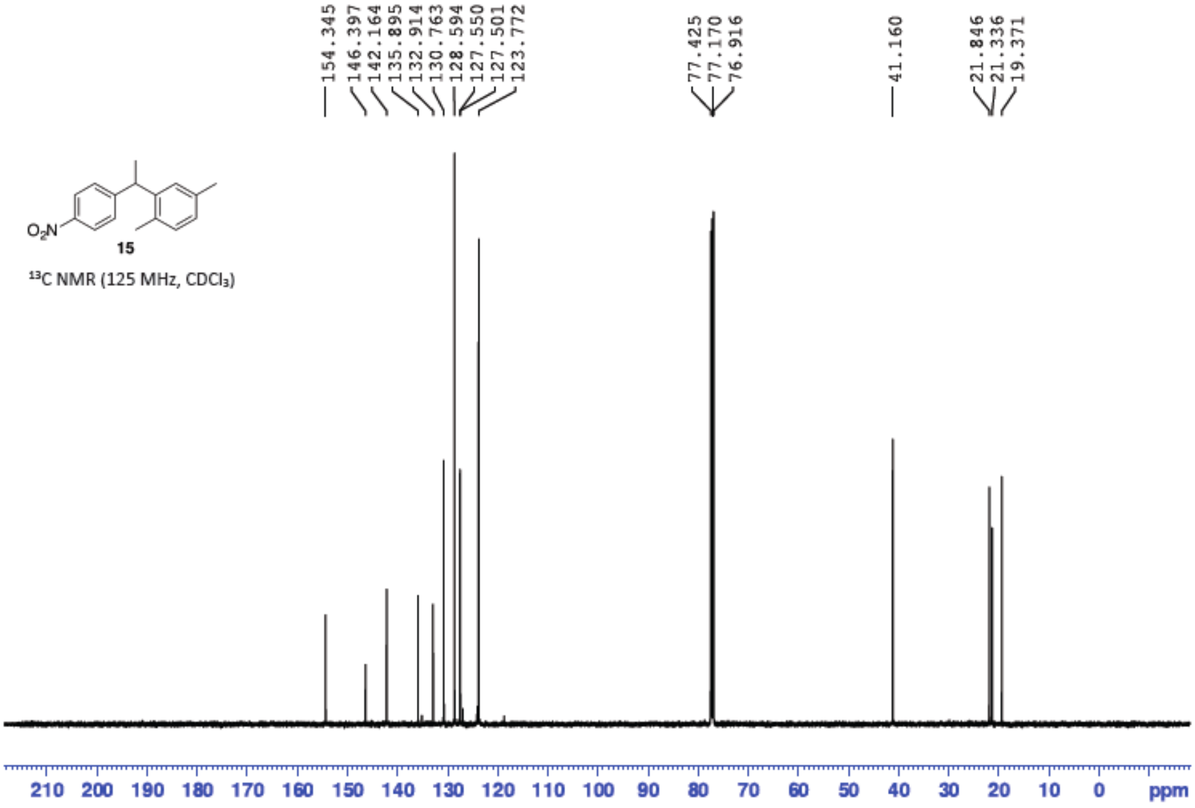
154.345
146.397
142.164
135.895
132.914
130.763
128.594
127.550
127.501
123.772
77.425
77.170
76.916
41.160
21.846
21.336
19.371
O2N
15
13C NMR (125 MHz, CDCl3)
210
200
190
180
170
160
150
140
130
120
110
100
90
80
70
60
50
40
30
20
10
0
ppm

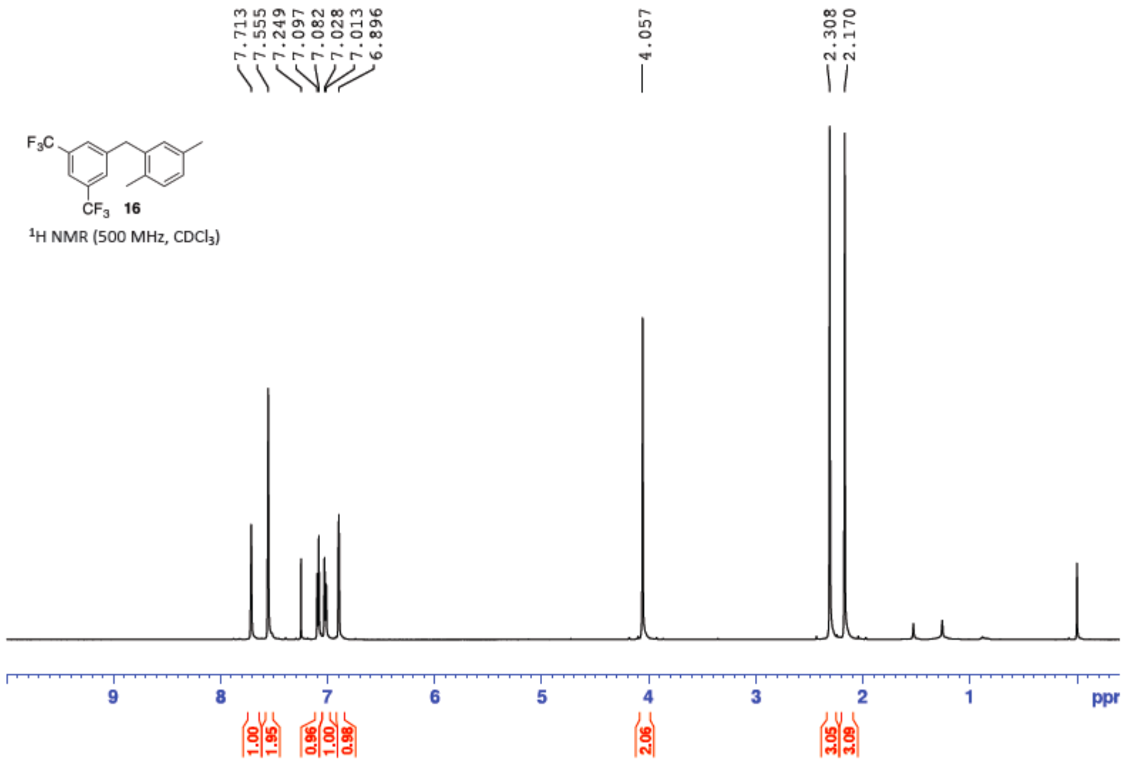
7.713
7.555
7.249
7.097
7.082
7.028
7.013
6.896
4.057
2.308
2.170
F3C
CF3
16
1H NMR (500 MHz, CDCl3)
1.00
1.95
0.96
1.00
0.98
2.06
3.05
3.09
ppm

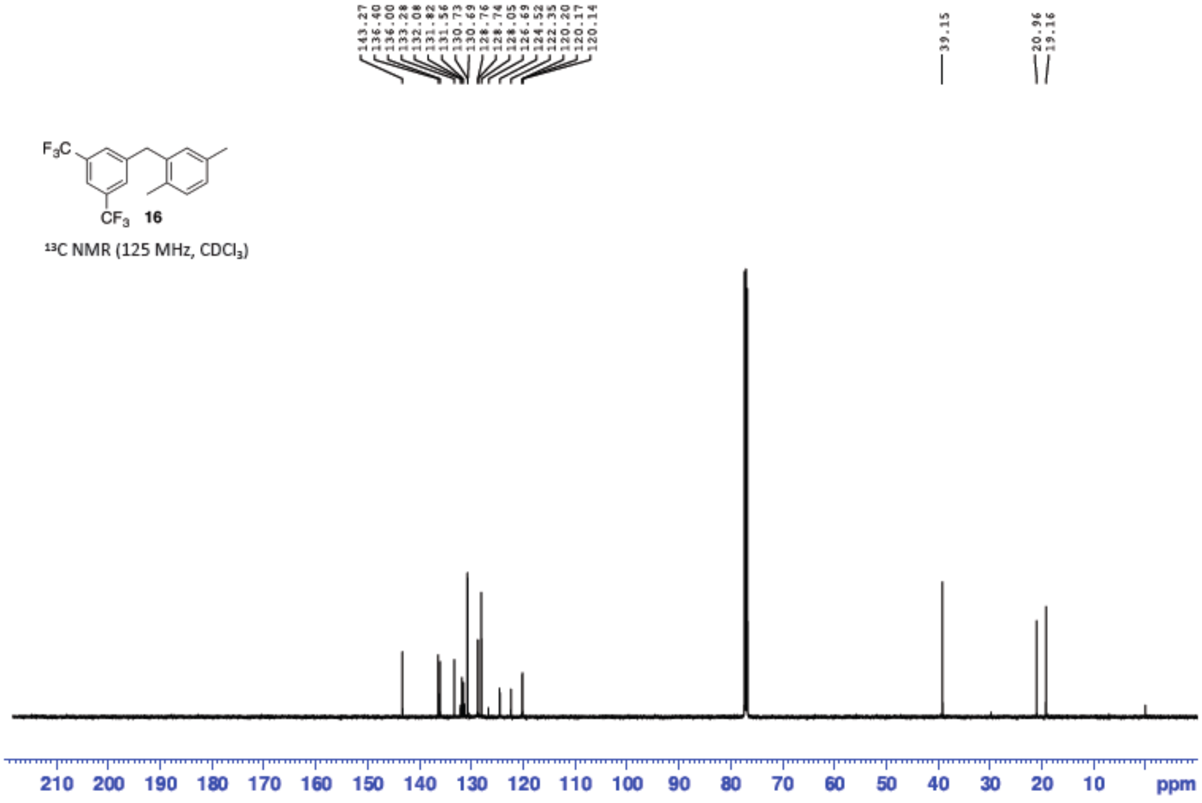
39.15
20.96
19.16
F3C
CF3
16
13C NMR (125 MHz, CDCl3)
ppm

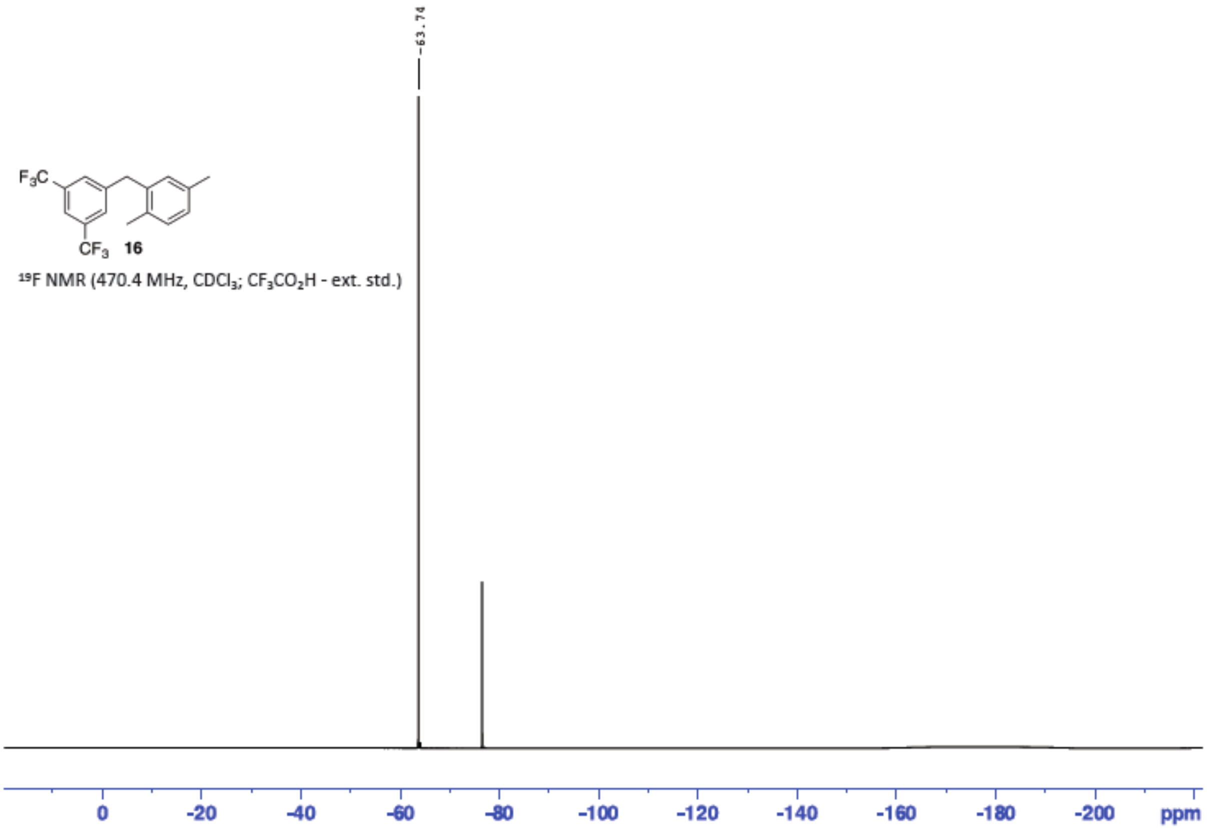

^{19}F NMR (470.4 MHz, $CDCl_3$; CF_3CO_2H - ext. std.)

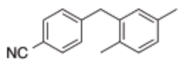

17

^{1}H NMR (500 MHz, $CDCl_3$)

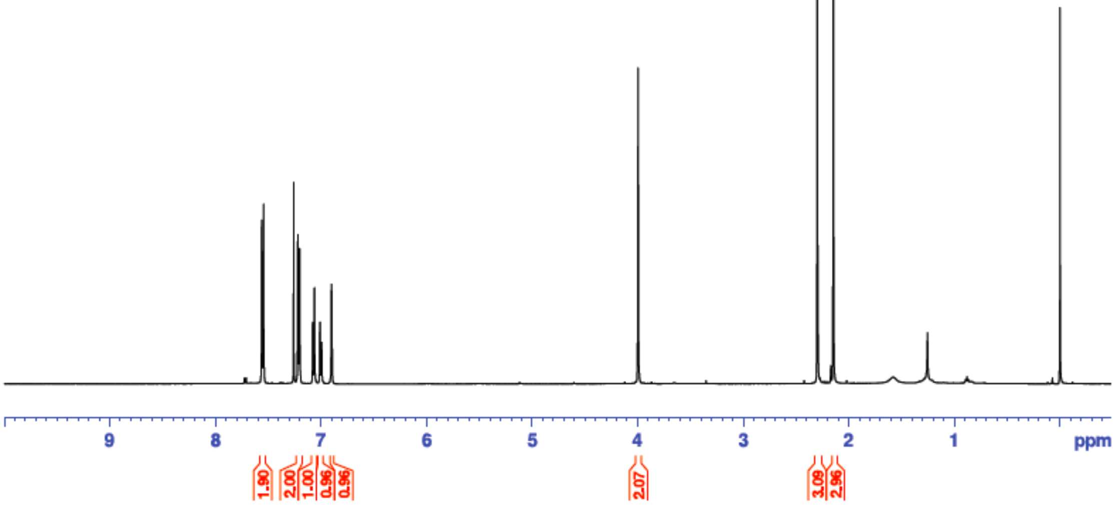

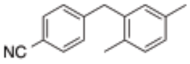

17

^{13}C NMR (125 MHz, $CDCl_3$)

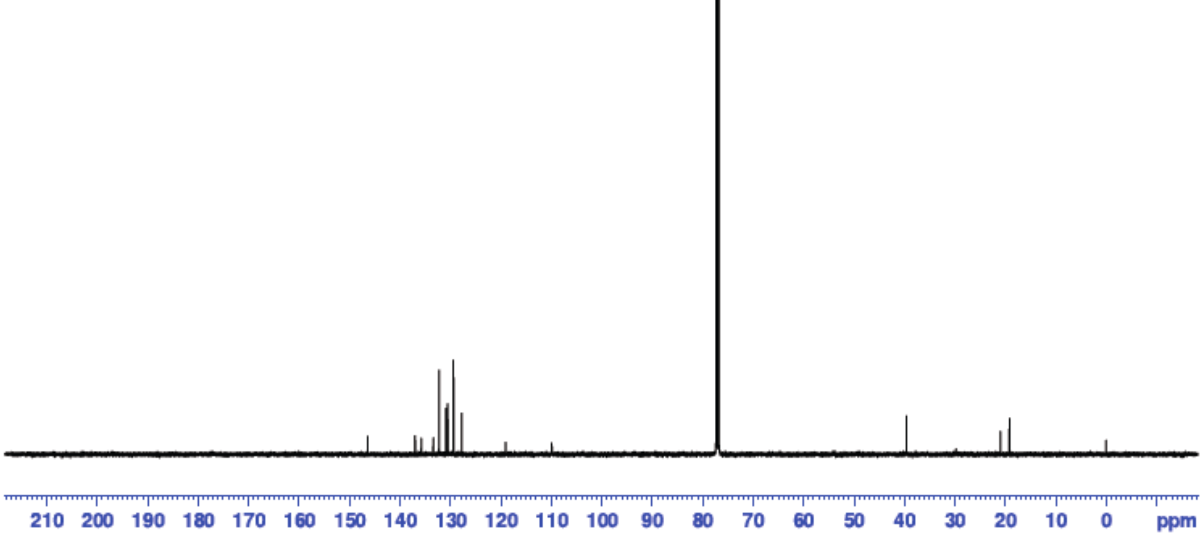

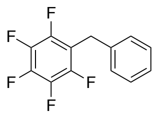

19

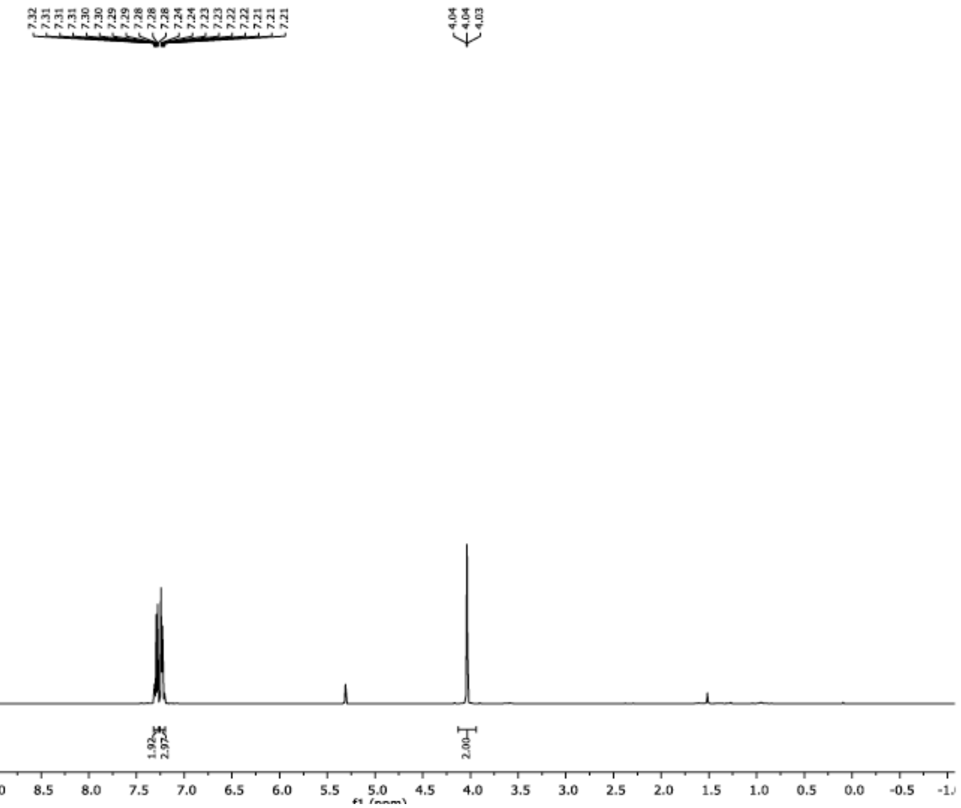
7.32 7.31 7.31 7.31 7.30 7.30 7.29 7.29 7.28 7.28 7.28 7.24 7.24 7.23 7.23 7.22 7.22 7.21 7.21 7.21
4.04 4.04 4.03
1.92
2.97
2.00
f1 (ppm)

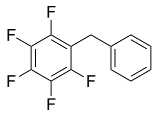

19

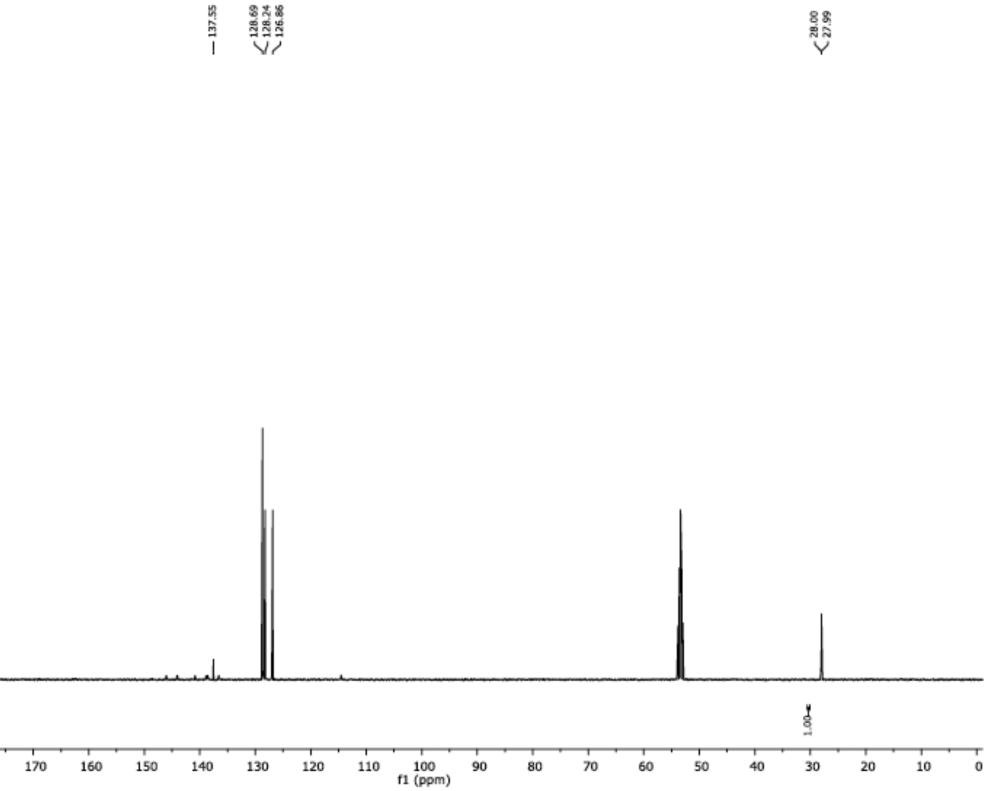
137.55
128.69 128.24 126.86
28.00 27.99
1.00
f1 (ppm)

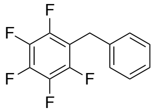

19

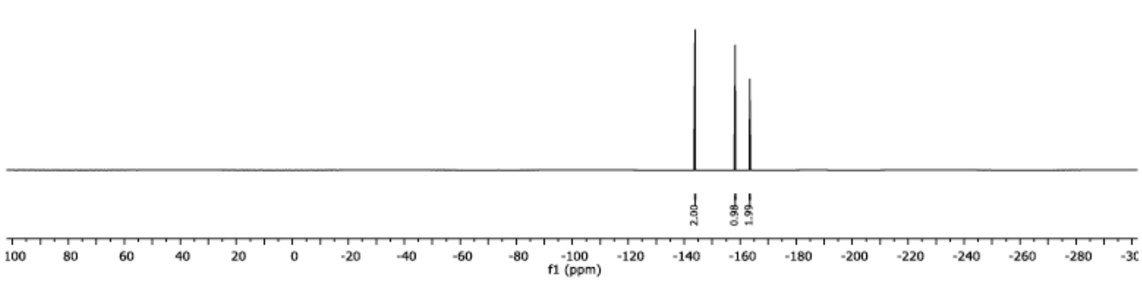

-143.85
-143.87
-143.90
-143.92
-158.19
-158.23
-158.28
-163.37
-163.38
-163.41
-163.43
-163.46
-163.47
2.00
0.98
1.99
100 80 60 40 20 0 -20 -40 -60 -80 -100 -120 -140 -160 -180 -200 -220 -240 -260 -280 -300
f1 (ppm)

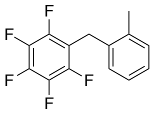

20
mixture of isomers

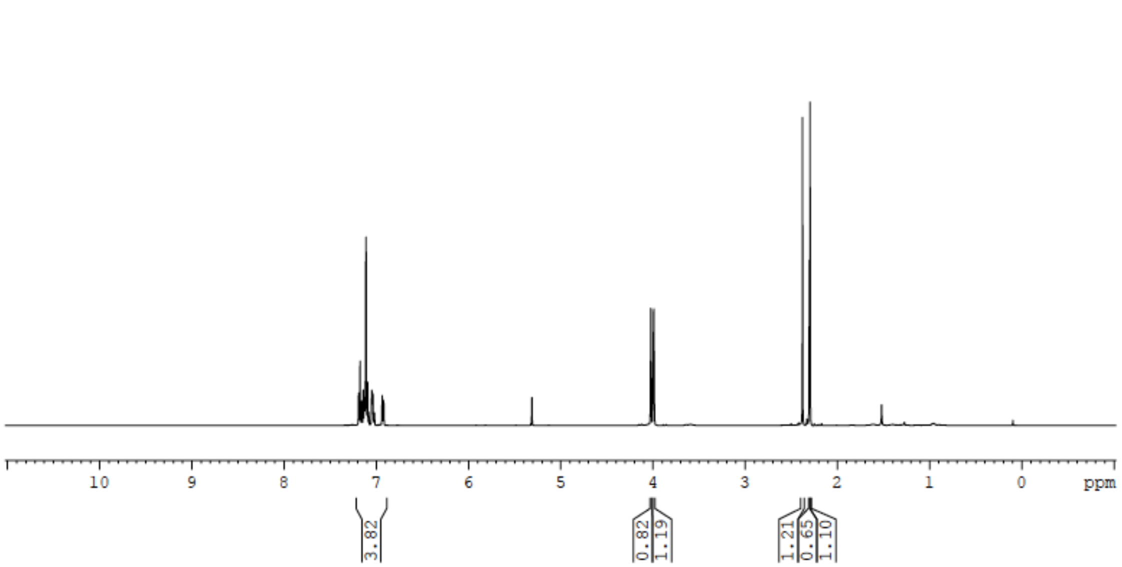

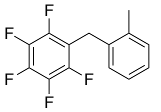

20
mixture of isomers

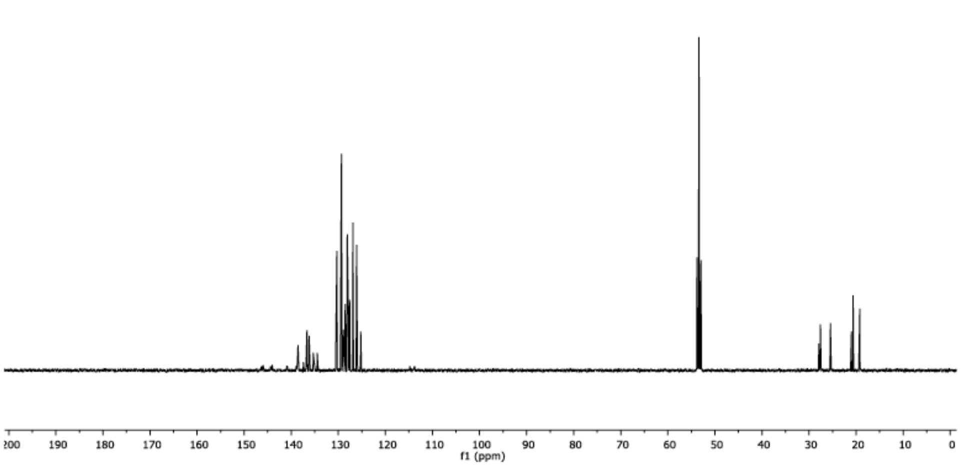

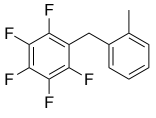

20
mixture of isomers

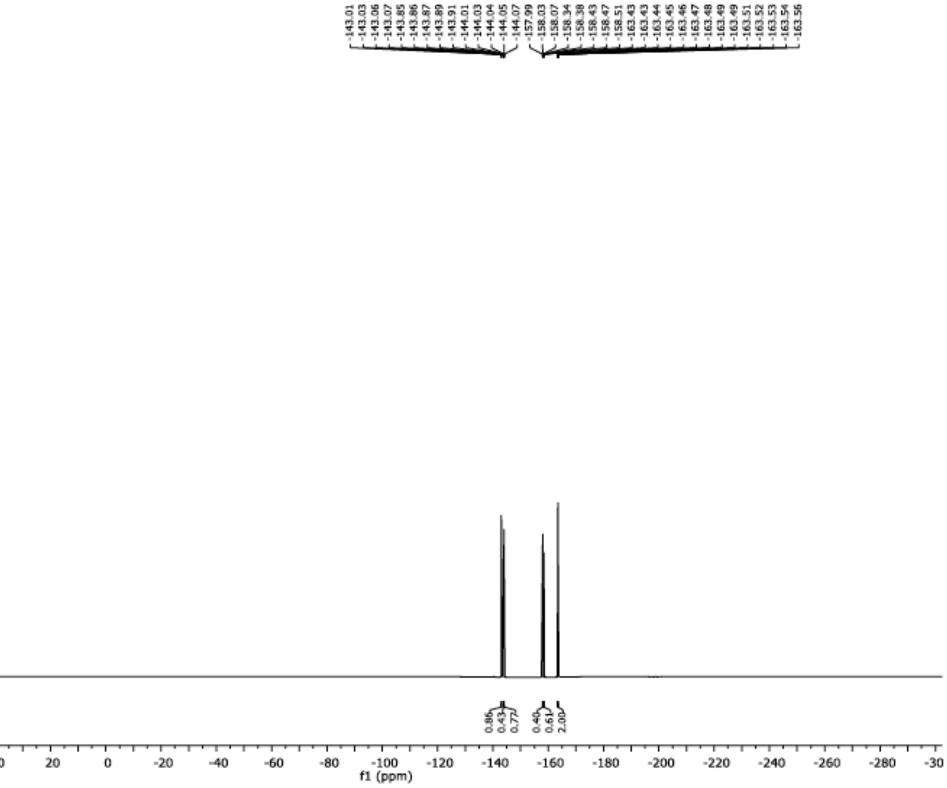

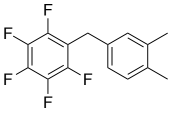

21
mixture of isomers

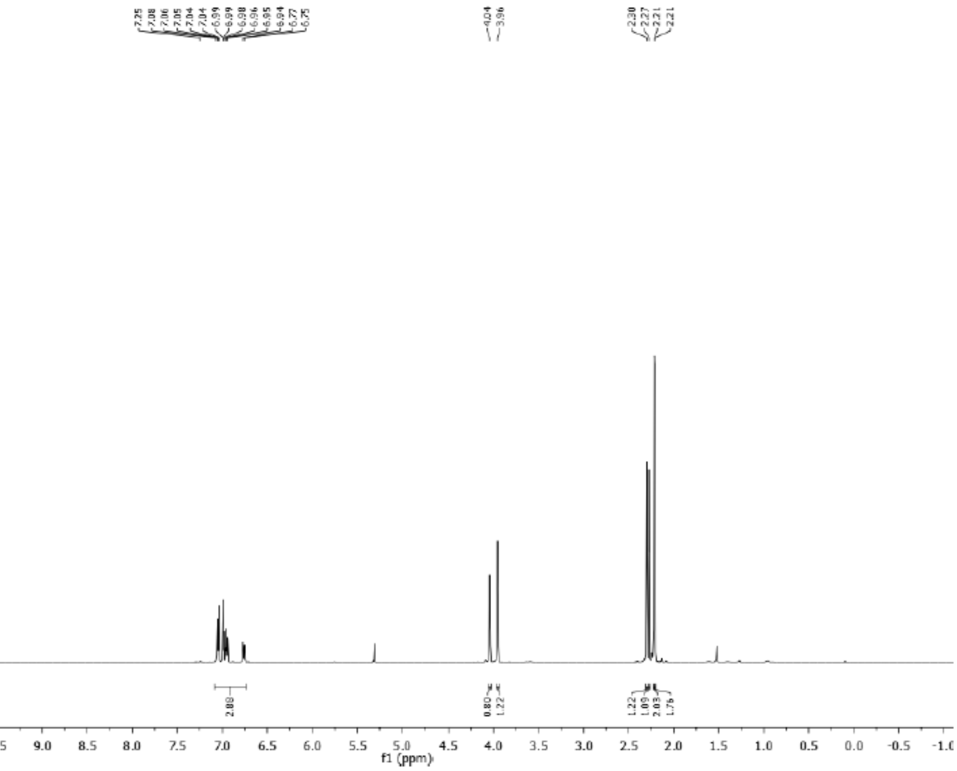

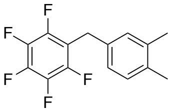

21
mixture of isomers

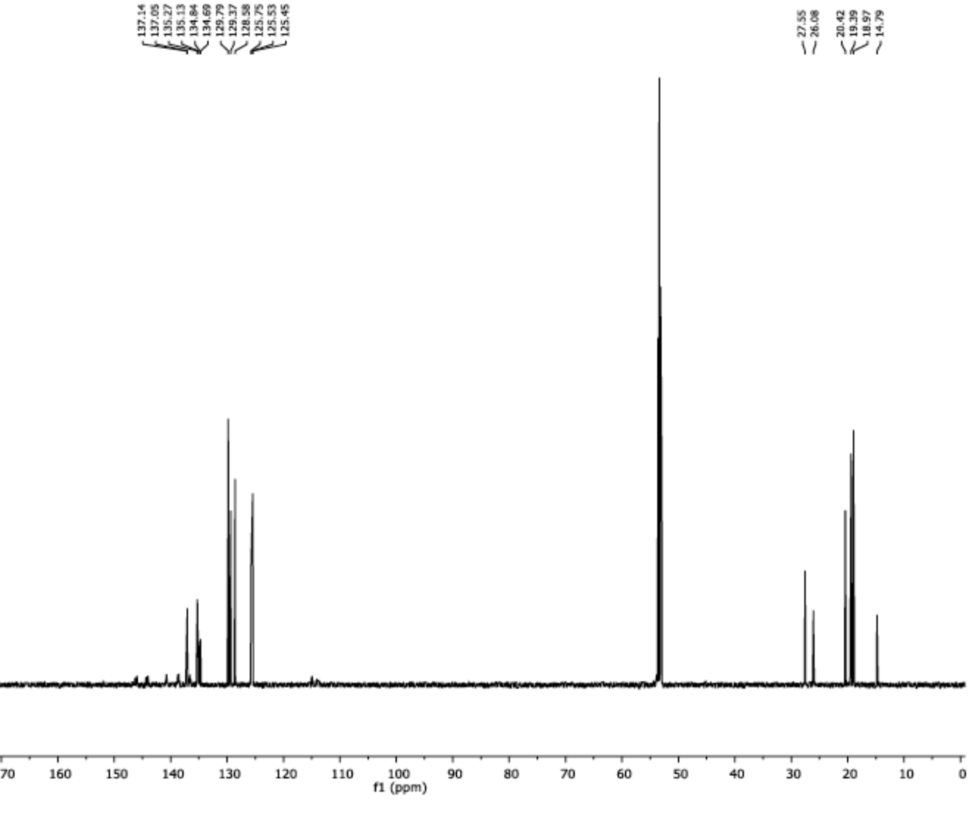

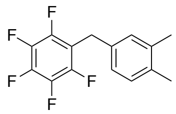

21
mixture of isomers

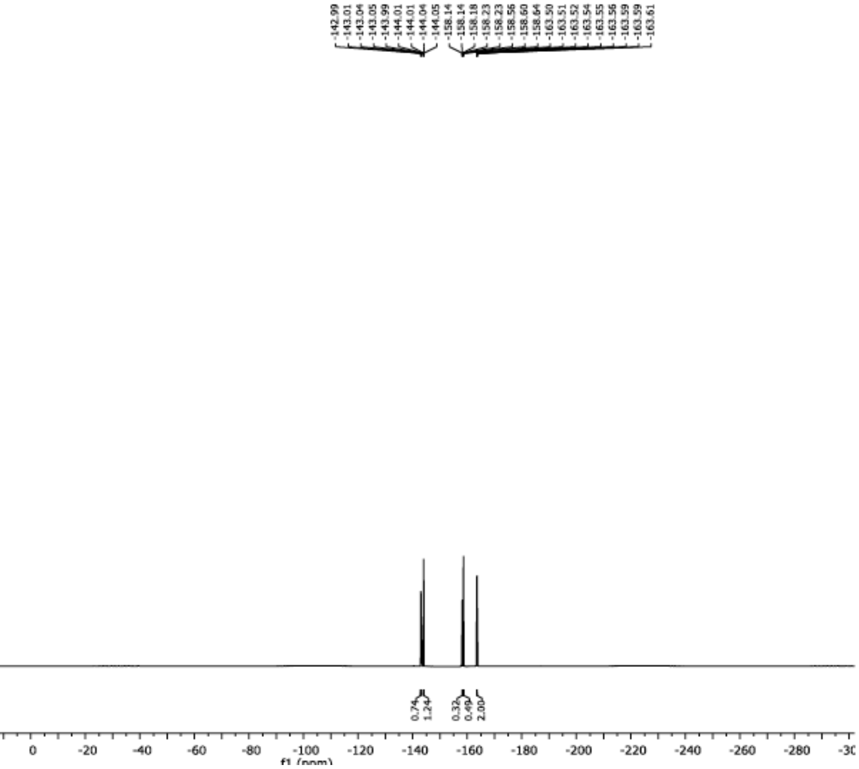

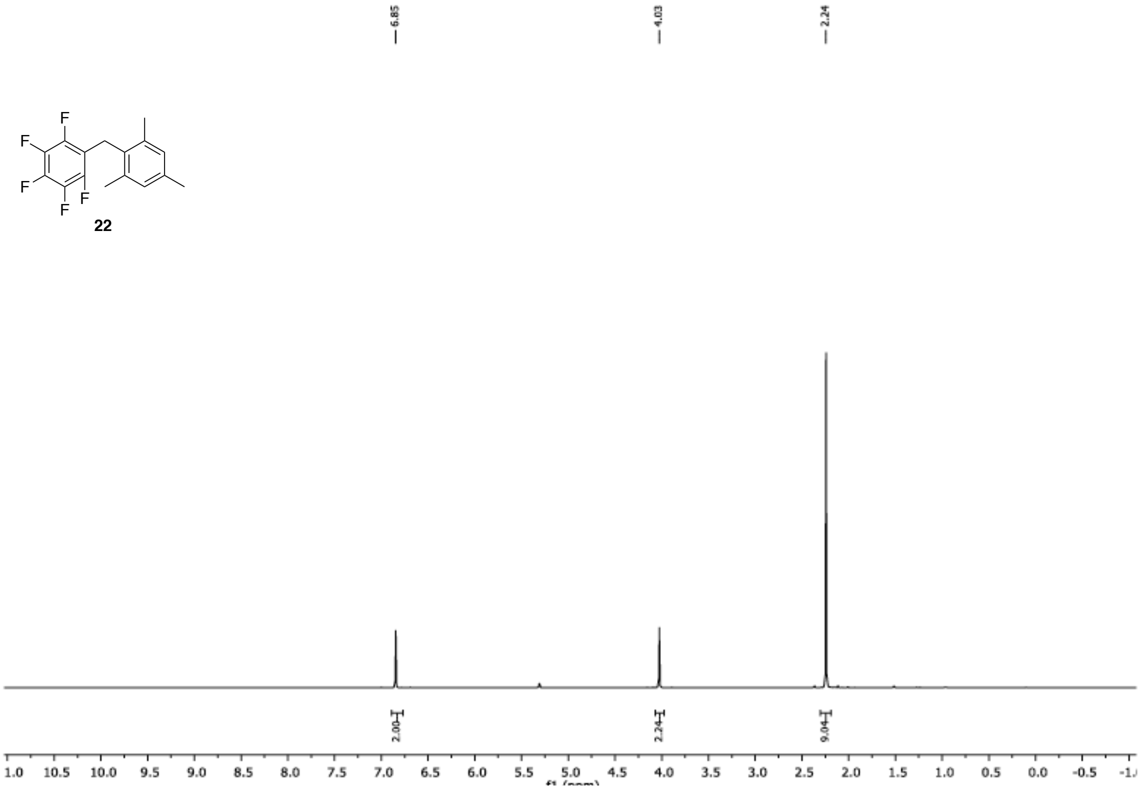

6.85
4.03
2.24
22
2.00
2.24
9.04
1.0 10.5 10.0 9.5 9.0 8.5 8.0 7.5 7.0 6.5 6.0 5.5 5.0 4.5 4.0 3.5 3.0 2.5 2.0 1.5 1.0 0.5 0.0 -0.5

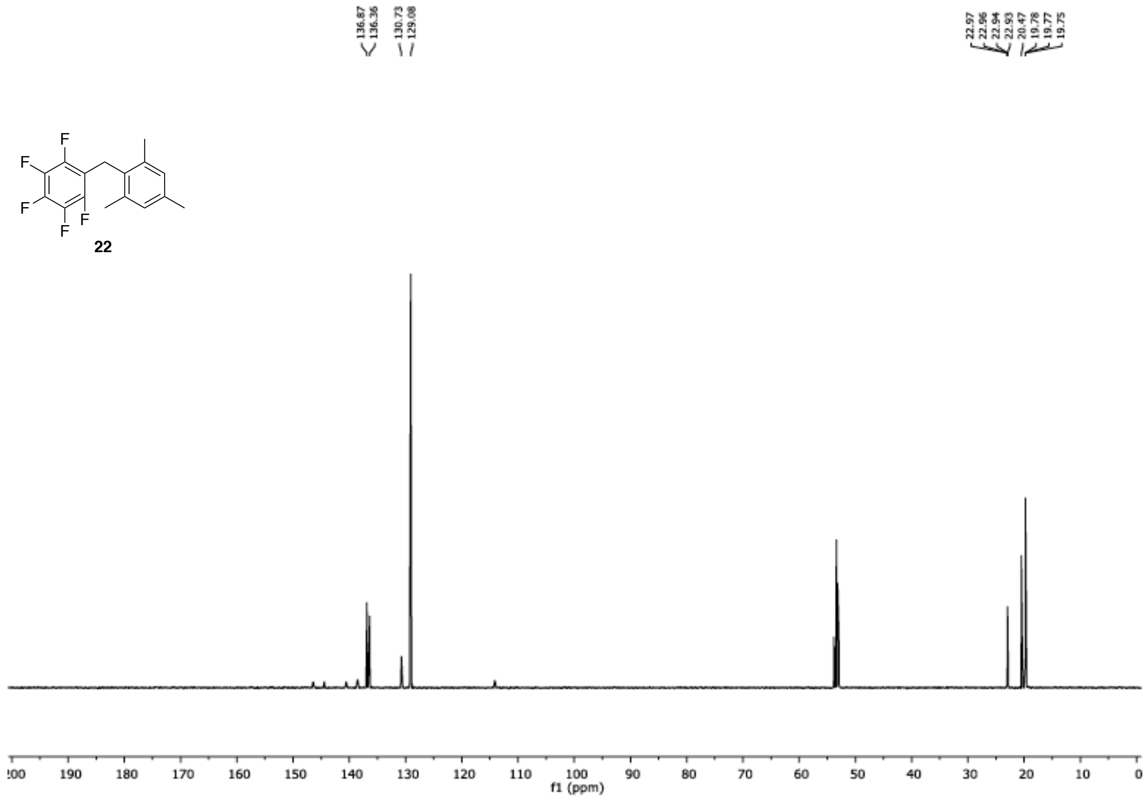

136.87
136.36
130.73
129.08
22.97
22.96
22.94
22.93
20.47
19.78
19.77
19.75
22
190 180 170 160 150 140 130 120 110 100 90 80 70 60 50 40 30 20 10 0
f1 (ppm)

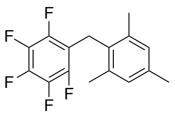

22

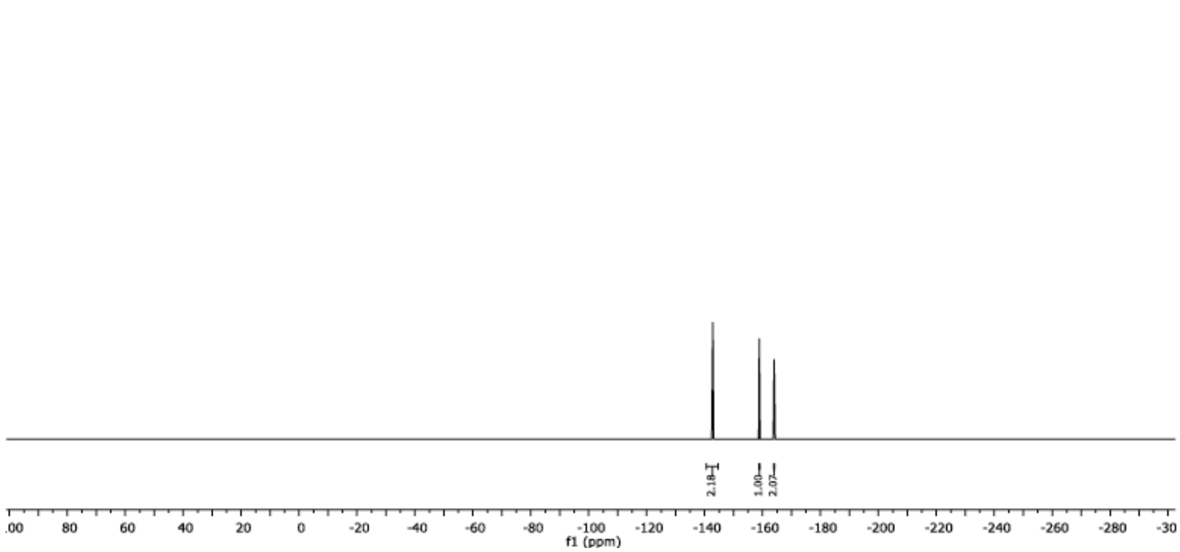

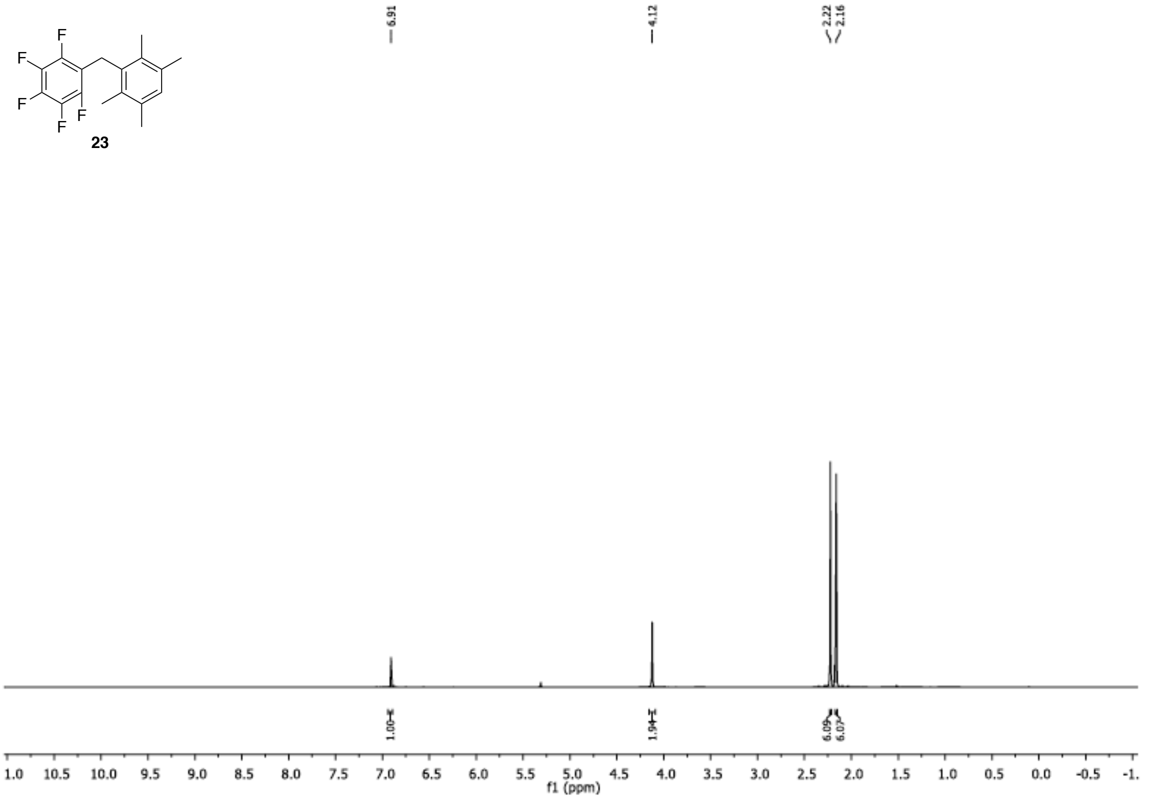
23
6.91
4.12
2.22
2.16
1.00
1.94
6.09
6.07
f1 (ppm)

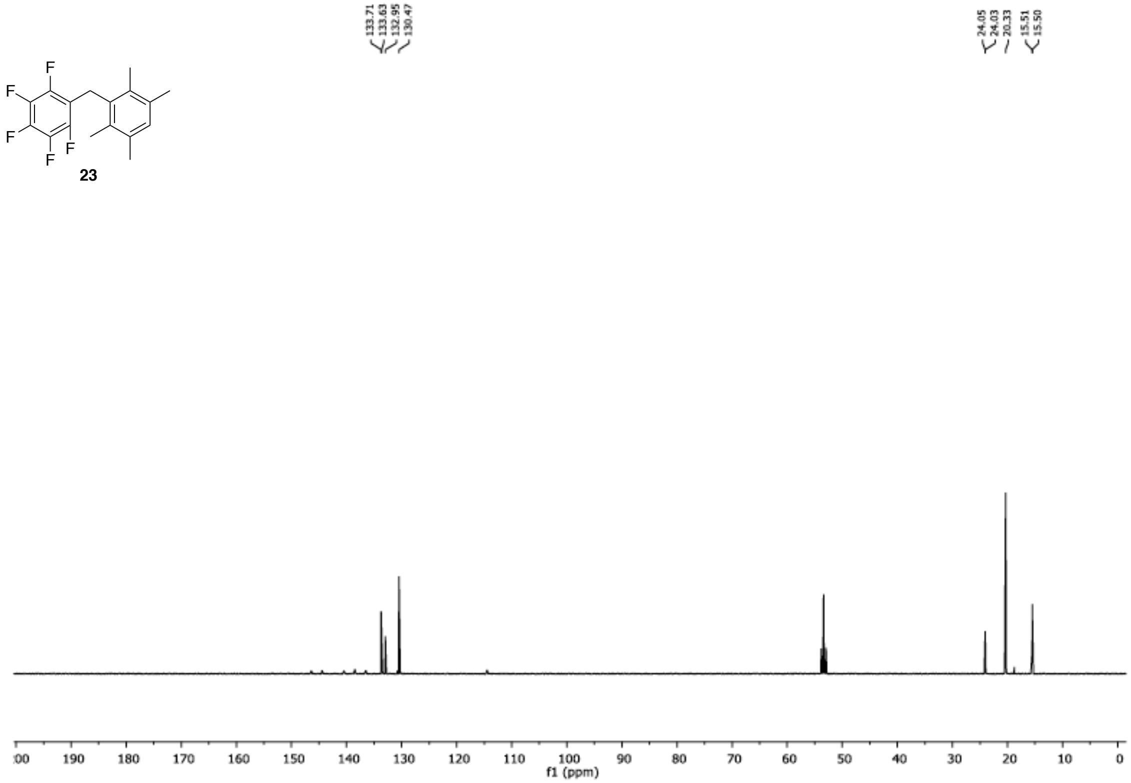
23
133.71
133.63
132.95
130.47
24.05
24.03
20.33
15.51
15.50
f1 (ppm)

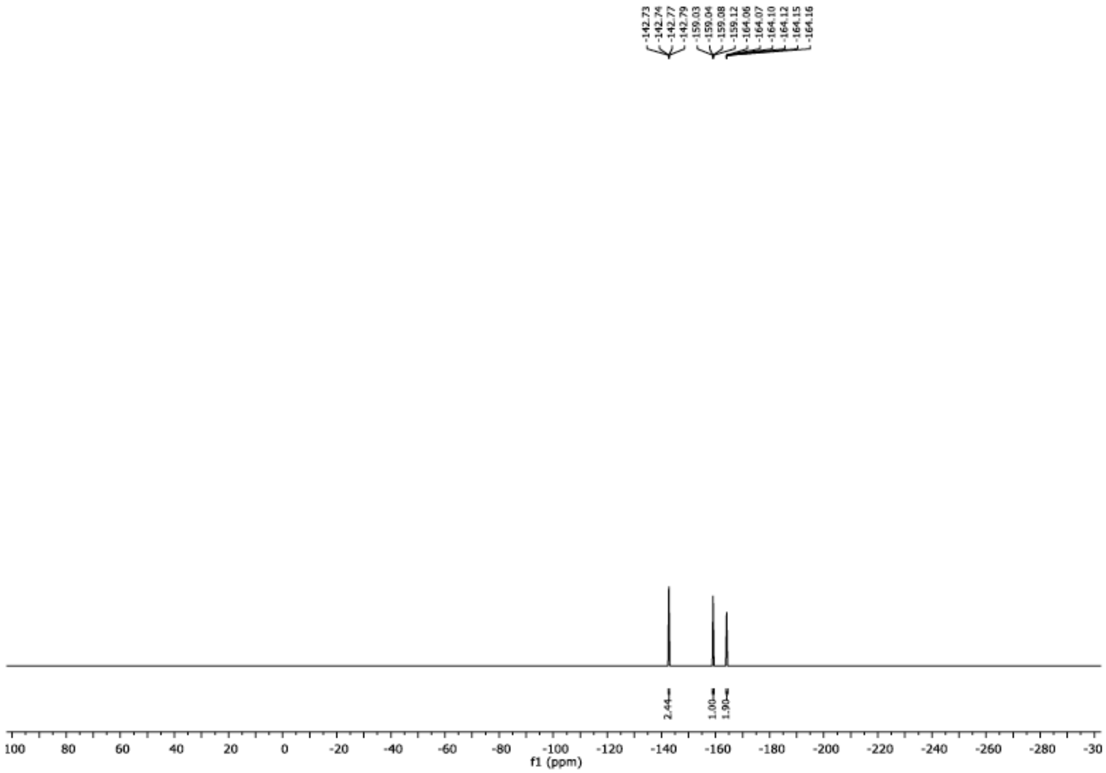
-142.73
-142.74
-142.77
-142.79
-159.03
-159.04
-159.08
-159.12
-164.06
-164.07
-164.10
-164.12
-164.15
-164.16
2.44
1.00
1.90
100 80 60 40 20 0 -20 -40 -60 -80 -100 -120 -140 -160 -180 -200 -220 -240 -260 -280 -30
f1 (ppm)

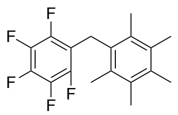

24

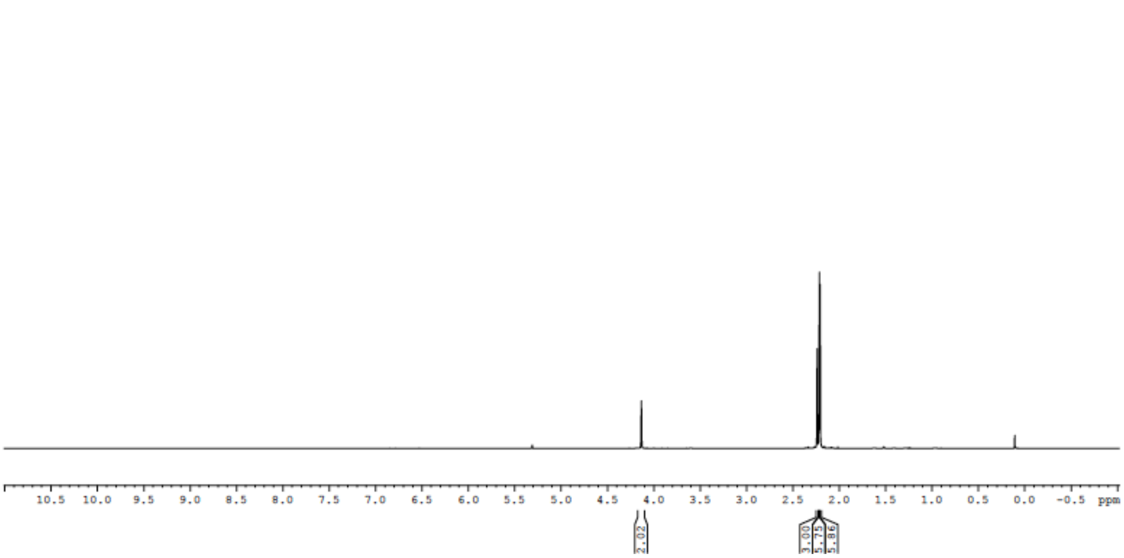

4.13
2.24
2.21
2.21
ppm
2.02
3.00
5.75
5.86

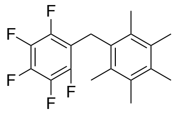

24

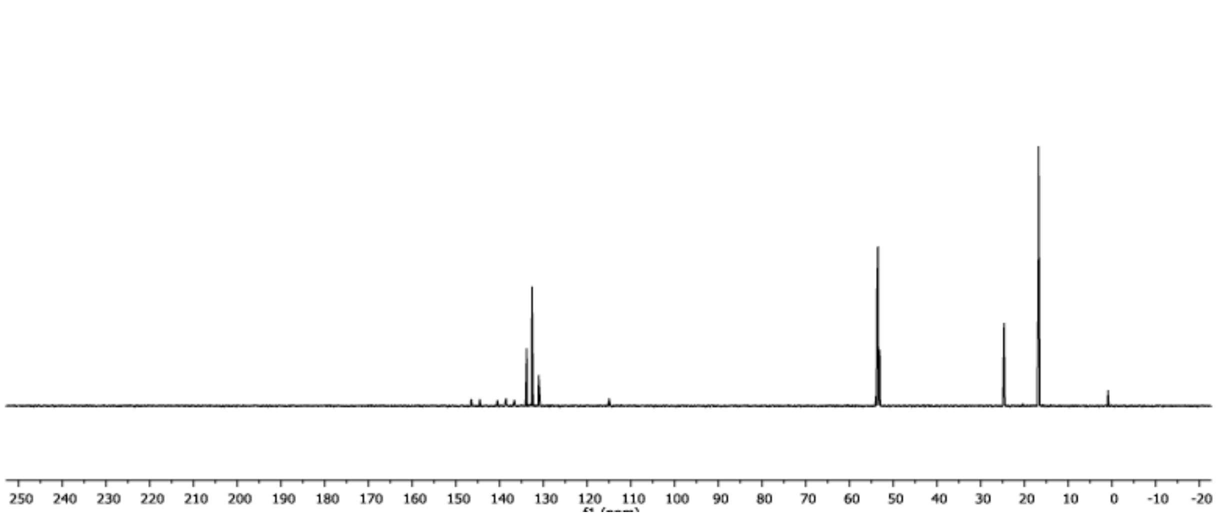

133.82
132.62
132.59
131.10
24.67
24.66
16.83
16.76
16.67
0.90

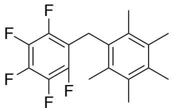

24

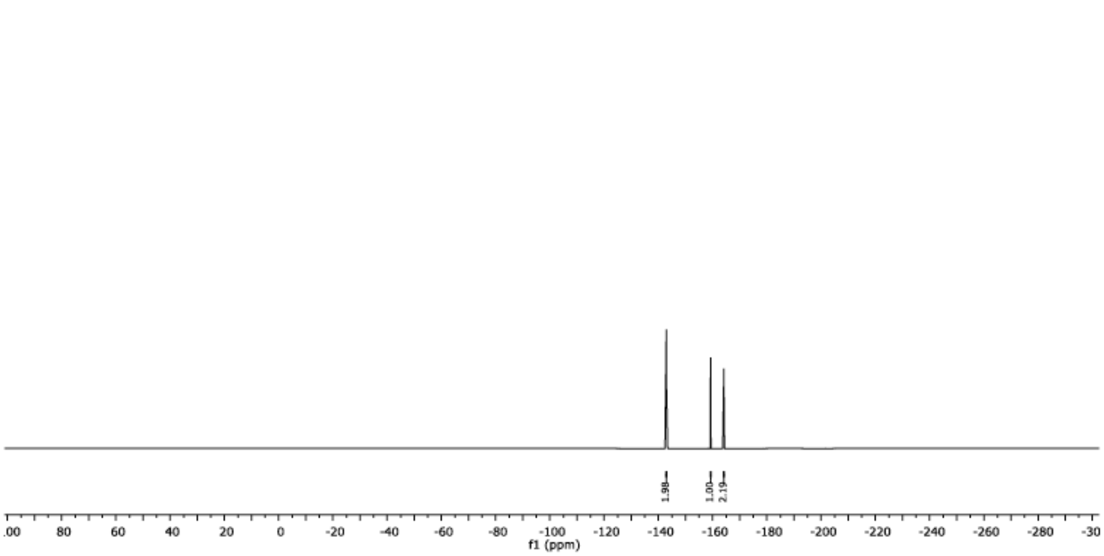

-142.89
-142.90
-142.93
-142.95
-159.20
-159.21
-159.22
-159.25
-159.26
-159.30
-164.11
-164.12
-164.15
-164.17
-164.20
-164.21
1.98
1.00
2.19
f1 (ppm)

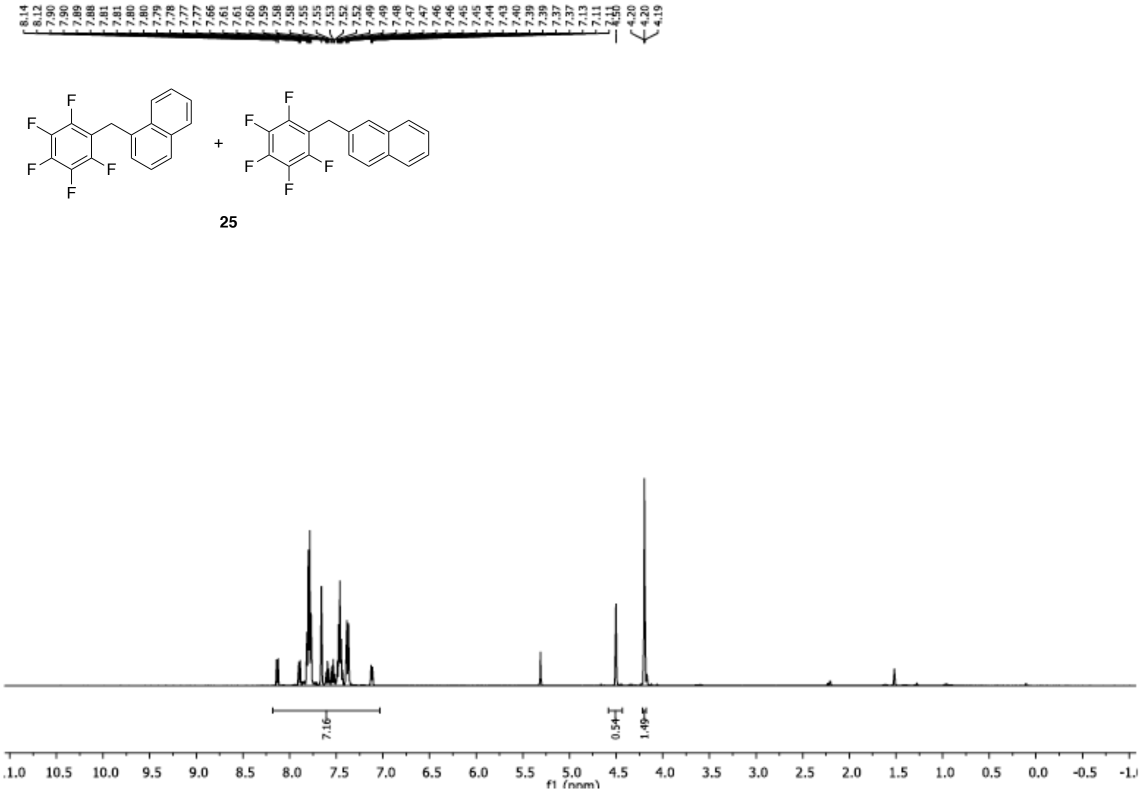

25

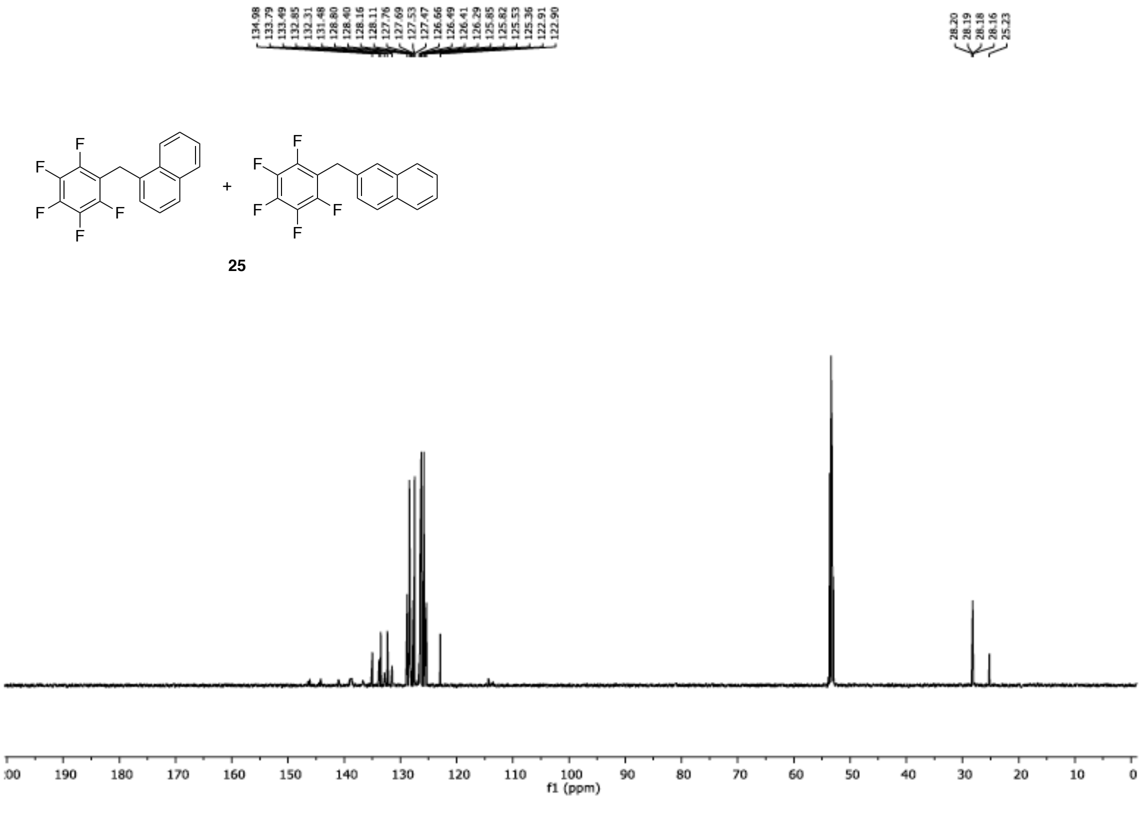

25

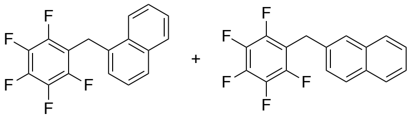

25

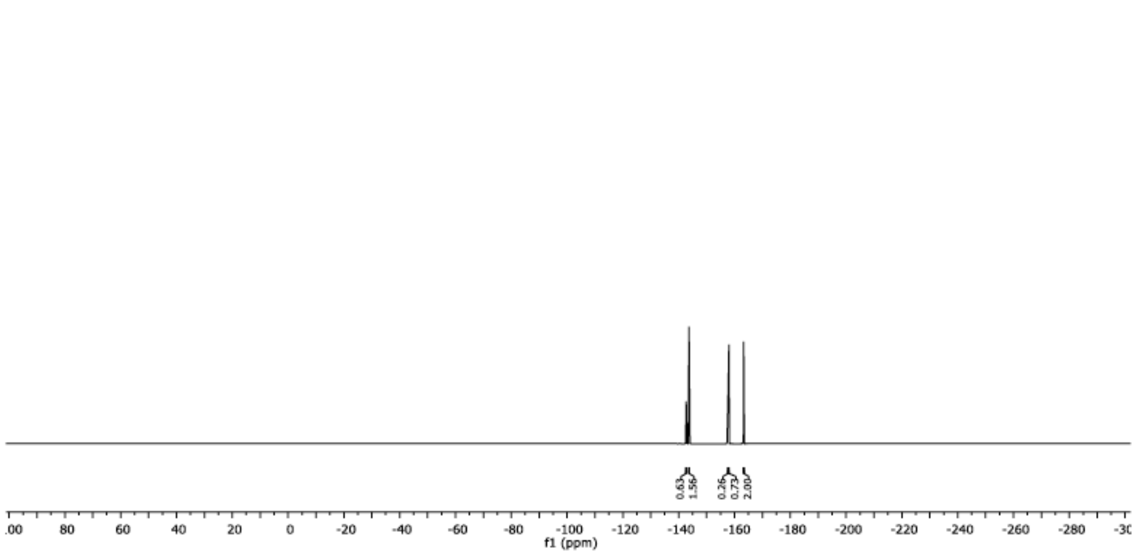

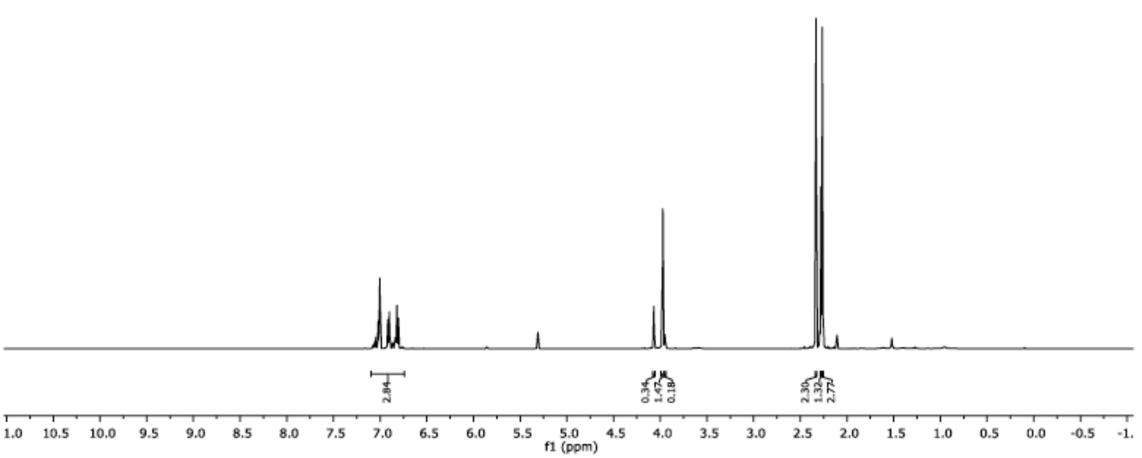

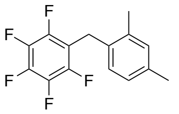

26
mixture of isomers

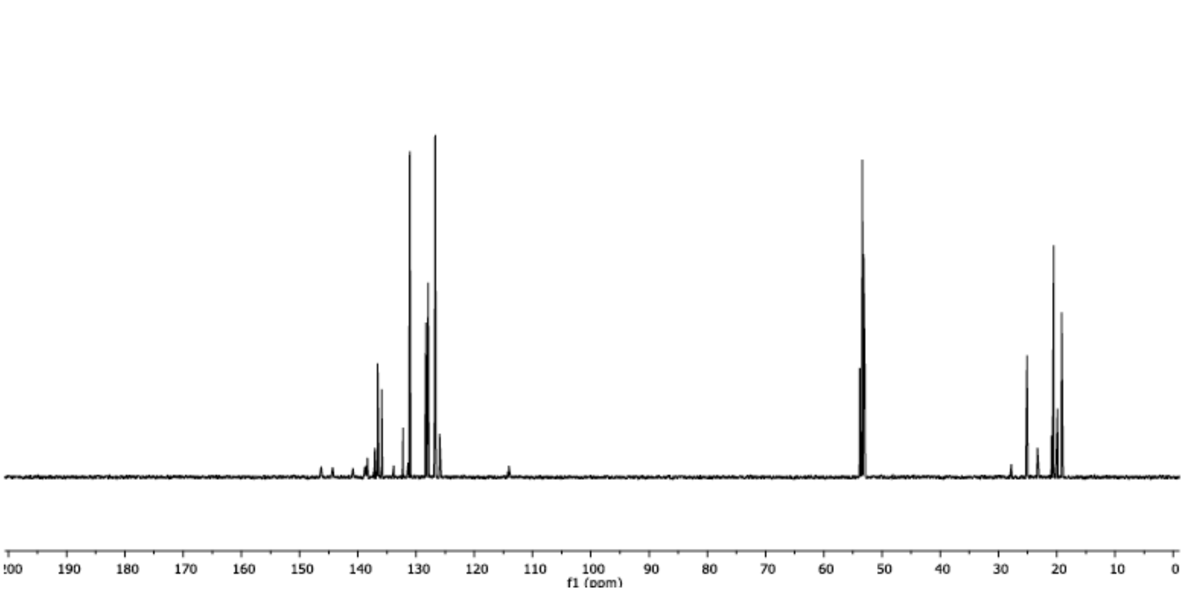

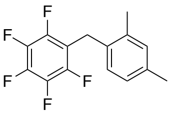

26
mixture of isomers

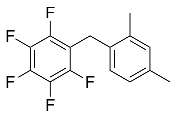

26
mixture of isomers

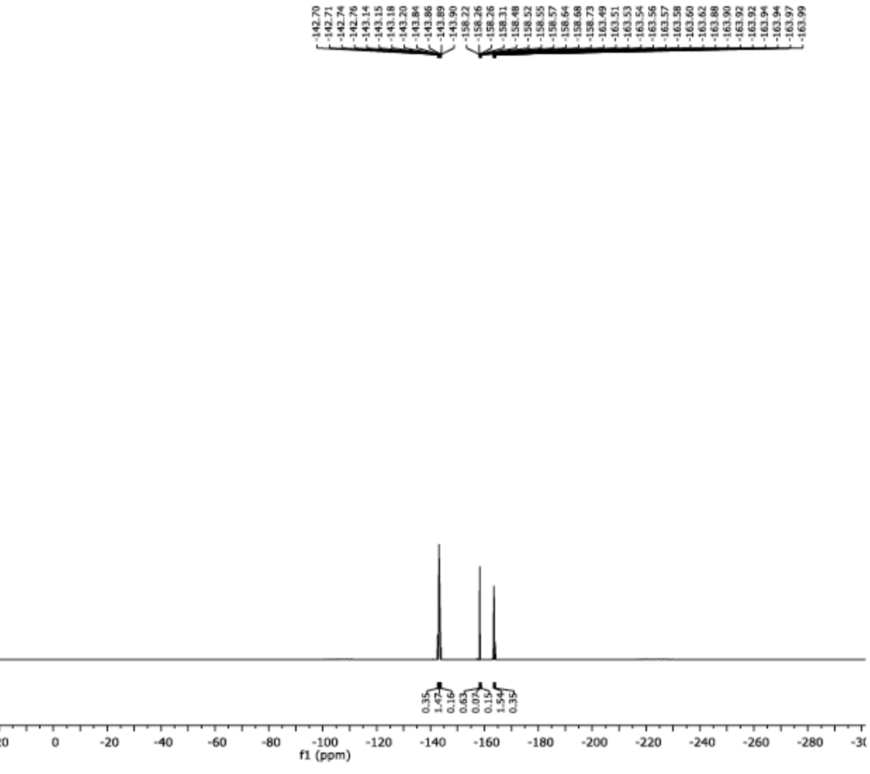

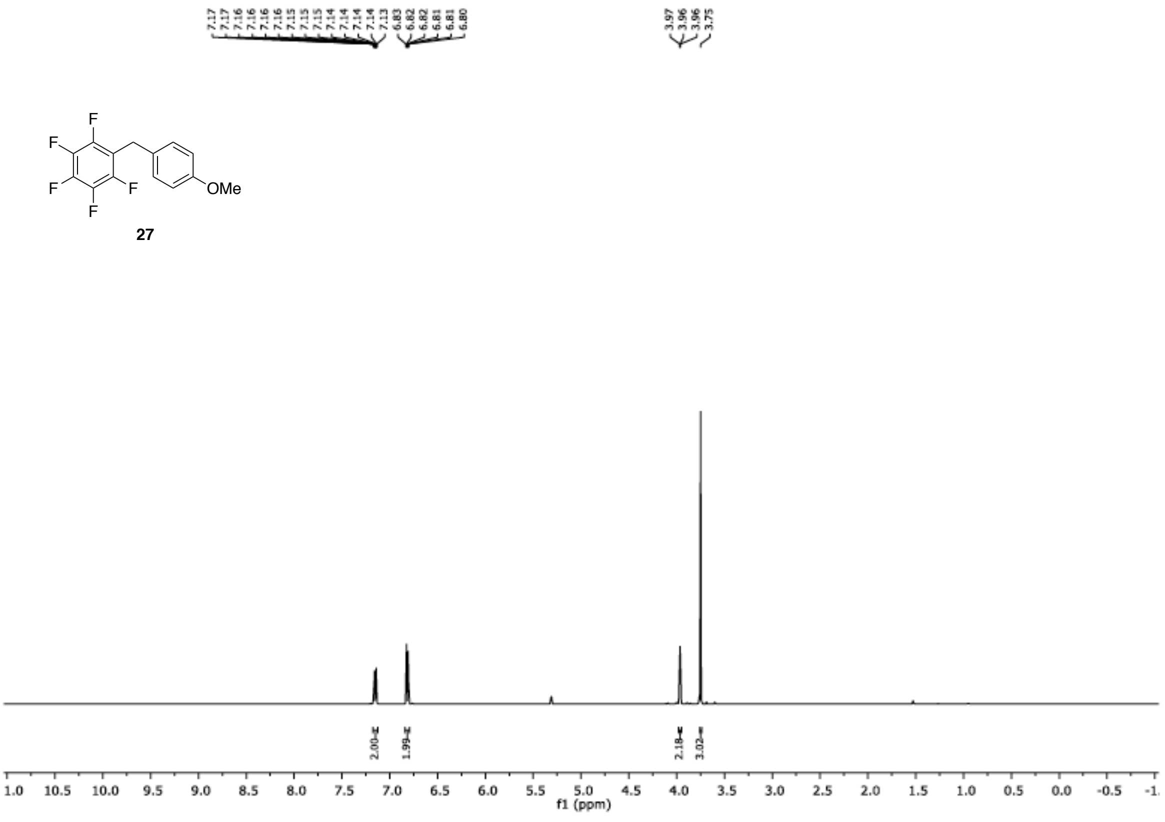
F
F
F
F
F
OMe
27
7.17 7.17 7.16 7.16 7.16 7.15 7.15 7.15 7.14 7.14 7.14 7.14 7.13 6.83 6.82 6.82 6.82 6.81 6.81 6.80
3.97 3.96 3.96 3.75
2.00 1.99 2.18 3.02
f1 (ppm)

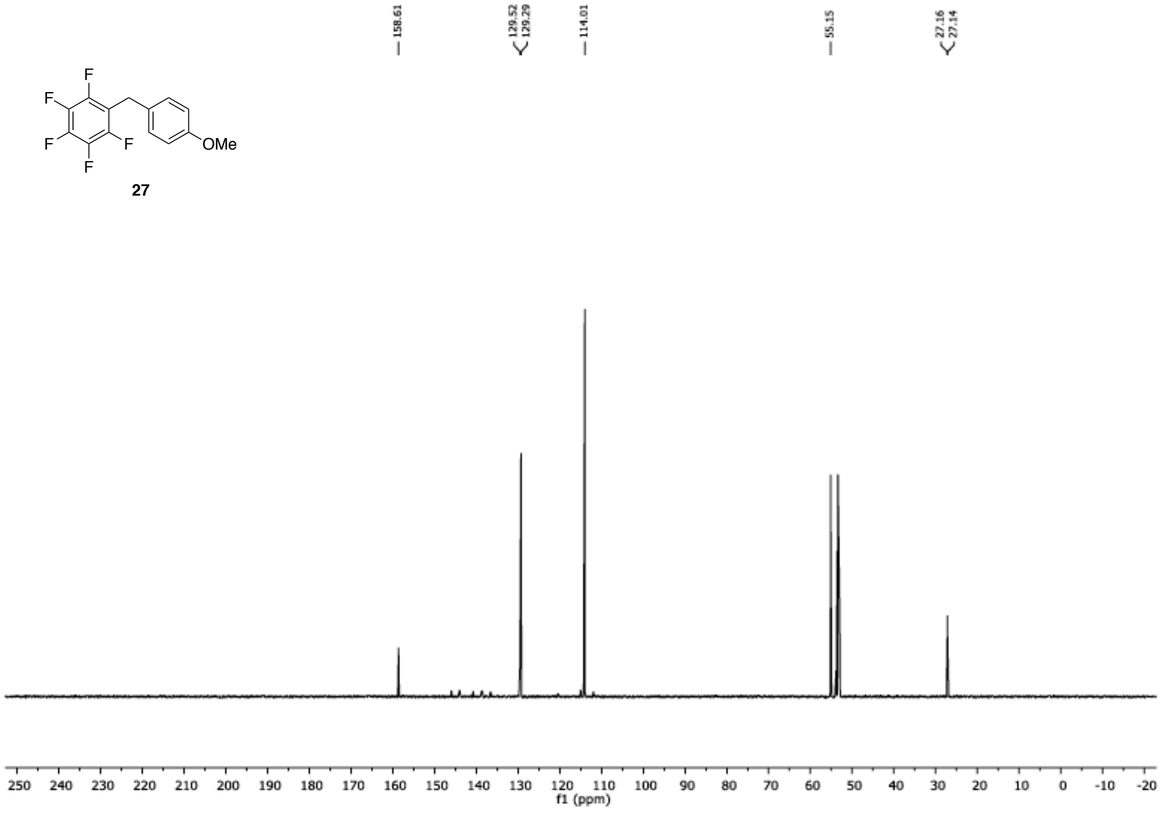
F
F
F
F
F
OMe
27
158.61
129.52 129.29
114.01
55.15
27.16 27.14
f1 (ppm)

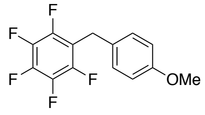
F
F
F
F
F
OMe

27

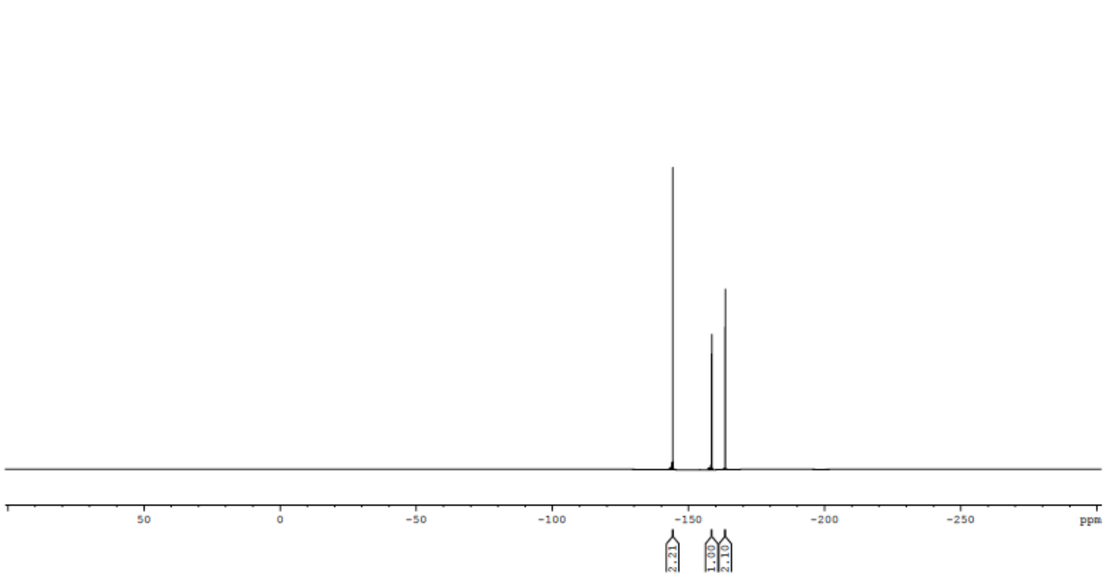
-144.19
-144.21
-144.24
-144.26
-158.49
-158.53
-158.57
-163.43
-163.45
-163.48
-163.49
-163.52
-163.54
50
0
-50
-100
-150
-200
-250
ppm
2.21
1.00
2.10

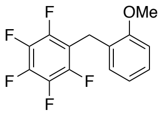
F
F
F
F
F
OMe

27

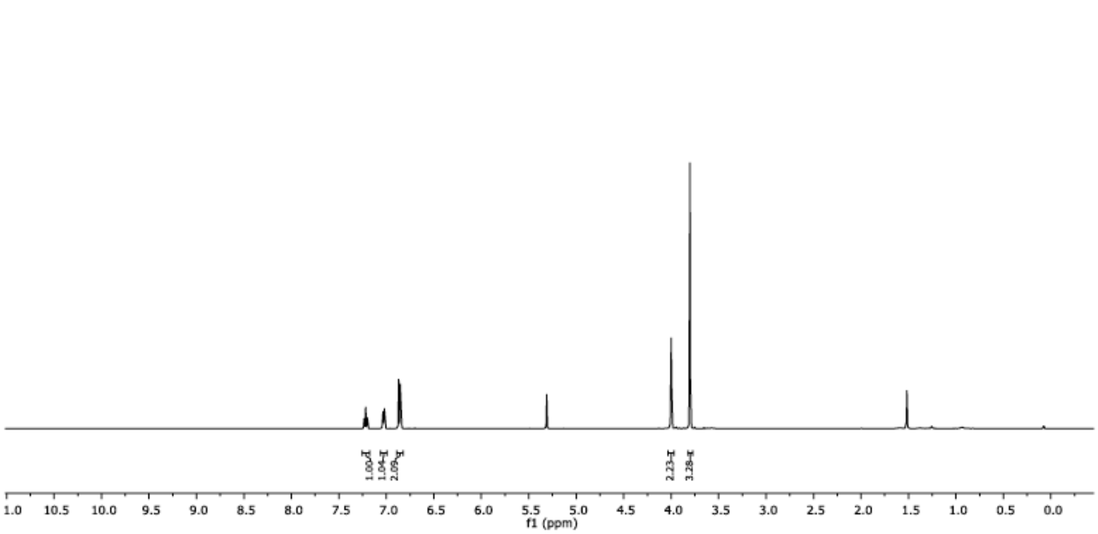
7.23
7.23
7.22
7.21
7.20
7.20
7.03
7.02
6.87
6.85
6.84
4.00
3.80
1.00
1.04
2.09
2.23
3.28
1.0
10.5
10.0
9.5
9.0
8.5
8.0
7.5
7.0
6.5
6.0
5.5
5.0
4.5
4.0
3.5
3.0
2.5
2.0
1.5
1.0
0.5
0.0
f1 (ppm)

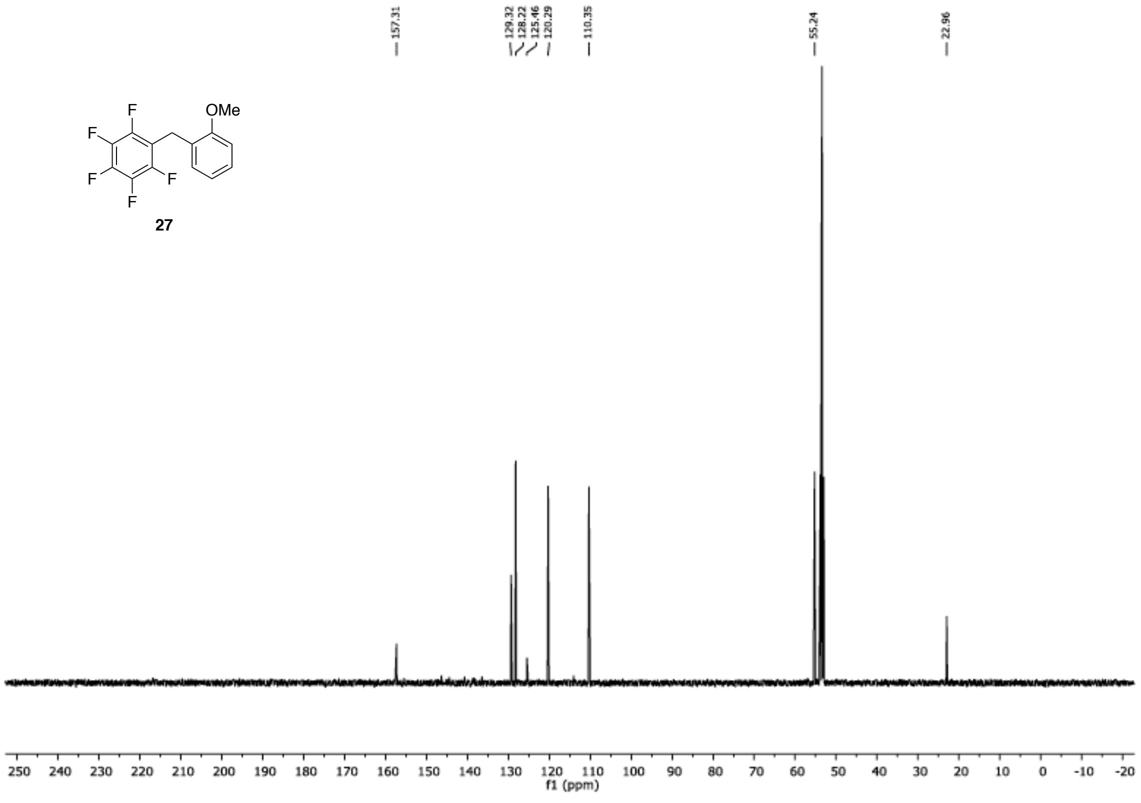
157.31
129.32
128.22
125.46
120.29
110.35
55.24
22.96
OMe
F
27
f1 (ppm)

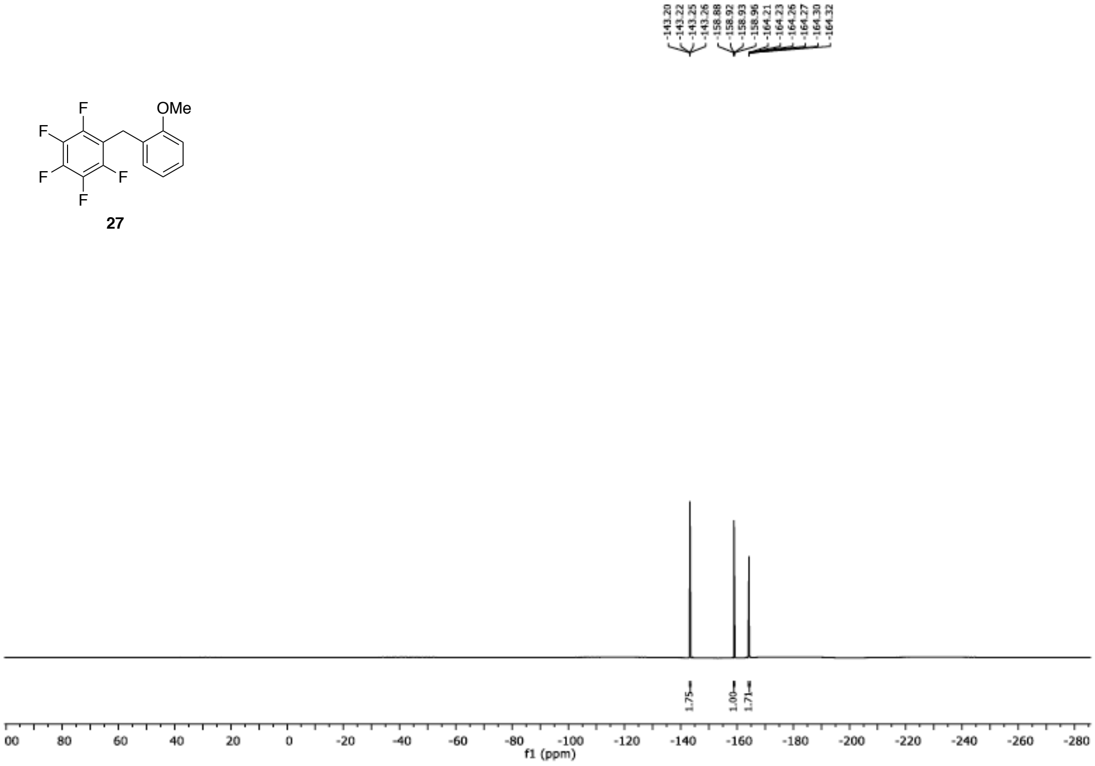
-143.20
-143.22
-143.25
-143.26
-158.88
-158.92
-158.93
-158.96
-164.21
-164.23
-164.26
-164.27
-164.30
-164.32
OMe
F
27
1.75
1.00
1.71
f1 (ppm)

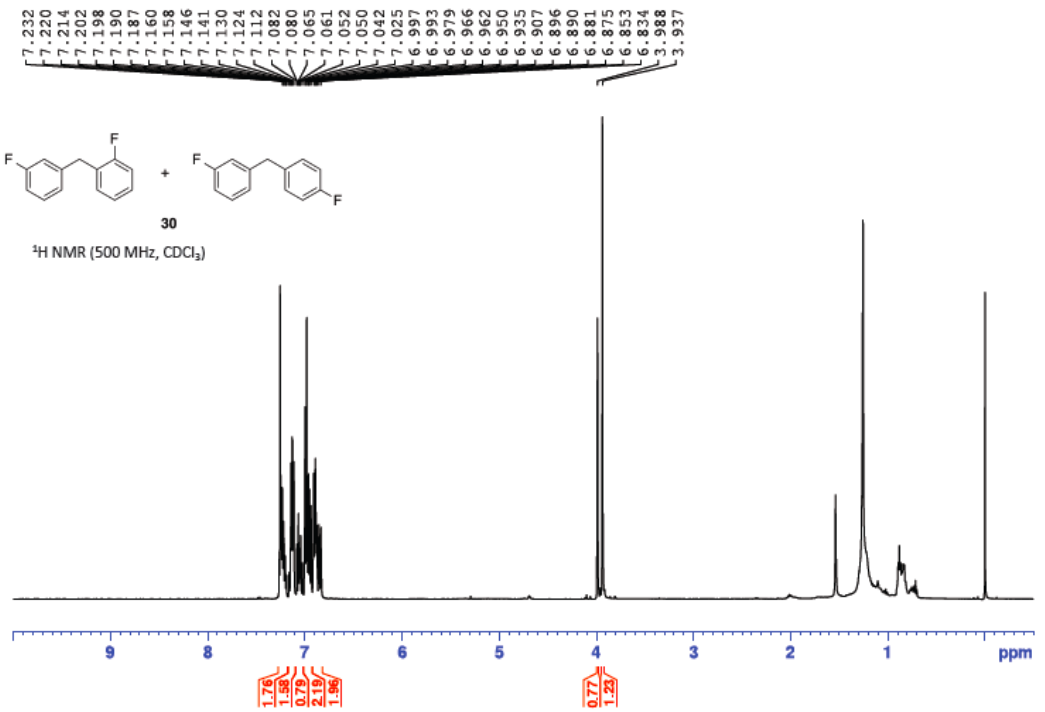
30
¹H NMR (500 MHz, CDCl₃)
ppm

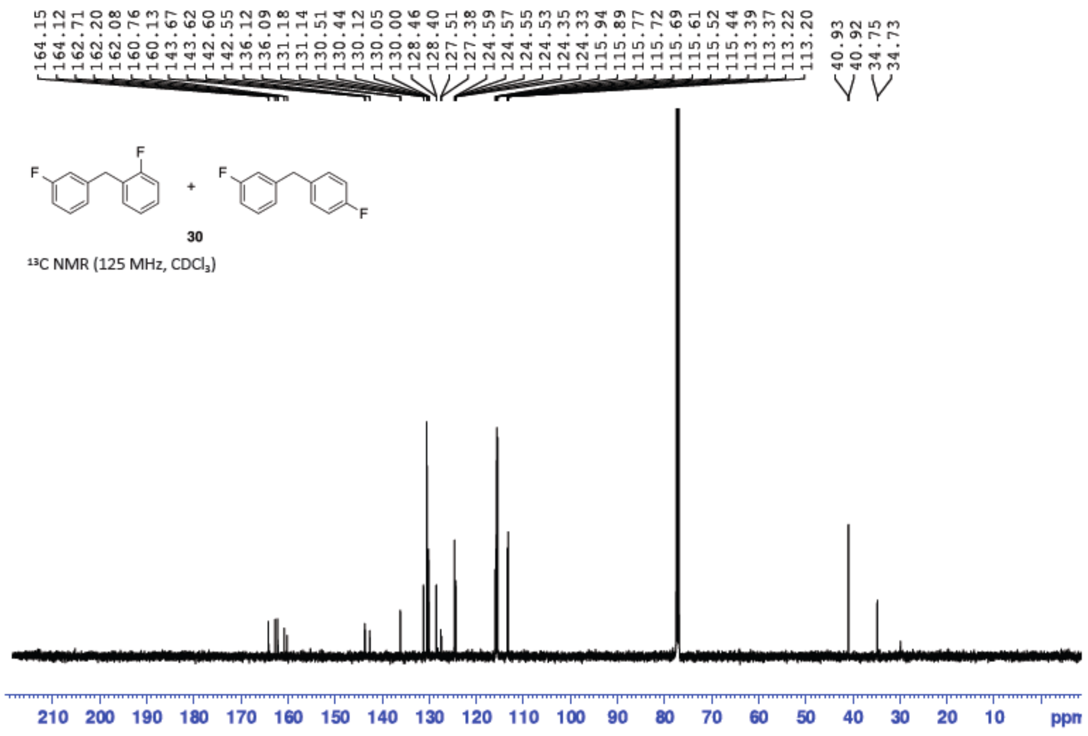
30
¹³C NMR (125 MHz, CDCl₃)
ppm

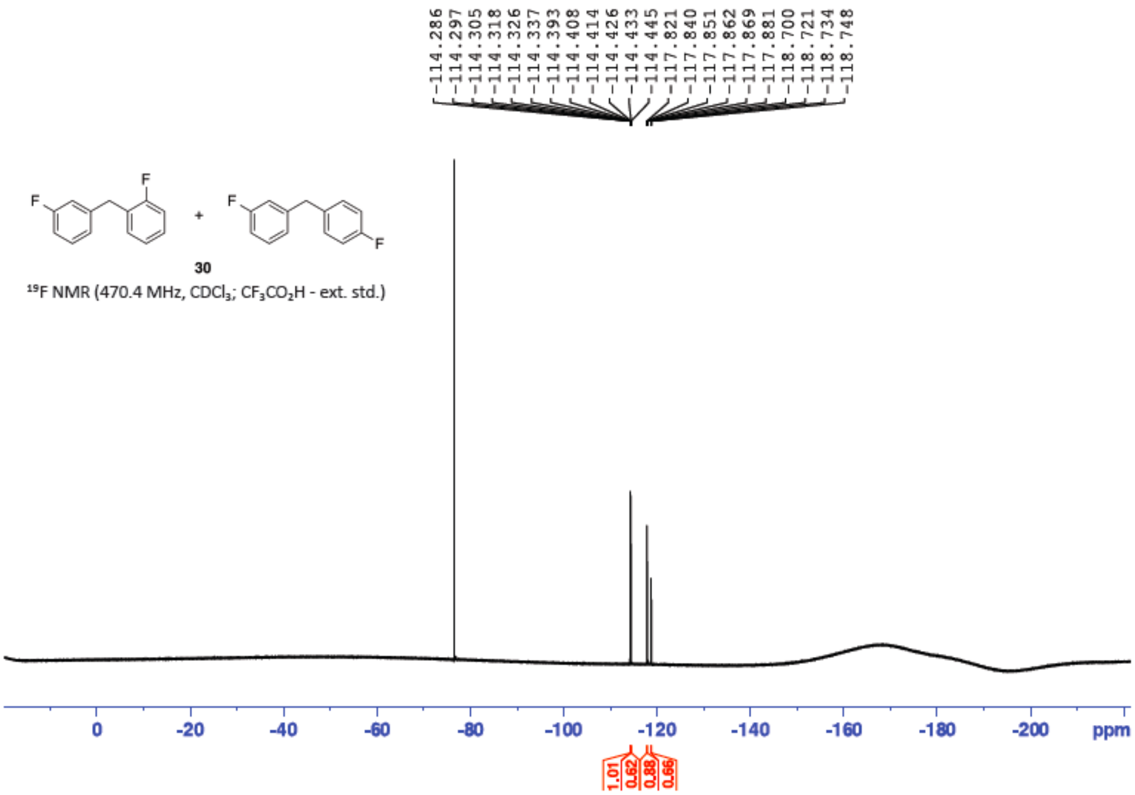
30
19F NMR (470.4 MHz, CDCl3; CF3CO2H - ext. std.)
-114.286
-114.297
-114.305
-114.318
-114.326
-114.337
-114.393
-114.408
-114.414
-114.426
-114.433
-114.445
-117.821
-117.840
-117.851
-117.862
-117.869
-117.881
-118.700
-118.721
-118.734
-118.748
0
-20
-40
-60
-80
-100
-120
-140
-160
-180
-200
ppm
1.01
0.62
0.88
0.66

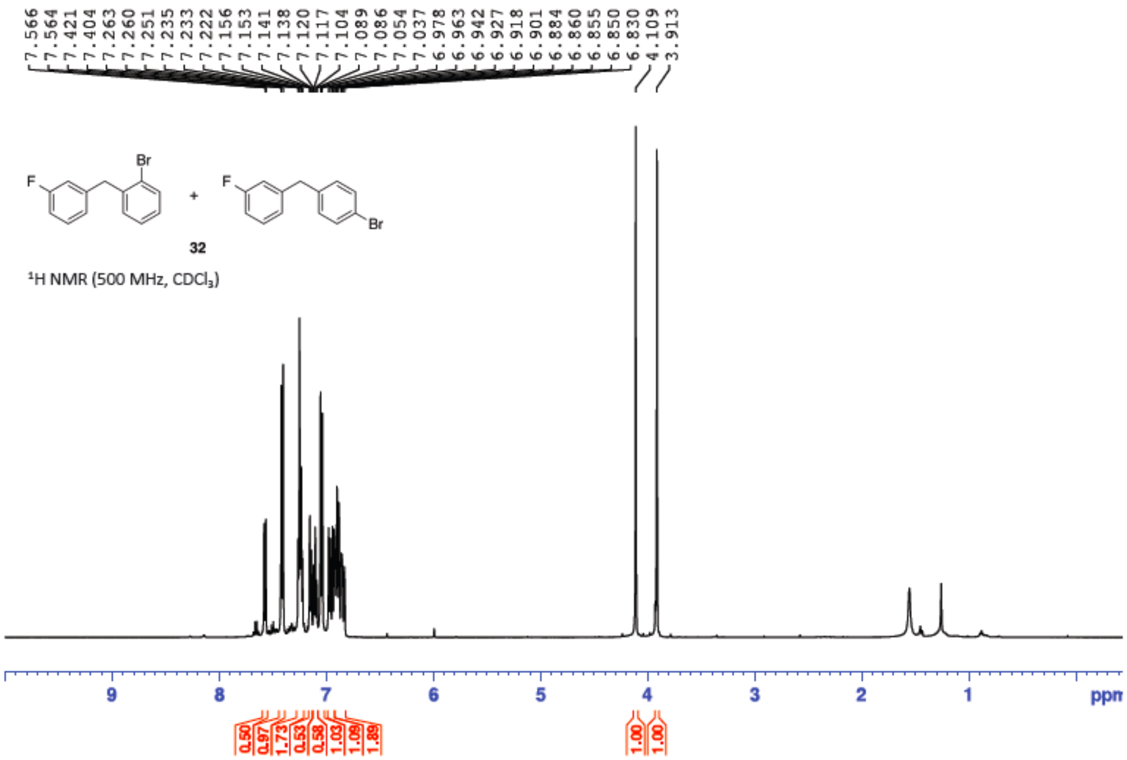
7.566
7.564
7.421
7.404
7.263
7.260
7.251
7.235
7.233
7.222
7.156
7.153
7.141
7.138
7.120
7.117
7.104
7.089
7.086
7.054
7.037
6.978
6.963
6.942
6.927
6.918
6.901
6.884
6.860
6.855
6.850
6.830
4.109
3.913
Br
F
+
F
Br
32
¹H NMR (500 MHz, CDCl₃)
9
8
7
6
5
4
3
2
1
ppm
0.50
0.97
1.73
0.53
0.58
1.03
1.09
1.89
1.00
1.00

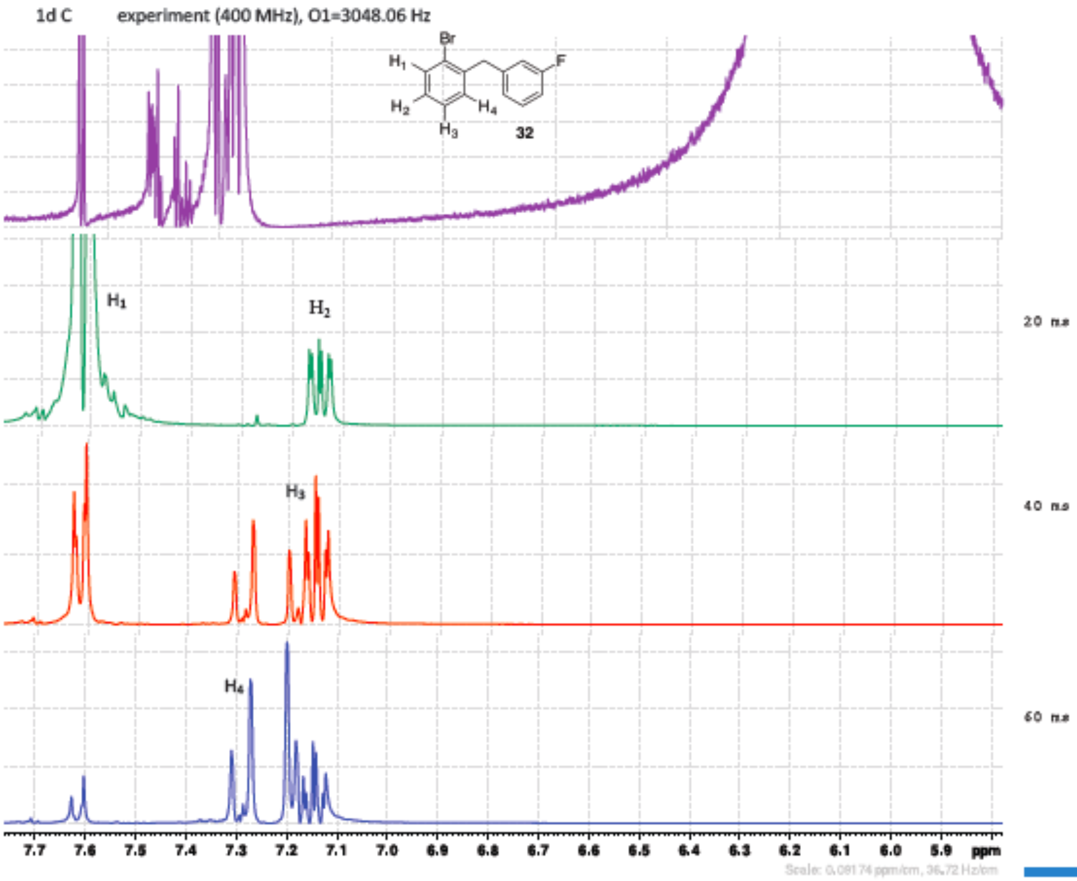
1d C
experiment (400 MHz), O1=3048.06 Hz
Br
F
H1
H2
H3
H4
32
H1
H2
20 ms
H3
40 ms
H4
60 ms
7.7
7.6
7.5
7.4
7.3
7.2
7.1
7.0
6.9
6.8
6.7
6.6
6.5
6.4
6.3
6.2
6.1
6.0
5.9
ppm

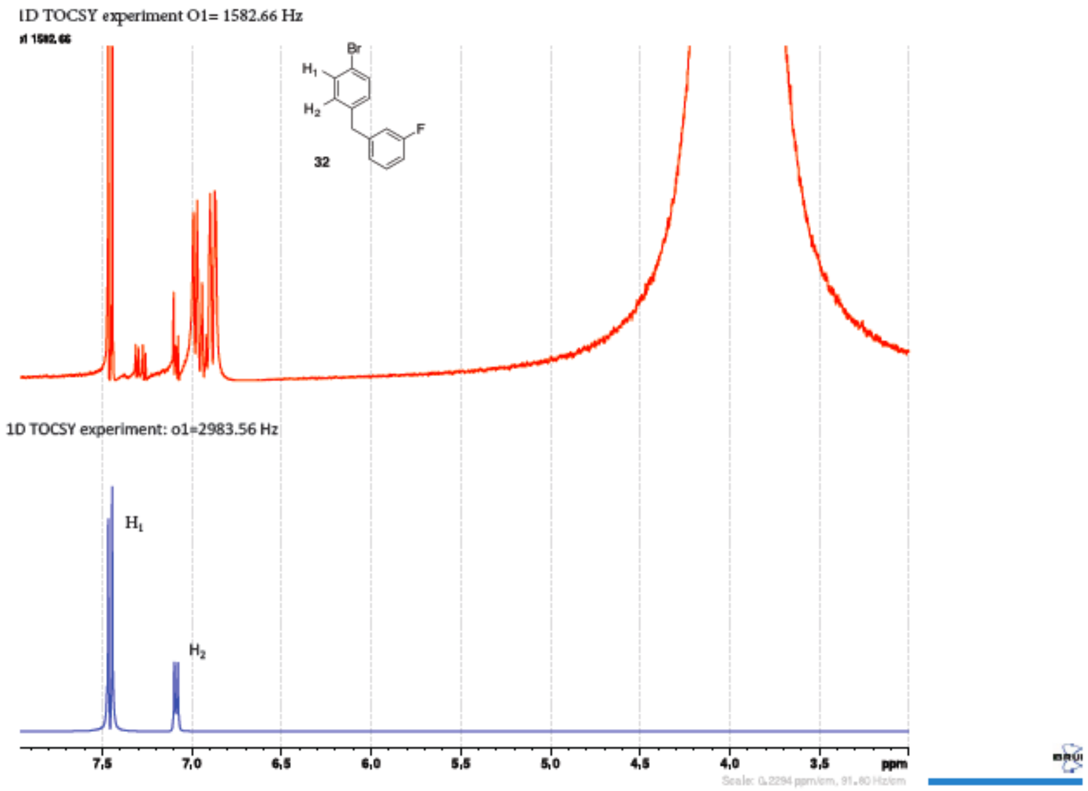
1D TOCSY experiment O1= 1582.66 Hz
Br
H1
H2
F
32
1D TOCSY experiment: o1=2983.56 Hz
H1
H2
7,5
7,0
6,5
6,0
5,5
5,0
4,5
4,0
3,5
ppm

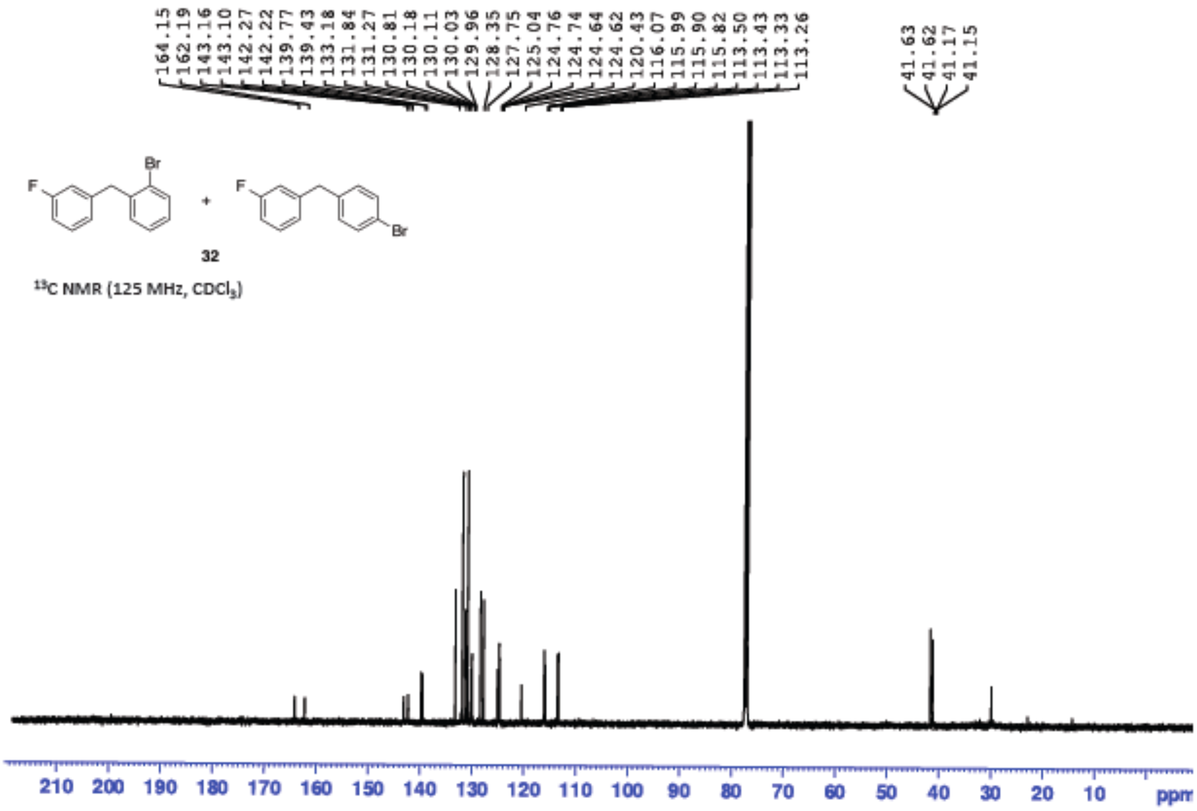
164.15
162.19
143.16
143.10
142.27
142.22
139.77
139.43
133.18
131.84
131.27
130.81
130.18
130.11
130.03
129.96
128.35
127.75
125.04
124.76
124.74
124.64
124.62
120.43
116.07
115.99
115.90
115.82
113.50
113.43
113.33
113.26
41.63
41.62
41.17
41.15
F
Br
+
32
13C NMR (125 MHz, CDCl3)
210
200
190
180
170
160
150
140
130
120
110
100
90
80
70
60
50
40
30
20
10
ppm

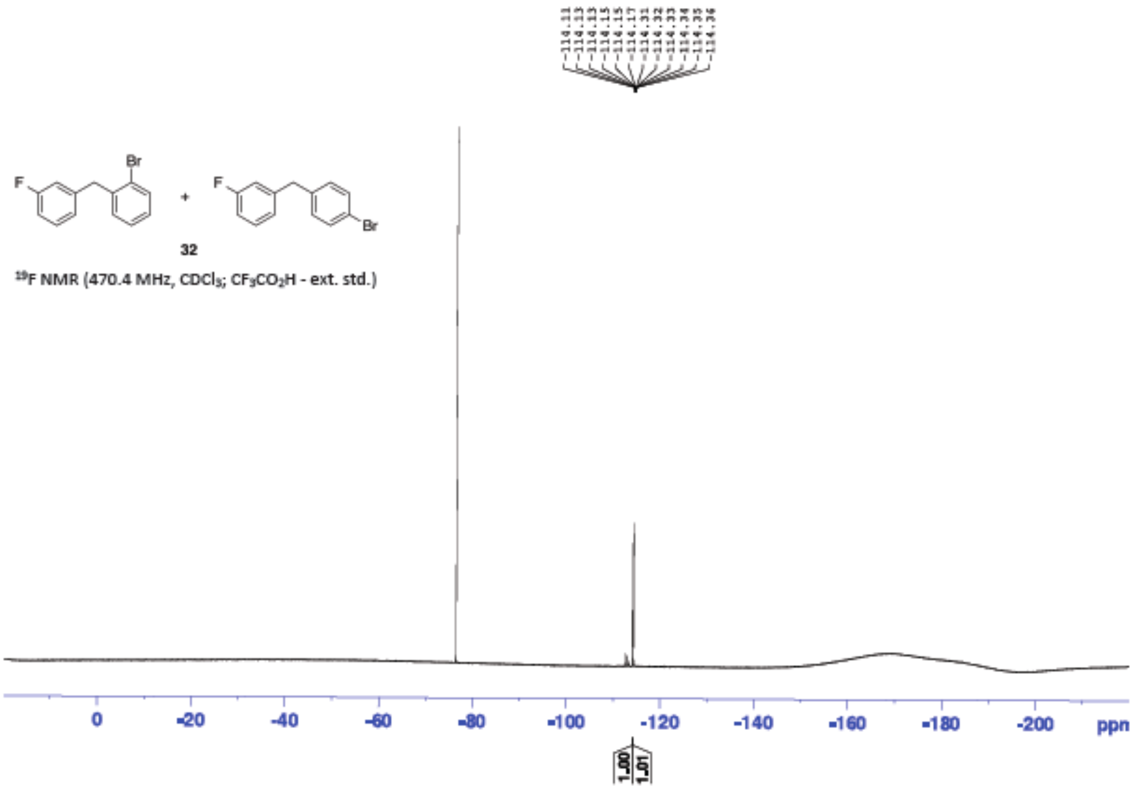

-114.11
-114.13
-114.13
-114.15
-114.15
-114.17
-114.31
-114.32
-114.33
-114.34
-114.35
-114.36
F
Br
+
F
Br
32
^{19}F NMR (470.4 MHz, $CDCl_3$; CF_3CO_2H - ext. std.)
0
-20
-40
-60
-80
-100
-120
-140
-160
-180
-200
ppm
1.00
1.01

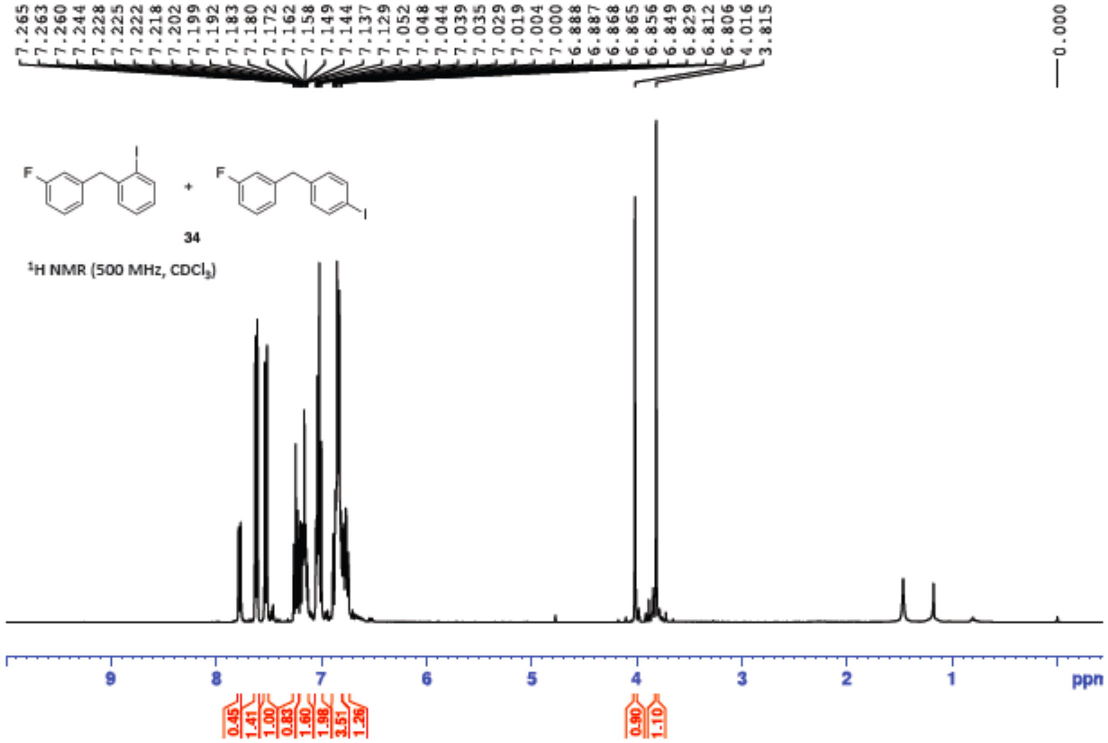
7.265
7.263
7.260
7.244
7.228
7.225
7.222
7.218
7.202
7.199
7.192
7.183
7.180
7.172
7.162
7.158
7.149
7.144
7.137
7.129
7.052
7.048
7.044
7.039
7.035
7.029
7.019
7.004
7.000
6.888
6.887
6.868
6.865
6.856
6.849
6.829
6.812
6.806
4.016
3.815
0.000
F
I
34
^{1}H NMR (500 MHz, CDCl$_3$)
ppm
0.45
1.41
1.00
0.83
1.60
1.98
3.51
1.26
0.90
1.10

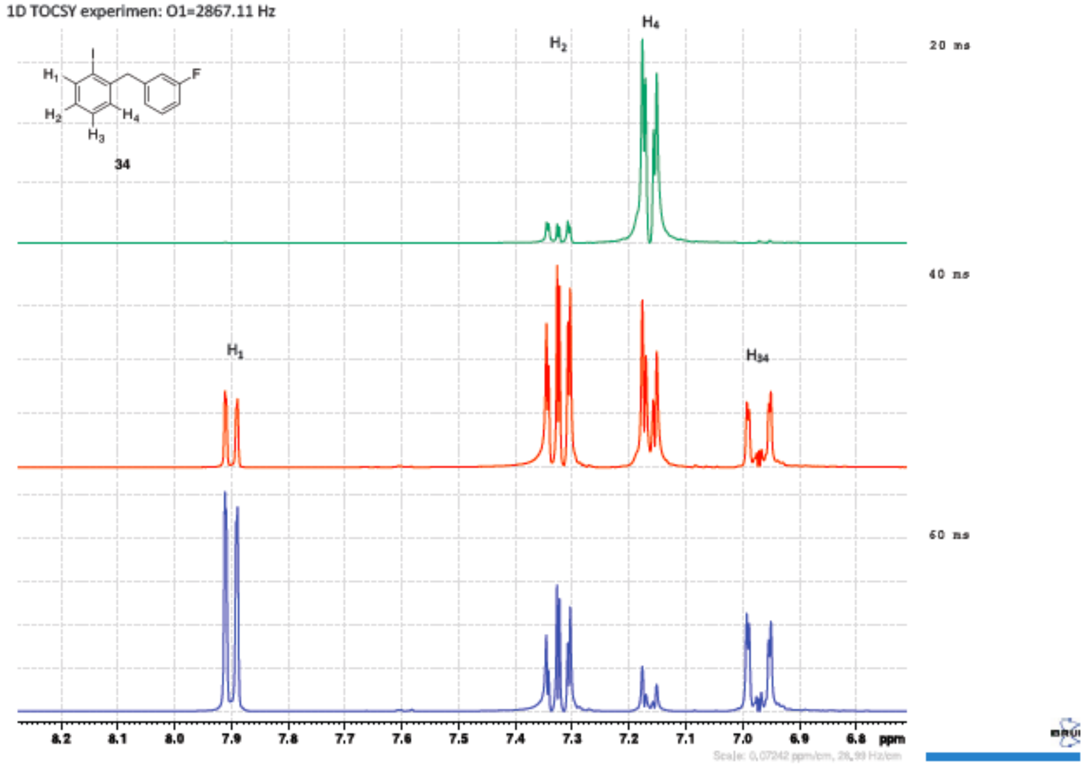
1D TOCSY experimen: O1=2867.11 Hz
H1
H2
H3
H4
I
F
34
20 ms
40 ms
60 ms
H34
ppm

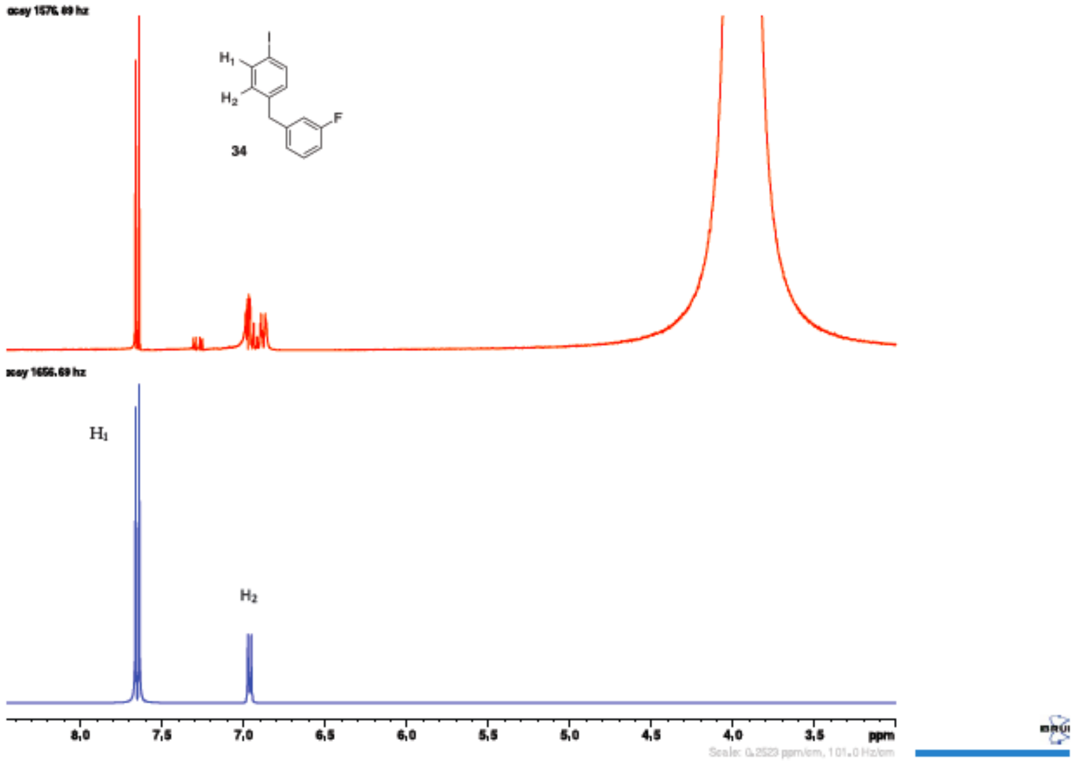
34
H1
H2
8,0 7,5 7,0 6,5 6,0 5,5 5,0 4,5 4,0 3,5 ppm

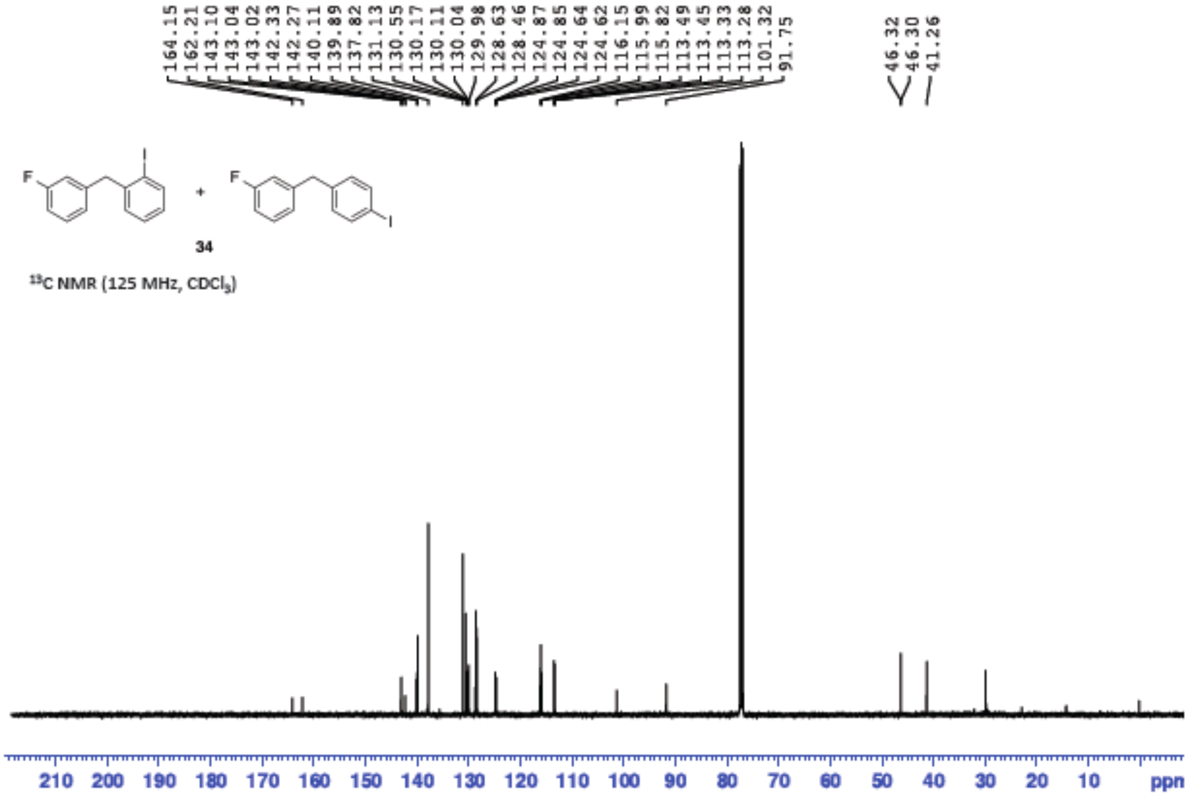
164.15
162.21
143.10
143.04
143.02
142.33
142.27
140.11
139.89
137.82
131.13
130.55
130.17
130.11
130.04
129.98
128.63
128.46
124.87
124.85
124.64
124.62
116.15
115.99
115.82
113.49
113.45
113.33
113.28
101.32
91.75
46.32
46.30
41.26
34
13C NMR (125 MHz, CDCl3)
210 200 190 180 170 160 150 140 130 120 110 100 90 80 70 60 50 40 30 20 10 ppm

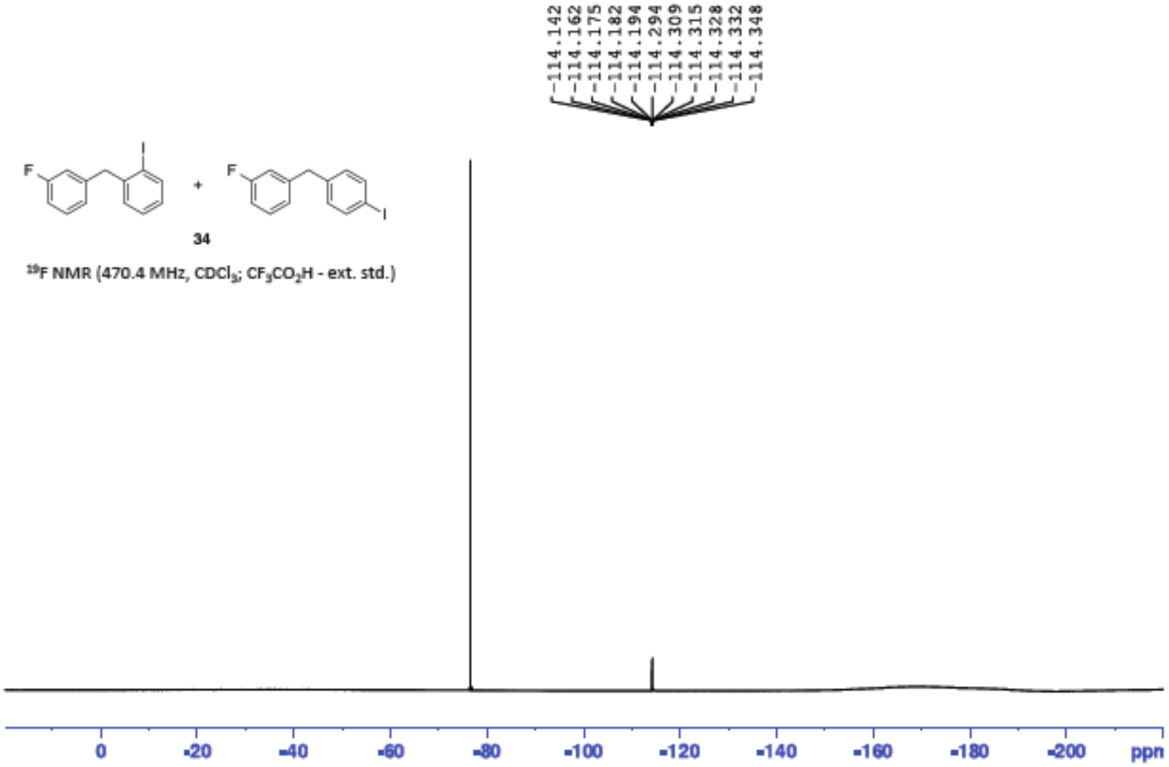
34
^{19}F NMR (470.4 MHz, $CDCl_3$; CF_3CO_2H - ext. std.)
-114.142
-114.162
-114.175
-114.182
-114.194
-114.294
-114.309
-114.315
-114.328
-114.332
-114.348
0
-20
-40
-60
-80
-100
-120
-140
-160
-180
-200
ppm

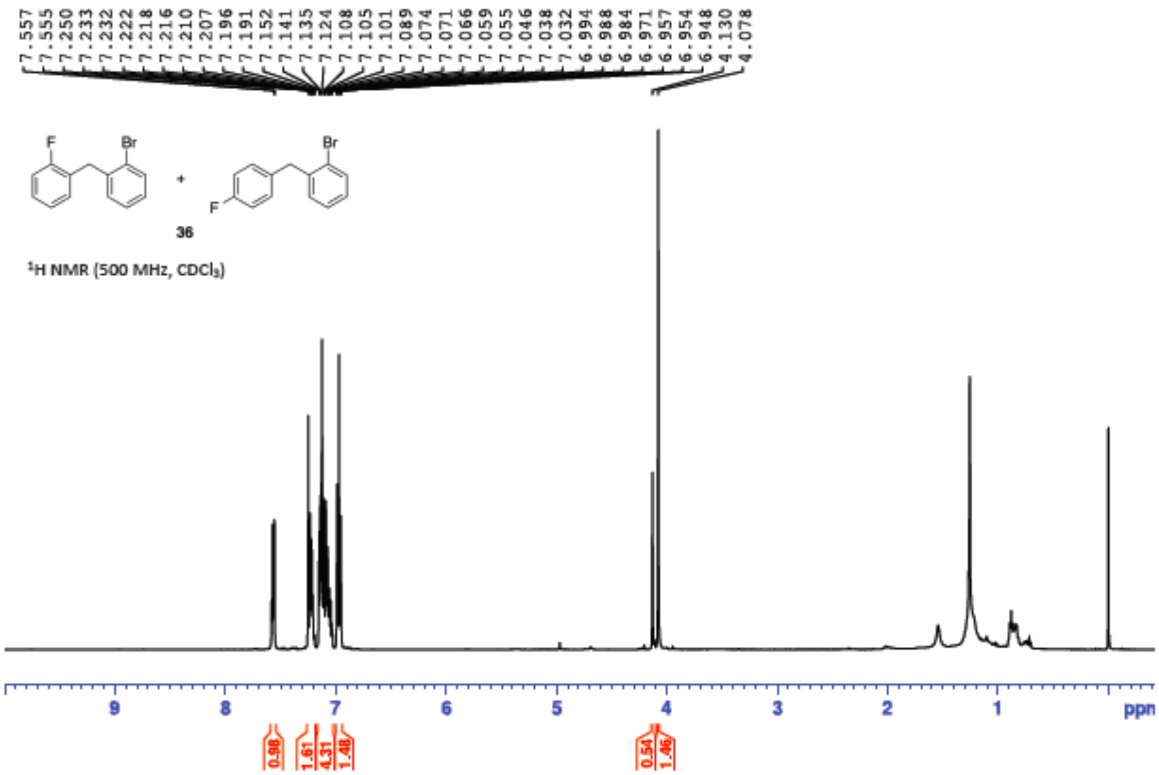
7.557 7.555 7.250 7.233 7.232 7.222 7.218 7.216 7.210 7.207 7.196 7.191 7.152 7.141 7.135 7.124 7.108 7.105 7.101 7.089 7.074 7.071 7.066 7.059 7.055 7.046 7.038 7.032 6.994 6.988 6.984 6.971 6.957 6.954 6.948 4.130 4.078
F
Br
+
36
1H NMR (500 MHz, CDCl3)
9 8 7 6 5 4 3 2 1 ppm
0.98 1.61 4.31 1.48 0.54 1.46

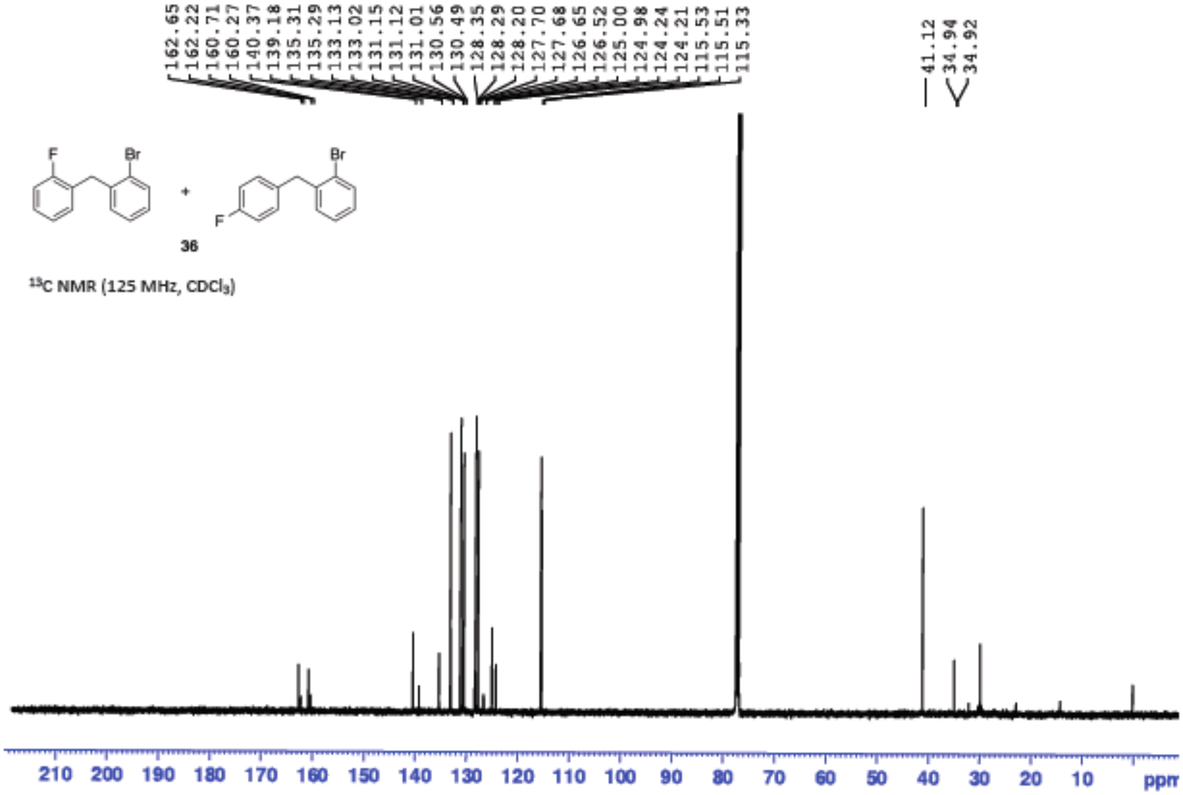
162.65 162.22 160.71 160.27 140.37 139.18 135.31 135.29 133.13 133.02 131.15 131.12 131.01 130.56 130.49 128.35 128.29 128.20 127.70 127.68 126.65 126.52 125.00 124.98 124.24 124.21 115.53 115.51 115.33
41.12 34.94 34.92
F
Br
+
36
13C NMR (125 MHz, CDCl3)
210 200 190 180 170 160 150 140 130 120 110 100 90 80 70 60 50 40 30 20 10 ppm

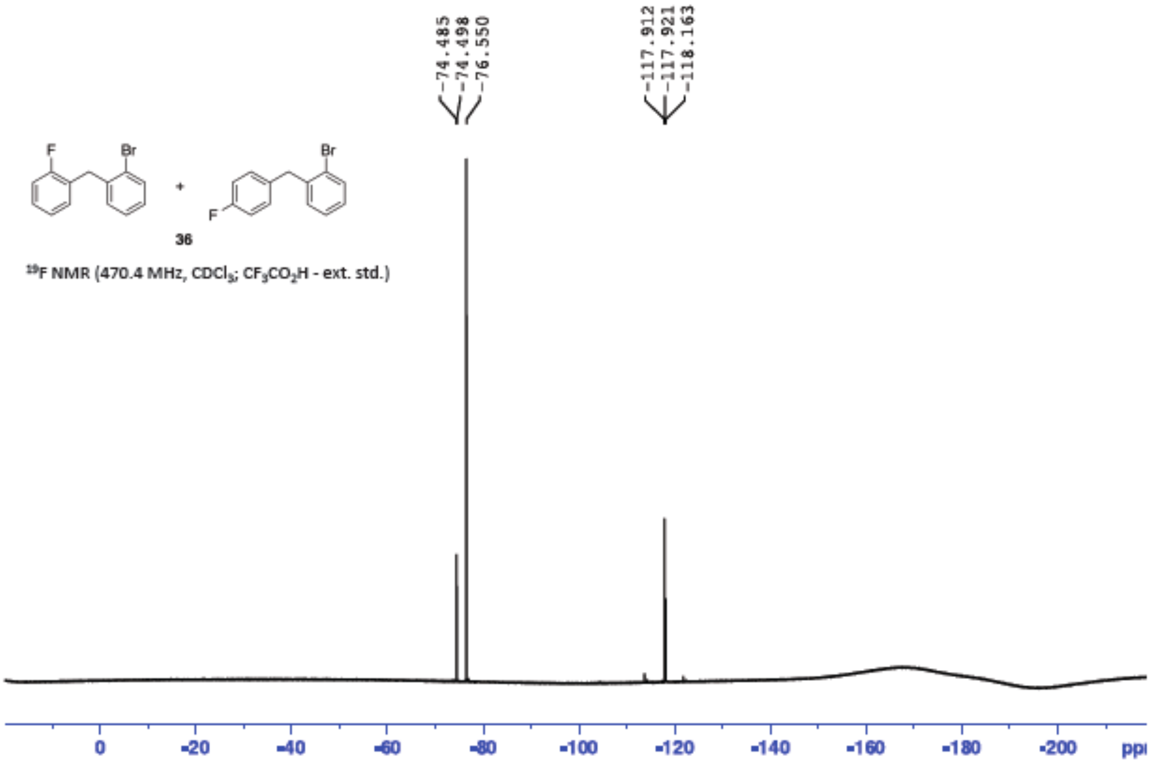

F
Br
+
Br
F
36
^{19}F NMR (470.4 MHz, $CDCl_3$; CF_3CO_2H - ext. std.)
-74.485
-74.498
-76.550
-117.912
-117.921
-118.163
0
-20
-40
-60
-80
-100
-120
-140
-160
-180
-200
ppm

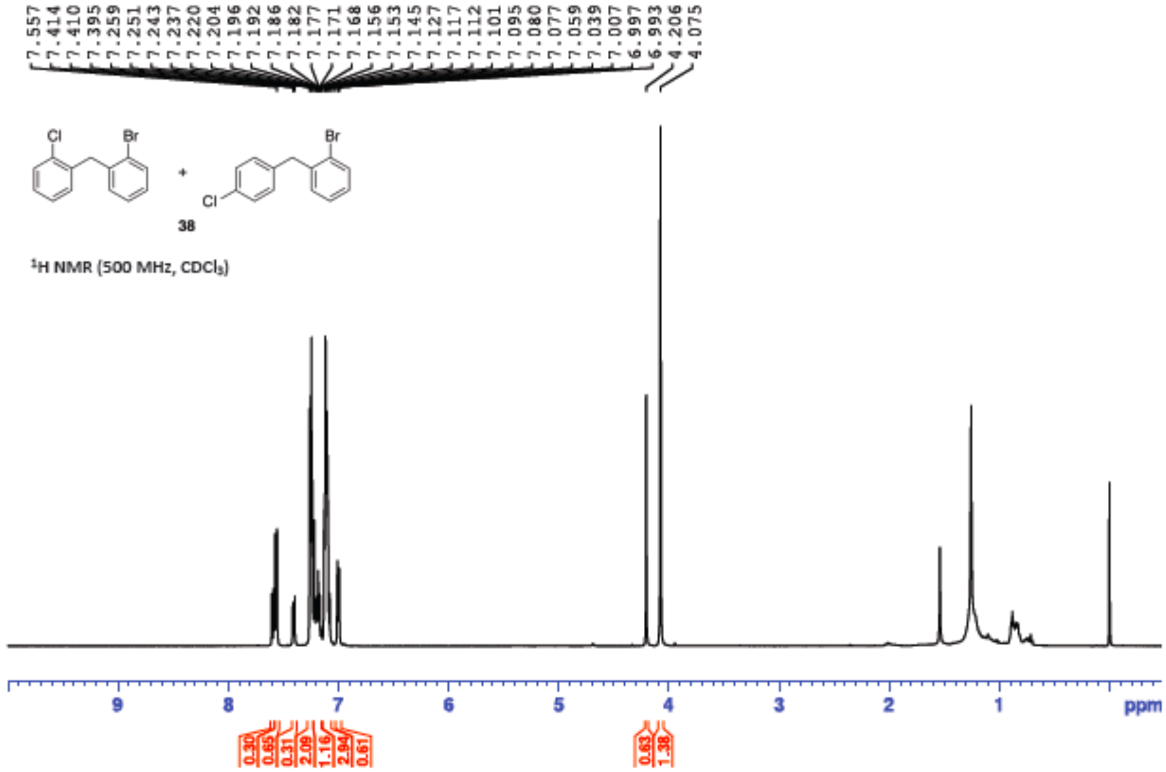
7.557
7.414
7.410
7.395
7.259
7.251
7.243
7.237
7.220
7.204
7.196
7.192
7.186
7.182
7.177
7.171
7.168
7.156
7.153
7.145
7.127
7.117
7.112
7.101
7.095
7.080
7.077
7.059
7.039
7.007
6.997
6.993
4.206
4.075
Cl
Br
+
Cl
Br
38
^{1}H NMR (500 MHz, CDCl$_3$)
9
8
7
6
5
4
3
2
1
ppm
0.30
0.65
0.31
2.09
1.16
2.94
0.61
0.63
1.38

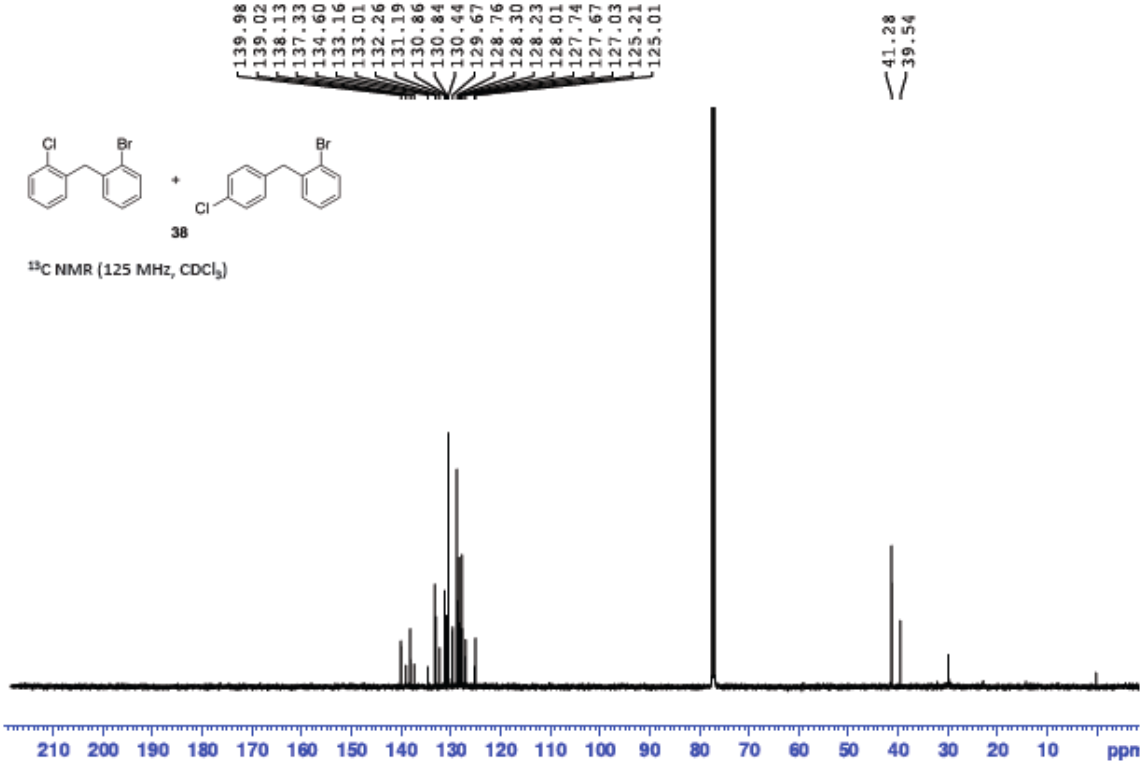
139.98
139.02
138.13
137.33
134.60
133.16
133.01
132.26
131.19
130.86
130.84
130.44
129.67
128.76
128.30
128.23
128.01
127.74
127.67
127.03
125.21
125.01
41.28
39.54
Cl
Br
+
Cl
Br
38
^{13}C NMR (125 MHz, CDCl$_3$)
210
200
190
180
170
160
150
140
130
120
110
100
90
80
70
60
50
40
30
20
10
ppm

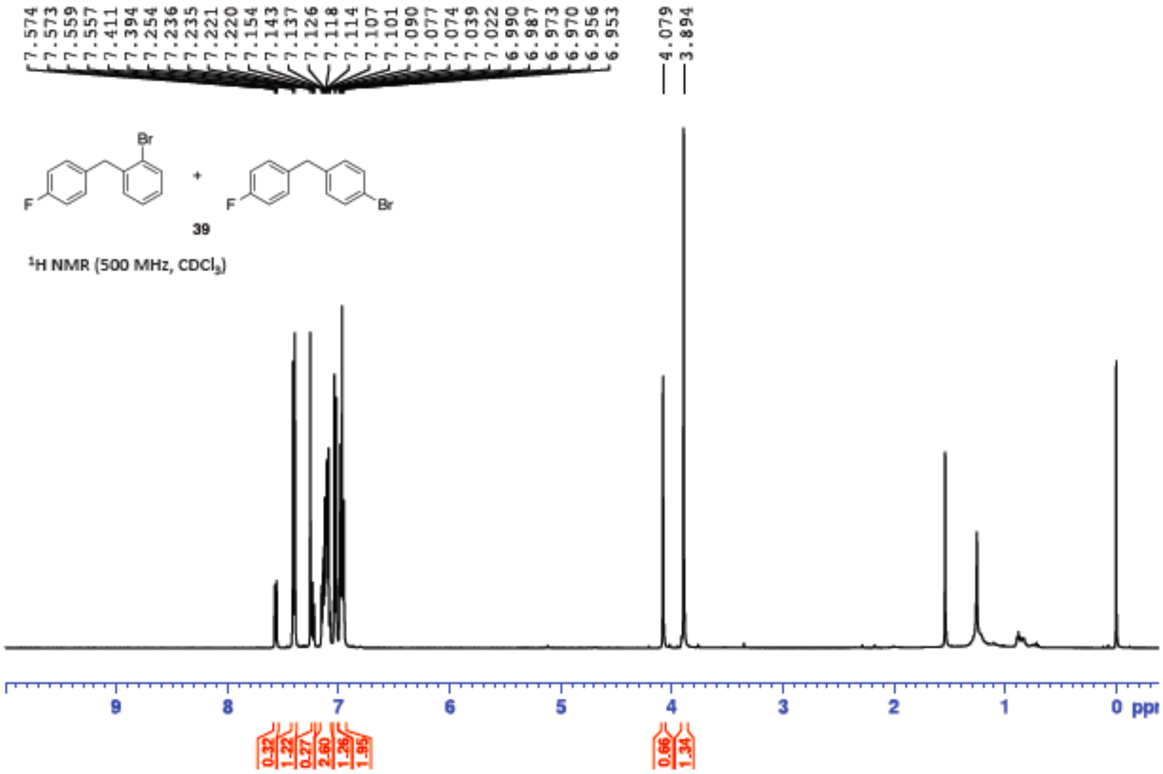
7.574 7.573 7.559 7.557 7.411 7.394 7.254 7.236 7.235 7.221 7.220 7.154 7.143 7.137 7.126 7.118 7.114 7.107 7.101 7.090 7.077 7.074 7.039 7.022 6.990 6.987 6.973 6.970 6.956 6.953
4.079 3.894
39
¹H NMR (500 MHz, CDCl₃)
ppm
0.32 1.22 0.27 2.60 1.26 1.95
0.66 1.34

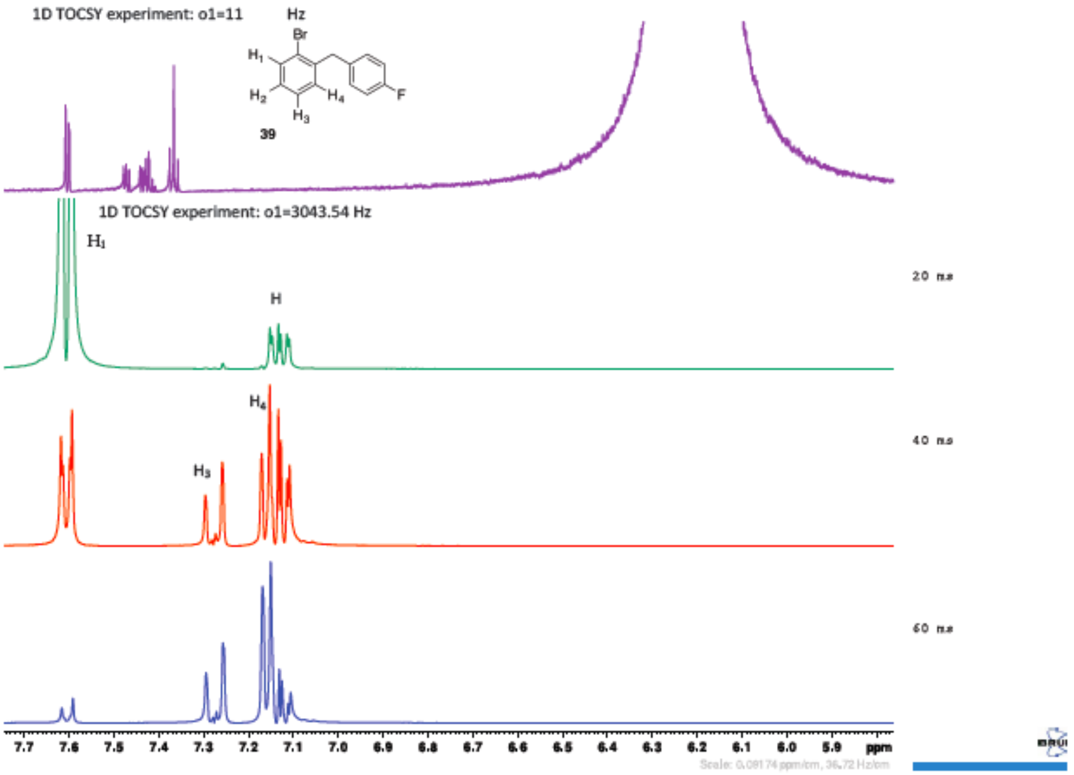
1D TOCSY experiment: o1=11 Hz
39
1D TOCSY experiment: o1=3043.54 Hz
H₁
H
H₄
H₃
20 ms
40 ms
60 ms
ppm

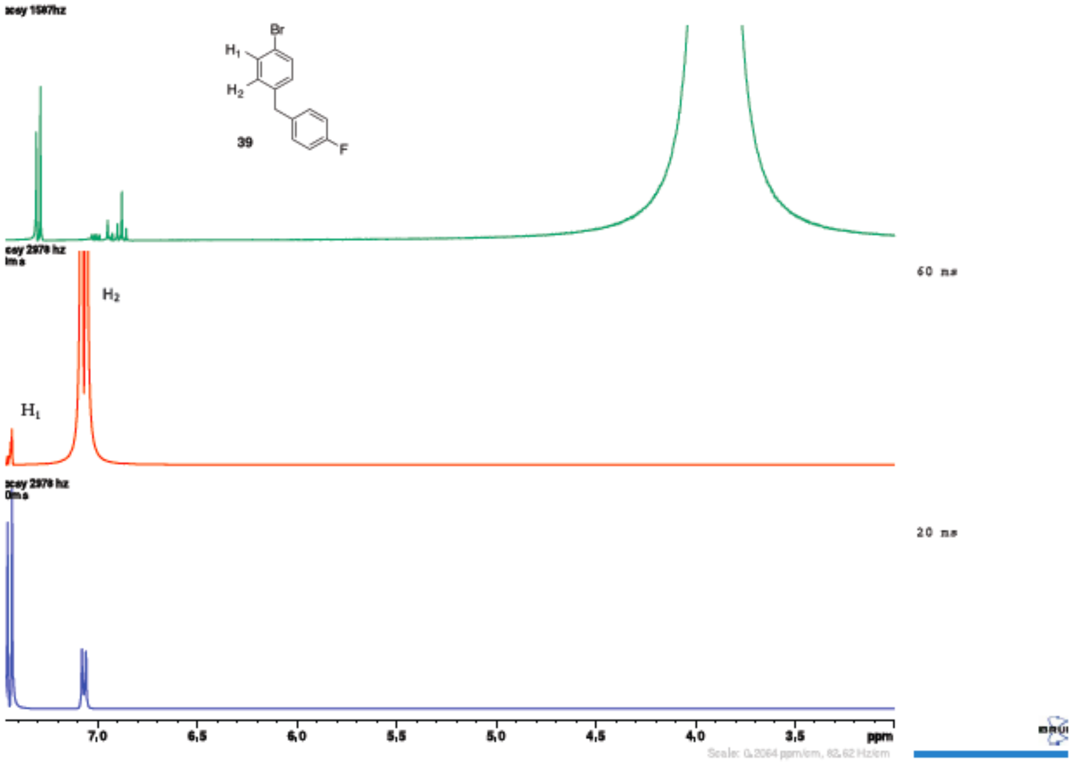

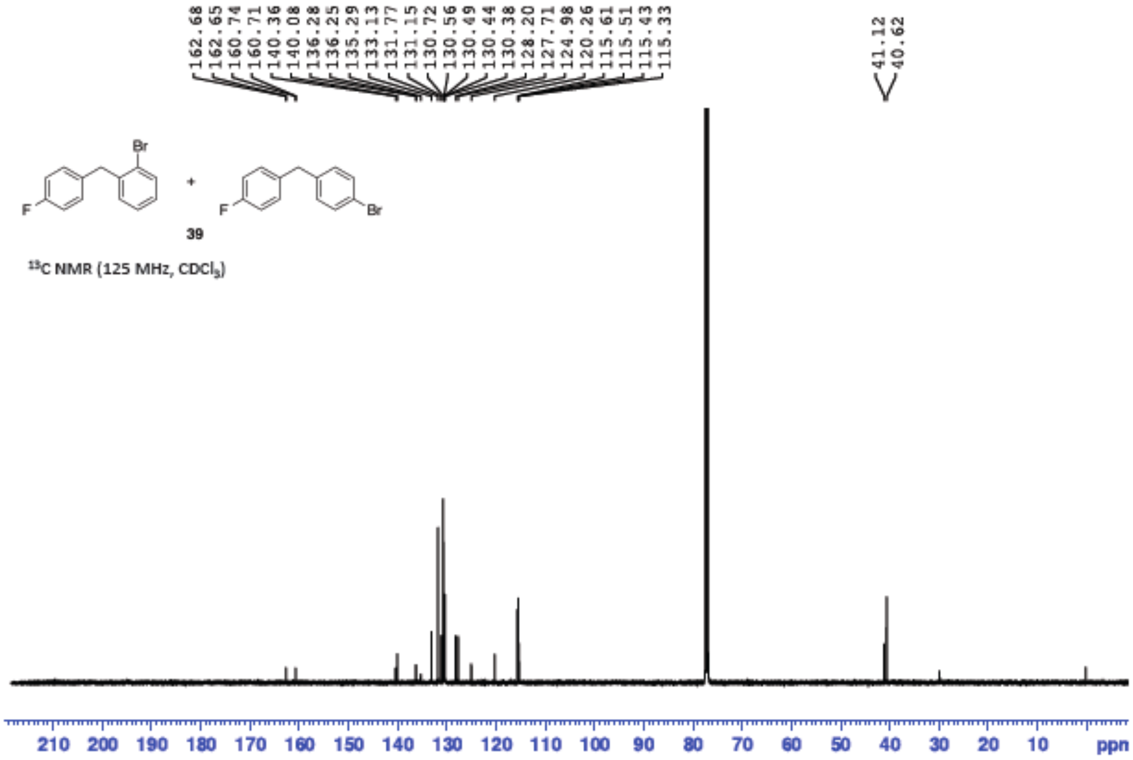

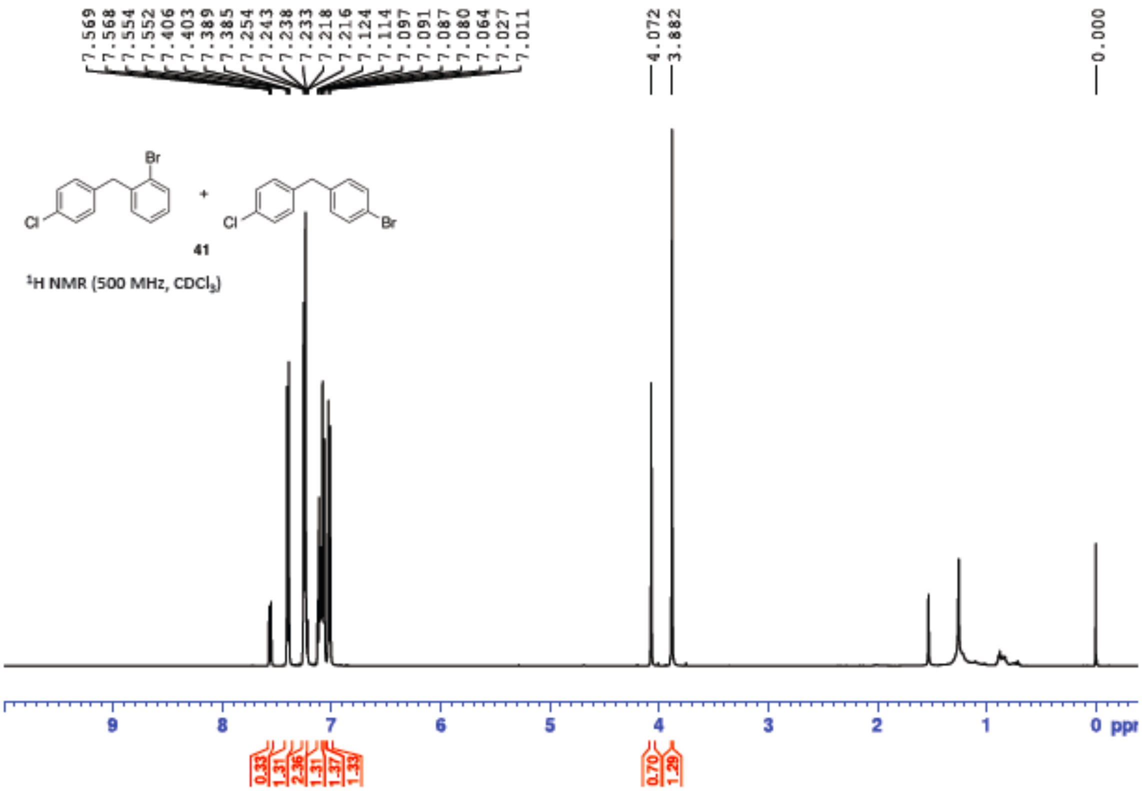
41
¹H NMR (500 MHz, CDCl₃)

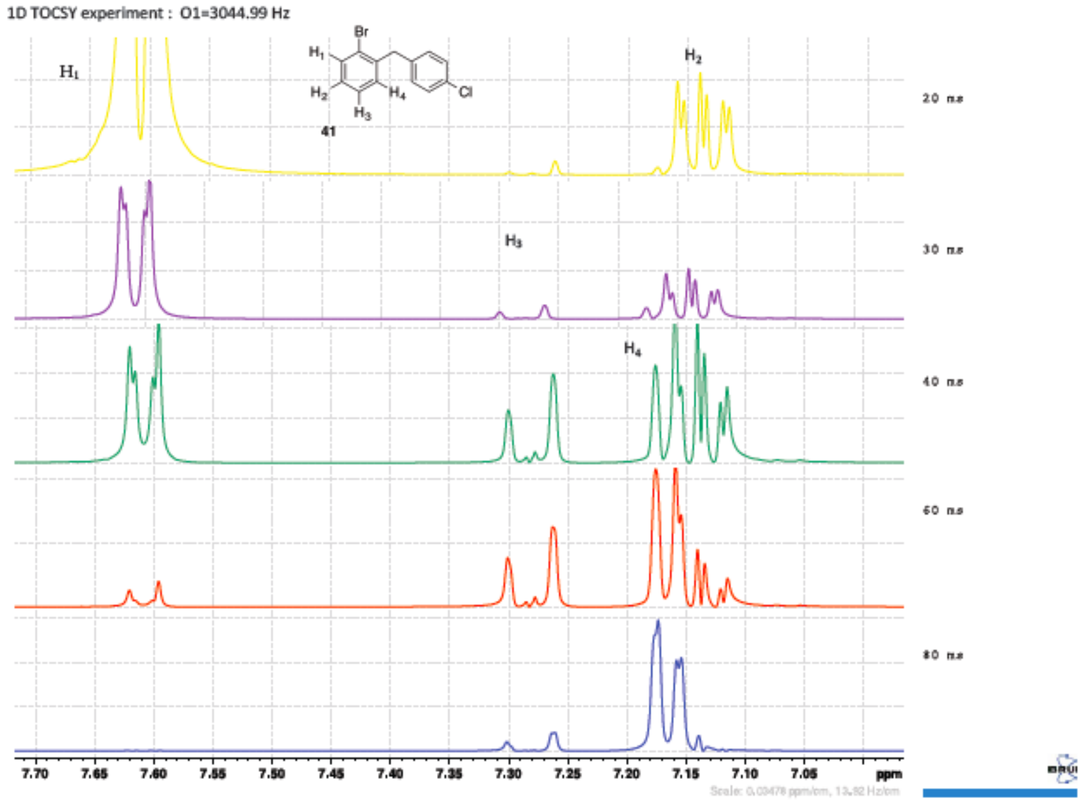
1D TOCSY experiment : O1=3044.99 Hz
41

1D TOCSY experiment: O1=2828.21 Hz

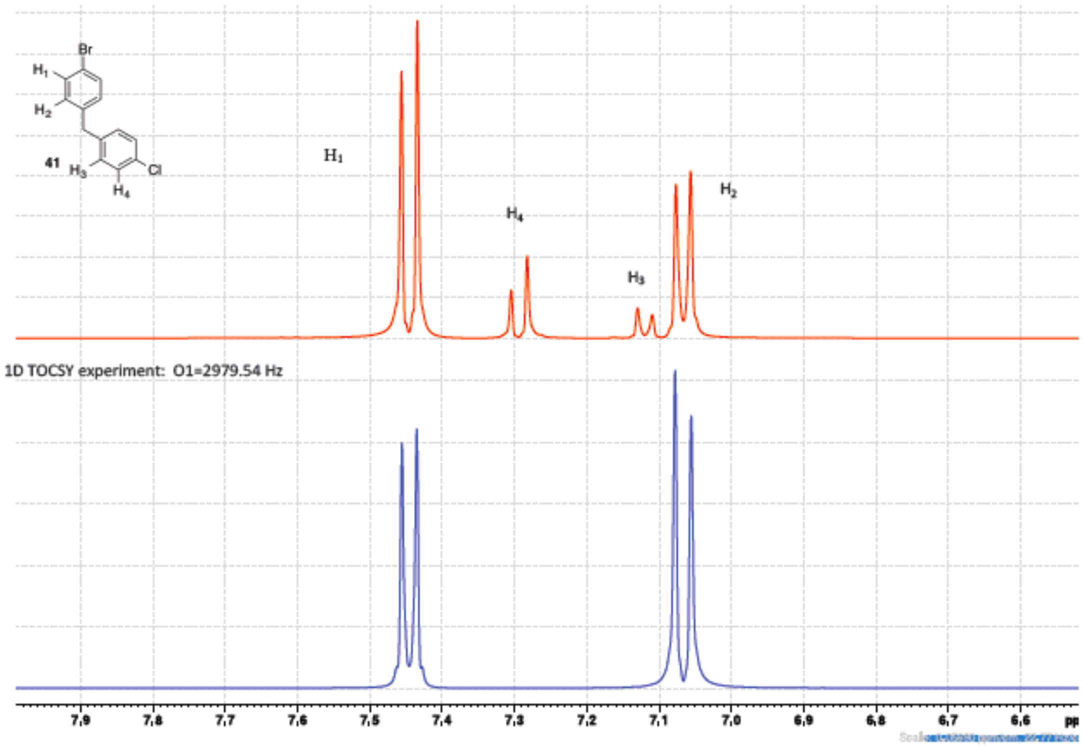

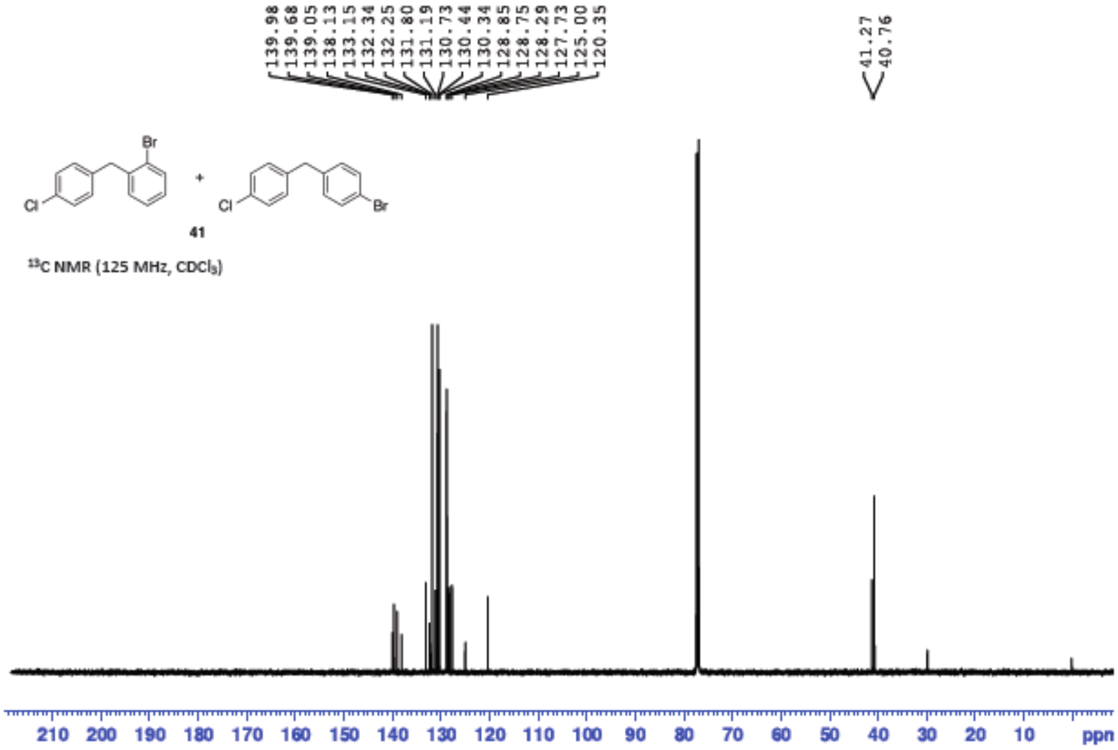

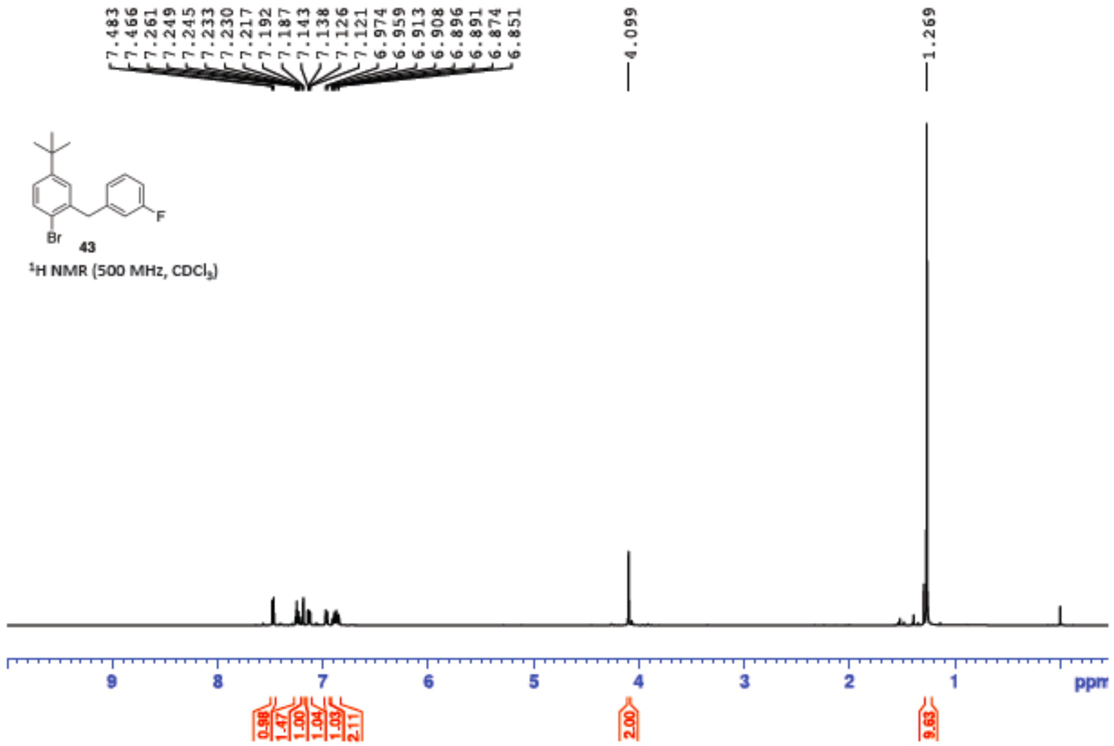
7.483
7.466
7.261
7.249
7.245
7.233
7.230
7.217
7.192
7.187
7.143
7.138
7.126
7.121
6.974
6.959
6.913
6.908
6.896
6.891
6.874
6.851
4.099
1.269
Br
F
43
^{1}H NMR (500 MHz, CDCl$_3$)
9
8
7
6
5
4
3
2
1
ppm
0.98
1.47
1.00
1.04
1.03
2.11
2.00
9.63

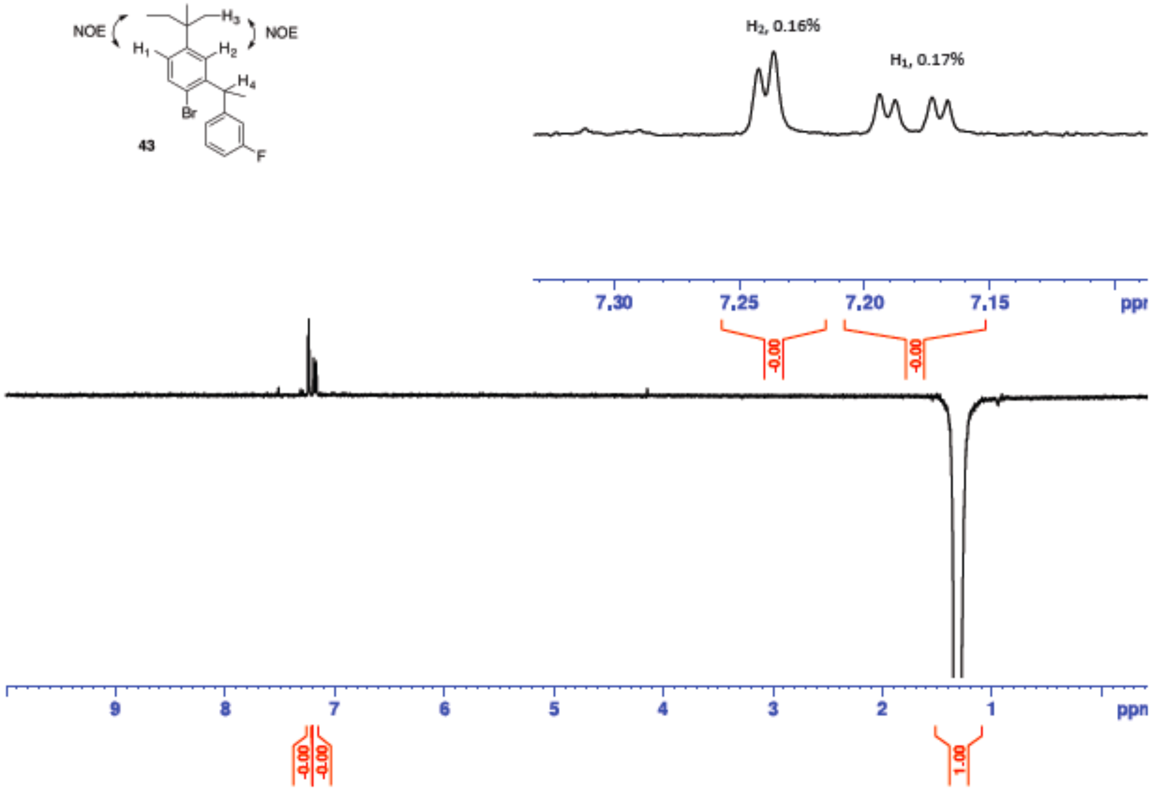
NOE
NOE
H$_1$
H$_2$
H$_3$
H$_4$
Br
F
43
H$_2$, 0.16%
H$_1$, 0.17%
7,30
7,25
7,20
7,15
ppm
-0.00
-0.00
9
8
7
6
5
4
3
2
1
ppm
-0.00
-0.00
1.00

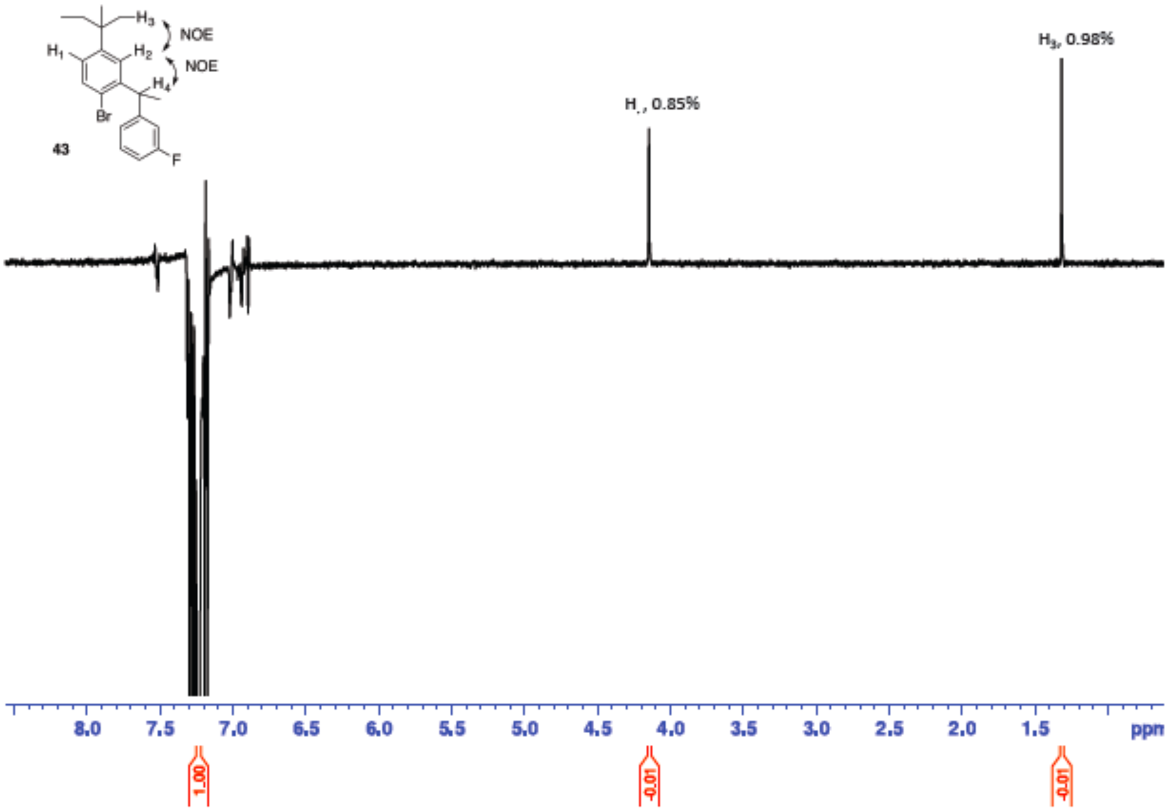
NOE
NOE
43
H, 0.85%
H3, 0.98%
8.0
7.5
7.0
6.5
6.0
5.5
5.0
4.5
4.0
3.5
3.0
2.5
2.0
1.5
ppm
1.00
-0.01
-0.01

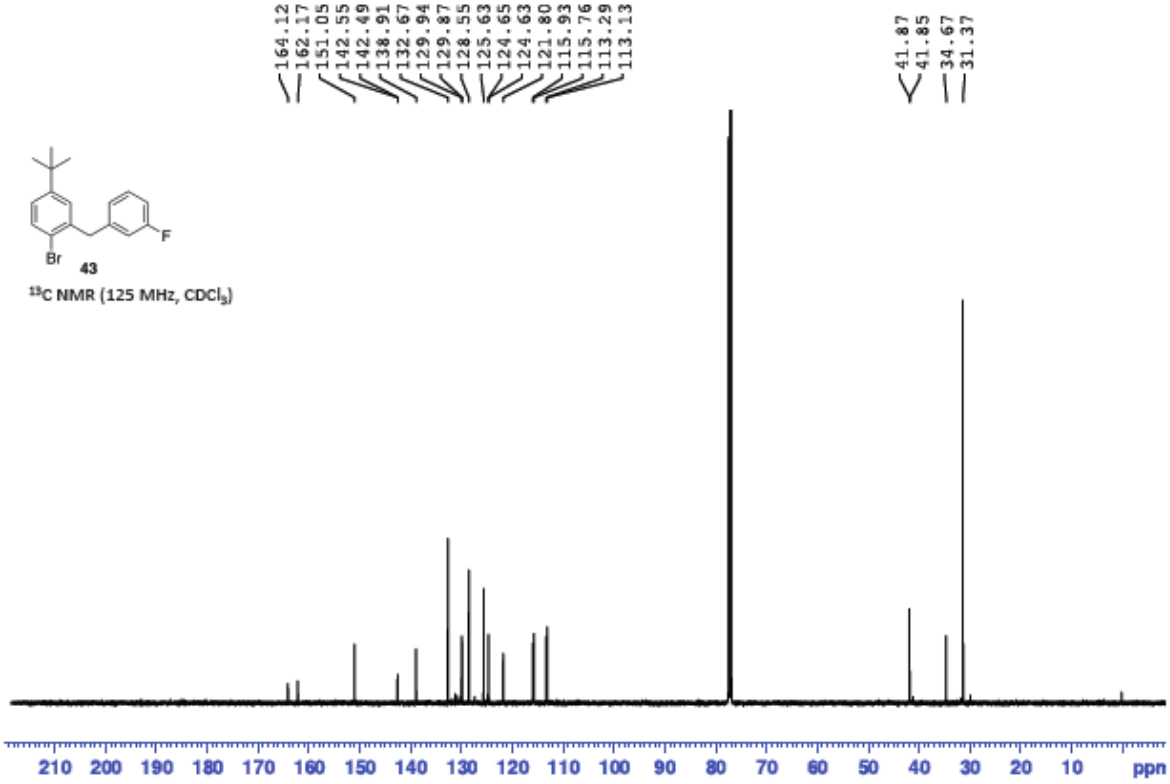
164.12
162.17
151.05
142.55
142.49
138.91
132.67
129.94
129.87
128.55
125.63
124.65
124.63
121.80
115.93
115.76
113.29
113.13
41.87
41.85
34.67
31.37
43
13C NMR (125 MHz, CDCl3)
210 200 190 180 170 160 150 140 130 120 110 100 90 80 70 60 50 40 30 20 10 ppm

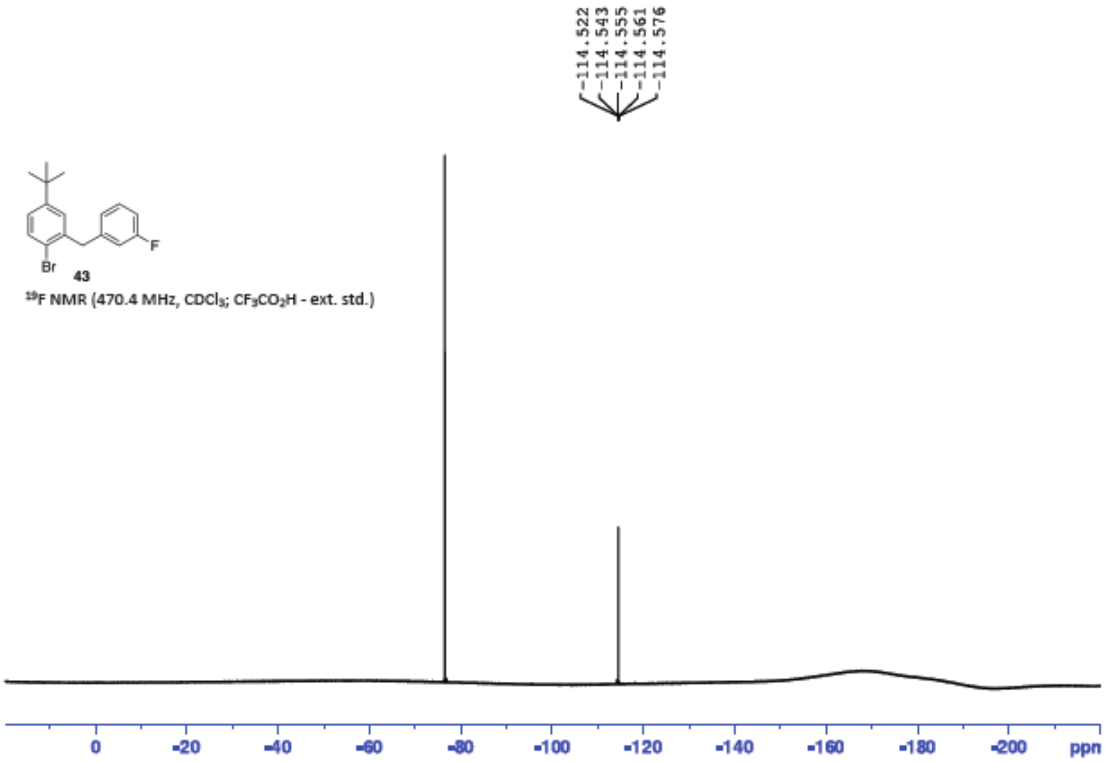
-114.522
-114.543
-114.555
-114.561
-114.576
43
^{19}F NMR (470.4 MHz, $CDCl_3$; CF_3CO_2H - ext. std.)
0
-20
-40
-60
-80
-100
-120
-140
-160
-180
-200
ppm

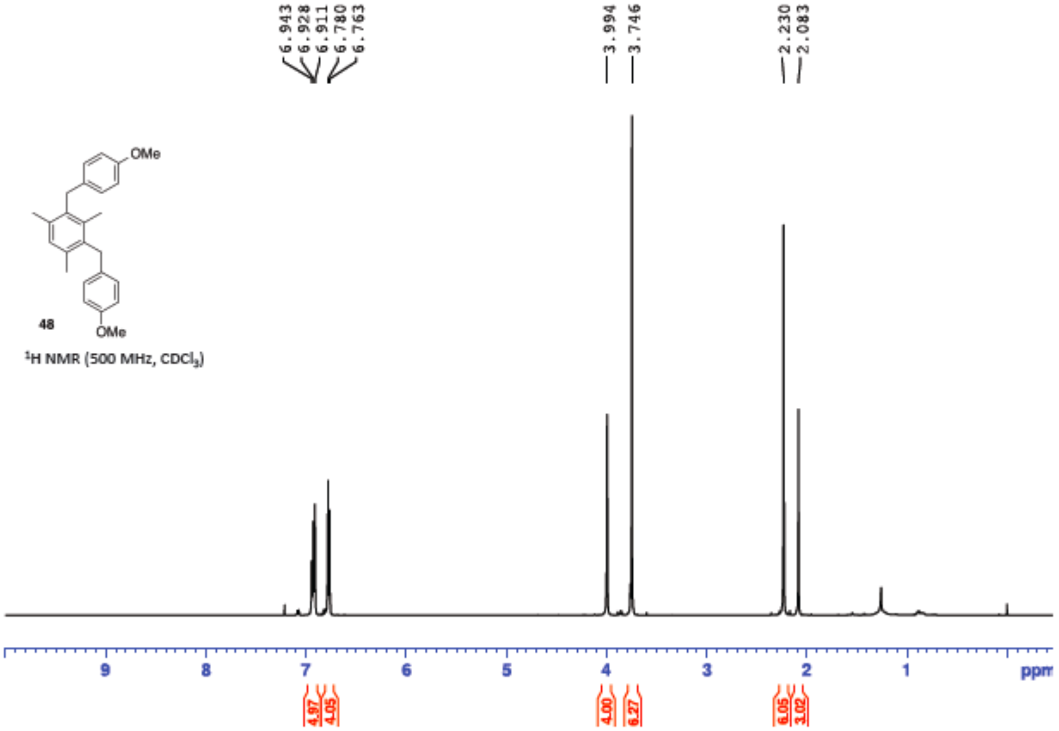
6.943
6.928
6.911
6.780
6.763
3.994
3.746
2.230
2.083
OMe
48
OMe
1H NMR (500 MHz, CDCl3)
9 8 7 6 5 4 3 2 1 ppm
4.97
4.05
4.00
6.27
6.05
3.02

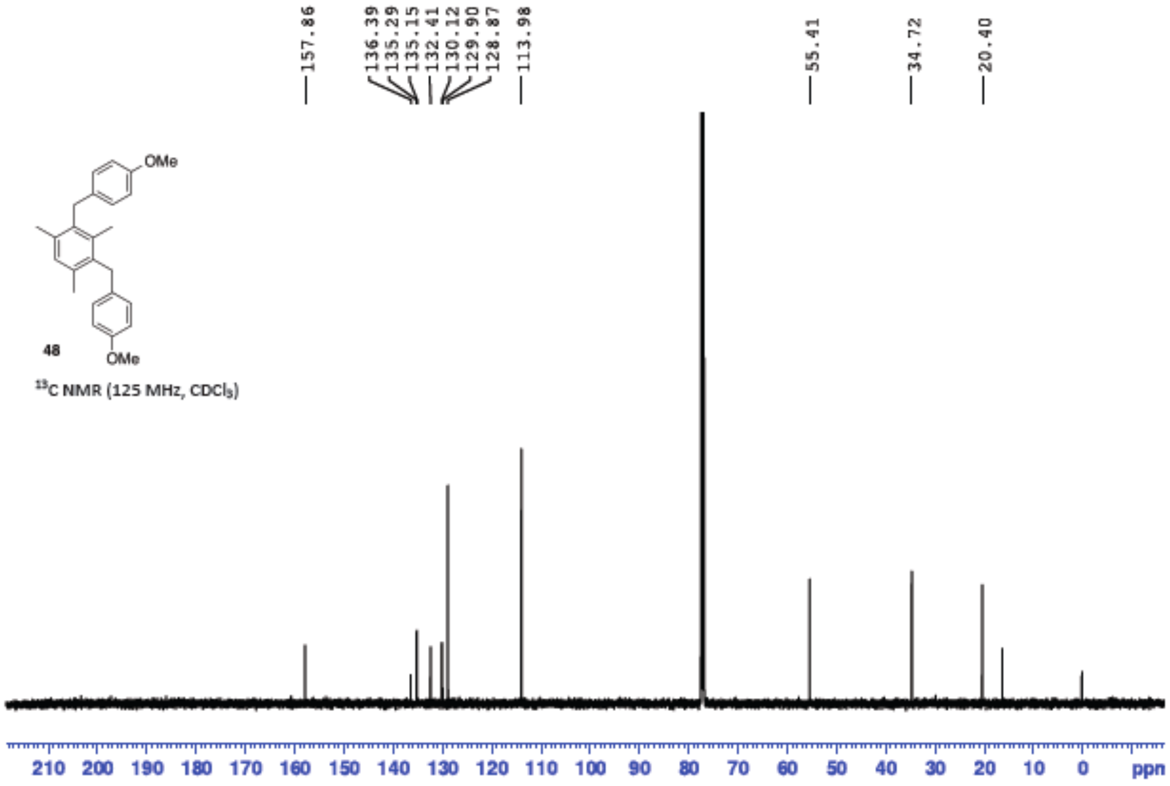
157.86
136.39
135.29
135.15
132.41
130.12
129.90
128.87
113.98
55.41
34.72
20.40
OMe
48
OMe
13C NMR (125 MHz, CDCl3)
210 200 190 180 170 160 150 140 130 120 110 100 90 80 70 60 50 40 30 20 10 0 ppm

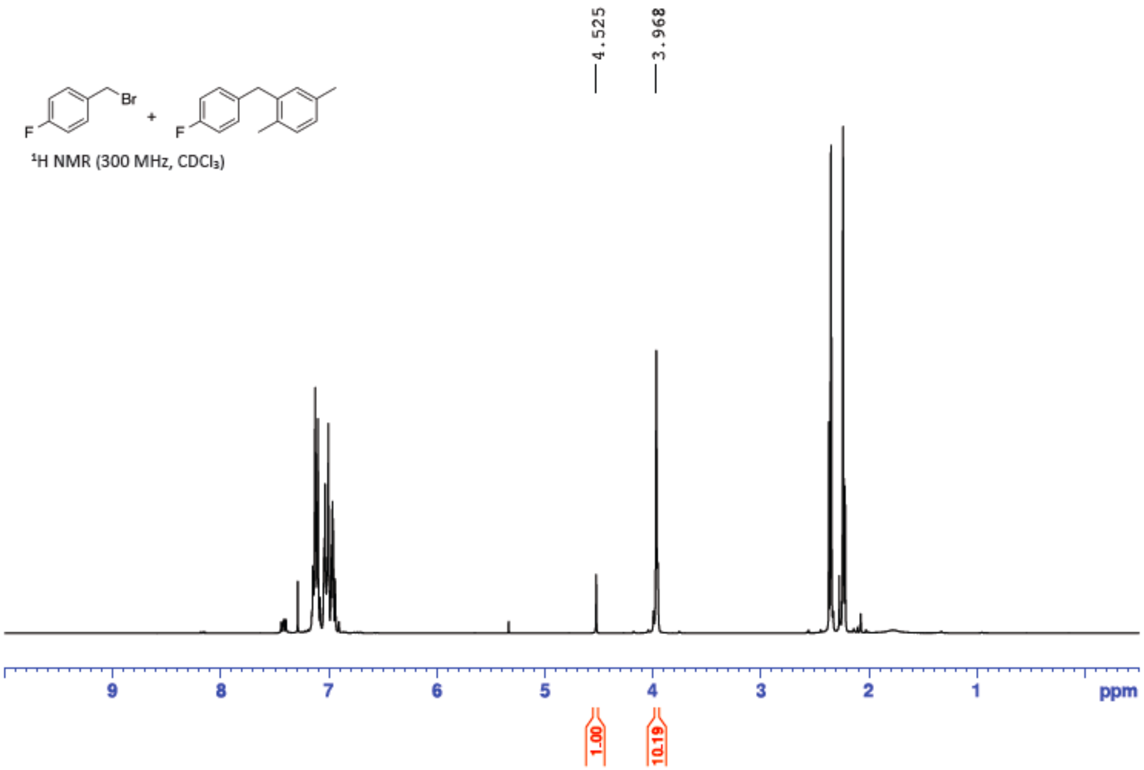

Br
F
F
¹H NMR (300 MHz, CDCl₃)
4.525
3.968
9
8
7
6
5
4
3
2
1
ppm
1.00
10.19

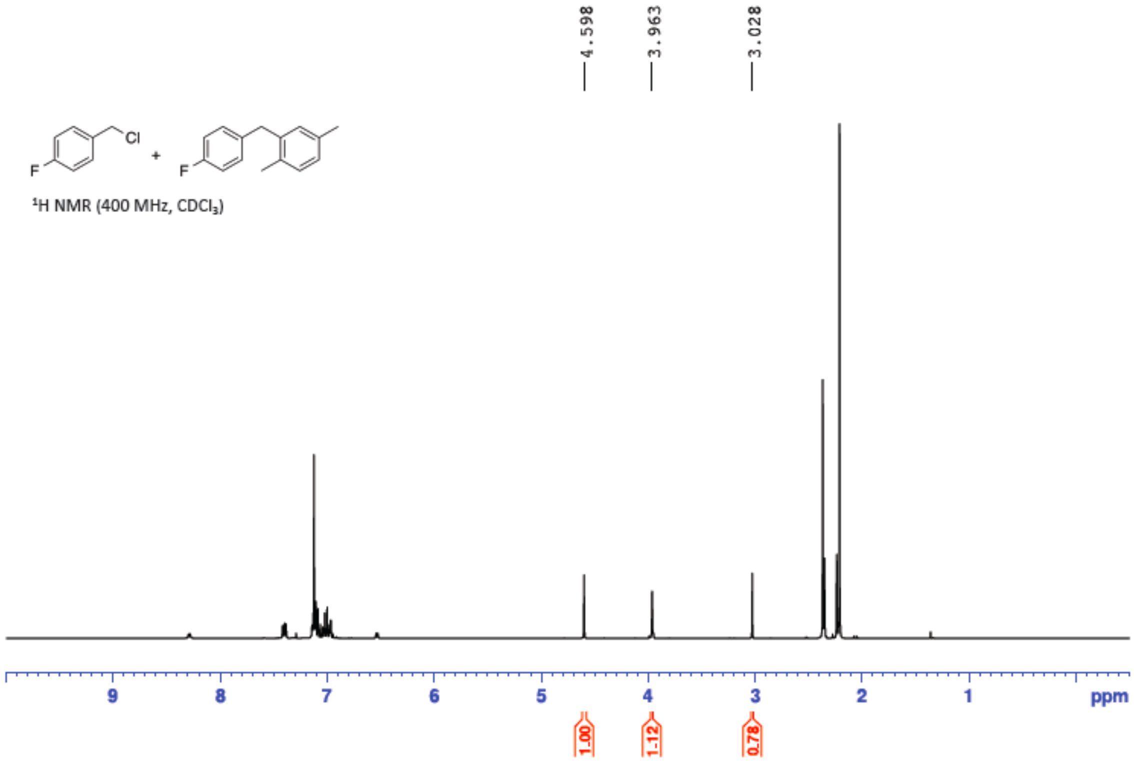

Cl
F
F
¹H NMR (400 MHz, CDCl₃)
4.598
3.963
3.028
9
8
7
6
5
4
3
2
1
ppm
1.00
1.12
0.78

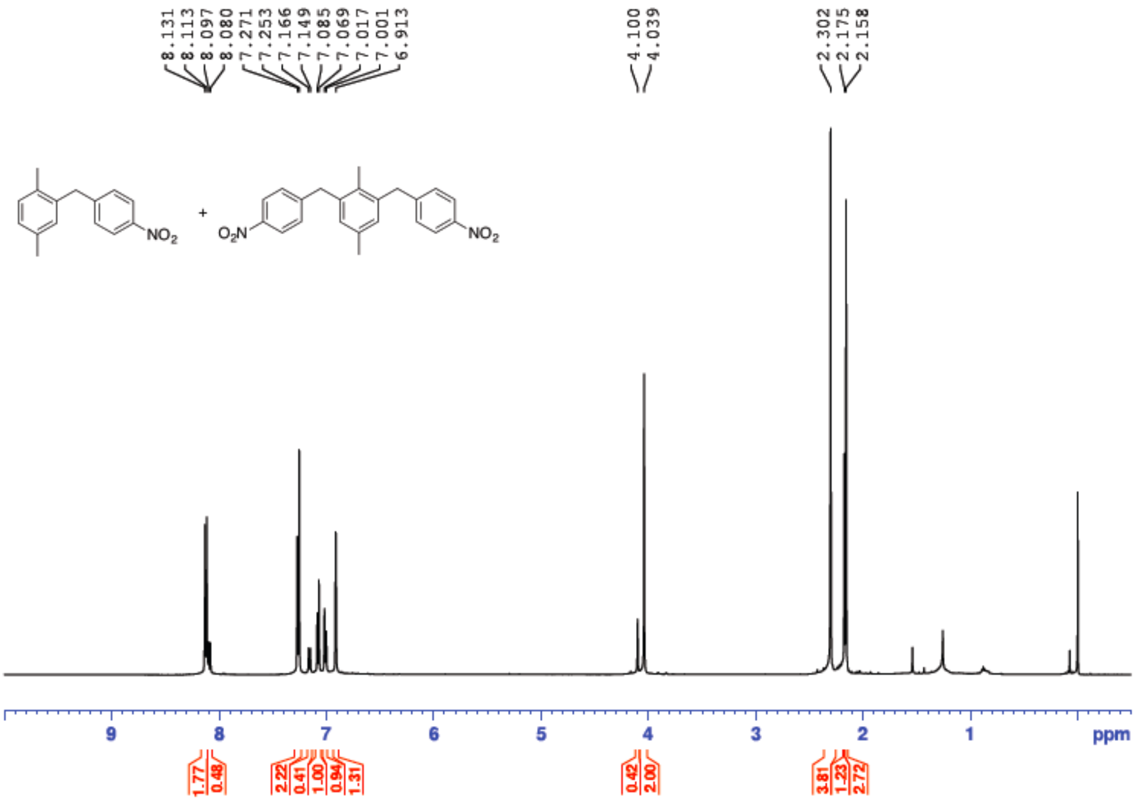
8.131
8.113
8.097
8.080
7.271
7.253
7.166
7.149
7.085
7.069
7.017
7.001
6.913
4.100
4.039
2.302
2.175
2.158
NO_2
+
O_2N
NO_2
9
8
7
6
5
4
3
2
1
ppm
1.77
0.48
2.22
0.41
1.00
0.94
1.31
0.42
2.00
3.81
1.23
2.72

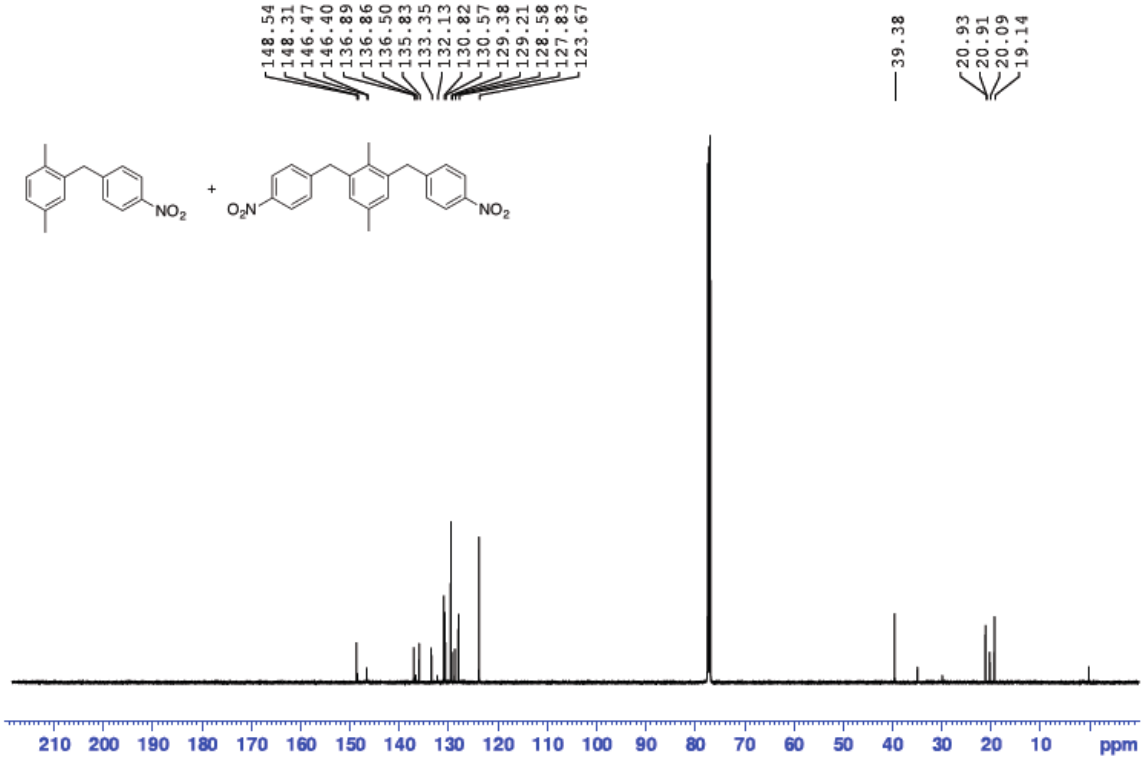
148.54
148.31
146.47
146.40
136.89
136.86
136.50
135.83
133.35
132.13
130.82
130.57
129.38
129.21
128.58
127.83
123.67
39.38
20.93
20.91
20.09
19.14
NO_2
+
O_2N
NO_2
210
200
190
180
170
160
150
140
130
120
110
100
90
80
70
60
50
40
30
20
10
ppm

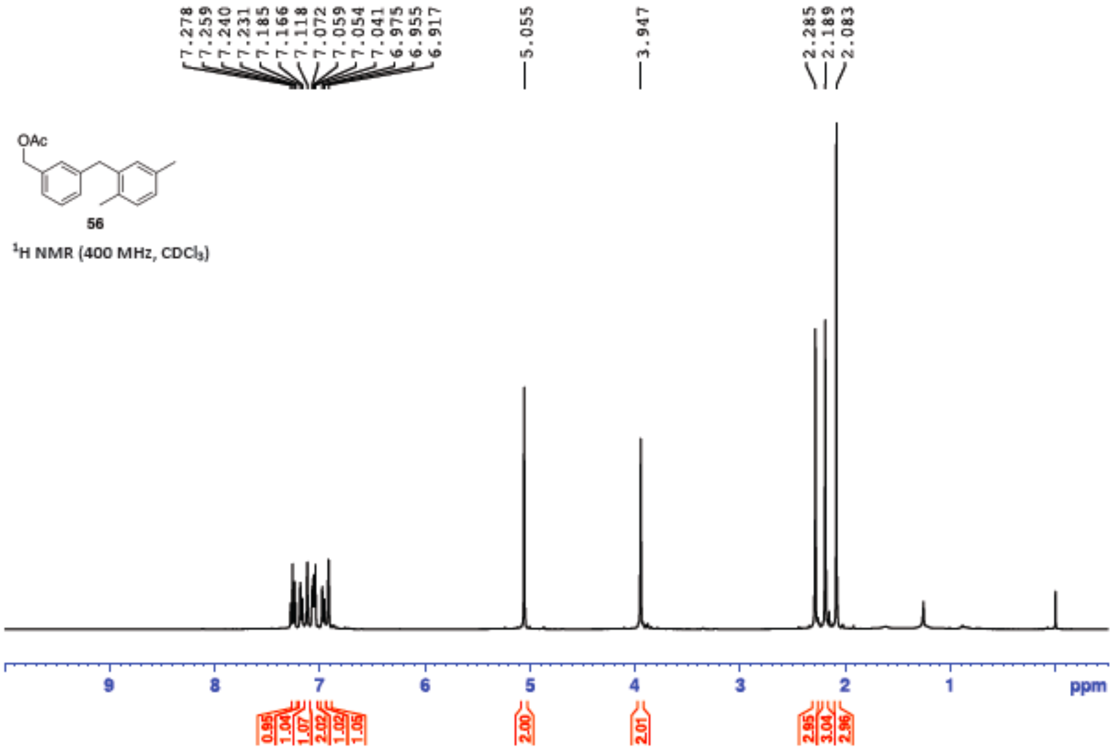
7.278
7.259
7.240
7.231
7.185
7.166
7.118
7.072
7.059
7.054
7.041
6.975
6.955
6.917
5.055
3.947
2.285
2.189
2.083
OAc
56
^{1}H NMR (400 MHz, CDCl$_3$)
9
8
7
6
5
4
3
2
1
ppm
0.95
1.04
1.07
2.02
1.02
1.05
2.00
2.01
2.95
3.04
2.96

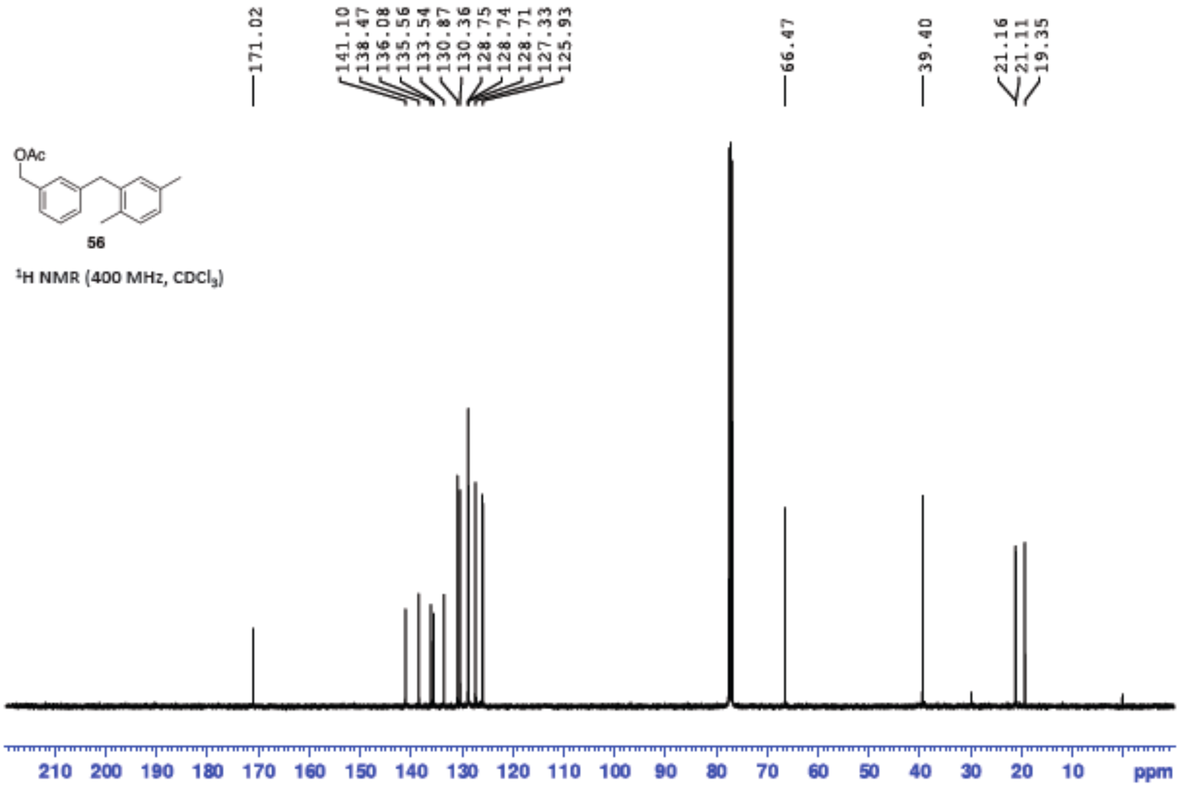
171.02
141.10
138.47
136.08
135.56
133.54
130.87
130.36
128.75
128.74
128.71
127.33
125.93
66.47
39.40
21.16
21.11
19.35
OAc
56
^{1}H NMR (400 MHz, CDCl$_3$)
210
200
190
180
170
160
150
140
130
120
110
100
90
80
70
60
50
40
30
20
10
ppm

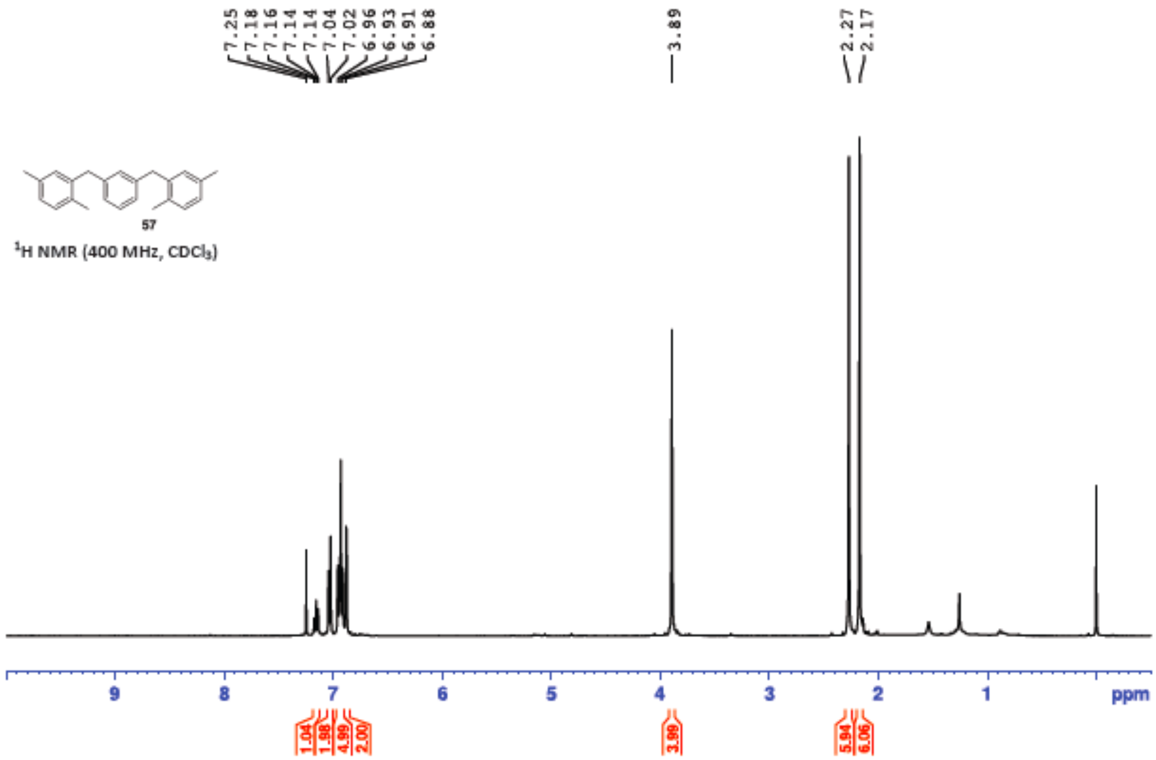
7.25
7.18
7.16
7.14
7.14
7.04
7.02
6.96
6.93
6.91
6.88
3.89
2.27
2.17
57
^1H NMR (400 MHz, $CDCl_3$)
9 8 7 6 5 4 3 2 1 ppm
1.04
1.98
4.99
2.00
3.99
5.94
6.06

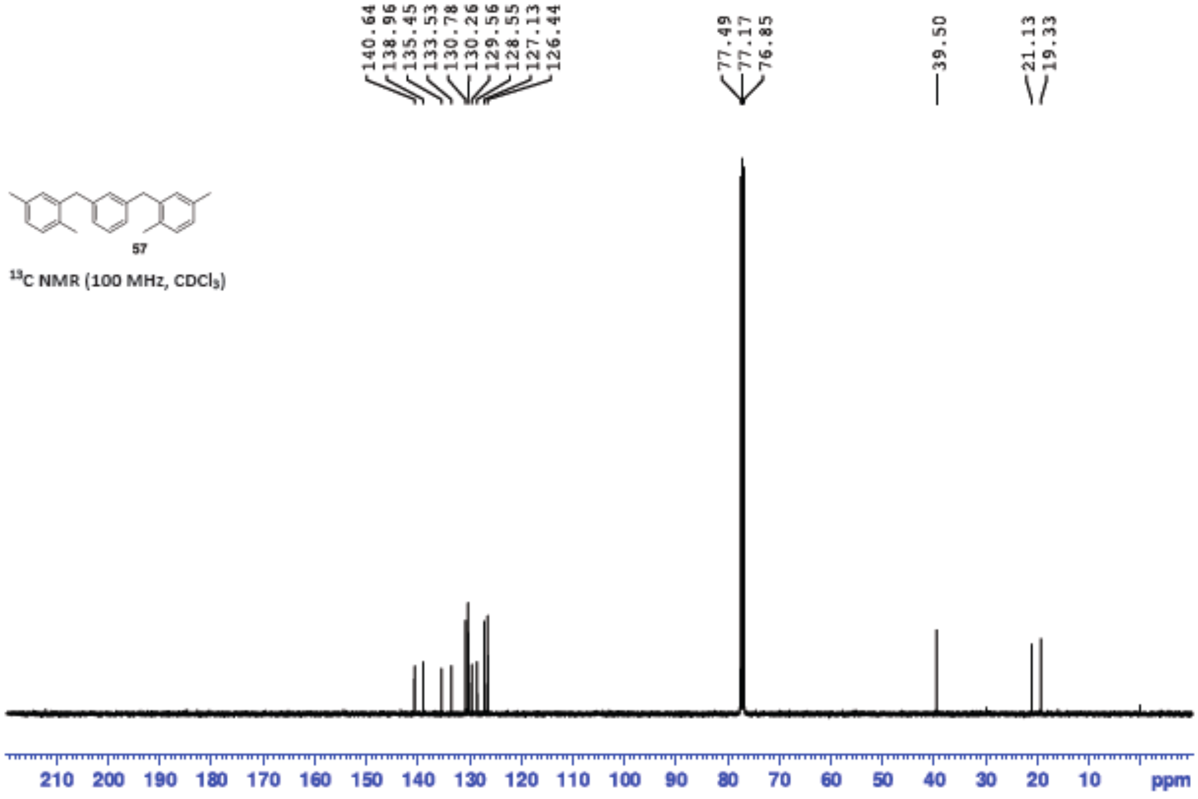
140.64
138.96
135.45
133.53
130.78
130.26
129.56
128.55
127.13
126.44
77.49
77.17
76.85
39.50
21.13
19.33
57
^{13}C NMR (100 MHz, $CDCl_3$)
210 200 190 180 170 160 150 140 130 120 110 100 90 80 70 60 50 40 30 20 10 ppm
